# Identification of *SCARA3* with potential roles in metabolic disorders

**DOI:** 10.18632/aging.202228

**Published:** 2020-12-09

**Authors:** Hui Peng, Qi Guo, Tian Su, Ye Xiao, Chang-Jun Li, Yan Huang, Xiang-Hang Luo

**Affiliations:** 1 Department of Endocrinology, Endocrinology Research Center, Xiangya Hospital of Central South University, Changsha, China

**Keywords:** adipogenesis, obesity, *SCARA3*, methylation, metabolic disorders

## Abstract

Obesity is characterized by the expansion of adipose tissue which is partially modulated by adipogenesis. In the present study, we identified five differentially expressed genes by incorporating two adipogenesis-related datasets from the GEO database and their correlation with adipogenic markers. However, the role of scavenger receptor class A member 3 (*SCARA3*) in obesity-related disorders has been rarely reported. We found that *Scara3* expression in old adipose tissue-derived mesenchymal stem cells (Ad-MSCs) was lower than it in young Ad-MSCs. Obese mice caused by deletion of the leptin receptor gene (*db/db*) or by a high-fat diet both showed reduced *Scara3* expression in inguinal white adipose tissue. Moreover, hypermethylation of *SCARA3* was observed in patients with type 2 diabetes and atherosclerosis. Data from the CTD database indicated that *SCARA3* is a potential target for metabolic diseases. Mechanistically, *JUN* was predicted as a transcriptional factor of *SCARA3* in different databases which is consistent with our further bioinformatics analysis. Collectively, our study suggested that *SCARA3* is potentially associated with age-related metabolic dysfunction, which provided new insights into the pathogenesis and treatment of obesity as well as other obesity-associated metabolic complications.

## INTRODUCTION

Obesity, especially metabolically unhealthy obesity, has been reported to increase the level of glucose and lipids in circulation, resulting in various metabolic disorders, such as cardiovascular diseases, fatty liver and diabetes mellitus [1–4]. With aging populations, the prevalence of obesity has increased significantly worldwide over the past five decades [5–9]. Thus, obesity imposes enormous economic and heath burdens and has become a marked challenge for individuals and public health.

Obesity is characterized by the excessive accumulation of adipose tissue and is associated with adipose oxidative stress [10, 11]. The expansion of adipose tissue is modulated via two distinct mechanisms [1]. On the one hand, adipocyte hyperplasia is characterized by the increase of number of adipocytes mediated by adipogenic differentiation. On the other hand, adipocyte hypertrophy is characterized by adipocyte enlargement [8]. To date, many studies have shown that adipogenic differentiation and subsequent adipocytes maturation play crucial roles in the etiology of obesity, as well as its metabolic complications [8, 12–15]. The expansion of white adipose tissues caused by excessive adipogenesis is found in mice and humans [16–18]. In addition, adipose tissue expansion either in mice induced by a high-fat diet or in leptin receptor-deficient db/db mice, was accompanied by increased adipogenic differentiation [19–21]. Mesenchymal stem cells (MSCs) are multipotent cells with the ability to differentiate into mature cells of several mesenchymal tissues, such as fat and bone [22, 23]. Previously, we identified several factors associated with the lineage commitment of MSCs that showed important impact on metabolic disorders during aging [6, 24–26]. During aging, age-related factors generally contribute to the accumulation of adipose tissue or metabolic dysfunction [6, 26–28]. However, the pathogenesis of obesity during aging mediated by MSCs-associated adipogenesis remains unclear.

In this study, we validated several potential genes (*GHR* (growth hormone receptor), *GPX3* (glutathione peroxidase 3), *SAA1* (serum amyloid A1), *SCARA3* (scavenger receptor class A member 3), and *WFDC1* (WAP four-disulfide core domain 1)) during adipogenesis through the selection of differentially expressed genes (DEGs) and weighted gene co-expression network analysis (WGCNA). Mechanistically, the downregulation of *SCARA3* mediated by methylation is highly likely to promote adipogenesis by regulating *JUN* (Jun proto-oncogene, AP-1 transcription factor subunit) involved transcription factors and peroxisome proliferator activated receptor (PPAR) signaling. Furthermore, *SCARA3* was associated with metabolic dysfunction in both humans and animals in an age-dependent manner. Thus, the results of the present study suggested that *SCARA3* is a potential new target for diagnosis and treatment of obesity, as well as other obesity-associated metabolic complications.

## RESULTS

### Identification of the key modules through WGCNA

**Figure 1:**
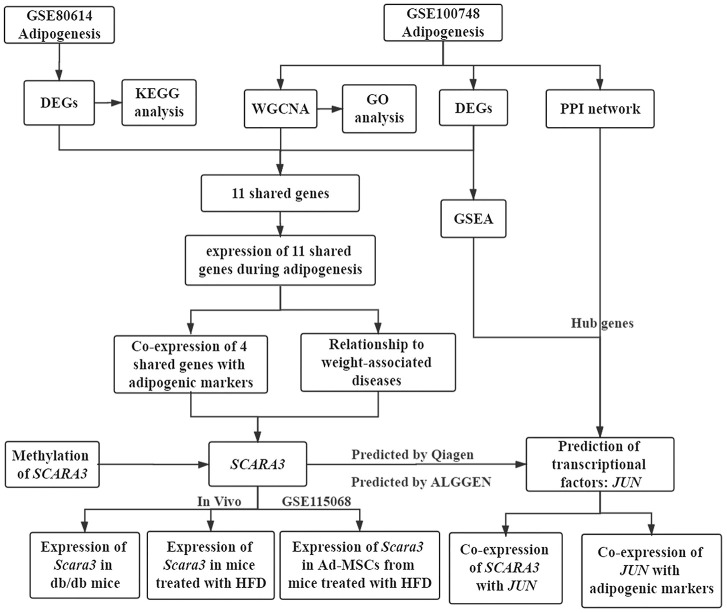
**Workflow of the present study.**

**Figure 2:**
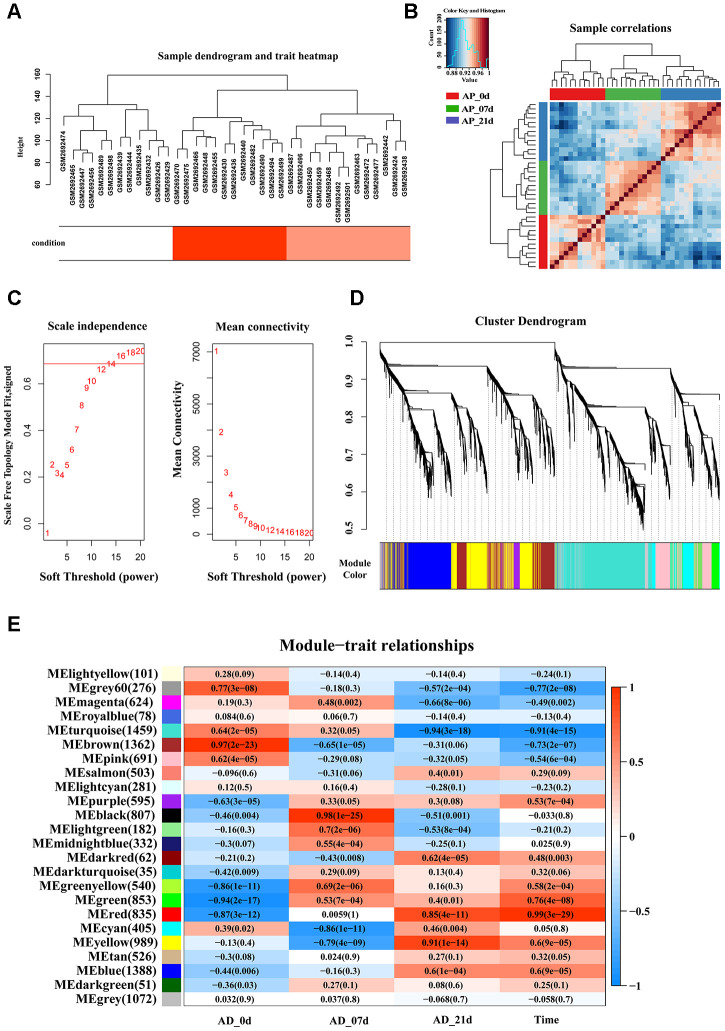
**Identification of key modules that correlate with adipogenesis in the GEO dataset through WGCNA.** (**A**) Sample dendrogram and trait heatmap. (**B**) Sample correlations among three different stages. (**C**) Analysis of the scale-free fit index (left) and the mean connectivity (right) for various soft-thresholding powers based on a scale-free R^2^ (R^2^ = 0.686, power = 14). (**D**) Dendrogram of all genes clustered based on a dissimilarity measure (1-TOM). Each branch in the dendrogram represents one gene and each module color represents one co-expression module. (**E**) Heatmap of the correlation between epigengene module and traits of adipogenesis. Each group contains the correlation coefficient and P value. The digits in the brackets on the left side represent the number of genes in the corresponding epigengene module.

**Figure 3:**
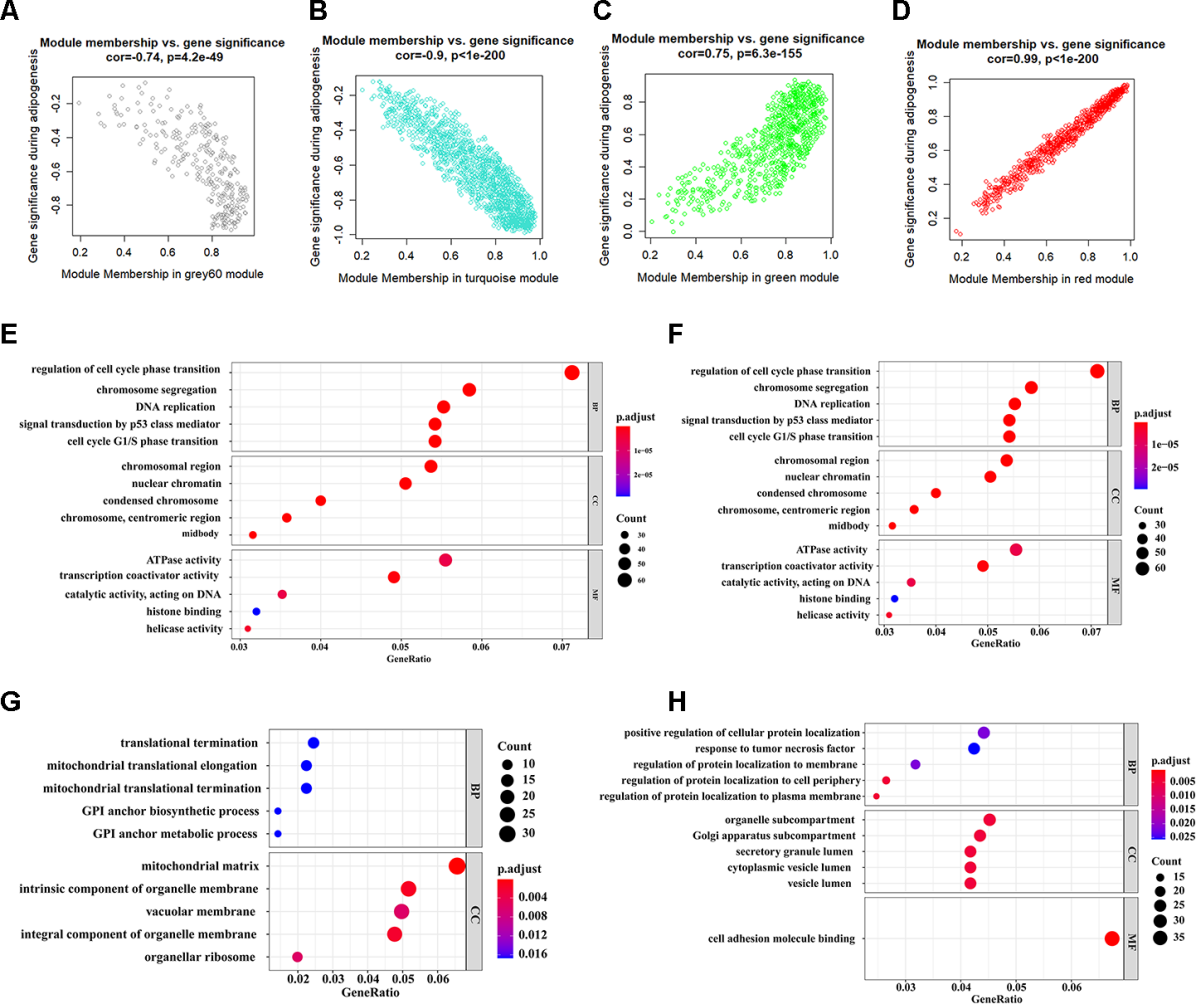
**Identification of functional annotation of the WGCNA module that correlated highly with adipogenesis.** (**A**–**D**) Scatter plot of module Eigengenes in the grey 60 module (**A**), turquoise module (**B**), green module (**C**), and red module (**D**). (**E**–**H**) Biological process GO terms for genes in the grey 60 module (**E**), turquoise module (**F**), green module (**G**), and red module (**H**). P-value cutoff = 0.01; q-value cutoff = 0.05. GO, Gene Ontology.

The workflow of the present study, comprising various bioinformatic data analyses and further validation *in vivo* is shown in Figure 1. To identify the key modules closely associated with adipogenesis, we firstly utilized WGCNA to analyze all genes from the adipogenesis-related dataset (GEO100748), which comprised three different stages of adipogenesis in mesenchymal stem cells (MSCs), such as the undifferentiated stage, day 7 of the differentiated stage and day 21 of the differentiated stage [29]. The sample correlation showed there was no obvious batch effect (Figure 2A, 2B). This dataset has 37 samples, and we selected the soft-thresholding power as 14, according to the instructions of WGCNA (https://horvath.genetics.ucla.edu/html/CoexpressionNetwork/Rpackages/WGCNA/) (Figure 2C). Twenty-four modules were identified based on the average linkage hierarchical clustering and the soft-thresholding power (Figure 2D). The top four modules with most close association with adipogenic stages were grey 60, turquoise, green and red modules (Figure 2E). Thus, we selected these top four modules for further analysis. The four modules contain 276, 1459, 853 and 835 genes, respectively (Supplementary Table 1–4). The grey 60 and turquoise modules were highly positively correlated with the stages of adipogenesis, while the modules of green and red are highly negatively correlated with the stages of adipogenesis (Grey 60: r = -0.74, p = 4.2e-49; Turquoise: r = -0.9, p=1e-200; Green: r = 0.75, p = 6.3e-155; Red: r = 0.99, p = 1e-200) (Figure 3A–3D). The enrichments for the genes in each module were evaluated through GO analysis (Figure 3E–3H).

### Identification of differentially expressed genes (DEGs)

**Figure 4:**
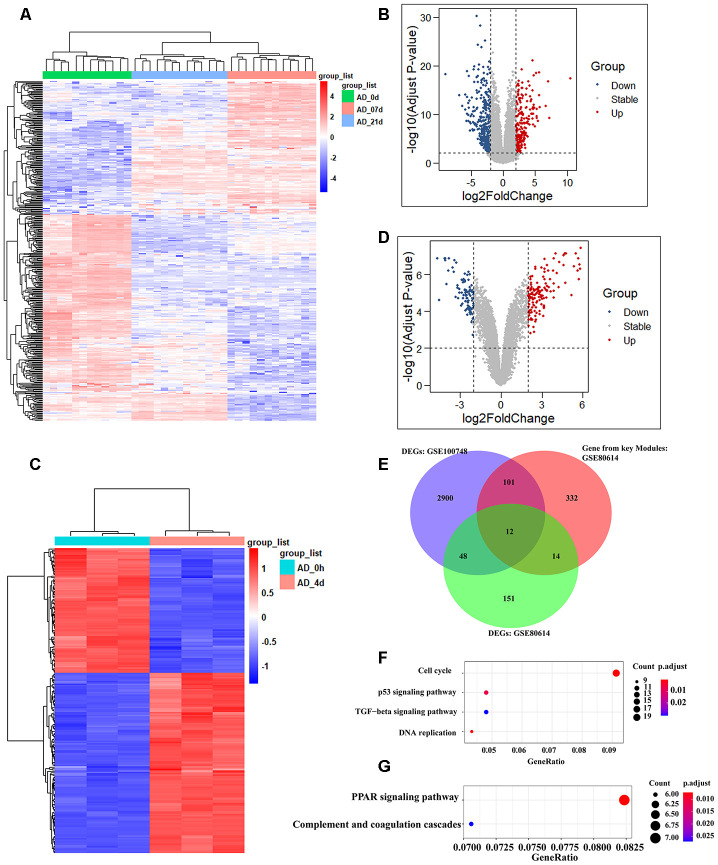
**Identification of shared genes during adipogenesis in two datasets.** (**A**, **B**) Heatmap (**A**) and volcano plot (**B**) of the DEGs from GSE100748 (Adjusted p-value **≤** 0.05 and |logFC| **≥** 2). (**C**, **D**) Heatmap (**C**) and volcano plot (**D**) of the DEGs from GSE80614 (Adjust p-value **≤** 0.05 and |logFC| **≥** 2). (**E**) Venn diagram for shared genes among DEGs and key modules of GSE100748 and DEGs of GSE80614. (**F**) KEGG analysis of DEGs from GSE100748. (**G**) KEGG analysis of DEGs from GSE80614.

**Figure 5:**
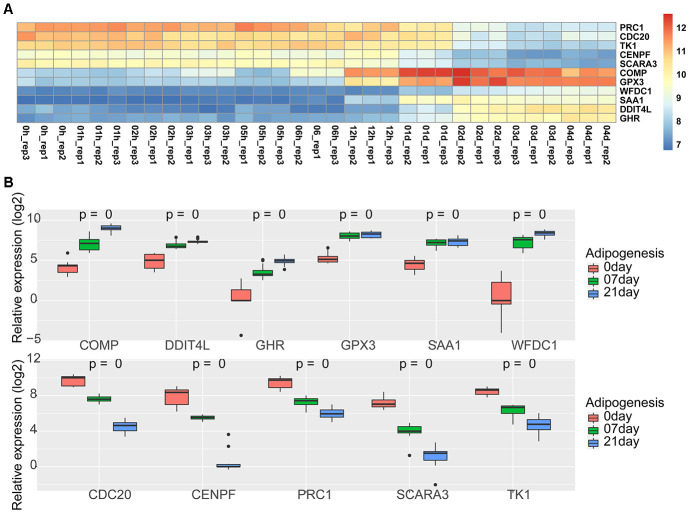
**Expression of 11 shared genes during adipogenesis.** (**A**) Heatmap of the expression of 11 shared genes at different adipogenic stages including the undifferentiated stage, 1 hour, 2 hours, 3 hours, 5 hours, 6 hours, 12 hours, day 1, day 2, day 3, and day 4 (GSE80614). (**B**) Heatmap of the expression of 11 shared genes at different adipogenic stages, including the undifferentiated stage, day 7, and day 21 (GSE100748).

We selected two available adipogenic differentiation datasets from the Gene Expression Omnibus (GEO (GEO100748 and GSE80614) to identify DEGs [29, 30]. The data from GEO100748 showed 1905 DEGs (Supplementary Table 5), and the data from GSE80614 showed 901 DEGs (Supplementary Table 6) (Adjusted p-value ≤ 0.05, |logFC| ≥ 1). We kept retained 459 genes from GEO100748 and 225 genes from GSE80614 whose absolute logFC values were greater than 2 (Figure 4A–4D). The overlapping genes among the key modules analyzed by WGCNA, during adipogenic differentiation from undifferentiating stage to day 4 or day 21, which identified 12 shared DEGs (Figure 4E). KEGG analysis of 459 DEGs from GEO100748 or GSE80614 indicated that the DEGs are highly related to cell cycle or PPAR signaling (Figure 4F, 4G). *CPA4* was removed because of its controversial expression in the two different datasets. The expression of the other 11 genes was demonstrated during the different stages of adipogenic differentiation. The results identified that five shared downregulated genes during adipogenesis including *TK1, SCARA3, PRC1, CENPF* and *CDC20*, as well as six shared upregulated genes *SAA1, WFDC1, GPX3, GHR, DDIT4L* and *COMP* (Figure 5A, 5B).

### The selection of shared key genes during adipogenesis

**Figure 6:**
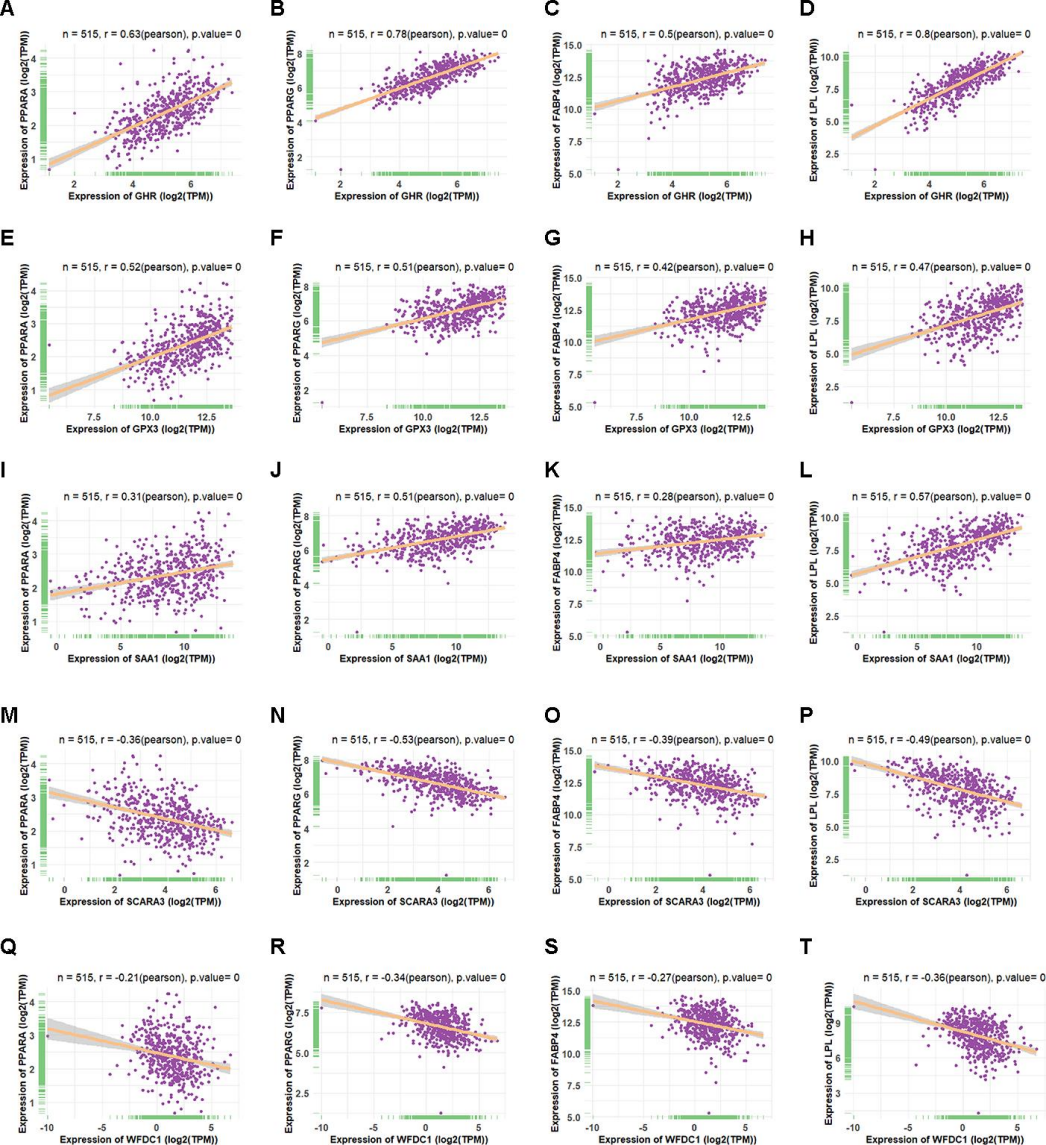
**Co-expression of four shared genes with adipogenic markers in adipose tissue.** (**A**–**D**) Correlation of *GHR* with *PPARA* (**A**)*, PPARG* (**B**)*, FABP4* (**C**), and *LPL* (**D**) expression in adipose tissue, based on data from the Genotype Tissue Expression (GTEx) databases, respectively. (**E**–**H**) Correlation of *GPX3* with *PPARA* (**E**)*, PPARG* (**F**)*, FABP4* (**G**), and *LPL* (**H**) expression in adipose tissue, based on data from the GTEx databases, respectively. (**I**–**L**) Correlation of *SAA1* with *PPARA* (**I**)*, PPARG* (**J**)*, FABP4* (**K**), and *LPL* (**L**) expression in adipose tissue, based on data from the GTEx databases, respectively. (**M**–**P**) Correlation of *SCARA3* with *PPARA* (**M**)*, PPARG* (**N**)*, FABP4* (**O**), and *LPL* (**P**) expression in adipose tissue, based on data from GTEx databases, respectively. (**Q**–**T**) Correlation of *WFDC1* with *PPARA* (**Q**)*, PPARG* (**R**), *FABP4* (**S**), and *LPL* (**T**) expression in adipose tissue, based on data from the GTEx databases, respectively.

**Figure 7:**
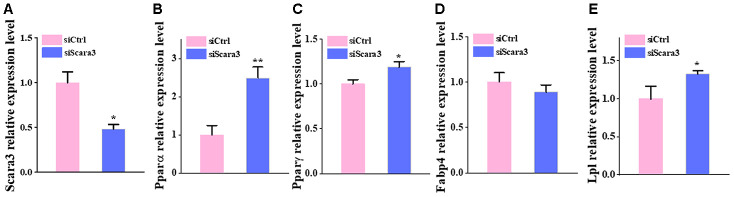
**The deficiency of Scara3 inhibits adipogenic differentiation in vitro.** The Ad-MSCs were transfected with NC siRNA and *Scara3* SiRNA followed by adipogenic differentiation. (**A**) qRT-PCR analysis of depletion of *Scara3*. (**B**–**E**) qRT-PCR analysis of the relative levels of *Pparα* (**B**), *PPARγ* (**C**), *Fabp4* (**D**), and *Lpl* (**E**). Error bars show standard deviation. *P < 0.05, **P < 0.01.

Based on the fact that *PPARG*, *FABP4*, *LPL*, and *PPARA* are crucial markers during adipogenesis [31, 32], we observed the correlation between the retained eleven genes and these four adipogenic markers. We calculated their co-expression based on the adipose tissue data from GTEX and found that only five genes (*GPX3*, *GHR*, *SAA1*, *SCARA3*, and *WFDC1*) correlated significantly with these markers. Moreover, *GPX3*, *GHR* and *SAA1* correlated positively with adipogenic differentiation, while *SCARA3* and *WFDC1* correlated negatively with adipogenic differentiation (Figure 6A–6T). *GPX3*, *GHR* and *SAA1* were removed from further study because they have been reported previously to be associated with obesity [33–35]. *WFDC1* was filtered out because of the controversial result between its gradually increased expression during adipogenesis and its reverse correlation with adipogenic markers. The association of *Scara3* with other four genes was showed in Supplementary Figure 1. During adipogenesis, although the deletion of *Scara3* gene in AD-MSCs did not elevate the expression of *Fabp4* compared to that in control group, it enhanced other adipogenic markers, such as *Pparα*, *Pparγ*, and *Lpl* (Figure 7A–7E). Together, these results verified the prediction that *Scara3* inhibits adipogenesis. Thus, we focused on *SCARA3* (included in turquoise model) for further analysis.

### The prediction of transcription factors of *SCARA3*

**Figure 8:**
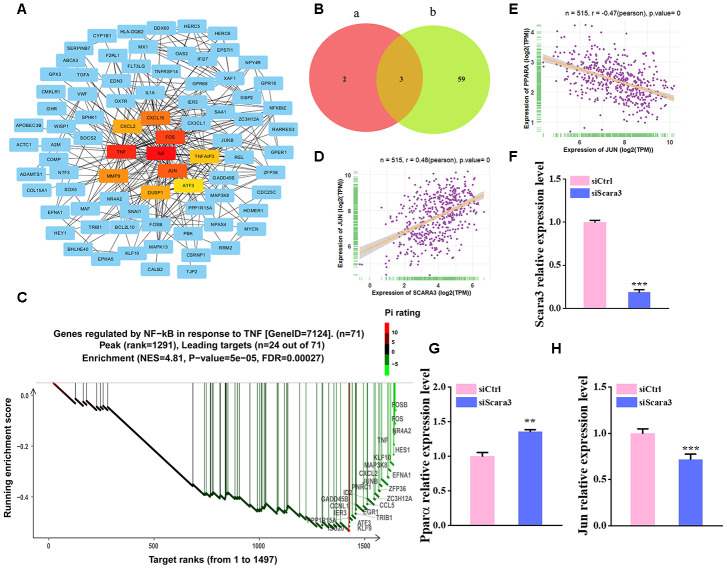
**Selection of the transcription factors of *SCARA3*.** (**A**) PPI network of the DEGs from GSE100748. (**B**) Venn diagram for shared genes through the PROMO (a) and GENECARD (b) databases. (**C**) GSEA analysis of the DEGs from GSE100748. (**D**) Correlation of *SCARA3* with *JUN* in expression in adipose tissue, based on data from GTEx databases. (**E**) Correlation of *JUN* with *PPARA* expression in adipose tissue, based on data from GTEx databases. (**F**) qRT-PCR analysis of depletion of *Scara3*. (**G**–**H**) qRT-PCR analysis of the relative levels of *Pparα* (**G**) and *Jun* (**H**). Error bars show standard deviation. **P < 0.01, ***P < 0.001.

To investigate the regulation of *SCARA3*, we constructed a Protein–protein interaction (PPI) network of adipogenic differentiation-associated DEGs (GSE100748) in the STRING database, and the top 10 hub genes were calculated in Cytoscape (Figure 8A). The result showed that the hub genes are mostly related to the AP-1 complex and *JUN* was ranked top. The PROMO database identified 62 transcription factors for *SCARA3* (Maximum matrix dissimilarity rate = 10%) (Supplementary Figure 2). The top five transcription factors for *SCARA3* by QIAGEN company were AP-1, ATF-2, GATA-1, PPAR-α and PPAR-γ. AP-1 is a heterodimer composed of proteins belonging to the c-FOS, c-Jun, ATF, and JDP families [36]. Thus, there were three shared transcription factors in PROMO database, including c-Jun, GATA-1, and PPAR-α (Figure 8B). Moreover, Gene Set Enrichment Analysis (GSEA) suggested that the signaling, “TNFA signaling via NFKB”, was inhibited during adipogenesis, and the key proteins identified in this signaling pathway also include various components of AP-1 (Figure 8C). We also found that *SCARA3* correlated significantly and positively with *JUN* (Pearson r = 0.48, p value = 0.0) (Figure 8D). Negative correlations of *JUN* with *PPARA* further supported its potential role in adipogenesis (Figure 8E). To further confirm the correlation between *SCARA3* with other genes associated with the signaling regulation, we interfered the *Scara3* gene in AD-MSCs (Figure 8F). Quantitative real-time reverse transcription PCR (qRT-PCR) results were in line with the predicted correlation. With the deficiency of *Scara3*, the expression of *Jun* and *Ppara* were both decreased (Figure 8G, 8H).

### The indications of *SCARA3* for phenotypic traits

**Figure 9:**
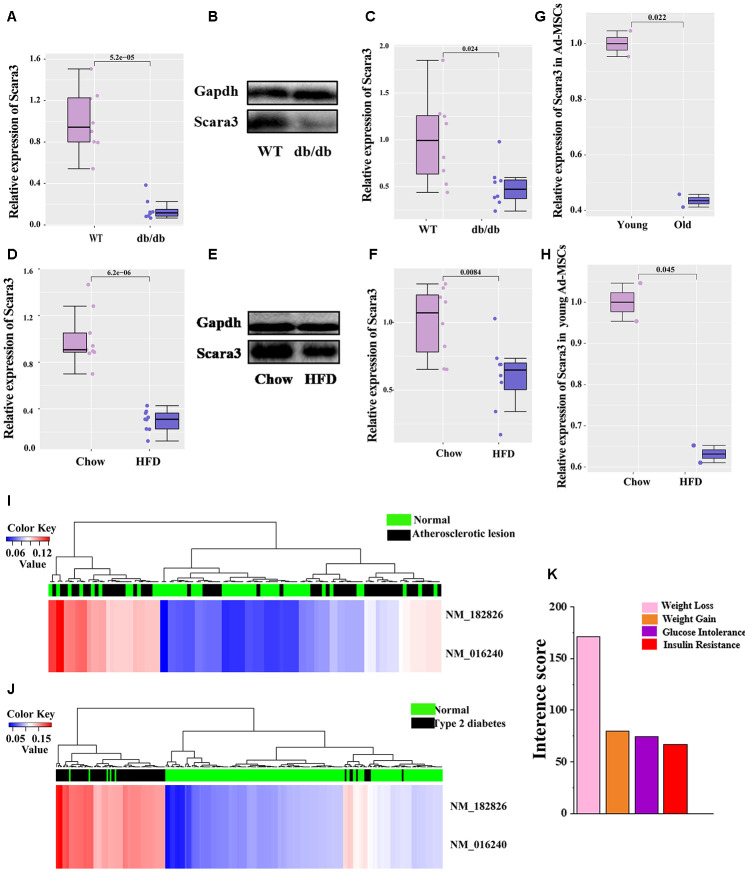
**Phenotypical traits of *SCARA3*.** (**A**) mRNA expression of Scara3 in iWAT of db/db mice. 8 mice were included in each group. (**B**, **C**) Protein expression of Scara3 in iWAT of db/db mice. 8 mice were included in in each group. (**D**) mRNA expression of Scara3 in iWAT of mice fed by HFD. 9 mice were included in each group. (**E**, **F**) Protein expression of Scara3 in iWAT of mice fed by HFD. 8 mice were included in each group. (**G**) Expression of *Scara3* in white adipose tissue-derived mesenchymal stem cells (Ad-MSCs) of young and old mice. Each group has 2 mice, respectively. Data were obtained from GEO database (GSE115068). (**H**) Expression of *Scara3* in Ad-MSCs of young mice fed a HFD. Each group has 2 mice, respectively. Data were obtained from GEO database (GSE115068). (**I**, **J**) Methylation of *SCARA3* in metabolic disorders, including atherosclerotic lesions (**I**) and type 2 diabetes (**J**). (**K**) Relationship of weight-associated diseases with *SCARA3* via a curated chemical interaction, based on the CTD database.

It is well established that db/db mice and mice fed by a high-fat diet (HFD) presents obvious obesity [21, 31, 37–39]. To determine the role of *SCARA3* *in vivo*, we collected the inguinal white adipose tissue (iWAT) from db/db mice and mice fed a HFD or chow control. qRT-PCR and Western-Blot indicated that the expression of *Scara3* decreased considerably in the iWAT in db/db mice compared with that in the controls (Figure 9A–9C). The similar decrease was observed in mice fed by HFD (Figure 9D–9F). To investigate the role of adipose tissue-derived mesenchymal stem cells (Ad-MSCs) in obesity, we analyzed the expression of *Scara3* in Ad-MSCs from GSE115068. Compared with the expression of *Scara3* in young Ad-MSCs, *Scara3* expression was lower in old Ad-MSCs (Figure 9G). HFD stimulus contributed to the downregulation of *Scara3* in young Ad-MSCs (Figure 9H). A previous study reported increased methylation of *SCARA3* in patients with type 2 diabetes mellitus [40]. Here, we also found that *SCARA3* methylation had some correlations with metabolic diseases, such as atherosclerotic lesions and type 2 diabetes via a search on the DiseaseMeth version 2.0 database (Figure 9I, 9J). Thus, we hypothesized that the decreased expression of *SCARA3* during adipogenesis might be caused by its methylation. The Comparative Toxicogenomics Database (CTD; http://ctdbase.org/) provides information about interactions between environmental chemicals and gene products and their relationships to diseases (Figure 9K). We searched the top fifty relationships between *SCARA3* with diseases. Intriguingly, the results implied that *SCARA3* is associated with several metabolic disorders, such as weight loss, weight gain, glucose intolerance and insulin resistance (Figure 9K).

## DISCUSSION

Obesity generally results in predisposition towards other metabolic diseases, which can all result in a decline of life quality and life expectancy [5, 41, 42]. Thus, researchers need to find some therapeutic targets to prevent the development of obesity. Based on the important effect of adipogenesis on obesity, we aimed to find the causes of obesity from the perspective of adipogenesis. Firstly, we identified the overlapping genes in two adipogenic differentiation-associated datasets by performing WGCNA and DEGs selection. We identified five DEGs: *GHR*, *GPX3*, *SAA1*, *SCARA3*, and *WFDC1.* Except for *SCARA3*, other the four genes have already been implicated in the regulation of the pathogenesis of obesity [33–35]. Through further functional analysis, we revealed more indications that *SCARA3* is an important gene during adipogenesis. Combining data form several databases, we further confirmed the possible mechanism mediated by *SCARA3* and potential therapeutic effect of *SCARA3* for obese individuals.

*SCARA3* encodes a macrophage scavenger receptor-like protein. *SCARA3* is ubiquitously expressed in human tissues, although it shows a relatively low expression in the liver and peripheral blood leukocytes [43]. Its ubiquitous expression was also revealed by the data from the GTEX database, which is a comprehensive public repository used to study tissue-specific gene expression and regulation. Cellular stress stimuli at low doses, such as UV radiation and hydrogen peroxide, both remarkably induced the expression of *SCARA3* in human fibroblasts. However, excessive oxidative stress contributed to lower *SCARA3* expression [43]. Thus, it has been reported to be a cellular stress response (CSR) gene with the role of protecting cells from oxidative stress by scavenging oxidative molecules or harmful products of oxidation [44, 45]. In addition, Yu et al. observed that *SCARA3* might be a tumor suppressor-related gene, because down-regulation of *SCARA3* was found in prostate cancer tissues and it is involved in cancers metastases and progression [46]. Obesity and its related disorders, such as diabetes and atherosclerosis, are thought to be related to impaired oxidative defense [10, 47, 48]. A previous study indicated that elevated methylation of *SCARA3* probably disturbed the oxidative stress protection in type 2 diabetes mellitus (T2DM) [40]. Consistent with these observations, our analysis showed that hypermethylation of *SCARA3* was associated with metabolic disorders, including T2DM and atherosclerosis lesions. Considering obesity can result from the environmental stimulus except for inheritable factors, epigenetic alterations, such as methylation, are a considerable cause of obesity [49]. Furthermore, previous studies revealed that promoter methylation contributed to the down-regulation of *SCARA3* in prostate cancer [46, 50]*.* Based on this evidence, we assumed that the downregulation of *SCARA3* during adipogenesis probably resulted from its methylation. The deletion of *Scara3* in Ad-MSCs caused the downregulated expression of adipogenic markers. Notably, we verified the lower expression of *Scara3* in db/db mice and in HFD-fed mice compared with that in the control groups. Consistently, downregulated expression of *Scara3* was tested in Ad-MSCs isolated from young mice fed a HFD. Lower *Scara3* expression was observed in young mice than in old mice. In addition, the data from the CTD database showed that *SCARA3* is associated with several metabolic disorders, such as weight loss, weight gain, glucose intolerance and insulin resistance. Taken together, these results revealed the potentially important roles of *SCARA3* in metabolic disorders.

Previous studies showed that scavenger receptor A gene regulatory elements targeted gene expression to macrophages and foam cells of atherosclerotic lesions [51, 52]. Furthermore, Horvai et al. demonstrated that scavenger receptor A gene regulatory elements contain binding sites for PU.1 and AP-1 in bone marrow progenitor cells [52]. In the present study, we predicted the transcription factors for *SCARA3* using different databases and found the activator protein 1 (AP-1) was shared in the different databases. AP-1 is a heterodimer composed of proteins belonging to the c-FOS, c-Jun, ATF, and JDP families, which is involved in various cellular processes, such as cell differentiation, proliferation and apoptosis [36, 53]. PPAR signaling and nuclear factor κB (NF-κB) pathway were both reported to be related to obesity [42, 54–56]. The filtered DEGs expressed during adipogenesis enriched in “TNFA signaling via NFKB” by GSEA, and several genes at the leading edge (*FOS*, *JUN, JUNB* and *ATF3*) mostly belonged to the AP-1 heterodimer. Moreover, the hub genes selected from the PPI network, such as *JUN*, *FOS* and *ATF3*, also suggested that the AP-1 heterodimer plays a major role in adipogenesis. C-Jun levels were observed to be decreased during adipogenic differentiation in MC3T3 cells and inhibited adipogenic differentiation [57, 58]. Based on this evidence, we supposed that *SCARA3* could bind to c-Jun and AP-1, thereby controlling adipogenic differentiation.

Collectively, we hypothesized that *SCARA3* contributes to obesity and obesity-related metabolic complications in an age-dependent manner. Specifically, *SCARA3* downregulation was possibly a consequence of its methylation. Mechanistically, the dysfunction of adipogenic differentiation might be associated with impaired modulation between *SCARA3* and *JUN*. Further studies will be worthy of being performed to validate the role of *SCARA3*, such as the phenotypical observation of *Scara3* knock-out mice and the methylation of *SCARA3* promoter in mice with obesity. Other function studies, such as Chromatin Immunoprecipitation (ChIP)-PCR and luciferase reporter assay, can further verify *SCARA3* gene regulates transcriptional factor JUN and PPAR signaling. Together, the present study combining bioinformatic analysis with the several biochemical functional analyses in vitro and *in vivo*, provided strong supports for the hypothesis that *SCARA3* is a potential target for the treatment of obesity and other metabolic disorders.

## Materials and Methods

### Identification of key genes using WGCNA method

The R package “WGCNA” was used to find the key modules and genes related to adipogenesis [59]. Firstly, we input the raw expression of matrix from GSE100748 and transformed it into the log2 format. Then, we tested the missing values and removed the data with low quality. After the genes with zero variance were filtered out, we generated the sampleTree to check the outliers. Secondly, we normalized the data through quantile normalization and generated a heatmap of sample correlations. Thirdly, we constructed the network and detected the modules using an automatic, one-step method. The adjacency matrix was transformed into topological overlap matrix (TOM). According to the TOM-based dissimilarity measure, we set the soft-thresholding power as 14 (scale free R2 = 0.686), the minimal module size as 30, and cut height as 0.25 to identify key modules. Although the scale free R2 did not reach 0.8 when the power was 14, we considered this was because of the obvious biological difference between different stages of adipogenesis. Based on the number of samples (37 samples in total), we finally set the power as 14. Finally, we calculated the correlation between modules and traits during adipogenesis. Further data analysis based on the top four modules (grey 60, turquoise, green and red modules) during adipogenesis was performed.

### Identification of DEGs

The series matrix files of GSE100748 and GSE80614 from GEO were downloaded. The data at different adipogenic stages from GSE100748 including the undifferentiated stage, day 7 and day 21 were retained. The data at different adipogenic stages from GSE80614 including the undifferentiated stage, and hour 1-6, hour 12 and day 1-4 were retained. To filter the DEGs, we only analyzed the expression at the undifferentiated stage and day 4. Firstly, we input the selected raw expression data of the matrix and transform it into the log2 format if needed. Secondly, we normalized data through quantile normalization. The R package “limma” [60] was applied to normalize the data and identify DEGs. The DEGs was retained if they had an adjust p-value ≤ 0.05 and |logFC| ≥ 1. Further data analysis we used the DEGs with an adjust p-value ≤ 0.05 and |logFC| ≥ 2 except for the GSEA analysis. Then, we exported the heatmap and volcano plot of DEGs with adjusted p-value ≤ 0.05 and |logFC| ≥ 2.

### Functional enrichment analyses

Gene Ontology (GO) analyses and Kyoto Encyclopedia of Genes and Genomes (KEGG) pathway analyses were carried out using the R package “Clusterprofiler” [61]. We used DEGs (adjusted p-value <= 0.05 and |logFC| ≥ 2) from GSE80614, and transformed them into the format of ENTREZ ID. Then we use the R package “Clusterprofiler” to evaluate the enriched signaling (P value Cutoff = 0.05, Show Category = 5). R/Bioconductor package “Pi” was used to perform xPierGSEA analysis to find the pathways most associated with the hub genes. Here, the DEGs with |logFC| ≤ 1 were selected as our targeted gene set. The range size was set from 20 to 5000.

### Construction of protein–protein interaction (PPI) networks of DEGs

STRING (http://string-db.org/) is a database of known and predicted protein-protein interactions. It was applied to predict the interacting genes among the DGEs using the default settings. The associated file of the network was imported into the Cytoscape software to visualize the network. The hub genes were identified using the plug-in cytohubba by setting the top node as 10 ranked by Degree.

### Co-expression calculation

The raw expression data of genes and clinical phenotypes were downloaded from the GTEx program (https://www.gtexportal.org/). Then, we removed the data without associated clinical information. Further analysis then focused on correlations in adipose tissue. The Pearson correlation (r) and p-value were calculated.

### Methylation and gene expression analyses

The human disease methylation database, DiseaseMeth version 2.0, is a web-based resource focusing on the aberrant methylomes of human diseases [62] (http://bioinfo.hrbmu.edu.cn/diseasemeth/). We utilized DiseaseMeth 2.0 to search the methylation levels of *SCARA3* related to metabolic disorders.

### CTD

The Comparative Toxicogenomics Database (CTD) illuminates how environmental chemicals affect human health [63] (http://ctdbase.org/). This database was used to validate *SCARA3* associations with weight-related diseases and metabolic disorders. Here, we selected our diseases of interested only among the top 50 relationships, such as weight loss, weight gain, glucose intolerance, and insulin resistance.

### Mice

Male C57BL/6J mice at 3 months of age were fed a HFD or chow control for four months before the start of the experiment to induce obesity. Db/db mice were used in our experiments at 12 weeks of age. All mice were maintained in a standard, specific pathogen-free facility of the Laboratory Animal Research Center of Central South University. All procedures involving mice were approved by the Animal Ethics Committee of the Central South University.

### qRT-PCR analysis

Total RNA from the iWAT of mice or cells was extracted using Trizol reagent (Takara) as described previously [6, 64]. RNA (1000 ng) was reverse-transcribed into first-strand cDNA using a Reverse Transcription Kit (Takara). qPCR was then performed using SYBR Green PCR Master Mix (Takara) and mRNA expression was normalized to the expression of the reference gene *Gapdh*.

### Western blot

1 mg adipose tissue was lysed in the mixture of RIPA (100 ul) and protease inhibitor (1:100). Western Blot was performed according to the previous described method [26]. The primary antibodies, Monoclonal Anti-SCARA3 antibody produced in mouse (#WH0051435M1-100UG; Sigma-Aldrich, MO, USA), and GAPDH (TA802519; ORIGENE), were incubated overnight at 4° C, then incubated with appropriate secondary antibodies for 1 hour at room temperature. The blots were visualized using ECL detection reagents.

### Cell isolation and culture

Ad-MSCs were isolated from iWAT of 1-month male C57BL/6J mice. Firstly, we isolated the iWAT from mice. Then, the tissue was cut and digested using 0.1% I type Collagenase (Gibco,17108-029), a mixture of the collagenases I in PBS. Then it was incubated in 37° C for 30 min and followed by centrifuge at 1000 rpm 5 min. The cells were then cultured in DMEM containing 10% fetal bovine serum, 100 U/mL penicillin, and 100 μg/ mL streptomycin. Culture medium was changed every other day. Cells were collected for cell transfection, adipogenic differentiation or RNA extraction.

### Cell transfection

The *Scara3* siRNA and the negative control (NC) were purchased from Ribibio (Guangzhou, China). The siRNAs were transfected at the concentration of 100 nM using lipofectamine 2000 (Invitrogen, USA) according to manufacturer's recommendations.

### Adipogenic differentiation

Adipogenic differentiation was conducted as described previously [25]. MSCs were treated with DMEM containing 10% fetal bovine serum, 100 U/mL penicillin, 100 μg/ mL streptomycin, 0.5 mM 3-isobutyl-1-methylxanthine, 5 μg/ml insulin, and 1 μM dexamethasone for 4 days. The medium was changed every other day.

### Statistical analysis

All data were analyzed using R and R studio software (Version 4.0.1) except for GSEA analysis (Version 3.6.3). Key modules were selected with the power as 14 (scale free R2 = 0.686), the minimal module size as 30, and the cut height as 0.25 using package “WGCNA”. DEGs were selected with adjusted P < 0.05 and |logFC| > 1 through the package “limma”. GO terms or KEGG pathways with adjusted P < 0.05 were considered statistically significant. GSEA was generated using the package “Pi”. Pearson correlation analysis was adopted to determine the linear relationship between the two groups. For the animal experiment, each group comprised ten mice. The animals were randomly divided into two groups including mice fed a HFD and those fed a control chow diet. qRT-PCR was performed independently three times and all results are expressed as means ± standard deviation (SD). Quantitative data from qRT-PCR, Western Blot or the expression of *Scara3* in white adipose tissue derived mesenchymal stem cells (Ad-MSCs) from the GEO database were imported into a spreadsheet and scaled and normalized to their appropriate controls using R. Two-way, paired or unpaired t-tests were performed using the package “ggplot”.

### Availability of data and materials

The raw data of the two adipogenesis-related microarray datasets are available in the GEO database (https://www.ncbi.nlm.nih.gov/geo/. Accession number: GSE100748 and GSE80614.). The data for *Scara3* expression in Ad-MSCs are available in the GEO database (Accession number: GSE115068). The transcription factors were predicted in the PROMO database (http://alggen.lsi.upc.es/cgi-bin/promo_v3/promo/promoinit.cgi?dirDB=TF_8.3). Associations of SCARA3 with metabolic disorders are available in CTD (http://ctdbase.org/). The methylation of *SCARA3* in patients with metabolic disorders is available in DiseaseMeth version 2.0 (http://bio-bigdata.hrbmu.edu.cn/diseasemeth/).

## Supplementary Material

Supplementary Figures

Supplementary Table 1

Supplementary Table 2

Supplementary Table 3

Supplementary Table 4

Supplementary Table 5

Supplementary Table 6
