# Supplementary material for: Identification of SCARA3 with potential roles in metabolic disorders
Source: Aging (Albany NY). 2020 Dec 9;13(2):2149–67. doi: 10.18632/aging.202228 (PMC7880357; doi:10.18632/aging.202228)
Supplement: Supplementary Figures [file aging-13-202228-s001.pdf]

SUPPLEMENTARY FIGURES

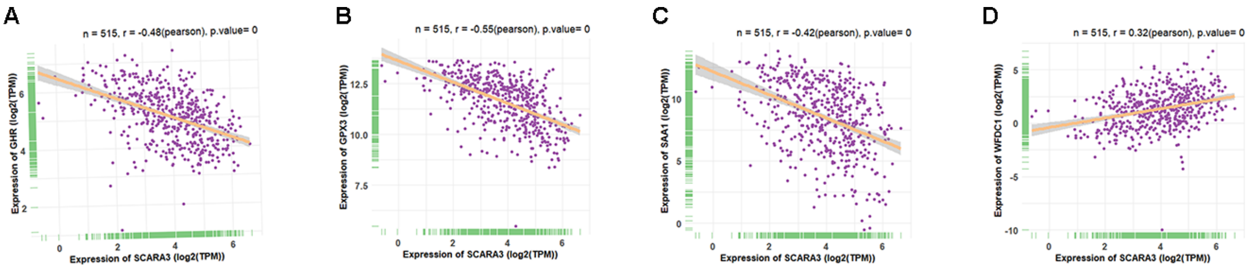

Supplementary Figure 1. Co-expression of *SCARA3* with four shared genes in adipose tissue. (**A**–**D**) Correlation of *SCARA3* with *GHR* (**A**), *GPX3* (**B**), *SAA1* (**C**), and *WFDC1* (**D**) expression in adipose tissue, based on data from the GTEx databases, respectively.

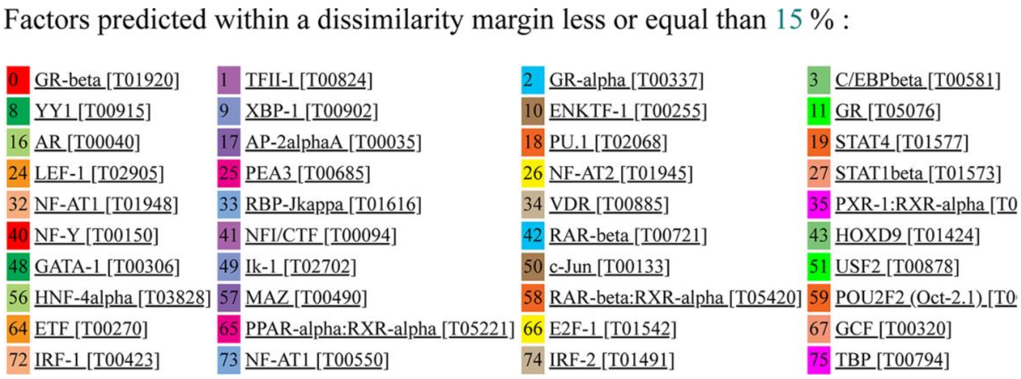

Factors predicted within a dissimilarity margin less or equal than 15 % :

0 GR-beta [T01920]	1 TFII-I [T00824]	2 GR-alpha [T00337]	3 C/EBPbeta [T00581]
8 YY1 [T00915]	9 XBP-1 [T00902]	10 ENKTF-1 [T00255]	11 GR [T05076]
16 AR [T00040]	17 AP-2alphaA [T00035]	18 PU.1 [T02068]	19 STAT4 [T01577]
24 LEF-1 [T02905]	25 PEA3 [T00685]	26 NF-AT2 [T01945]	27 STAT1beta [T01573]
32 NF-AT1 [T01948]	33 RBP-Jkappa [T01616]	34 VDR [T00885]	35 PXR-1:RXR-alpha [T0
40 NF-Y [T00150]	41 NFI/CTF [T00094]	42 RAR-beta [T00721]	43 HOXD9 [T01424]
48 GATA-1 [T00306]	49 Ik-1 [T02702]	50 c-Jun [T00133]	51 USF2 [T00878]
56 HNF-4alpha [T03828]	57 MAZ [T00490]	58 RAR-beta:RXR-alpha [T05420]	59 POU2F2 (Oct-2.1) [T0
64 ETF [T00270]	65 PPAR-alpha:RXR-alpha [T05221]	66 E2F-1 [T01542]	67 GCF [T00320]
72 IRF-1 [T00423]	73 NF-AT1 [T00550]	74 IRF-2 [T01491]	75 TBP [T00794]

Supplementary Figure 2. Predictions of transcriptional factors of *SCARA3* in PROMO databases.
