# Supplementary material for: Identification of SCARA3 with potential roles in metabolic disorders
Source: Aging (Albany NY). 2020 Dec 9;13(2):2149–67. doi: 10.18632/aging.202228 (PMC7880357; doi:10.18632/aging.202228)
Supplement: Supplementary Table 1 [file aging-13-202228-s002.docx]

Supplementary Table 1: Association with adipogenic stages in Grey 60 model.

| moduleGenes | MM | MMP | GS | GSP |
| --- | --- | --- | --- | --- |
| 1 CDC25B | 0.962289 | 2.26E-21 | -0.75163 | 8.25E-08 |
| 2 WDR37 | 0.957988 | 1.45E-20 | -0.74093 | 1.57E-07 |
| 3 ADA | 0.955287 | 4.21E-20 | -0.77149 | 2.28E-08 |
| 4 MNT | 0.953524 | 8.17E-20 | -0.84689 | 3.93E-11 |
| 5 EFS | 0.952339 | 1.26E-19 | -0.69757 | 1.60E-06 |
| 6 GTF2IRD1 | 0.952321 | 1.27E-19 | -0.70899 | 9.05E-07 |
| 7 CBX4 | 0.951369 | 1.77E-19 | -0.84137 | 6.98E-11 |
| 8 ZBTB17 | 0.943367 | 2.39E-18 | -0.73055 | 2.85E-07 |
| 9 C15ORF39 | 0.943028 | 2.65E-18 | -0.80848 | 1.43E-09 |
| 10 CRAMP1L | 0.94219 | 3.40E-18 | -0.80803 | 1.48E-09 |
| 11 ISGF3G | 0.941901 | 3.70E-18 | -0.76057 | 4.70E-08 |
| 12 PHF15 | 0.941749 | 3.87E-18 | -0.71376 | 7.07E-07 |
| 13 IFITM3 | 0.940294 | 5.89E-18 | -0.76309 | 3.99E-08 |
| 14 LY6E | 0.932227 | 5.07E-17 | -0.69794 | 1.57E-06 |
| 15 EP300 | 0.930971 | 6.92E-17 | -0.73344 | 2.42E-07 |
| 16 TKT | 0.930086 | 8.59E-17 | -0.81687 | 7.00E-10 |
| 17 TRIM25 | 0.929864 | 9.06E-17 | -0.71095 | 8.18E-07 |
| 18 RBM9 | 0.927594 | 1.55E-16 | -0.86039 | 8.76E-12 |
| 19 SRPX | 0.925525 | 2.50E-16 | -0.87958 | 7.72E-13 |
| 20 RAD9A | 0.924403 | 3.22E-16 | -0.77718 | 1.54E-08 |
| 21 SLC25A29 | 0.919363 | 9.57E-16 | -0.87733 | 1.05E-12 |
| 22 CCDC23 | 0.918766 | 1.08E-15 | -0.76692 | 3.10E-08 |
| 23 POLR1D | 0.918336 | 1.18E-15 | -0.89442 | 8.72E-14 |
| 24 TNFRSF21 | 0.916019 | 1.90E-15 | -0.67174 | 5.32E-06 |
| 25 TP53I11 | 0.915989 | 1.91E-15 | -0.60405 | 7.54E-05 |
| 26 OSBPL7 | 0.91316 | 3.33E-15 | -0.80277 | 2.27E-09 |
| 27 TSPYL2 | 0.912942 | 3.47E-15 | -0.84583 | 4.40E-11 |
| 28 NUMA1 | 0.911759 | 4.35E-15 | -0.81645 | 7.27E-10 |
| 29 HYOU1 | 0.907574 | 9.47E-15 | -0.5897 | 0.000123 |
| 30 IER5 | 0.907124 | 1.03E-14 | -0.92715 | 1.72E-16 |
| 31 CEBPB | 0.905979 | 1.26E-14 | -0.90333 | 2.01E-14 |
| 32 ANPEP | 0.90399 | 1.79E-14 | -0.58685 | 0.000135 |
| 33 HLA-A | 0.903053 | 2.10E-14 | -0.90797 | 8.81E-15 |
| 34 GCHFR | 0.901917 | 2.56E-14 | -0.82822 | 2.52E-10 |
| 35 VPS37B | 0.900781 | 3.10E-14 | -0.51457 | 0.001121 |
| 36 KLF13 | 0.90024 | 3.39E-14 | -0.90153 | 2.73E-14 |
| 37 ILF3 | 0.897897 | 5.00E-14 | -0.90706 | 1.04E-14 |
| 38 BIN1 | 0.896211 | 6.56E-14 | -0.51283 | 0.001173 |
| 39 TOP1MT | 0.894176 | 9.07E-14 | -0.76158 | 4.40E-08 |
| 40 DTX3 | 0.894127 | 9.14E-14 | -0.54644 | 0.000467 |
| 41 SSBP4 | 0.893698 | 9.77E-14 | -0.7853 | 8.64E-09 |
| 42 RNF31 | 0.892948 | 1.10E-13 | -0.70294 | 1.23E-06 |
| 43 KIAA0195 | 0.892754 | 1.13E-13 | -0.73178 | 2.66E-07 |
| 44 H1FX | 0.892607 | 1.16E-13 | -0.82889 | 2.36E-10 |
| 45 NINJ1 | 0.892529 | 1.17E-13 | -0.83934 | 8.57E-11 |
| 46 IFITM1 | 0.890945 | 1.50E-13 | -0.89469 | 8.36E-14 |
| 47 LOC153222 | 0.890287 | 1.65E-13 | -0.68325 | 3.16E-06 |
| 48 CYP2S1 | 0.890276 | 1.65E-13 | -0.80665 | 1.66E-09 |

| 49 PSME1 | 0.889983 | | 1.73E-13 | -0.7412 | 1.55E-07 |  |
| --- | --- | --- | --- | --- | --- | --- |
| 50 DKFZP761I2123 | 0.889589 | | 1.84E-13 | -0.83751 | 1.03E-10 |  |
| 51 PERLD1 | 0.889268 | | 1.93E-13 | -0.85281 | 2.07E-11 |  |
| 52 TMEM51 | 0.886955 | | 2.71E-13 | -0.75249 | 7.83E-08 |  |
| 53 PSCD1 | 0.886923 | | 2.73E-13 | -0.94549 | 1.25E-18 |  |
| 54 CHCHD5 | 0.885989 | | 3.12E-13 | -0.61175 | 5.75E-05 |  |
| 55 SERPINB1 | 0.885188 | | 3.51E-13 | -0.69215 | 2.08E-06 |  |
| 56 GPX1 | 0.884026 | | 4.14E-13 | -0.62003 | 4.26E-05 |  |
| 57 ZP4 | 0.88267 | | 5.02E-13 | -0.67536 | 4.52E-06 |  |
| 58 COMMD4 | 0.882067 | | 5.47E-13 | -0.62345 | 3.76E-05 |  |
| 59 ZNF598 | 0.881658 | | 5.79E-13 | -0.82219 | 4.38E-10 |  |
| 60 MSX1 | 0.880577 | | 6.73E-13 | -0.90398 | 1.79E-14 |  |
| 61 MTMR3 | 0.87878 | | 8.61E-13 | -0.53131 | 0.000715 |  |
| 62 TMEM85 | 0.877523 | | 1.02E-12 | -0.68472 | 2.95E-06 |  |
| 63 ATP5G2 | 0.875178 | | 1.39E-12 | -0.80081 | 2.66E-09 |  |
| 64 KIAA0913 | 0.87513 | | 1.40E-12 | -0.75383 | 7.20E-08 |  |
| 65 IFITM2 | 0.874776 | | 1.47E-12 | -0.88449 | 3.88E-13 |  |
| 66 ING3 | 0.871804 | | 2.16E-12 | -0.8059 | 1.77E-09 |  |
| 67 TBX3 | 0.871365 | | 2.29E-12 | -0.86108 | 8.07E-12 |  |
| 68 LTBP4 | 0.870986 | | 2.40E-12 | -0.67954 | 3.75E-06 |  |
| 69 CD99 | 0.870098 | | 2.69E-12 | -0.66474 | 7.21E-06 |  |
| 70 FAM100B | 0.869647 | | 2.85E-12 | -0.50986 | 0.001266 |  |
| 71 TMEM88 | 0.86905 | | 3.07E-12 | -0.69709 | 1.64E-06 |  |
| 72 ATBF1 | 0.867881 | | 3.55E-12 | -0.70618 | 1.04E-06 |  |
| 73 ETS2 | 0.867094 | | 3.91E-12 | -0.85801 | 1.15E-11 |  |
| 74 SNAI2 | 0.86473 | | 5.22E-12 | -0.93038 | 8.01E-17 |  |
| 75 IGF2BP3 | 0.863425 | | 6.11E-12 | -0.40003 | 0.014159 |  |
| 76 ATXN7L2 | 0.863408 | | 6.12E-12 | -0.80843 | 1.43E-09 |  |
| 77 CX3CL1 | 0.861413 | | 7.76E-12 | -0.80915 | 1.35E-09 |  |
| 78 IFI6 | 0.861392 | | 7.78E-12 | -0.90793 | 8.87E-15 |  |
| 79 SRRM2 | 0.860781 | | 8.36E-12 | -0.5864 | 0.000137 |  |
| 80 MTMR4 | 0.860436 | | 8.71E-12 | -0.83571 | 1.23E-10 |  |
| 81 THRAP4 | 0.859349 | | 9.88E-12 | -0.57963 | 0.00017 |  |
| 82 ATF5 | 0.858596 | | 1.08E-11 | -0.7379 | 1.88E-07 |  |
| 83 ENG | 0.858497 | | 1.09E-11 | -0.49198 | 0.001983 |  |
| 84 HLA-H | 0.857428 | | 1.23E-11 | -0.85171 | 2.34E-11 |  |
| 85 UNC93B1 | 0.857323 | | 1.25E-11 | -0.43461 | 0.007189 |  |
| 86 TMEM35 | 0.85652 | | 1.37E-11 | -0.87864 | 8.77E-13 |  |
| 87 ISG20 | 0.856301 | | 1.40E-11 | -0.9028 | 2.20E-14 |  |
| 88 SPEN | 0.855913 | | 1.47E-11 | -0.90754 | 9.52E-15 |  |
| 89 WDR58 | 0.854946 | | 1.63E-11 | -0.88605 | 3.10E-13 |  |
| 90 DUS3L | 0.854203 | | 1.78E-11 | -0.72965 | 3.00E-07 |  |
| 91 C18ORF56 | 0.853019 | | 2.03E-11 | -0.93196 | 5.42E-17 |  |
| 92 NKD2 | 0.852898 | | 2.05E-11 | -0.77546 | 1.74E-08 |  |
| 93 FOXF2 | 0.85234 | | 2.18E-11 | -0.85944 | 9.78E-12 |  |
| 94 SRGAP1 | 0.851288 | | 2.45E-11 | -0.67388 | 4.83E-06 |  |
| 95 ZNF384 | 0.85021 | | 2.76E-11 | -0.74578 | 1.18E-07 |  |
| 96 BCL3 | 0.849964 | | 2.83E-11 | -0.52311 | 0.000894 |  |
| 97 MAP3K11 | 0.84995 | | 2.83E-11 | -0.40552 | 0.012772 |  |
| 98 NNAT | 0.849877 | | 2.86E-11 | -0.91675 | 1.64E-15 |  |
| 99 CLN3 | | 0.849397 | | 3.01E-11 | -0.47618 | 0.002891 |
| 100 MBD6 | | 0.849139 | | 3.09E-11 | -0.85613 | 1.43E-11 |
| 101 PRDM1 | | 0.846968 | | 3.90E-11 | -0.88625 | 3.01E-13 |
| 102 ZBED4 | | 0.846608 | | 4.05E-11 | -0.87536 | 1.36E-12 |
| 103 NUP214 | | 0.845268 | | 4.67E-11 | -0.60069 | 8.46E-05 |
| 104 ZDHHC7 | | 0.845158 | | 4.72E-11 | -0.61299 | 5.50E-05 |
| 105 COL6A2 | | 0.841721 | | 6.73E-11 | -0.74741 | 1.07E-07 |
| 106 DUSP10 | | 0.841297 | | 7.03E-11 | -0.5167 | 0.00106 |
| 107 TP53I3 | | 0.840373 | | 7.72E-11 | -0.50635 | 0.001386 |
| 108 ACVRL1 | | 0.83911 | | 8.77E-11 | -0.85765 | 1.20E-11 |
| 109 CCDC59 | | 0.838132 | | 9.67E-11 | -0.6046 | 7.39E-05 |
| 110 TUBB3 | | 0.837925 | | 9.87E-11 | -0.78944 | 6.37E-09 |
| 111 PITPNC1 | | 0.835596 | | 1.24E-10 | -0.76055 | 4.71E-08 |
| 112 FBXW7 | | 0.83533 | | 1.28E-10 | -0.6535 | 1.16E-05 |
| 113 MGC7036 | | 0.832416 | | 1.69E-10 | -0.85404 | 1.81E-11 |
| 114 LSS | | 0.829299 | | 2.28E-10 | -0.7368 | 2.00E-07 |
| 115 ZBTB5 | | 0.82908 | | 2.32E-10 | -0.55646 | 0.000348 |
| 116 PSCD2 | | 0.828392 | | 2.48E-10 | -0.85625 | 1.41E-11 |
| 117 PRIC285 | | 0.827339 | | 2.73E-10 | -0.9174 | 1.44E-15 |
| 118 JARID2 | | 0.827149 | | 2.78E-10 | -0.79689 | 3.62E-09 |
| 119 XRCC1 | | 0.825718 | | 3.17E-10 | -0.68874 | 2.45E-06 |
| 120 UAP1L1 | | 0.825072 | | 3.37E-10 | -0.41538 | 0.010569 |
| 121 SLC9A8 | | 0.822183 | | 4.38E-10 | -0.6459 | 1.58E-05 |
| 122 TRAF4 | | 0.821714 | | 4.57E-10 | -0.87795 | 9.64E-13 |
| 123 C9ORF86 | | 0.820687 | | 5.01E-10 | -0.41186 | 0.011317 |
| 124 ACIN1 | | 0.820071 | | 5.29E-10 | -0.39521 | 0.015481 |
| 125 SRC | | 0.820031 | | 5.31E-10 | -0.38681 | 0.018029 |
| 126 CYP27A1 | | 0.819735 | | 5.45E-10 | -0.52879 | 0.000766 |
| 127 NPTX1 | | 0.81763 | | 6.56E-10 | -0.87168 | 2.20E-12 |
| 128 ARL9 | | 0.813278 | | 9.55E-10 | -0.88469 | 3.77E-13 |
| 129 HERC5 | | 0.812033 | | 1.06E-09 | -0.91077 | 5.25E-15 |
| 130 DCP1A | | 0.811196 | | 1.14E-09 | -0.68755 | 2.59E-06 |
| 131 KLP1 | | 0.810007 | | 1.26E-09 | -0.39864 | 0.01453 |
| 132 DACT1 | | 0.809859 | | 1.27E-09 | -0.91601 | 1.90E-15 |
| 133 ZZEF1 | | 0.80739 | | 1.56E-09 | -0.70436 | 1.14E-06 |
| 134 SOX9 | | 0.804716 | | 1.94E-09 | -0.48907 | 0.002129 |
| 135 INSIG1 | | 0.804492 | | 1.98E-09 | -0.91032 | 5.71E-15 |
| 136 C11ORF56 | | 0.803077 | | 2.22E-09 | -0.61668 | 4.82E-05 |
| 137 ASB6 | | 0.802642 | | 2.30E-09 | -0.73315 | 2.46E-07 |
| 138 DMAP1 | | 0.802429 | | 2.34E-09 | -0.86764 | 3.66E-12 |
| 139 RAB38 | | 0.80165 | | 2.49E-09 | -0.67149 | 5.37E-06 |
| 140 METAP1 | | 0.800619 | | 2.70E-09 | -0.49058 | 0.002052 |
| 141 SAP130 | | 0.799669 | | 2.91E-09 | -0.93605 | 1.89E-17 |
| 142 RIN1 | | 0.798818 | | 3.11E-09 | -0.66387 | 7.49E-06 |
| 143 LEPREL2 | | 0.798641 | | 3.16E-09 | -0.77373 | 1.96E-08 |
| 144 GTF3C5 | | 0.797083 | | 3.56E-09 | -0.77835 | 1.42E-08 |
| 145 STK11IP | | 0.796266 | | 3.80E-09 | -0.50103 | 0.001585 |
| 146 RFXANK | | 0.796182 | | 3.82E-09 | -0.66263 | 7.90E-06 |
| 147 C18ORF8 | | 0.795795 | | 3.94E-09 | -0.56628 | 0.000259 |
| 148 MLL4 | | 0.793964 | | 4.53E-09 | -0.58294 | 0.000153 |
| 149 PCNXL2 | | 0.789661 | | 6.27E-09 | -0.36487 | 0.026392 |
| 150 PLD2 | | 0.78903 | | 6.57E-09 | -0.67681 | 4.24E-06 |
| 151 ENTPD6 | | 0.788495 | | 6.84E-09 | -0.42554 | 0.008646 |
| 152 C1ORF159 | | 0.787863 | | 7.16E-09 | -0.47093 | 0.003264 |
| 153 POLE4 | | 0.787339 | | 7.44E-09 | -0.37887 | 0.020755 |
| 154 USP30 | | 0.786297 | | 8.04E-09 | -0.64219 | 1.83E-05 |
| 155 ABCA5 | | 0.785678 | | 8.41E-09 | -0.57794 | 0.00018 |
| 156 CASP1 | | 0.784477 | | 9.18E-09 | -0.76506 | 3.51E-08 |
| 157 CART1 | | 0.783743 | | 9.68E-09 | -0.64963 | 1.36E-05 |
| 158 FLJ25222 | | 0.779805 | | 1.28E-08 | -0.48509 | 0.002342 |
| 159 ZNF385 | | 0.77903 | | 1.36E-08 | -0.60285 | 7.86E-05 |
| 160 AIP | | 0.778752 | | 1.38E-08 | -0.33466 | 0.042908 |
| 161 MGC33212 | | 0.778541 | | 1.40E-08 | -0.44401 | 0.005908 |
| 162 UBAP2 | | 0.774601 | | 1.85E-08 | -0.67125 | 5.43E-06 |
| 163 MRPS30 | | 0.773677 | | 1.97E-08 | -0.47901 | 0.002705 |
| 164 ETFB | | 0.77341 | | 2.00E-08 | -0.44658 | 0.005593 |
| 165 H1F0 | | 0.771458 | | 2.29E-08 | -0.32713 | 0.048121 |
| 166 SFRS8 | | 0.771113 | | 2.34E-08 | -0.36719 | 0.025378 |
| 167 IBRDC3 | | 0.769889 | | 2.54E-08 | -0.34063 | 0.039115 |
| 168 TNRC5 | | 0.76838 | | 2.82E-08 | -0.39152 | 0.016561 |
| 169 KIAA1815 | | 0.767892 | | 2.91E-08 | -0.54386 | 0.000503 |
| 170 LOC349114 | | 0.762799 | | 4.07E-08 | -0.45449 | 0.004715 |
| 171 AATF | | 0.760873 | | 4.61E-08 | -0.33886 | 0.040211 |
| 172 ARHGEF17 | | 0.758589 | | 5.34E-08 | -0.65563 | 1.06E-05 |
| 173 IL17RC | | 0.755919 | | 6.32E-08 | -0.36659 | 0.025637 |
| 174 BMP5 | | 0.75401 | | 7.12E-08 | -0.57929 | 0.000172 |
| 175 SLC29A3 | | 0.751851 | | 8.14E-08 | -0.4488 | 0.005333 |
| 176 PLEKHF1 | | 0.743483 | | 1.35E-07 | -0.47057 | 0.00329 |
| 177 STAU1 | | 0.742307 | | 1.45E-07 | -0.60547 | 7.17E-05 |
| 178 VANGL2 | | 0.74169 | | 1.50E-07 | -0.63253 | 2.67E-05 |
| 179 DPP4 | | 0.735726 | | 2.13E-07 | -0.64428 | 1.68E-05 |
| 180 PLA2G7 | | 0.735508 | | 2.15E-07 | -0.61778 | 4.63E-05 |
| 181 RPS6KA1 | | 0.732749 | | 2.52E-07 | -0.57627 | 0.00019 |
| 182 SFRS4 | | 0.729082 | | 3.09E-07 | -0.53191 | 0.000704 |
| 183 CUTA | | 0.726657 | | 3.54E-07 | -0.36461 | 0.026508 |
| 184 KIAA0415 | | 0.723874 | | 4.12E-07 | -0.34085 | 0.038975 |
| 185 AKAP8L | | 0.722357 | | 4.48E-07 | -0.32521 | 0.049525 |
| 186 C3ORF14 | | 0.720912 | | 4.84E-07 | -0.50225 | 0.001537 |
| 187 CORO1A | | 0.71493 | | 6.65E-07 | -0.55834 | 0.000329 |
| 188 BCL9L | | 0.709447 | | 8.84E-07 | -0.34128 | 0.038713 |
| 189 CORO7 | | 0.707941 | | 9.55E-07 | -0.37282 | 0.023052 |
| 190 CENTG2 | | 0.690958 | | 2.20E-06 | -0.49756 | 0.001728 |
| 191 ZNF205 | | 0.689267 | | 2.39E-06 | -0.38939 | 0.017211 |
| 192 AP4M1 | | 0.68346 | | 3.13E-06 | -0.61542 | 5.04E-05 |
| 193 C11ORF35 | | 0.680632 | | 3.56E-06 | -0.43034 | 0.007847 |
| 194 NDUFB11 | | 0.677387 | | 4.13E-06 | -0.49905 | 0.001666 |
| 195 KLHL26 | | 0.669784 | | 5.79E-06 | -0.51026 | 0.001253 |
| 196 YTHDF2 | | 0.668153 | | 6.22E-06 | -0.50958 | 0.001275 |
| 197 UPF3A | | 0.668082 | | 6.24E-06 | -0.52615 | 0.000824 |
| 198 STAT6 | | 0.660603 | | 8.61E-06 | -0.44235 | 0.006119 |
| 199 DHDH | | 0.66045 | 8.66E-06 | | -0.53302 | 0.000682 |
| 200 RAB33A | | 0.659305 | 9.09E-06 | | -0.47421 | 0.003026 |
| 201 ABCB9 | | 0.651926 | 1.24E-05 | | -0.40773 | 0.012249 |
| 202 TH | | 0.651688 | 1.25E-05 | | -0.37452 | 0.022385 |
| 203 EID3 | | 0.648944 | 1.40E-05 | | -0.54281 | 0.000518 |
| 204 SHF | | 0.645264 | 1.62E-05 | | -0.45879 | 0.004289 |
| 205 SYT14 | | 0.643955 | 1.71E-05 | | -0.56468 | 0.000272 |
| 206 ANKRD13B | | 0.64051 | 1.96E-05 | | -0.45939 | 0.004233 |
| 207 MTA1 | | 0.639629 | 2.03E-05 | | -0.37849 | 0.020892 |
| 208 C6ORF85 | | 0.636772 | 2.26E-05 | | -0.35824 | 0.029469 |
| 209 IFI30 | | 0.635388 | 2.39E-05 | | -0.40364 | 0.013235 |
| 210 LMAN2 | | 0.629162 | 3.03E-05 | | -0.51569 | 0.001088 |
| 211 RRAGC | | 0.625433 | 3.49E-05 | | -0.36814 | 0.024973 |
| 212 STXBP5 | | 0.620686 | 4.16E-05 | | -0.54322 | 0.000512 |
| 213 BAX | | 0.620548 | 4.18E-05 | | -0.39568 | 0.015347 |
| 214 LOC401357 | | 0.619917 | 4.28E-05 | | -0.62788 | 3.18E-05 |
| 215 UGCGL1 | | 0.60021 | 8.61E-05 | | -0.58593 | 0.000139 |
| 216 GLCCI1 | | 0.589017 | 0.000125 | | -0.39023 | 0.016951 |
| 217 NOXA1 | | 0.584915 | 0.000144 | | -0.4672 | 0.003553 |
| 218 GOLGA2 | | 0.578076 | 0.000179 | | -0.36867 | 0.024747 |
| 219 ARMC5 | | 0.575176 | 0.000196 | | -0.36168 | 0.027838 |
| 220 STX17 | | 0.569786 | 0.000232 | | -0.48463 | 0.002368 |
| 221 CASP4 | | 0.548165 | 0.000444 | | -0.46265 | 0.003937 |
| 222 MSI2 | | 0.546111 | 0.000472 | | -0.38612 | 0.018254 |
| 223 LTV1 | | 0.545549 | 0.000479 | | -0.43755 | 0.006765 |
| 224 ZNF232 | | 0.539609 | 0.000568 | | -0.53614 | 0.000626 |
| 225 CD96 | | 0.538261 | 0.00059 | | -0.54894 | 0.000434 |
| 226 PORCN | | 0.519148 | 0.000993 | | -0.38513 | 0.01858 |
| 227 IL8 | | 0.496903 | 0.001757 | | -0.36105 | 0.028131 |
| 228 PHF20 | | 0.491243 | 0.002019 | | -0.32688 | 0.048303 |
| 229 TMEM185B | | 0.482949 | 0.002465 | | -0.41083 | 0.011543 |
| 230 KCNQ1 | | 0.471698 | 0.003206 | | -0.43696 | 0.006849 |
| 231 TMEM86B | | 0.455641 | 0.004598 | | -0.33387 | 0.043433 |
| 232 ATP5S | | 0.455477 | 0.004614 | | -0.33927 | 0.039955 |
| 233 KCTD14 | | 0.376395 | 0.02167 | | -0.33002 | 0.046062 |
