# Supplementary material for: Identification of SCARA3 with potential roles in metabolic disorders
Source: Aging (Albany NY). 2020 Dec 9;13(2):2149–67. doi: 10.18632/aging.202228 (PMC7880357; doi:10.18632/aging.202228)
Supplement: Supplementary Table 2 [file aging-13-202228-s003.docx]

Supplementary Table 2: Association with adipogenic stages in turquoise model.

| moduleGenes | MM | MMP | GS | GSP |
| --- | --- | --- | --- | --- |
| 1 CCNE2 | 0.979202 | 7.75E-26 | -0.89773 | 5.14E-14 |
| 2 RYBP | 0.975882 | 1.01E-24 | -0.8779 | 9.70E-13 |
| 3 ANKRD50 | 0.975679 | 1.17E-24 | -0.89976 | 3.68E-14 |
| 4 SVEP1 | 0.973535 | 5.03E-24 | -0.94882 | 4.25E-19 |
| 5 ARL5B | 0.972629 | 9.00E-24 | -0.92551 | 2.51E-16 |
| 6 BNC2 | 0.97036 | 3.56E-23 | -0.87367 | 1.70E-12 |
| 7 H2AFZ | 0.969513 | 5.79E-23 | -0.90766 | 9.31E-15 |
| 8 FBL | 0.967747 | 1.53E-22 | -0.87165 | 2.21E-12 |
| 9 RING1 | 0.966451 | 3.02E-22 | -0.89692 | 5.85E-14 |
| 10 SMAD6 | 0.96626 | 3.33E-22 | -0.85592 | 1.46E-11 |
| 11 SNRK | 0.965816 | 4.17E-22 | -0.90948 | 6.68E-15 |
| 12 PIK3R2 | 0.965339 | 5.29E-22 | -0.87218 | 2.06E-12 |
| 13 C19ORF48 | 0.96531 | 5.37E-22 | -0.90349 | 1.95E-14 |
| 14 VPS37D | 0.963951 | 1.04E-21 | -0.89743 | 5.40E-14 |
| 15 TOX | 0.962965 | 1.66E-21 | -0.87389 | 1.65E-12 |
| 16 POLR1E | 0.962765 | 1.82E-21 | -0.90509 | 1.47E-14 |
| 17 NASP | 0.962724 | 1.85E-21 | -0.93207 | 5.27E-17 |
| 18 FBXL10 | 0.962206 | 2.35E-21 | -0.88506 | 3.58E-13 |
| 19 CDC2 | 0.961923 | 2.67E-21 | -0.95407 | 6.68E-20 |
| 20 KIF15 | 0.961262 | 3.59E-21 | -0.94451 | 1.69E-18 |
| 21 NCOA1 | 0.960507 | 5.00E-21 | -0.82968 | 2.20E-10 |
| 22 SMC4 | 0.959468 | 7.81E-21 | -0.92813 | 1.37E-16 |
| 23 EFNB3 | 0.958763 | 1.05E-20 | -0.87058 | 2.53E-12 |
| 24 NUSAP1 | 0.958252 | 1.30E-20 | -0.94784 | 5.89E-19 |
| 25 ZFHX4 | 0.95806 | 1.40E-20 | -0.89891 | 4.23E-14 |
| 26 RCC2 | 0.958001 | 1.44E-20 | -0.78871 | 6.73E-09 |
| 27 CDT1 | 0.95776 | 1.59E-20 | -0.96458 | 7.67E-22 |
| 28 SUMO2 | 0.95761 | 1.69E-20 | -0.89364 | 9.87E-14 |
| 29 TRMT5 | 0.956987 | 2.17E-20 | -0.84383 | 5.42E-11 |
| 30 PRRX1 | 0.956614 | 2.51E-20 | -0.94183 | 3.78E-18 |
| 31 FLJ25476 | 0.956299 | 2.84E-20 | -0.8261 | 3.06E-10 |
| 32 FLJ40629 | 0.95589 | 3.34E-20 | -0.97343 | 5.39E-24 |
| 33 ID1 | 0.955641 | 3.67E-20 | -0.89674 | 6.03E-14 |
| 34 COL5A1 | 0.955063 | 4.59E-20 | -0.87733 | 1.05E-12 |
| 35 HMGB2 | 0.954507 | 5.66E-20 | -0.91237 | 3.87E-15 |
| 36 CCNF | 0.95409 | 6.62E-20 | -0.90518 | 1.45E-14 |
| 37 MND1 | 0.951939 | 1.45E-19 | -0.95023 | 2.64E-19 |
| 38 CXXC1 | 0.951149 | 1.92E-19 | -0.81536 | 7.99E-10 |
| 39 C8ORF72 | 0.95094 | 2.06E-19 | -0.89294 | 1.10E-13 |
| 40 CDC20 | 0.950927 | 2.07E-19 | -0.97847 | 1.41E-25 |
| 41 PUM1 | 0.950667 | 2.27E-19 | -0.9252 | 2.70E-16 |
| 42 PPM1D | 0.950621 | 2.30E-19 | -0.79554 | 4.01E-09 |
| 43 NRP1 | 0.950598 | 2.32E-19 | -0.94817 | 5.27E-19 |
| 44 RBMX | 0.950306 | 2.57E-19 | -0.94153 | 4.13E-18 |
| 45 CCNA2 | 0.950303 | 2.57E-19 | -0.94571 | 1.16E-18 |
| 46 BRD2 | 0.949401 | 3.50E-19 | -0.95542 | 4.00E-20 |
| 47 SIX5 | 0.949235 | 3.70E-19 | -0.96088 | 4.25E-21 |
| 48 DDX48 | 0.948986 | 4.02E-19 | -0.93962 | 7.13E-18 |

| 49 RCOR2 | 0.94771 | | 6.14E-19 | -0.77752 | | 1.51E-08 |  |  |
| --- | --- | --- | --- | --- | --- | --- | --- | --- |
| 50 CASP2 | 0.947508 | | 6.55E-19 | -0.85526 | | 1.58E-11 |  |  |
| 51 C20ORF129 | 0.947502 | | 6.56E-19 | -0.93842 | | 9.95E-18 |  |  |
| 52 TMEM97 | 0.947236 | | 7.16E-19 | -0.85014 | | 2.78E-11 |  |  |
| 53 BIRC5 | 0.947065 | | 7.56E-19 | -0.949 | | 4.01E-19 |  |  |
| 54 NEK2 | 0.946796 | | 8.25E-19 | -0.94286 | | 2.79E-18 |  |  |
| 55 C21ORF66 | 0.946713 | | 8.47E-19 | -0.83912 | | 8.76E-11 |  |  |
| 56 MCM7 | 0.946237 | | 9.86E-19 | -0.96043 | | 5.16E-21 |  |  |
| 57 COL18A1 | 0.945884 | | 1.10E-18 | -0.86324 | | 6.25E-12 |  |  |
| 58 ABHD8 | 0.945783 | | 1.14E-18 | -0.81554 | | 7.86E-10 |  |  |
| 59 NR4A2 | 0.945417 | | 1.28E-18 | -0.97048 | | 3.31E-23 |  |  |
| 60 VGLL4 | 0.944954 | | 1.47E-18 | -0.92885 | | 1.16E-16 |  |  |
| 61 ANKRD10 | 0.944259 | | 1.83E-18 | -0.87534 | | 1.36E-12 |  |  |
| 62 E2F2 | 0.944136 | | 1.90E-18 | -0.90642 | | 1.16E-14 |  |  |
| 63 AUTS2 | 0.94413 | | 1.90E-18 | -0.92036 | | 7.76E-16 |  |  |
| 64 C22ORF18 | 0.944112 | | 1.91E-18 | -0.94502 | | 1.44E-18 |  |  |
| 65 HCAP-G | 0.942989 | | 2.68E-18 | -0.95241 | | 1.23E-19 |  |  |
| 66 SMARCA4 | 0.942866 | | 2.78E-18 | -0.925 | | 2.81E-16 |  |  |
| 67 CENPF | 0.942799 | | 2.84E-18 | -0.94172 | | 3.90E-18 |  |  |
| 68 TNFRSF19 | 0.942376 | | 3.22E-18 | -0.8917 | | 1.33E-13 |  |  |
| 69 ASF1B | 0.94198 | | 3.62E-18 | -0.88428 | | 4.00E-13 |  |  |
| 70 CENPM | 0.941568 | | 4.08E-18 | -0.92349 | | 3.94E-16 |  |  |
| 71 KIF11 | 0.941084 | | 4.69E-18 | -0.93504 | | 2.47E-17 |  |  |
| 72 HMMR | 0.941083 | | 4.70E-18 | -0.93996 | | 6.48E-18 |  |  |
| 73 CBX2 | 0.940802 | | 5.09E-18 | -0.77267 | | 2.11E-08 |  |  |
| 74 HMGN1 | 0.94052 | | 5.52E-18 | -0.77987 | | 1.28E-08 |  |  |
| 75 SLC30A1 | 0.940378 | | 5.75E-18 | -0.88783 | | 2.38E-13 |  |  |
| 76 TAF4 | 0.940158 | | 6.12E-18 | -0.79547 | | 4.04E-09 |  |  |
| 77 ASPM | 0.939883 | | 6.62E-18 | -0.95986 | | 6.60E-21 |  |  |
| 78 CD248 | 0.939811 | | 6.75E-18 | -0.85413 | | 1.79E-11 |  |  |
| 79 ID3 | 0.939139 | | 8.16E-18 | -0.98093 | | 1.73E-26 |  |  |
| 80 IL11RA | 0.939101 | | 8.25E-18 | -0.85147 | | 2.40E-11 |  |  |
| 81 CRISPLD2 | 0.938207 | | 1.06E-17 | -0.89734 | | 5.47E-14 |  |  |
| 82 TAF1C | 0.938172 | | 1.07E-17 | -0.91928 | | 9.73E-16 |  |  |
| 83 TRRAP | 0.937337 | | 1.34E-17 | -0.91354 | | 3.09E-15 |  |  |
| 84 BUB1 | 0.935024 | | 2.48E-17 | -0.95969 | | 7.12E-21 |  |  |
| 85 WDR22 | 0.933964 | | 3.26E-17 | -0.86394 | | 5.74E-12 |  |  |
| 86 HMGB3 | 0.933943 | | 3.28E-17 | -0.93241 | | 4.85E-17 |  |  |
| 87 BTBD11 | 0.933764 | | 3.44E-17 | -0.92061 | | 7.36E-16 |  |  |
| 88 TTK | 0.932507 | | 4.73E-17 | -0.93116 | | 6.61E-17 |  |  |
| 89 MPHOSPH1 | 0.932321 | | 4.95E-17 | -0.94229 | | 3.30E-18 |  |  |
| 90 ULK1 | 0.93204 | | 5.31E-17 | -0.81234 | | 1.03E-09 |  |  |
| 91 ZNF508 | 0.931924 | | 5.47E-17 | -0.95367 | | 7.73E-20 |  |  |
| 92 AZI1 | 0.931907 | | 5.49E-17 | -0.95782 | | 1.55E-20 |  |  |
| 93 DDOST | 0.931379 | | 6.26E-17 | -0.91105 | | 4.98E-15 |  |  |
| 94 TROAP | 0.931035 | | 6.81E-17 | -0.95383 | | 7.29E-20 |  |  |
| 95 TYMS | 0.930926 | | 7.00E-17 | -0.95617 | | 2.99E-20 |  |  |
| 96 TMSL8 | 0.930423 | | 7.91E-17 | -0.93378 | | 3.42E-17 |  |  |
| 97 ZNF10 | 0.930317 | | 8.12E-17 | -0.82795 | | 2.58E-10 |  |  |
| 98 RKHD3 | 0.930308 | | 8.14E-17 | -0.95996 | | 6.32E-21 |  |  |
| 99 ABL1 | | 0.929824 | | 9.15E-17 | -0.75679 | | 5.98E-08 |  |
| 100 CDC45L | | 0.929629 | | 9.59E-17 | -0.92948 | | 9.94E-17 |  |
| 101 LDB2 | | 0.929558 | | 9.75E-17 | -0.94363 | | 2.21E-18 |  |
| 102 ANGPTL4 | | 0.929437 | | 1.00E-16 | -0.98898 | | 1.25E-30 |  |
| 103 EZH2 | | 0.928353 | | 1.30E-16 | -0.87881 | | 8.58E-13 |  |
| 104 SIPA1L2 | | 0.92826 | | 1.33E-16 | -0.88677 | | 2.79E-13 |  |
| 105 ZNF608 | | 0.928231 | | 1.34E-16 | -0.87455 | | 1.52E-12 |  |
| 106 POLQ | | 0.928006 | | 1.41E-16 | -0.91327 | | 3.26E-15 |  |
| 107 TFAM | | 0.927252 | | 1.68E-16 | -0.92896 | | 1.13E-16 |  |
| 108 DDX5 | | 0.927153 | | 1.72E-16 | -0.95308 | | 9.61E-20 |  |
| 109 SLC24A6 | | 0.926978 | | 1.79E-16 | -0.95022 | | 2.65E-19 |  |
| 110 CIRBP | | 0.926946 | | 1.81E-16 | -0.82463 | | 3.51E-10 |  |
| 111 PARD6G | | 0.926788 | | 1.87E-16 | -0.80205 | | 2.41E-09 |  |
| 112 HCFC1 | | 0.926634 | | 1.94E-16 | -0.94675 | | 8.36E-19 |  |
| 113 FAM64A | | 0.9266 | | 1.96E-16 | -0.94128 | | 4.44E-18 |  |
| 114 ZNF286 | | 0.925849 | | 2.32E-16 | -0.80307 | | 2.22E-09 |  |
| 115 RCL1 | | 0.925449 | | 2.55E-16 | -0.83807 | | 9.74E-11 |  |
| 116 SSBP2 | | 0.92506 | | 2.78E-16 | -0.8123 | | 1.04E-09 |  |
| 117 RALGDS | | 0.925042 | | 2.79E-16 | -0.94064 | | 5.34E-18 |  |
| 118 KLHL22 | | 0.924967 | | 2.84E-16 | -0.82284 | | 4.13E-10 |  |
| 119 REC8L1 | | 0.924815 | | 2.94E-16 | -0.95767 | | 1.64E-20 |  |
| 120 CTDSP2 | | 0.924603 | | 3.08E-16 | -0.74307 | | 1.39E-07 |  |
| 121 SUSD2 | | 0.923558 | | 3.89E-16 | -0.93976 | | 6.85E-18 |  |
| 122 LSM8 | | 0.923499 | | 3.94E-16 | -0.77297 | | 2.06E-08 |  |
| 123 HNRPUL1 | | 0.923351 | | 4.07E-16 | -0.89799 | | 4.92E-14 |  |
| 124 ZNF586 | | 0.922865 | | 4.52E-16 | -0.94199 | | 3.61E-18 |  |
| 125 AURKB | | 0.922488 | | 4.91E-16 | -0.93651 | | 1.67E-17 |  |
| 126 Septin 4 | | 0.922285 | | 5.14E-16 | -0.8398 | | 8.18E-11 |  |
| 127 OIP5 | | 0.921885 | | 5.60E-16 | -0.9559 | | 3.32E-20 |  |
| 128 PBK | | 0.921618 | | 5.93E-16 | -0.90956 | | 6.58E-15 |  |
| 129 ID2 | | 0.921399 | | 6.22E-16 | -0.94364 | | 2.20E-18 |  |
| 130 DUT | | 0.920926 | | 6.88E-16 | -0.82724 | | 2.76E-10 |  |
| 131 WDR51A | | 0.920504 | | 7.52E-16 | -0.94867 | | 4.47E-19 |  |
| 132 KIAA1434 | | 0.920118 | | 8.17E-16 | -0.81963 | | 5.50E-10 |  |
| 133 PFS2 | | 0.920083 | | 8.23E-16 | -0.93306 | | 4.11E-17 |  |
| 134 LRIG3 | | 0.919884 | | 8.58E-16 | -0.79054 | | 5.87E-09 |  |
| 135 TRIM33 | | 0.919652 | | 9.01E-16 | -0.81062 | | 1.20E-09 |  |
| 136 CBLB | | 0.919599 | | 9.11E-16 | -0.74188 | | 1.49E-07 |  |
| 137 HDAC1 | | 0.919537 | | 9.23E-16 | -0.94044 | | 5.65E-18 |  |
| 138 CCDC45 | | 0.919335 | | 9.62E-16 | -0.91284 | | 3.54E-15 |  |
| 139 GUSB | | 0.91929 | | 9.71E-16 | -0.93328 | | 3.88E-17 |  |
| 140 GMNN | | 0.918983 | | 1.04E-15 | -0.91389 | | 2.89E-15 |  |
| 141 PBX3 | | 0.918852 | | 1.06E-15 | -0.87323 | | 1.80E-12 |  |
| 142 SLIT2 | | 0.91863 | | 1.11E-15 | -0.73936 | | 1.72E-07 |  |
| 143 CPT1C | | 0.918117 | | 1.24E-15 | -0.90906 | | 7.21E-15 |  |
| 144 CDC2L6 | | 0.918076 | | 1.25E-15 | -0.78063 | | 1.21E-08 |  |
| 145 DNMT1 | | 0.917813 | | 1.32E-15 | -0.89976 | | 3.68E-14 |  |
| 146 ARID1A | | 0.917427 | | 1.43E-15 | -0.94762 | | 6.31E-19 |  |
| 147 NOTCH1 | | 0.91737 | | 1.44E-15 | -0.77908 | | 1.35E-08 |  |
| 148 C5 | | 0.917039 | | 1.54E-15 | -0.78868 | | 6.74E-09 |  |
| 149 HDHD1A | | 0.916977 | | 1.56E-15 | -0.81798 | | 6.36E-10 |  |
| 150 RBM10 | | 0.916817 | | 1.61E-15 | -0.91147 | | 4.60E-15 |  |
| 151 HIRIP3 | | 0.916779 | | 1.63E-15 | -0.83924 | | 8.66E-11 |  |
| 152 TOP3A | | 0.916483 | | 1.73E-15 | -0.88002 | | 7.26E-13 |  |
| 153 SLC12A9 | | 0.916221 | | 1.82E-15 | -0.92944 | | 1.00E-16 |  |
| 154 ZNF254 | | 0.915732 | | 2.01E-15 | -0.89203 | | 1.27E-13 |  |
| 155 TMEM98 | | 0.915617 | | 2.05E-15 | -0.82828 | | 2.50E-10 |  |
| 156 PPP1CC | | 0.915599 | | 2.06E-15 | -0.78009 | | 1.26E-08 |  |
| 157 FSTL5 | | 0.915133 | | 2.26E-15 | -0.84481 | | 4.89E-11 |  |
| 158 FLJ12505 | | 0.914661 | | 2.48E-15 | -0.83305 | | 1.59E-10 |  |
| 159 DLX1 | | 0.914543 | | 2.54E-15 | -0.86331 | | 6.20E-12 |  |
| 160 CENPA | | 0.914512 | | 2.56E-15 | -0.92728 | | 1.67E-16 |  |
| 161 FBXO21 | | 0.914398 | | 2.61E-15 | -0.80783 | | 1.51E-09 |  |
| 162 TMEM50B | | 0.914296 | | 2.67E-15 | -0.95029 | | 2.58E-19 |  |
| 163 CCNB2 | | 0.91426 | | 2.69E-15 | -0.96477 | | 7.01E-22 |  |
| 164 TOP2A | | 0.913656 | | 3.02E-15 | -0.91419 | | 2.72E-15 |  |
| 165 RAD51AP1 | | 0.911852 | | 4.28E-15 | -0.92721 | | 1.70E-16 |  |
| 166 CNAP1 | | 0.911261 | | 4.78E-15 | -0.90548 | | 1.38E-14 |  |
| 167 LOC139886 | | 0.911208 | | 4.83E-15 | -0.87122 | | 2.33E-12 |  |
| 168 SULF2 | | 0.91081 | | 5.21E-15 | -0.71995 | | 5.10E-07 |  |
| 169 SFRS10 | | 0.910344 | | 5.69E-15 | -0.81299 | | 9.78E-10 |  |
| 170 ZNF256 | | 0.910106 | | 5.94E-15 | -0.83587 | | 1.21E-10 |  |
| 171 HMGB1 | | 0.909966 | | 6.10E-15 | -0.89324 | | 1.05E-13 |  |
| 172 SUZ12 | | 0.909789 | | 6.31E-15 | -0.77585 | | 1.69E-08 |  |
| 173 CHSY1 | | 0.909729 | | 6.38E-15 | -0.80119 | | 2.58E-09 |  |
| 174 KIF20A | | 0.909058 | | 7.22E-15 | -0.9078 | | 9.09E-15 |  |
| 175 PRIM1 | | 0.908561 | | 7.91E-15 | -0.94842 | | 4.86E-19 |  |
| 176 SIAH1 | | 0.908295 | | 8.30E-15 | -0.89041 | | 1.62E-13 |  |
| 177 APOBEC3B | | 0.907982 | | 8.79E-15 | -0.96865 | | 9.37E-23 |  |
| 178 CDCA3 | | 0.90717 | | 1.02E-14 | -0.92816 | | 1.36E-16 |  |
| 179 SMARCAD1 | | 0.906725 | | 1.10E-14 | -0.83086 | | 1.96E-10 |  |
| 180 C13ORF23 | | 0.906723 | | 1.10E-14 | -0.87185 | | 2.15E-12 |  |
| 181 NETO2 | | 0.906688 | | 1.11E-14 | -0.77758 | | 1.50E-08 |  |
| 182 MAB21L2 | | 0.906605 | | 1.13E-14 | -0.76749 | | 2.99E-08 |  |
| 183 CCDC56 | | 0.905562 | | 1.36E-14 | -0.83988 | | 8.12E-11 |  |
| 184 RRM2 | | 0.905402 | | 1.40E-14 | -0.87945 | | 7.86E-13 |  |
| 185 PCNA | | 0.904411 | | 1.66E-14 | -0.7747 | | 1.83E-08 |  |
| 186 MXD4 | | 0.903237 | | 2.04E-14 | -0.68314 | | 3.18E-06 |  |
| 187 SMOX | | 0.903197 | | 2.05E-14 | -0.87762 | | 1.01E-12 |  |
| 188 COL12A1 | | 0.903129 | | 2.08E-14 | -0.80093 | | 2.63E-09 |  |
| 189 LOC55565 | | 0.902754 | | 2.22E-14 | -0.87974 | | 7.55E-13 |  |
| 190 TGFBR3 | | 0.902736 | | 2.22E-14 | -0.8201 | | 5.27E-10 |  |
| 191 CDC42EP4 | | 0.902712 | | 2.23E-14 | -0.79069 | | 5.80E-09 |  |
| 192 ZNF695 | | 0.901692 | | 2.66E-14 | -0.86503 | | 5.04E-12 |  |
| 193 KIAA0644 | | 0.901678 | | 2.66E-14 | -0.75024 | | 8.99E-08 |  |
| 194 MGC13170 | | 0.901458 | | 2.76E-14 | -0.81622 | | 7.41E-10 |  |
| 195 GLTSCR1 | | 0.901299 | | 2.84E-14 | -0.91451 | | 2.56E-15 |  |
| 196 JMJD1B | | 0.901096 | | 2.94E-14 | -0.8436 | | 5.55E-11 |  |
| 197 ARHGEF7 | | 0.901041 | | 2.97E-14 | -0.95272 | | 1.10E-19 |  |
| 198 COX10 | | 0.900627 | | 3.18E-14 | -0.82141 | | 4.69E-10 |  |
| 199 AMD1 | | 0.900588 | | 3.20E-14 | -0.76135 | | 4.47E-08 |  |
| 200 TRIM5 | | 0.900277 | | 3.37E-14 | -0.81764 | | 6.55E-10 |  |
| 201 ZCCHC14 | | 0.900228 | | 3.40E-14 | -0.87339 | | 1.76E-12 |  |
| 202 RPS9 | | 0.900168 | | 3.43E-14 | -0.76898 | | 2.70E-08 |  |
| 203 ZNF537 | | 0.899675 | | 3.73E-14 | -0.85121 | | 2.47E-11 |  |
| 204 NR1H3 | | 0.899306 | | 3.96E-14 | -0.71404 | | 6.97E-07 |  |
| 205 PIGC | | 0.899246 | | 4.00E-14 | -0.8359 | | 1.21E-10 |  |
| 206 SIRT1 | | 0.898896 | | 4.24E-14 | -0.90852 | | 7.97E-15 |  |
| 207 CDCA5 | | 0.898736 | | 4.36E-14 | -0.95627 | | 2.87E-20 |  |
| 208 QARS | | 0.898727 | | 4.36E-14 | -0.81828 | | 6.19E-10 |  |
| 209 RNF150 | | 0.898623 | | 4.44E-14 | -0.67791 | | 4.03E-06 |  |
| 210 GLTSCR2 | | 0.898586 | | 4.46E-14 | -0.77297 | | 2.06E-08 |  |
| 211 ZNF689 | | 0.898532 | | 4.50E-14 | -0.71171 | | 7.86E-07 |  |
| 212 LOC91461 | | 0.898398 | | 4.60E-14 | -0.96325 | | 1.45E-21 |  |
| 213 MMD | | 0.898373 | | 4.62E-14 | -0.78068 | | 1.21E-08 |  |
| 214 EVL | | 0.898322 | | 4.66E-14 | -0.8068 | | 1.64E-09 |  |
| 215 MEIS2 | | 0.89814 | | 4.80E-14 | -0.71585 | | 6.34E-07 |  |
| 216 USP6NL | | 0.898061 | | 4.87E-14 | -0.91172 | | 4.39E-15 |  |
| 217 DDEFL1 | | 0.897929 | | 4.97E-14 | -0.86669 | | 4.11E-12 |  |
| 218 COL3A1 | | 0.89743 | | 5.39E-14 | -0.8345 | | 1.38E-10 |  |
| 219 EXO1 | | 0.897429 | | 5.39E-14 | -0.85912 | | 1.01E-11 |  |
| 220 PCOLCE | | 0.897158 | | 5.64E-14 | -0.80992 | | 1.27E-09 |  |
| 221 PIAS4 | | 0.896568 | | 6.20E-14 | -0.81154 | | 1.11E-09 |  |
| 222 CAPN5 | | 0.896553 | | 6.21E-14 | -0.72742 | | 3.39E-07 |  |
| 223 EXOSC2 | | 0.895949 | | 6.85E-14 | -0.80399 | | 2.06E-09 |  |
| 224 SPHK2 | | 0.8959 | | 6.90E-14 | -0.86564 | | 4.67E-12 |  |
| 225 NUP62 | | 0.895869 | | 6.93E-14 | -0.93954 | | 7.28E-18 |  |
| 226 POLE3 | | 0.895723 | | 7.10E-14 | -0.86738 | | 3.78E-12 |  |
| 227 ZNF161 | | 0.895678 | | 7.15E-14 | -0.9519 | | 1.47E-19 |  |
| 228 LRRCC1 | | 0.895638 | | 7.20E-14 | -0.9158 | | 1.98E-15 |  |
| 229 SESTD1 | | 0.895614 | | 7.22E-14 | -0.93641 | | 1.72E-17 |  |
| 230 CEP55 | | 0.895336 | | 7.55E-14 | -0.95545 | | 3.95E-20 |  |
| 231 BCL2L12 | | 0.895333 | | 7.55E-14 | -0.88752 | | 2.50E-13 |  |
| 232 CDCA7 | | 0.895036 | | 7.92E-14 | -0.86285 | | 6.55E-12 |  |
| 233 UBE2C | | 0.894821 | | 8.19E-14 | -0.93546 | | 2.21E-17 |  |
| 234 KIFC1 | | 0.894736 | | 8.30E-14 | -0.92411 | | 3.44E-16 |  |
| 235 KIAA0182 | | 0.893977 | | 9.36E-14 | -0.90287 | | 2.17E-14 |  |
| 236 SF3B3 | | 0.893281 | | 1.04E-13 | -0.78824 | | 6.96E-09 |  |
| 237 BCL9 | | 0.892527 | | 1.17E-13 | -0.8835 | | 4.47E-13 |  |
| 238 PAXIP1 | | 0.892329 | | 1.21E-13 | -0.7584 | | 5.40E-08 |  |
| 239 PLEKHH3 | | 0.892314 | | 1.21E-13 | -0.94649 | | 9.10E-19 |  |
| 240 RUSC1 | | 0.892192 | | 1.23E-13 | -0.92832 | | 1.31E-16 |  |
| 241 EDG2 | | 0.892158 | | 1.24E-13 | -0.93794 | | 1.14E-17 |  |
| 242 HNRPM | | 0.89184 | | 1.30E-13 | -0.9467 | | 8.51E-19 |  |
| 243 SPC24 | | 0.891641 | | 1.34E-13 | -0.94191 | | 3.69E-18 |  |
| 244 SDC1 | | 0.890762 | | 1.54E-13 | -0.94065 | | 5.32E-18 |  |
| 245 C6ORF47 | | 0.890714 | | 1.55E-13 | -0.78044 | | 1.23E-08 |  |
| 246 MGC3121 | | 0.890573 | | 1.58E-13 | -0.90482 | | 1.55E-14 |  |
| 247 NOLC1 | | 0.890103 | | 1.70E-13 | -0.89821 | | 4.75E-14 |  |
| 248 FGFR3 | | 0.889906 | | 1.75E-13 | -0.78265 | | 1.05E-08 |  |
| 249 EVI1 | | 0.88925 | | 1.93E-13 | -0.66784 | | 6.31E-06 |  |
| 250 RPL18 | | 0.888623 | | 2.12E-13 | -0.92797 | | 1.42E-16 |  |
| 251 FLJ13909 | | 0.887602 | | 2.47E-13 | -0.90128 | | 2.85E-14 |  |
| 252 KHSRP | | 0.887311 | | 2.58E-13 | -0.84191 | | 6.60E-11 |  |
| 253 RCD-8 | | 0.886869 | | 2.75E-13 | -0.91313 | | 3.35E-15 |  |
| 254 BARD1 | | 0.886243 | | 3.01E-13 | -0.8874 | | 2.54E-13 |  |
| 255 GAS2L3 | | 0.886052 | | 3.10E-13 | -0.8882 | | 2.26E-13 |  |
| 256 VARSL | | 0.88596 | | 3.14E-13 | -0.87393 | | 1.64E-12 |  |
| 257 SMARCE1 | | 0.885754 | | 3.23E-13 | -0.76393 | | 3.78E-08 |  |
| 258 KIAA0376 | | 0.885654 | | 3.28E-13 | -0.7678 | | 2.93E-08 |  |
| 259 UBE2I | | 0.885396 | | 3.40E-13 | -0.91284 | | 3.54E-15 |  |
| 260 NXF1 | | 0.885315 | | 3.44E-13 | -0.97325 | | 6.04E-24 |  |
| 261 SUHW3 | | 0.884879 | | 3.67E-13 | -0.72824 | | 3.24E-07 |  |
| 262 PAQR8 | | 0.88486 | | 3.68E-13 | -0.88978 | | 1.78E-13 |  |
| 263 IRX2 | | 0.884808 | | 3.71E-13 | -0.814 | | 8.97E-10 |  |
| 264 AOF2 | | 0.884427 | | 3.91E-13 | -0.77997 | | 1.27E-08 |  |
| 265 DTL | | 0.8844 | | 3.93E-13 | -0.86073 | | 8.41E-12 |  |
| 266 KIAA0922 | | 0.884395 | | 3.93E-13 | -0.73322 | | 2.45E-07 |  |
| 267 MDK | | 0.883724 | | 4.33E-13 | -0.91745 | | 1.42E-15 |  |
| 268 PHC2 | | 0.883457 | | 4.49E-13 | -0.76608 | | 3.28E-08 |  |
| 269 C8ORF70 | | 0.883408 | | 4.52E-13 | -0.94725 | | 7.12E-19 |  |
| 270 BCLAF1 | | 0.883226 | | 4.64E-13 | -0.88673 | | 2.80E-13 |  |
| 271 HMGCS1 | | 0.883193 | | 4.66E-13 | -0.70536 | | 1.09E-06 |  |
| 272 KIAA0683 | | 0.882546 | | 5.11E-13 | -0.85853 | | 1.09E-11 |  |
| 273 LSM2 | | 0.882525 | | 5.13E-13 | -0.80765 | | 1.53E-09 |  |
| 274 SMARCD1 | | 0.882307 | | 5.29E-13 | -0.97782 | | 2.37E-25 |  |
| 275 C14ORF93 | | 0.8819 | | 5.60E-13 | -0.7512 | | 8.48E-08 |  |
| 276 BCL2 | | 0.881717 | | 5.74E-13 | -0.816 | | 7.55E-10 |  |
| 277 PKIA | | 0.881617 | | 5.82E-13 | -0.92555 | | 2.49E-16 |  |
| 278 LRFN3 | | 0.88128 | | 6.10E-13 | -0.78864 | | 6.76E-09 |  |
| 279 WASL | | 0.881277 | | 6.10E-13 | -0.76377 | | 3.82E-08 |  |
| 280 MLLT6 | | 0.881161 | | 6.20E-13 | -0.90806 | | 8.67E-15 |  |
| 281 RPS3 | | 0.881112 | | 6.25E-13 | -0.70627 | | 1.04E-06 |  |
| 282 FLJ25416 | | 0.880216 | | 7.07E-13 | -0.85511 | | 1.60E-11 |  |
| 283 PNRC2 | | 0.879966 | | 7.32E-13 | -0.69729 | | 1.62E-06 |  |
| 284 ZNF618 | | 0.879737 | | 7.55E-13 | -0.89875 | | 4.34E-14 |  |
| 285 KIF2C | | 0.879682 | | 7.61E-13 | -0.90705 | | 1.04E-14 |  |
| 286 C6ORF204 | | 0.878825 | | 8.56E-13 | -0.83539 | | 1.27E-10 |  |
| 287 LOC653170 | | 0.878176 | | 9.34E-13 | -0.75281 | | 7.67E-08 |  |
| 288 ZNF548 | | 0.878002 | | 9.57E-13 | -0.92065 | | 7.30E-16 |  |
| 289 ELF2 | | 0.877734 | | 9.92E-13 | -0.91886 | | 1.06E-15 |  |
| 290 RBM22 | | 0.877572 | | 1.01E-12 | -0.90956 | | 6.58E-15 |  |
| 291 GZMH | | 0.876615 | | 1.15E-12 | -0.80847 | | 1.43E-09 |  |
| 292 CCNB1 | | 0.87658 | | 1.16E-12 | -0.91 | | 6.06E-15 |  |
| 293 PSD3 | | 0.876152 | | 1.23E-12 | -0.72154 | | 4.68E-07 |  |
| 294 CDCA2 | | 0.875397 | | 1.36E-12 | -0.9059 | | 1.28E-14 |  |
| 295 FMNL2 | | 0.875322 | | 1.37E-12 | -0.94458 | | 1.66E-18 |  |
| 296 PDXP | | 0.875222 | | 1.39E-12 | -0.65745 | | 9.83E-06 |  |
| 297 ZNF263 | | 0.875019 | | 1.42E-12 | -0.89577 | | 7.04E-14 |  |
| 298 FNDC5 | | 0.874908 | | 1.45E-12 | -0.91691 | | 1.58E-15 |  |
| 299 KIAA0999 | | 0.874873 | | 1.45E-12 | -0.7581 | | 5.50E-08 |  |
| 300 LRRC14 | | 0.874779 | | 1.47E-12 | -0.68876 | | 2.44E-06 |  |
| 301 TIA1 | | 0.874735 | | 1.48E-12 | -0.95609 | | 3.09E-20 |  |
| 302 SCARB2 | | 0.874325 | | 1.56E-12 | -0.723 | | 4.32E-07 |  |
| 303 PHACTR4 | | 0.874006 | | 1.63E-12 | -0.97814 | | 1.84E-25 |  |
| 304 SFRS6 | | 0.873835 | | 1.66E-12 | -0.91663 | | 1.68E-15 |  |
| 305 BRD1 | | 0.873676 | | 1.70E-12 | -0.87566 | | 1.31E-12 |  |
| 306 TMEM64 | | 0.873662 | | 1.70E-12 | -0.64166 | | 1.87E-05 |  |
| 307 C16ORF30 | | 0.873564 | | 1.72E-12 | -0.92763 | | 1.54E-16 |  |
| 308 KIAA1618 | | 0.873294 | | 1.78E-12 | -0.91612 | | 1.86E-15 |  |
| 309 FOXM1 | | 0.873123 | | 1.82E-12 | -0.95583 | | 3.41E-20 |  |
| 310 AMT | | 0.872977 | | 1.86E-12 | -0.88539 | | 3.41E-13 |  |
| 311 SERPINF1 | | 0.872899 | | 1.88E-12 | -0.774 | | 1.92E-08 |  |
| 312 KIAA1683 | | 0.872777 | | 1.91E-12 | -0.74153 | | 1.52E-07 |  |
| 313 C9ORF140 | | 0.872692 | | 1.93E-12 | -0.91904 | | 1.02E-15 |  |
| 314 CHTF18 | | 0.871883 | | 2.14E-12 | -0.8987 | | 4.38E-14 |  |
| 315 KIAA1267 | | 0.871632 | | 2.21E-12 | -0.93357 | | 3.61E-17 |  |
| 316 C9ORF103 | | 0.871531 | | 2.24E-12 | -0.91906 | | 1.02E-15 |  |
| 317 EIF4B | | 0.8715 | | 2.25E-12 | -0.77465 | | 1.84E-08 |  |
| 318 RBM4 | | 0.870831 | | 2.45E-12 | -0.92921 | | 1.06E-16 |  |
| 319 CKS1B | | 0.870636 | | 2.51E-12 | -0.87275 | | 1.92E-12 |  |
| 320 MCM4 | | 0.870287 | | 2.62E-12 | -0.93534 | | 2.28E-17 |  |
| 321 KIF18A | | 0.87024 | | 2.64E-12 | -0.92482 | | 2.94E-16 |  |
| 322 HNRPR | | 0.8701 | | 2.69E-12 | -0.85072 | | 2.61E-11 |  |
| 323 NIPBL | | 0.870048 | | 2.71E-12 | -0.84863 | | 3.27E-11 |  |
| 324 SLC7A14 | | 0.869891 | | 2.76E-12 | -0.90321 | | 2.05E-14 |  |
| 325 DKFZP762E1312 0.869537 | | | | 2.89E-12 | -0.89962 | | 3.76E-14 |  |
| 326 Septin 9 0.869536 | | | | 2.89E-12 | -0.65431 | | 1.12E-05 |  |
| 327 TMEM70 0.868992 | | | | 3.09E-12 | -0.80963 | | 1.30E-09 |  |
| 328 LRP5L 0.868746 | | | | 3.19E-12 | -0.73153 | | 2.70E-07 |  |
| 329 MARCKSL1 0.868544 | | | | 3.27E-12 | -0.88968 | | 1.81E-13 |  |
| 330 ZNF289 0.868515 | | | | 3.28E-12 | -0.76113 | | 4.53E-08 |  |
| 331 ADCY3 0.868428 | | | | 3.32E-12 | -0.76653 | | 3.19E-08 |  |
| 332 KIF14 0.868064 | | | | 3.47E-12 | -0.86304 | | 6.40E-12 |  |
| 333 FANCD2 0.867991 | | | | 3.50E-12 | -0.92092 | | 6.89E-16 |  |
| 334 EFNB2 0.867093 | | | | 3.91E-12 | -0.8298 | | 2.17E-10 |  |
| 335 PHCA 0.866953 | | | | 3.98E-12 | -0.75788 | | 5.58E-08 |  |
| 336 RNF144 0.866866 | | | | 4.02E-12 | -0.75414 | | 7.06E-08 |  |
| 337 NAPRT1 0.866639 | | | | 4.14E-12 | -0.91962 | | 9.07E-16 |  |
| 338 MCM3 0.865992 | | | | 4.48E-12 | -0.83455 | | 1.38E-10 |  |
| 339 AGGF1 0.865792 | | | | 4.59E-12 | -0.70901 | | 9.04E-07 |  |
| 340 CDC25C 0.86498 | | | | 5.07E-12 | -0.83699 | | 1.08E-10 |  |
| 341 BTG2 0.864397 | | | | 5.44E-12 | -0.86646 | | 4.23E-12 |  |
| 342 SIM2 0.864087 | | | | 5.64E-12 | -0.80461 | | 1.96E-09 |  |
| 343 CRKL 0.864022 | | | | 5.69E-12 | -0.71455 | | 6.79E-07 |  |
| 344 C21ORF2 0.863811 | | | | 5.83E-12 | -0.82173 | | 4.56E-10 |  |
| 345 FRZB 0.863485 | | | | 6.07E-12 | -0.62393 | | 3.69E-05 |  |
| 346 HHEX 0.863276 | | | | 6.22E-12 | -0.69486 | | 1.83E-06 |  |
| 347 RASL12 0.863199 | | | | 6.28E-12 | -0.71004 | | 8.57E-07 |  |
| 348 FBLN1 0.86306 | | | | 6.38E-12 | -0.61439 | | 5.23E-05 |  |
| 349 RPL4 | | 0.862545 | | 6.79E-12 | -0.64482 | | 1.65E-05 |  |
| 350 TK1 | | 0.8624 | | 6.91E-12 | -0.94621 | | 9.95E-19 |  |
| 351 C1R | | 0.862015 | | 7.23E-12 | -0.81229 | | 1.04E-09 |  |
| 352 TEAD2 | | 0.861585 | | 7.61E-12 | -0.93264 | | 4.57E-17 |  |
| 353 SFRS5 | | 0.861541 | | 7.65E-12 | -0.94328 | | 2.46E-18 |  |
| 354 TNS3 | | 0.861536 | | 7.65E-12 | -0.89013 | | 1.69E-13 |  |
| 355 C20ORF20 | | 0.861316 | | 7.85E-12 | -0.90546 | | 1.38E-14 |  |
| 356 PRR11 | | 0.86115 | | 8.01E-12 | -0.95369 | | 7.70E-20 |  |
| 357 RBM23 | | 0.860249 | | 8.90E-12 | -0.88566 | | 3.28E-13 |  |
| 358 CDKN3 | | 0.860145 | | 9.01E-12 | -0.95285 | | 1.05E-19 |  |
| 359 C17ORF85 | | 0.859936 | | 9.23E-12 | -0.80391 | | 2.08E-09 |  |
| 360 SIN3A | | 0.859768 | | 9.41E-12 | -0.66193 | | 8.13E-06 |  |
| 361 ZNF317 | | 0.859662 | | 9.53E-12 | -0.74692 | | 1.10E-07 |  |
| 362 NCAPG2 | | 0.85945 | | 9.77E-12 | -0.90222 | | 2.43E-14 |  |
| 363 BMP2K | | 0.85924 | | 1.00E-11 | -0.71174 | | 7.86E-07 |  |
| 364 HNRPA1 | | 0.8591 | | 1.02E-11 | -0.75088 | | 8.64E-08 |  |
| 365 HADHSC | | 0.85873 | | 1.06E-11 | -0.70264 | | 1.25E-06 |  |
| 366 CREBBP | | 0.858588 | | 1.08E-11 | -0.95501 | | 4.68E-20 |  |
| 367 PTTG1IP | | 0.85789 | | 1.17E-11 | -0.72684 | | 3.50E-07 |  |
| 368 NLGN1 | | 0.857542 | | 1.22E-11 | -0.77451 | | 1.86E-08 |  |
| 369 TMTC4 | | 0.857306 | | 1.25E-11 | -0.83687 | | 1.10E-10 |  |
| 370 MYH10 | | 0.857036 | | 1.29E-11 | -0.84733 | | 3.75E-11 |  |
| 371 FLJ13912 | | 0.856895 | | 1.31E-11 | -0.80154 | | 2.51E-09 |  |
| 372 ADAR | | 0.856854 | | 1.32E-11 | -0.7667 | | 3.15E-08 |  |
| 373 TTC10 | | 0.856653 | | 1.35E-11 | -0.76857 | | 2.78E-08 |  |
| 374 C5ORF5 | | 0.856626 | | 1.35E-11 | -0.68997 | | 2.31E-06 |  |
| 375 CABLES2 | | 0.856303 | | 1.40E-11 | -0.90766 | | 9.33E-15 |  |
| 376 TJAP1 | | 0.856257 | | 1.41E-11 | -0.70387 | | 1.17E-06 |  |
| 377 C1ORF73 | | 0.856235 | | 1.41E-11 | -0.85735 | | 1.24E-11 |  |
| 378 ATPBD1B | | 0.856183 | | 1.42E-11 | -0.79186 | | 5.31E-09 |  |
| 379 ZNF518 | | 0.85607 | | 1.44E-11 | -0.83333 | | 1.55E-10 |  |
| 380 GLRX5 | | 0.856046 | | 1.44E-11 | -0.73259 | | 2.54E-07 |  |
| 381 CNOT1 | | 0.855898 | | 1.47E-11 | -0.67978 | | 3.71E-06 |  |
| 382 TIMELESS | | 0.855797 | | 1.48E-11 | -0.94536 | | 1.30E-18 |  |
| 383 C20ORF11 | | 0.855519 | | 1.53E-11 | -0.70456 | | 1.13E-06 |  |
| 384 RFC3 | | 0.855179 | | 1.59E-11 | -0.80241 | | 2.34E-09 |  |
| 385 TPR | | 0.855159 | | 1.60E-11 | -0.92061 | | 7.36E-16 |  |
| 386 DDX18 | | 0.854904 | | 1.64E-11 | -0.86413 | | 5.62E-12 |  |
| 387 NDE1 | | 0.85445 | | 1.73E-11 | -0.67228 | | 5.19E-06 |  |
| 388 MXD3 | | 0.854355 | | 1.75E-11 | -0.88654 | | 2.88E-13 |  |
| 389 RGL1 | | 0.853772 | | 1.86E-11 | -0.69405 | | 1.90E-06 |  |
| 390 CABC1 | | 0.852947 | | 2.04E-11 | -0.81092 | | 1.17E-09 |  |
| 391 IHPK2 | | 0.852781 | | 2.08E-11 | -0.85059 | | 2.64E-11 |  |
| 392 ATG4B | | 0.852333 | | 2.18E-11 | -0.78498 | | 8.85E-09 |  |
| 393 CDCA8 | | 0.852291 | | 2.19E-11 | -0.91607 | | 1.88E-15 |  |
| 394 C14ORF32 | | 0.852221 | | 2.21E-11 | -0.68042 | | 3.60E-06 |  |
| 395 MCM10 | | 0.85211 | | 2.24E-11 | -0.83063 | | 2.01E-10 |  |
| 396 COQ2 | | 0.851317 | | 2.44E-11 | -0.8914 | | 1.39E-13 |  |
| 397 ARHGAP17 | | 0.851293 | | 2.45E-11 | -0.85213 | | 2.24E-11 |  |
| 398 FZD2 | | 0.850998 | | 2.53E-11 | -0.75067 | | 8.75E-08 |  |
| 399 PUNC | | 0.85059 | | 2.64E-11 | -0.65287 | | 1.19E-05 |  |
| 400 ARS2 | | 0.850414 | | 2.70E-11 | -0.91871 | | 1.10E-15 |  |
| 401 BRPF1 | | 0.850176 | | 2.77E-11 | -0.96582 | | 4.16E-22 |  |
| 402 MIDN | | 0.849954 | | 2.83E-11 | -0.96852 | | 1.00E-22 |  |
| 403 RTKN | | 0.849612 | | 2.94E-11 | -0.66305 | | 7.76E-06 |  |
| 404 FKBP7 | | 0.849545 | | 2.96E-11 | -0.65768 | | 9.73E-06 |  |
| 405 SIVA | | 0.84926 | | 3.05E-11 | -0.71988 | | 5.12E-07 |  |
| 406 CDH18 | | 0.849248 | | 3.06E-11 | -0.9135 | | 3.12E-15 |  |
| 407 PP2447 | | 0.849192 | | 3.08E-11 | -0.78881 | | 6.68E-09 |  |
| 408 PIM1 | | 0.848486 | | 3.32E-11 | -0.95366 | | 7.78E-20 |  |
| 409 TFDP1 | | 0.848429 | | 3.34E-11 | -0.77948 | | 1.31E-08 |  |
| 410 SCARA3 | | 0.848424 | | 3.34E-11 | -0.93083 | | 7.16E-17 |  |
| 411 GM632 | | 0.848086 | | 3.46E-11 | -0.74553 | | 1.20E-07 |  |
| 412 C12ORF32 | | 0.847967 | | 3.51E-11 | -0.83401 | | 1.45E-10 |  |
| 413 LOC201725 | | 0.847945 | | 3.52E-11 | -0.84098 | | 7.26E-11 |  |
| 414 LOC147808 | | 0.847881 | | 3.54E-11 | -0.80942 | | 1.32E-09 |  |
| 415 ZNF22 | | 0.847844 | | 3.55E-11 | -0.85327 | | 1.97E-11 |  |
| 416 DLX2 | | 0.847674 | | 3.62E-11 | -0.83063 | | 2.01E-10 |  |
| 417 HMGN2 | | 0.847618 | | 3.64E-11 | -0.68546 | | 2.85E-06 |  |
| 418 BRCA1 | | 0.847417 | | 3.72E-11 | -0.85187 | | 2.30E-11 |  |
| 419 MATR3 | | 0.846986 | | 3.89E-11 | -0.66763 | | 6.37E-06 |  |
| 420 RUNX3 | | 0.846927 | | 3.92E-11 | -0.96862 | | 9.51E-23 |  |
| 421 TFAP2A | | 0.846714 | | 4.01E-11 | -0.80401 | | 2.06E-09 |  |
| 422 PCBP1 | | 0.846104 | | 4.27E-11 | -0.81319 | | 9.61E-10 |  |
| 423 EGR3 | | 0.845903 | | 4.37E-11 | -0.93124 | | 6.48E-17 |  |
| 424 OLFML3 | | 0.845861 | | 4.38E-11 | -0.74952 | | 9.40E-08 |  |
| 425 AXIIR | | 0.845851 | | 4.39E-11 | -0.91334 | | 3.21E-15 |  |
| 426 PCDH9 | | 0.845827 | | 4.40E-11 | -0.89039 | | 1.63E-13 |  |
| 427 RPA1 | | 0.845733 | | 4.44E-11 | -0.68076 | | 3.54E-06 |  |
| 428 SNAPC4 | | 0.845573 | | 4.52E-11 | -0.90774 | | 9.18E-15 |  |
| 429 TP53BP2 | | 0.845525 | | 4.54E-11 | -0.93594 | | 1.95E-17 |  |
| 430 HES1 | | 0.8449 | | 4.85E-11 | -0.95884 | | 1.02E-20 |  |
| 431 ANKFY1 | | 0.844775 | | 4.91E-11 | -0.62505 | | 3.54E-05 |  |
| 432 MAFB | | 0.843546 | | 5.58E-11 | -0.84169 | | 6.75E-11 |  |
| 433 FOXO3A | | 0.842967 | | 5.93E-11 | -0.61157 | | 5.79E-05 |  |
| 434 KLF9 | | 0.842865 | | 5.99E-11 | -0.95978 | | 6.84E-21 |  |
| 435 ACAD10 | | 0.842768 | | 6.05E-11 | -0.68369 | | 3.10E-06 |  |
| 436 SLC35F1 | | 0.842679 | | 6.10E-11 | -0.68147 | | 3.43E-06 |  |
| 437 GEMIN4 | | 0.842669 | | 6.11E-11 | -0.66725 | | 6.47E-06 |  |
| 438 OGG1 | | 0.842577 | | 6.17E-11 | -0.66437 | | 7.33E-06 |  |
| 439 CTSC | | 0.842513 | | 6.21E-11 | -0.94737 | | 6.85E-19 |  |
| 440 P2RY5 | | 0.842107 | | 6.47E-11 | -0.71493 | | 6.65E-07 |  |
| 441 MED4 | | 0.842051 | | 6.51E-11 | -0.73268 | | 2.53E-07 |  |
| 442 C17ORF70 | | 0.841668 | | 6.77E-11 | -0.80371 | | 2.11E-09 |  |
| 443 PRC1 | | 0.841574 | | 6.84E-11 | -0.93812 | | 1.08E-17 |  |
| 444 SQLE | | 0.841555 | | 6.85E-11 | -0.74001 | | 1.66E-07 |  |
| 445 EGR1 | | 0.841434 | | 6.93E-11 | -0.94985 | | 3.00E-19 |  |
| 446 PBX1 | | 0.841201 | | 7.10E-11 | -0.63634 | | 2.30E-05 |  |
| 447 MGC39900 | | 0.84119 | | 7.11E-11 | -0.94512 | | 1.40E-18 |  |
| 448 NIN | | 0.841036 | | 7.22E-11 | -0.76646 | | 3.20E-08 |  |
| 449 SET | | 0.840542 | | 7.59E-11 | -0.78841 | | 6.88E-09 |  |
| 450 TCF20 | | 0.840352 | | 7.74E-11 | -0.86294 | | 6.47E-12 |  |
| 451 SPRY2 | | 0.840289 | | 7.79E-11 | -0.86402 | | 5.69E-12 |  |
| 452 RFC2 | | 0.840183 | | 7.87E-11 | -0.77327 | | 2.02E-08 |  |
| 453 ACVR1 | | 0.840173 | | 7.88E-11 | -0.69752 | | 1.60E-06 |  |
| 454 SEC11L1 | | 0.84007 | | 7.96E-11 | -0.808 | | 1.49E-09 |  |
| 455 MED6 | | 0.840025 | | 8.00E-11 | -0.83157 | | 1.83E-10 |  |
| 456 EEF2 | | 0.840014 | | 8.01E-11 | -0.61726 | | 4.71E-05 |  |
| 457 CHD1 | | 0.83992 | | 8.08E-11 | -0.72367 | | 4.17E-07 |  |
| 458 COL21A1 | | 0.839665 | | 8.30E-11 | -0.83872 | | 9.12E-11 |  |
| 459 ECH1 | | 0.839415 | | 8.51E-11 | -0.93064 | | 7.51E-17 |  |
| 460 ZNF521 | | 0.839336 | | 8.57E-11 | -0.76529 | | 3.46E-08 |  |
| 461 PRRG1 | | 0.839283 | | 8.62E-11 | -0.63434 | | 2.49E-05 |  |
| 462 RBM15B | | 0.838648 | | 9.19E-11 | -0.69682 | | 1.66E-06 |  |
| 463 DLG7 | | 0.838361 | | 9.45E-11 | -0.892 | | 1.27E-13 |  |
| 464 ABCC5 | | 0.837948 | | 9.85E-11 | -0.68486 | | 2.93E-06 |  |
| 465 LOC158160 | | 0.837571 | | 1.02E-10 | -0.76003 | | 4.87E-08 |  |
| 466 KIAA0528 | | 0.837016 | | 1.08E-10 | -0.6686 | | 6.10E-06 |  |
| 467 MGC15476 | | 0.836941 | | 1.09E-10 | -0.64405 | | 1.70E-05 |  |
| 468 SLC26A11 | | 0.836933 | | 1.09E-10 | -0.78869 | | 6.74E-09 |  |
| 469 ANLN | | 0.836034 | | 1.19E-10 | -0.88862 | | 2.12E-13 |  |
| 470 IFT88 | | 0.836017 | | 1.19E-10 | -0.67059 | | 5.59E-06 |  |
| 471 LBR | | 0.835919 | | 1.20E-10 | -0.74353 | | 1.35E-07 |  |
| 472 EEF1D | | 0.835681 | | 1.23E-10 | -0.74217 | | 1.46E-07 |  |
| 473 MARCKS | | 0.835656 | | 1.24E-10 | -0.71287 | | 7.40E-07 |  |
| 474 ATF4 | | 0.835311 | | 1.28E-10 | -0.77668 | | 1.60E-08 |  |
| 475 NULP1 | | 0.835177 | | 1.29E-10 | -0.6022 | | 8.04E-05 |  |
| 476 C6ORF173 | | 0.834668 | | 1.36E-10 | -0.91443 | | 2.60E-15 |  |
| 477 DDX46 | | 0.834589 | | 1.37E-10 | -0.74761 | | 1.05E-07 |  |
| 478 PRKRIR | | 0.83456 | | 1.38E-10 | -0.62668 | | 3.33E-05 |  |
| 479 PARP1 | | 0.834214 | | 1.42E-10 | -0.82964 | | 2.20E-10 |  |
| 480 MRPS31 | | 0.834135 | | 1.43E-10 | -0.74214 | | 1.46E-07 |  |
| 481 NFATC4 | | 0.833372 | | 1.54E-10 | -0.90045 | | 3.28E-14 |  |
| 482 PTEN | | 0.833117 | | 1.58E-10 | -0.67049 | | 5.62E-06 |  |
| 483 COQ7 | | 0.83285 | | 1.62E-10 | -0.78666 | | 7.83E-09 |  |
| 484 ZNF462 | | 0.832669 | | 1.65E-10 | -0.94525 | | 1.34E-18 |  |
| 485 SSR2 | | 0.832362 | | 1.70E-10 | -0.74141 | | 1.53E-07 |  |
| 486 CUTL1 | | 0.832029 | | 1.76E-10 | -0.82212 | | 4.40E-10 |  |
| 487 GRAMD1A | | 0.831983 | | 1.76E-10 | -0.74769 | | 1.05E-07 |  |
| 488 PSRC1 | | 0.831855 | | 1.79E-10 | -0.8603 | | 8.84E-12 |  |
| 489 FAM46C | | 0.831762 | | 1.80E-10 | -0.87949 | | 7.81E-13 |  |
| 490 LAMP2 | | 0.831597 | | 1.83E-10 | -0.69373 | | 1.93E-06 |  |
| 491 GCN5L2 | | 0.831476 | | 1.85E-10 | -0.71379 | | 7.06E-07 |  |
| 492 REV3L | | 0.831311 | | 1.88E-10 | -0.83182 | | 1.79E-10 |  |
| 493 TH1L | | 0.830969 | | 1.94E-10 | -0.80352 | | 2.14E-09 |  |
| 494 SCML1 | | 0.830493 | | 2.03E-10 | -0.94195 | | 3.65E-18 |  |
| 495 RNASEH2A | | 0.83043 | | 2.04E-10 | -0.91798 | | 1.27E-15 |  |
| 496 C18ORF24 | | 0.830301 | | 2.07E-10 | -0.91227 | | 3.95E-15 |  |
| 497 GTPBP6 | | 0.829908 | | 2.15E-10 | -0.92527 | | 2.65E-16 |  |
| 498 FEN1 | | 0.828958 | | 2.35E-10 | -0.8483 | | 3.39E-11 |  |
| 499 RHOBTB3 | | 0.828932 | | 2.36E-10 | -0.68801 | 2.53E-06 | |  |
| 500 NFYA | | 0.828765 | | 2.39E-10 | -0.71395 | 7.00E-07 | |  |
| 501 PPM1G | | 0.828602 | | 2.43E-10 | -0.77792 | 1.47E-08 | |  |
| 502 ZNF350 | | 0.828542 | | 2.44E-10 | -0.8186 | 6.02E-10 | |  |
| 503 GNG2 | | 0.828439 | | 2.47E-10 | -0.84791 | 3.53E-11 | |  |
| 504 ARHGEF19 | | 0.828437 | | 2.47E-10 | -0.82645 | 2.97E-10 | |  |
| 505 TACC1 | | 0.828355 | | 2.49E-10 | -0.85606 | 1.44E-11 | |  |
| 506 SBK1 | | 0.828139 | | 2.54E-10 | -0.70592 | 1.06E-06 | |  |
| 507 BCKDHB | | 0.827645 | | 2.66E-10 | -0.64508 | 1.63E-05 | |  |
| 508 PPWD1 | | 0.827418 | | 2.71E-10 | -0.63367 | 2.55E-05 | |  |
| 509 KCNK10 | | 0.826883 | | 2.85E-10 | -0.78242 | 1.06E-08 | |  |
| 510 KIF4A | | 0.825555 | | 3.22E-10 | -0.88477 | 3.72E-13 | |  |
| 511 THAP11 | | 0.82533 | | 3.29E-10 | -0.63814 | 2.15E-05 | |  |
| 512 PTBP1 | | 0.825098 | | 3.36E-10 | -0.89503 | 7.92E-14 | |  |
| 513 FAM53C | | 0.82503 | | 3.38E-10 | -0.62793 | 3.18E-05 | |  |
| 514 SGOL1 | | 0.824854 | | 3.44E-10 | -0.90474 | 1.57E-14 | |  |
| 515 CNOT10 | | 0.82466 | | 3.50E-10 | -0.6157 | 4.99E-05 | |  |
| 516 FKSG14 | | 0.824305 | | 3.61E-10 | -0.86791 | 3.54E-12 | |  |
| 517 TBC1D2B | | 0.823937 | | 3.74E-10 | -0.63441 | 2.48E-05 | |  |
| 518 GLI2 | | 0.823688 | | 3.82E-10 | -0.90209 | 2.48E-14 | |  |
| 519 AXIN2 | | 0.823162 | | 4.01E-10 | -0.85742 | 1.23E-11 | |  |
| 520 C9ORF102 | | 0.822925 | | 4.09E-10 | -0.64905 | 1.39E-05 | |  |
| 521 CTRC | | 0.822871 | | 4.11E-10 | -0.85902 | 1.03E-11 | |  |
| 522 SOX11 | | 0.822352 | | 4.31E-10 | -0.85325 | 1.97E-11 | |  |
| 523 CPXM | | 0.822324 | | 4.32E-10 | -0.77981 | 1.28E-08 | |  |
| 524 FEM1A | | 0.821904 | | 4.49E-10 | -0.75871 | 5.29E-08 | |  |
| 525 GPR154 | | 0.821344 | | 4.72E-10 | -0.89264 | 1.15E-13 | |  |
| 526 ZIK1 | | 0.821159 | | 4.80E-10 | -0.76939 | 2.63E-08 | |  |
| 527 TGIF1 | | 0.820878 | | 4.92E-10 | -0.94335 | 2.40E-18 | |  |
| 528 RFWD3 | | 0.820704 | | 5.00E-10 | -0.66757 | 6.38E-06 | |  |
| 529 PRKCA | | 0.820461 | | 5.11E-10 | -0.75532 | 6.56E-08 | |  |
| 530 EDN3 | | 0.820345 | | 5.16E-10 | -0.81316 | 9.64E-10 | |  |
| 531 MGC4562 | | 0.820151 | | 5.25E-10 | -0.63475 | 2.45E-05 | |  |
| 532 ITPR2 | | 0.819979 | | 5.33E-10 | -0.82768 | 2.65E-10 | |  |
| 533 L3MBTL3 | | 0.819731 | | 5.45E-10 | -0.70871 | 9.18E-07 | |  |
| 534 SMC6L1 | | 0.819278 | | 5.67E-10 | -0.65951 | 9.01E-06 | |  |
| 535 SEPHS1 | | 0.81871 | | 5.96E-10 | -0.56513 | 0.000268 | |  |
| 536 SUOX | | 0.818408 | | 6.12E-10 | -0.56946 | 0.000235 | |  |
| 537 C6ORF84 | | 0.818215 | | 6.23E-10 | -0.81992 | 5.36E-10 | |  |
| 538 IRF2BP2 | | 0.81813 | | 6.27E-10 | -0.57478 | 0.000199 | |  |
| 539 C14ORF120 | | 0.818 | | 6.35E-10 | -0.72736 | 3.41E-07 | |  |
| 540 ZNF559 | | 0.817847 | | 6.43E-10 | -0.84771 | 3.61E-11 | |  |
| 541 FDFT1 | | 0.817493 | | 6.63E-10 | -0.68682 | 2.68E-06 | |  |
| 542 RBM14 | | 0.817387 | | 6.70E-10 | -0.56415 | 0.000276 | |  |
| 543 CEP1 | | 0.817169 | | 6.83E-10 | -0.84337 | 5.69E-11 | |  |
| 544 LDLR | | 0.816802 | | 7.05E-10 | -0.58514 | 0.000142 | |  |
| 545 TPX2 | | 0.816759 | | 7.07E-10 | -0.86704 | 3.94E-12 | |  |
| 546 IXL | | 0.816328 | | 7.34E-10 | -0.67204 | 5.25E-06 | |  |
| 547 SECISBP2 | | 0.816273 | | 7.38E-10 | -0.7753 | 1.76E-08 | |  |
| 548 GTSE1 | | 0.816222 | | 7.41E-10 | -0.84997 | 2.83E-11 | |  |
| 549 WEE1 | | 0.816173 | | 7.44E-10 | -0.58617 | 0.000138 | |  |
| 550 RFC5 | | 0.816111 | | 7.48E-10 | -0.70651 | 1.03E-06 | |  |
| 551 C1ORF135 | | 0.815989 | | 7.56E-10 | -0.80398 | 2.06E-09 | |  |
| 552 PLK4 | | 0.815794 | | 7.69E-10 | -0.88866 | 2.11E-13 | |  |
| 553 SLC25A10 | | 0.81554 | | 7.86E-10 | -0.75702 | 5.89E-08 | |  |
| 554 MFAP2 | | 0.81536 | | 7.98E-10 | -0.67254 | 5.13E-06 | |  |
| 555 MIS12 | | 0.815155 | | 8.13E-10 | -0.63453 | 2.47E-05 | |  |
| 556 RIF1 | | 0.815149 | | 8.13E-10 | -0.71787 | 5.70E-07 | |  |
| 557 IFI44L | | 0.814491 | | 8.61E-10 | -0.78813 | 7.02E-09 | |  |
| 558 ING1 | | 0.814314 | | 8.74E-10 | -0.96102 | 4.00E-21 | |  |
| 559 USP42 | | 0.814229 | | 8.80E-10 | -0.62282 | 3.85E-05 | |  |
| 560 FLJ31413 | | 0.81411 | | 8.89E-10 | -0.73551 | 2.15E-07 | |  |
| 561 FNBP1L | | 0.814069 | | 8.92E-10 | -0.56085 | 0.000305 | |  |
| 562 RGS10 | | 0.813928 | | 9.03E-10 | -0.66951 | 5.86E-06 | |  |
| 563 PHF13 | | 0.813288 | | 9.54E-10 | -0.91221 | 4.00E-15 | |  |
| 564 SFRS7 | | 0.813005 | | 9.77E-10 | -0.61443 | 5.22E-05 | |  |
| 565 ZNF519 | | 0.812488 | | 1.02E-09 | -0.8054 | 1.84E-09 | |  |
| 566 GLE1L | | 0.812437 | | 1.03E-09 | -0.6254 | 3.49E-05 | |  |
| 567 ZNF480 | | 0.812368 | | 1.03E-09 | -0.77442 | 1.87E-08 | |  |
| 568 APRIN | | 0.812283 | | 1.04E-09 | -0.58723 | 0.000133 | |  |
| 569 HMG20B | | 0.811428 | | 1.12E-09 | -0.80699 | 1.62E-09 | |  |
| 570 PHIP | | 0.811092 | | 1.15E-09 | -0.61603 | 4.93E-05 | |  |
| 571 PLA2G4B | | 0.811077 | | 1.15E-09 | -0.90381 | 1.85E-14 | |  |
| 572 C1ORF112 | | 0.81086 | | 1.17E-09 | -0.6669 | 6.57E-06 | |  |
| 573 HSF2 | | 0.810837 | | 1.17E-09 | -0.72198 | 4.57E-07 | |  |
| 574 SF3A3 | | 0.81057 | | 1.20E-09 | -0.63788 | 2.17E-05 | |  |
| 575 FUS | | 0.810326 | | 1.22E-09 | -0.8183 | 6.18E-10 | |  |
| 576 KNTC2 | | 0.810133 | | 1.24E-09 | -0.85045 | 2.68E-11 | |  |
| 577 C7ORF27 | | 0.809953 | | 1.26E-09 | -0.7068 | 1.01E-06 | |  |
| 578 STAT2 | | 0.80995 | | 1.26E-09 | -0.82332 | 3.95E-10 | |  |
| 579 RSBN1L | | 0.809656 | | 1.30E-09 | -0.76523 | 3.47E-08 | |  |
| 580 DPYD | | 0.809209 | | 1.34E-09 | -0.84786 | 3.55E-11 | |  |
| 581 ADAM17 | | 0.808485 | | 1.43E-09 | -0.8539 | 1.84E-11 | |  |
| 582 FAM105B | | 0.808477 | | 1.43E-09 | -0.70464 | 1.13E-06 | |  |
| 583 CDKN2C | | 0.808316 | | 1.45E-09 | -0.6525 | 1.21E-05 | |  |
| 584 EFNA4 | | 0.808088 | | 1.48E-09 | -0.61204 | 5.69E-05 | |  |
| 585 EIF4EBP2 | | 0.808059 | | 1.48E-09 | -0.62936 | 3.01E-05 | |  |
| 586 ZNF721 | | 0.808035 | | 1.48E-09 | -0.65236 | 1.21E-05 | |  |
| 587 NUCB1 | | 0.806717 | | 1.65E-09 | -0.74662 | 1.12E-07 | |  |
| 588 JARID1B | | 0.806699 | | 1.65E-09 | -0.91622 | 1.82E-15 | |  |
| 589 UBQLN4 | | 0.80668 | | 1.66E-09 | -0.59945 | 8.83E-05 | |  |
| 590 NUP54 | | 0.806483 | | 1.68E-09 | -0.7229 | 4.35E-07 | |  |
| 591 KIAA0446 | | 0.805837 | | 1.78E-09 | -0.61321 | 5.46E-05 | |  |
| 592 C19ORF37 | | 0.805268 | | 1.86E-09 | -0.81013 | 1.25E-09 | |  |
| 593 FLJ10159 | | 0.805136 | | 1.88E-09 | -0.62682 | 3.31E-05 | |  |
| 594 SAFB | | 0.804606 | | 1.96E-09 | -0.53187 | 0.000704 | |  |
| 595 C20ORF100 | | 0.804246 | | 2.02E-09 | -0.92516 | 2.72E-16 | |  |
| 596 SNIP1 | | 0.803661 | | 2.12E-09 | -0.76985 | 2.55E-08 | |  |
| 597 UBN1 | | 0.803398 | | 2.16E-09 | -0.90206 | 2.50E-14 | |  |
| 598 RPUSD4 | | 0.803363 | | 2.17E-09 | -0.75533 | 6.56E-08 | |  |
| 599 ECHDC3 | | 0.802882 | | 2.25E-09 | -0.63416 | 2.51E-05 | |  |
| 600 RNPS1 | | 0.802297 | | 2.36E-09 | -0.8511 | 2.50E-11 | |  |
| 601 CAMSAP1 | | 0.801855 | | 2.45E-09 | -0.84548 | 4.56E-11 | |  |
| 602 WDR73 | | 0.801636 | | 2.49E-09 | -0.63353 | 2.57E-05 | |  |
| 603 PTTG1 | | 0.801252 | | 2.57E-09 | -0.93261 | 4.60E-17 | |  |
| 604 LOC340156 | | 0.801187 | | 2.58E-09 | -0.64035 | 1.97E-05 | |  |
| 605 KUA | | 0.800885 | | 2.64E-09 | -0.80849 | 1.43E-09 | |  |
| 606 HSPA14 | | 0.800883 | | 2.64E-09 | -0.74611 | 1.15E-07 | |  |
| 607 ZNF326 | | 0.800565 | | 2.71E-09 | -0.65886 | 9.26E-06 | |  |
| 608 MGC11335 | | 0.800227 | | 2.79E-09 | -0.54642 | 0.000467 | |  |
| 609 ZCCHC3 | | 0.800048 | | 2.82E-09 | -0.6218 | 3.99E-05 | |  |
| 610 DPYSL2 | | 0.799371 | | 2.98E-09 | -0.58409 | 0.000147 | |  |
| 611 BLMH | | 0.799283 | | 3.00E-09 | -0.53956 | 0.000568 | |  |
| 612 AARS | | 0.799072 | | 3.05E-09 | -0.83297 | 1.60E-10 | |  |
| 613 GLI3 | | 0.79888 | | 3.10E-09 | -0.9031 | 2.09E-14 | |  |
| 614 DEPDC1B | | 0.798617 | | 3.16E-09 | -0.63187 | 2.73E-05 | |  |
| 615 WDR79 | | 0.798523 | | 3.18E-09 | -0.57413 | 0.000203 | |  |
| 616 FLJ20674 | | 0.798507 | | 3.19E-09 | -0.82618 | 3.04E-10 | |  |
| 617 RBM5 | | 0.798389 | | 3.22E-09 | -0.85401 | 1.81E-11 | |  |
| 618 PSIP1 | | 0.797749 | | 3.38E-09 | -0.58426 | 0.000147 | |  |
| 619 C2ORF26 | | 0.797468 | | 3.46E-09 | -0.78992 | 6.15E-09 | |  |
| 620 CENPE | | 0.796618 | | 3.69E-09 | -0.78929 | 6.44E-09 | |  |
| 621 AFG3L2 | | 0.796595 | | 3.70E-09 | -0.7135 | 7.17E-07 | |  |
| 622 NAT10 | | 0.796152 | | 3.83E-09 | -0.62619 | 3.39E-05 | |  |
| 623 LOC728643 | | 0.795765 | | 3.95E-09 | -0.72092 | 4.84E-07 | |  |
| 624 ZNF278 | | 0.795447 | | 4.04E-09 | -0.81976 | 5.43E-10 | |  |
| 625 RNF38 | | 0.795416 | | 4.05E-09 | -0.70984 | 8.66E-07 | |  |
| 626 ANKRD16 | | 0.794677 | | 4.29E-09 | -0.7149 | 6.66E-07 | |  |
| 627 LRBA | | 0.794673 | | 4.29E-09 | -0.87087 | 2.44E-12 | |  |
| 628 RBJ | | 0.794324 | | 4.41E-09 | -0.58303 | 0.000153 | |  |
| 629 CPSF1 | | 0.794116 | | 4.48E-09 | -0.74152 | 1.52E-07 | |  |
| 630 FLJ32745 | | 0.793969 | | 4.53E-09 | -0.61192 | 5.71E-05 | |  |
| 631 SETMAR | | 0.793741 | | 4.61E-09 | -0.83138 | 1.87E-10 | |  |
| 632 MADD | | 0.793361 | | 4.74E-09 | -0.74998 | 9.13E-08 | |  |
| 633 PSMC3IP | | 0.79326 | | 4.78E-09 | -0.65892 | 9.24E-06 | |  |
| 634 H3F3B | | 0.793237 | | 4.79E-09 | -0.94814 | 5.32E-19 | |  |
| 635 SCRIB | | 0.792698 | | 4.99E-09 | -0.74382 | 1.32E-07 | |  |
| 636 DKFZP586H2123 0.791852 | | | | 5.32E-09 | -0.70625 | 1.04E-06 | |  |
| 637 EIF3S6 0.791753 | | | | 5.36E-09 | -0.53745 | 0.000603 | |  |
| 638 PGBD2 0.79142 | | | | 5.49E-09 | -0.57703 | 0.000185 | |  |
| 639 EMD 0.791277 | | | | 5.55E-09 | -0.71176 | 7.85E-07 | |  |
| 640 HNRPA0 0.790839 | | | | 5.74E-09 | -0.54514 | 0.000485 | |  |
| 641 FBXO28 0.790824 | | | | 5.75E-09 | -0.72119 | 4.77E-07 | |  |
| 642 ERP29 0.790544 | | | | 5.87E-09 | -0.66511 | 7.10E-06 | |  |
| 643 CTCF 0.790184 | | | | 6.03E-09 | -0.83922 | 8.68E-11 | |  |
| 644 HNRPA1L-2 0.790054 | | | | 6.09E-09 | -0.67097 | 5.50E-06 | |  |
| 645 BRPF3 0.78944 | | | | 6.37E-09 | -0.90867 | 7.76E-15 | |  |
| 646 TSC22D1 0.789016 | | | | 6.58E-09 | -0.73791 | 1.87E-07 | |  |
| 647 PCDHA4 0.788871 | | | | 6.65E-09 | -0.70093 | 1.36E-06 | |  |
| 648 SPSB3 0.788779 | | | | 6.69E-09 | -0.68466 | 2.96E-06 | |  |
| 649 DHX9 | | 0.788459 | | 6.85E-09 | -0.66785 | 6.30E-06 | |  |
| 650 PHF21A | | 0.788264 | | 6.95E-09 | -0.80834 | 1.44E-09 | |  |
| 651 GLG1 | | 0.788039 | | 7.07E-09 | -0.61038 | 6.03E-05 | |  |
| 652 March 5 | | 0.78744 | | 7.39E-09 | -0.7412 | 1.55E-07 | |  |
| 653 PHF20L1 | | 0.787325 | | 7.45E-09 | -0.77398 | 1.93E-08 | |  |
| 654 PCMTD2 | | 0.787302 | | 7.47E-09 | -0.67101 | 5.49E-06 | |  |
| 655 MDGA1 | | 0.786831 | | 7.73E-09 | -0.7501 | 9.07E-08 | |  |
| 656 RP13-15M17.2 | | 0.786545 | | 7.89E-09 | -0.71198 | 7.76E-07 | |  |
| 657 ATP5A1 | | 0.786517 | | 7.91E-09 | -0.67923 | 3.80E-06 | |  |
| 658 KIAA1553 | | 0.786252 | | 8.06E-09 | -0.68713 | 2.64E-06 | |  |
| 659 RBM25 | | 0.786106 | | 8.15E-09 | -0.57938 | 0.000172 | |  |
| 660 IPO9 | | 0.785889 | | 8.28E-09 | -0.77482 | 1.82E-08 | |  |
| 661 PHC1 | | 0.784755 | | 8.99E-09 | -0.84404 | 5.30E-11 | |  |
| 662 LOC339123 | | 0.784334 | | 9.27E-09 | -0.56189 | 0.000296 | |  |
| 663 ACSS1 | | 0.783646 | | 9.75E-09 | -0.78051 | 1.22E-08 | |  |
| 664 FRMPD4 | | 0.783215 | | 1.01E-08 | -0.64124 | 1.90E-05 | |  |
| 665 HELLS | | 0.78282 | | 1.03E-08 | -0.78003 | 1.26E-08 | |  |
| 666 HMG20A | | 0.782388 | | 1.07E-08 | -0.50551 | 0.001415 | |  |
| 667 CXXC6 | | 0.782055 | | 1.09E-08 | -0.78459 | 9.10E-09 | |  |
| 668 HIC1 | | 0.78095 | | 1.18E-08 | -0.85027 | 2.74E-11 | |  |
| 669 EIF3S6IP | | 0.780694 | | 1.20E-08 | -0.60446 | 7.43E-05 | |  |
| 670 C1ORF164 | | 0.78032 | | 1.24E-08 | -0.79413 | 4.47E-09 | |  |
| 671 RBBP6 | | 0.779952 | | 1.27E-08 | -0.77979 | 1.28E-08 | |  |
| 672 AZIN1 | | 0.779714 | | 1.29E-08 | -0.77086 | 2.38E-08 | |  |
| 673 ZNF426 | | 0.779521 | | 1.31E-08 | -0.71401 | 6.98E-07 | |  |
| 674 LRIG2 | | 0.779458 | | 1.31E-08 | -0.86387 | 5.80E-12 | |  |
| 675 C1ORF85 | | 0.778785 | | 1.38E-08 | -0.8302 | 2.09E-10 | |  |
| 676 CLDN23 | | 0.778631 | | 1.39E-08 | -0.78459 | 9.11E-09 | |  |
| 677 CCNI | | 0.778602 | | 1.40E-08 | -0.50836 | 0.001316 | |  |
| 678 ZNF336 | | 0.778128 | | 1.44E-08 | -0.82011 | 5.27E-10 | |  |
| 679 SLC25A34 | | 0.777914 | | 1.47E-08 | -0.8927 | 1.14E-13 | |  |
| 680 HECTD1 | | 0.777868 | | 1.47E-08 | -0.65086 | 1.29E-05 | |  |
| 681 BPHL | | 0.777846 | | 1.47E-08 | -0.8604 | 8.74E-12 | |  |
| 682 MAP2K6 | | 0.77722 | | 1.54E-08 | -0.6889 | 2.43E-06 | |  |
| 683 THOC1 | | 0.777086 | | 1.55E-08 | -0.70535 | 1.09E-06 | |  |
| 684 SPRED1 | | 0.77674 | | 1.59E-08 | -0.50921 | 0.001288 | |  |
| 685 PRICKLE2 | | 0.776558 | | 1.61E-08 | -0.7641 | 3.74E-08 | |  |
| 686 PDCL | | 0.776475 | | 1.62E-08 | -0.57777 | 0.000181 | |  |
| 687 KCTD15 | | 0.775902 | | 1.69E-08 | -0.77317 | 2.04E-08 | |  |
| 688 RAE1 | | 0.775745 | | 1.71E-08 | -0.79144 | 5.48E-09 | |  |
| 689 ARIH2 | | 0.77544 | | 1.74E-08 | -0.57414 | 0.000203 | |  |
| 690 RAB40C | | 0.775372 | | 1.75E-08 | -0.68048 | 3.59E-06 | |  |
| 691 RNF175 | | 0.774927 | | 1.80E-08 | -0.72411 | 4.07E-07 | |  |
| 692 FLJ10154 | | 0.774157 | | 1.90E-08 | -0.56039 | 0.00031 | |  |
| 693 BLM | | 0.77371 | | 1.96E-08 | -0.8479 | 3.53E-11 | |  |
| 694 FLJ13236 | | 0.773486 | | 1.99E-08 | -0.86227 | 7.01E-12 | |  |
| 695 COL5A2 | | 0.772747 | | 2.10E-08 | -0.61228 | 5.64E-05 | |  |
| 696 KIAA0240 | | 0.772698 | | 2.10E-08 | -0.66587 | 6.87E-06 | |  |
| 697 KIAA1706 | | 0.771915 | | 2.22E-08 | -0.85515 | 1.60E-11 | |  |
| 698 FLJ14768 | | 0.771358 | | 2.30E-08 | -0.76894 | 2.71E-08 | |  |
| 699 USP37 | | 0.770996 | | 2.36E-08 | -0.73566 | 2.13E-07 | |  |
| 700 CLCN6 | | 0.770798 | | 2.39E-08 | -0.7838 | 9.64E-09 | |  |
| 701 LOC112869 | | 0.77024 | | 2.49E-08 | -0.71344 | 7.19E-07 | |  |
| 702 RPL13A | | 0.770174 | | 2.50E-08 | -0.51334 | 0.001157 | |  |
| 703 BTNL2 | | 0.770067 | | 2.51E-08 | -0.78317 | 1.01E-08 | |  |
| 704 SPIN | | 0.769702 | | 2.58E-08 | -0.82953 | 2.23E-10 | |  |
| 705 TOB1 | | 0.769602 | | 2.59E-08 | -0.57024 | 0.000229 | |  |
| 706 METTL7A | | 0.769144 | | 2.68E-08 | -0.59169 | 0.000115 | |  |
| 707 ZNF672 | | 0.768763 | | 2.74E-08 | -0.57862 | 0.000176 | |  |
| 708 LYK5 | | 0.768725 | | 2.75E-08 | -0.60883 | 6.38E-05 | |  |
| 709 TCF12 | | 0.768704 | | 2.76E-08 | -0.52589 | 0.000829 | |  |
| 710 WASF3 | | 0.768581 | | 2.78E-08 | -0.50294 | 0.001511 | |  |
| 711 C22ORF8 | | 0.767078 | | 3.07E-08 | -0.67626 | 4.35E-06 | |  |
| 712 PMPCB | | 0.767047 | | 3.08E-08 | -0.66489 | 7.17E-06 | |  |
| 713 TP53INP1 | | 0.766898 | | 3.11E-08 | -0.52237 | 0.000912 | |  |
| 714 ADNP | | 0.766258 | | 3.24E-08 | -0.57492 | 0.000198 | |  |
| 715 CPSF4 | | 0.765998 | | 3.30E-08 | -0.73982 | 1.68E-07 | |  |
| 716 LKAP | | 0.765828 | | 3.34E-08 | -0.61468 | 5.17E-05 | |  |
| 717 TIGD2 | | 0.764859 | | 3.56E-08 | -0.77556 | 1.73E-08 | |  |
| 718 CCNG2 | | 0.764285 | | 3.69E-08 | -0.57689 | 0.000186 | |  |
| 719 AGPAT4 | | 0.764266 | | 3.70E-08 | -0.72427 | 4.03E-07 | |  |
| 720 TAF6L | | 0.763757 | | 3.82E-08 | -0.85092 | 2.55E-11 | |  |
| 721 RPS15 | | 0.763408 | | 3.91E-08 | -0.68576 | 2.81E-06 | |  |
| 722 PRKRIP1 | | 0.762607 | | 4.12E-08 | -0.6403 | 1.97E-05 | |  |
| 723 NFIL3 | | 0.761702 | | 4.37E-08 | -0.85106 | 2.51E-11 | |  |
| 724 TGFBI | | 0.761578 | | 4.40E-08 | -0.84181 | 6.67E-11 | |  |
| 725 RIN2 | | 0.760635 | | 4.68E-08 | -0.86882 | 3.16E-12 | |  |
| 726 PRDM4 | | 0.760446 | | 4.74E-08 | -0.71667 | 6.07E-07 | |  |
| 727 CHODL | | 0.760297 | | 4.78E-08 | -0.49145 | 0.002009 | |  |
| 728 ZCCHC11 | | 0.760126 | | 4.84E-08 | -0.8361 | 1.18E-10 | |  |
| 729 FLJ37440 | | 0.760056 | | 4.86E-08 | -0.85509 | 1.61E-11 | |  |
| 730 RGS17 | | 0.75974 | | 4.96E-08 | -0.58596 | 0.000139 | |  |
| 731 ADM | | 0.759243 | | 5.12E-08 | -0.57767 | 0.000181 | |  |
| 732 ELAC1 | | 0.759189 | | 5.13E-08 | -0.64883 | 1.40E-05 | |  |
| 733 ANGPT1 | | 0.758795 | | 5.27E-08 | -0.57779 | 0.000181 | |  |
| 734 MLF1IP | | 0.757947 | | 5.56E-08 | -0.73967 | 1.69E-07 | |  |
| 735 ACVR1B | | 0.757424 | | 5.74E-08 | -0.61682 | 4.79E-05 | |  |
| 736 EDD1 | | 0.757196 | | 5.83E-08 | -0.54989 | 0.000423 | |  |
| 737 CECR5 | | 0.756795 | | 5.98E-08 | -0.5412 | 0.000543 | |  |
| 738 TCF7L2 | | 0.756568 | | 6.06E-08 | -0.78082 | 1.19E-08 | |  |
| 739 C14ORF145 | | 0.756496 | | 6.09E-08 | -0.6505 | 1.31E-05 | |  |
| 740 C16ORF53 | | 0.755773 | | 6.38E-08 | -0.68684 | 2.68E-06 | |  |
| 741 HNRPA3 | | 0.754857 | | 6.75E-08 | -0.76449 | 3.64E-08 | |  |
| 742 RPS2 | | 0.754386 | | 6.96E-08 | -0.51616 | 0.001075 | |  |
| 743 APP | | 0.754122 | | 7.07E-08 | -0.84259 | 6.16E-11 | |  |
| 744 BAZ2B | | 0.7538 | | 7.21E-08 | -0.67207 | 5.24E-06 | |  |
| 745 FLJ22795 | | 0.753695 | | 7.26E-08 | -0.57321 | 0.000209 | |  |
| 746 SYT11 | | 0.753308 | | 7.44E-08 | -0.65236 | 1.21E-05 | |  |
| 747 FECH | | 0.753171 | | 7.50E-08 | -0.6944 | 1.87E-06 | |  |
| 748 CHAF1A | | 0.752988 | | 7.59E-08 | -0.73628 | 2.06E-07 | |  |
| 749 STX16 | | 0.752844 | | 7.66E-08 | -0.55029 | 0.000418 | |  |
| 750 XRCC3 | | 0.752782 | | 7.69E-08 | -0.759 | 5.20E-08 | |  |
| 751 C11ORF11 | | 0.752406 | | 7.87E-08 | -0.63714 | 2.23E-05 | |  |
| 752 ILKAP | | 0.752192 | | 7.97E-08 | -0.62769 | 3.21E-05 | |  |
| 753 NCOA4 | | 0.752104 | | 8.02E-08 | -0.55614 | 0.000352 | |  |
| 754 WDR19 | | 0.75138 | | 8.38E-08 | -0.45071 | 0.005119 | |  |
| 755 NELF | | 0.751088 | | 8.53E-08 | -0.67346 | 4.93E-06 | |  |
| 756 CNP | | 0.750611 | | 8.79E-08 | -0.77627 | 1.64E-08 | |  |
| 757 USP3 | | 0.750483 | | 8.86E-08 | -0.82216 | 4.39E-10 | |  |
| 758 RAI1 | | 0.75001 | | 9.12E-08 | -0.6708 | 5.54E-06 | |  |
| 759 LPIN2 | | 0.750001 | | 9.12E-08 | -0.65132 | 1.27E-05 | |  |
| 760 PTPLAD1 | | 0.749724 | | 9.28E-08 | -0.68829 | 2.50E-06 | |  |
| 761 FANCC | | 0.749137 | | 9.62E-08 | -0.61253 | 5.59E-05 | |  |
| 762 SCAP | | 0.74905 | | 9.67E-08 | -0.50678 | 0.00137 | |  |
| 763 TRFP | | 0.748419 | | 1.00E-07 | -0.44137 | 0.006245 | |  |
| 764 GRB10 | | 0.747607 | | 1.06E-07 | -0.62153 | 4.03E-05 | |  |
| 765 TRPC6 | | 0.747473 | | 1.06E-07 | -0.67811 | 4.00E-06 | |  |
| 766 C8ORF13 | | 0.74591 | | 1.17E-07 | -0.60215 | 8.05E-05 | |  |
| 767 GPRC5C | | 0.745328 | | 1.21E-07 | -0.56101 | 0.000304 | |  |
| 768 ZNF507 | | 0.744974 | | 1.24E-07 | -0.68025 | 3.63E-06 | |  |
| 769 PAM | | 0.744933 | | 1.24E-07 | -0.60031 | 8.57E-05 | |  |
| 770 SLD5 | | 0.744794 | | 1.25E-07 | -0.5766 | 0.000188 | |  |
| 771 SLC45A4 | | 0.744109 | | 1.30E-07 | -0.63407 | 2.51E-05 | |  |
| 772 NSDHL | | 0.744004 | | 1.31E-07 | -0.69294 | 2.00E-06 | |  |
| 773 E4F1 | | 0.743619 | | 1.34E-07 | -0.64238 | 1.82E-05 | |  |
| 774 CPS1 | | 0.743177 | | 1.38E-07 | -0.5949 | 0.000103 | |  |
| 775 PDE4D | | 0.742707 | | 1.42E-07 | -0.45156 | 0.005025 | |  |
| 776 MSH6 | | 0.742353 | | 1.45E-07 | -0.54342 | 0.000509 | |  |
| 777 CDK10 | | 0.742343 | | 1.45E-07 | -0.72455 | 3.97E-07 | |  |
| 778 BRD8 | | 0.742071 | | 1.47E-07 | -0.64666 | 1.53E-05 | |  |
| 779 ZNF297B | | 0.741878 | | 1.49E-07 | -0.60852 | 6.45E-05 | |  |
| 780 VWA1 | | 0.741761 | | 1.50E-07 | -0.66171 | 8.21E-06 | |  |
| 781 ABHD11 | | 0.741217 | | 1.55E-07 | -0.59424 | 0.000105 | |  |
| 782 ACAA2 | | 0.740119 | | 1.65E-07 | -0.78684 | 7.72E-09 | |  |
| 783 ERV3 | | 0.739475 | | 1.71E-07 | -0.69847 | 1.53E-06 | |  |
| 784 MKRN1 | | 0.739222 | | 1.74E-07 | -0.52985 | 0.000745 | |  |
| 785 KCTD10 | | 0.738947 | | 1.76E-07 | -0.64694 | 1.51E-05 | |  |
| 786 APAF1 | | 0.738233 | | 1.84E-07 | -0.7389 | 1.77E-07 | |  |
| 787 PLK1 | | 0.737702 | | 1.90E-07 | -0.77808 | 1.45E-08 | |  |
| 788 DFFB | | 0.737638 | | 1.90E-07 | -0.73512 | 2.20E-07 | |  |
| 789 MAML1 | | 0.737589 | | 1.91E-07 | -0.55472 | 0.000367 | |  |
| 790 FLJ20364 | | 0.737102 | | 1.96E-07 | -0.54738 | 0.000455 | |  |
| 791 EEF2K | | 0.736633 | | 2.02E-07 | -0.69184 | 2.11E-06 | |  |
| 792 MDS028 | | 0.736547 | | 2.03E-07 | -0.84275 | 6.06E-11 | |  |
| 793 ELAVL1 | | 0.736471 | | 2.04E-07 | -0.65318 | 1.17E-05 | |  |
| 794 FLJ10379 | | 0.736467 | | 2.04E-07 | -0.6163 | 4.88E-05 | |  |
| 795 FLJ20280 | | 0.735725 | | 2.13E-07 | -0.65905 | 9.19E-06 | |  |
| 796 CHERP | | 0.735647 | | 2.14E-07 | -0.71864 | 5.47E-07 | |  |
| 797 C1ORF123 | | 0.735474 | | 2.16E-07 | -0.71544 | 6.48E-07 | |  |
| 798 KIAA0556 | | 0.735178 | | 2.19E-07 | -0.69285 | 2.01E-06 | |  |
| 799 TMED10 | | 0.734956 | | 2.22E-07 | -0.56512 | 0.000268 | |  |
| 800 FLJ45032 | | 0.734067 | | 2.34E-07 | -0.79402 | 4.51E-09 | |  |
| 801 March 7 | | 0.73379 | | 2.37E-07 | -0.70827 | 9.39E-07 | |  |
| 802 ITGA8 | | 0.733387 | | 2.43E-07 | -0.78716 | 7.55E-09 | |  |
| 803 OXA1L | | 0.732447 | | 2.56E-07 | -0.6482 | 1.44E-05 | |  |
| 804 NT5C3 | | 0.73236 | | 2.57E-07 | -0.75982 | 4.93E-08 | |  |
| 805 CBFB | | 0.731894 | | 2.64E-07 | -0.56676 | 0.000255 | |  |
| 806 XRCC5 | | 0.729827 | | 2.97E-07 | -0.61325 | 5.45E-05 | |  |
| 807 TRIM24 | | 0.729027 | | 3.10E-07 | -0.66824 | 6.20E-06 | |  |
| 808 ZNF690 | | 0.728768 | | 3.15E-07 | -0.69171 | 2.12E-06 | |  |
| 809 RCCD1 | | 0.728122 | | 3.26E-07 | -0.51476 | 0.001115 | |  |
| 810 LAMB1 | | 0.72785 | | 3.31E-07 | -0.60824 | 6.51E-05 | |  |
| 811 NFIA | | 0.72748 | | 3.38E-07 | -0.5684 | 0.000242 | |  |
| 812 DNAJC8 | | 0.727048 | | 3.46E-07 | -0.56538 | 0.000266 | |  |
| 813 NY-SAR-48 | | 0.727048 | | 3.46E-07 | -0.60887 | 6.37E-05 | |  |
| 814 SLC38A2 | | 0.726258 | | 3.62E-07 | -0.50092 | 0.001589 | |  |
| 815 ENOSF1 | | 0.726017 | | 3.67E-07 | -0.83254 | 1.67E-10 | |  |
| 816 SNN | | 0.72543 | | 3.79E-07 | -0.64071 | 1.94E-05 | |  |
| 817 ZNF266 | | 0.725247 | | 3.83E-07 | -0.77396 | 1.93E-08 | |  |
| 818 TENC1 | | 0.72515 | | 3.85E-07 | -0.7735 | 1.99E-08 | |  |
| 819 GCC1 | | 0.724771 | | 3.93E-07 | -0.49802 | 0.001709 | |  |
| 820 DERPC | | 0.724765 | | 3.93E-07 | -0.58885 | 0.000126 | |  |
| 821 KIAA0980 | | 0.724451 | | 4.00E-07 | -0.67357 | 4.90E-06 | |  |
| 822 PIK4CA | | 0.72395 | | 4.11E-07 | -0.76659 | 3.17E-08 | |  |
| 823 UCHL5IP | | 0.723768 | | 4.15E-07 | -0.61208 | 5.68E-05 | |  |
| 824 RPS4X | | 0.723441 | | 4.22E-07 | -0.45754 | 0.00441 | |  |
| 825 RG9MTD3 | | 0.723009 | | 4.32E-07 | -0.67358 | 4.90E-06 | |  |
| 826 NEK8 | | 0.722937 | | 4.34E-07 | -0.68751 | 2.59E-06 | |  |
| 827 MRPS27 | | 0.722684 | | 4.40E-07 | -0.77526 | 1.76E-08 | |  |
| 828 RC74 | | 0.722267 | | 4.50E-07 | -0.79338 | 4.73E-09 | |  |
| 829 MEIS3P1 | | 0.721392 | | 4.72E-07 | -0.82196 | 4.47E-10 | |  |
| 830 AHCTF1 | | 0.721376 | | 4.72E-07 | -0.75536 | 6.55E-08 | |  |
| 831 LUC7L | | 0.721166 | | 4.77E-07 | -0.7386 | 1.80E-07 | |  |
| 832 SOCS1 | | 0.721007 | | 4.82E-07 | -0.59328 | 0.000109 | |  |
| 833 FGD1 | | 0.72009 | | 5.06E-07 | -0.79117 | 5.60E-09 | |  |
| 834 C6ORF70 | | 0.720015 | | 5.08E-07 | -0.79579 | 3.94E-09 | |  |
| 835 HS6ST1 | | 0.719388 | | 5.25E-07 | -0.54523 | 0.000484 | |  |
| 836 TMEM106C | | 0.719318 | | 5.27E-07 | -0.61459 | 5.19E-05 | |  |
| 837 BOP1 | | 0.718412 | | 5.53E-07 | -0.51874 | 0.001004 | |  |
| 838 CYORF15A | | 0.718405 | | 5.54E-07 | -0.63631 | 2.30E-05 | |  |
| 839 FBXO33 | | 0.718227 | | 5.59E-07 | -0.80439 | 2.00E-09 | |  |
| 840 ARID5B | | 0.716881 | | 6.00E-07 | -0.72399 | 4.10E-07 | |  |
| 841 CLCN7 | | 0.716354 | | 6.17E-07 | -0.66064 | 8.59E-06 | |  |
| 842 PRKDC | | 0.715921 | | 6.31E-07 | -0.58003 | 0.000168 | |  |
| 843 TRHDE | | 0.715496 | | 6.46E-07 | -0.62726 | 3.26E-05 | |  |
| 844 TPCN2 | | 0.714272 | | 6.88E-07 | -0.65567 | 1.06E-05 | |  |
| 845 ILVBL | | 0.714166 | | 6.92E-07 | -0.55745 | 0.000338 | |  |
| 846 ZC3H3 | | 0.713431 | | 7.19E-07 | -0.80519 | 1.87E-09 | |  |
| 847 WDR57 | | 0.713355 | | 7.22E-07 | -0.55749 | 0.000338 | |  |
| 848 SUPT16H | | 0.712829 | | 7.42E-07 | -0.69963 | 1.45E-06 | |  |
| 849 EIF4A1 | | 0.711166 | | 8.09E-07 | -0.84773 | 3.60E-11 | |  |
| 850 TXNL4B | | 0.709737 | | 8.71E-07 | -0.75366 | 7.28E-08 | |  |
| 851 CTF8 | | 0.709488 | | 8.82E-07 | -0.54871 | 0.000437 | |  |
| 852 CTDSPL2 | | 0.709364 | | 8.88E-07 | -0.52897 | 0.000763 | |  |
| 853 TSPAN32 | | 0.707691 | | 9.67E-07 | -0.54864 | 0.000438 | |  |
| 854 LOC440944 | | 0.707554 | | 9.74E-07 | -0.70336 | 1.20E-06 | |  |
| 855 PEX16 | | 0.706698 | | 1.02E-06 | -0.66622 | 6.77E-06 | |  |
| 856 SLC2A12 | | 0.705938 | | 1.06E-06 | -0.66807 | 6.24E-06 | |  |
| 857 LARS2 | | 0.704879 | | 1.11E-06 | -0.59763 | 9.40E-05 | |  |
| 858 SMURF1 | | 0.703979 | | 1.17E-06 | -0.606 | 7.04E-05 | |  |
| 859 FLJ13305 | | 0.703287 | | 1.21E-06 | -0.70685 | 1.01E-06 | |  |
| 860 U2AF1L4 | | 0.702633 | | 1.25E-06 | -0.648 | 1.45E-05 | |  |
| 861 PIK3R3 | | 0.702089 | | 1.28E-06 | -0.49225 | 0.00197 | |  |
| 862 ANKRD41 | | 0.701814 | | 1.30E-06 | -0.68424 | 3.02E-06 | |  |
| 863 CASP6 | | 0.700793 | | 1.37E-06 | -0.72826 | 3.24E-07 | |  |
| 864 C3ORF30 | | 0.69985 | | 1.43E-06 | -0.7272 | 3.44E-07 | |  |
| 865 ZBED1 | | 0.699376 | | 1.47E-06 | -0.76985 | 2.55E-08 | |  |
| 866 USP52 | | 0.699124 | | 1.48E-06 | -0.5638 | 0.000279 | |  |
| 867 RSAFD1 | | 0.698563 | | 1.53E-06 | -0.49912 | 0.001663 | |  |
| 868 TCF4 | | 0.698558 | | 1.53E-06 | -0.5059 | 0.001402 | |  |
| 869 OTUB1 | | 0.698509 | | 1.53E-06 | -0.67649 | 4.30E-06 | |  |
| 870 ZXDC | | 0.697786 | | 1.58E-06 | -0.48711 | 0.002232 | |  |
| 871 RAPGEFL1 | | 0.697469 | | 1.61E-06 | -0.75467 | 6.83E-08 | |  |
| 872 ZBTB33 | | 0.69708 | | 1.64E-06 | -0.53723 | 0.000607 | |  |
| 873 NISCH | | 0.696371 | | 1.70E-06 | -0.70365 | 1.19E-06 | |  |
| 874 ZFYVE26 | | 0.695577 | | 1.76E-06 | -0.58934 | 0.000124 | |  |
| 875 SELENBP1 | | 0.695179 | | 1.80E-06 | -0.57322 | 0.000209 | |  |
| 876 NUP160 | | 0.695118 | | 1.80E-06 | -0.63589 | 2.34E-05 | |  |
| 877 C9ORF39 | | 0.694984 | | 1.82E-06 | -0.77226 | 2.17E-08 | |  |
| 878 C10ORF119 | | 0.694917 | | 1.82E-06 | -0.63007 | 2.93E-05 | |  |
| 879 TSEN54 | | 0.694599 | | 1.85E-06 | -0.7243 | 4.03E-07 | |  |
| 880 SPIRE2 | | 0.694174 | | 1.89E-06 | -0.76889 | 2.72E-08 | |  |
| 881 NGDN | | 0.693548 | | 1.95E-06 | -0.79311 | 4.83E-09 | |  |
| 882 ZW10 | | 0.693288 | | 1.97E-06 | -0.4753 | 0.00295 | |  |
| 883 E2F6 | | 0.693176 | | 1.98E-06 | -0.52429 | 0.000866 | |  |
| 884 ISCA2 | | 0.692649 | | 2.03E-06 | -0.65547 | 1.07E-05 | |  |
| 885 TRIAD3 | | 0.692645 | | 2.03E-06 | -0.50825 | 0.00132 | |  |
| 886 RPIA | | 0.692611 | | 2.04E-06 | -0.68405 | 3.05E-06 | |  |
| 887 PFTK1 | | 0.692545 | | 2.04E-06 | -0.6605 | 8.64E-06 | |  |
| 888 VWCE | | 0.692437 | | 2.05E-06 | -0.73933 | 1.73E-07 | |  |
| 889 PEX1 | | 0.691759 | | 2.12E-06 | -0.53492 | 0.000647 | |  |
| 890 MPDU1 | | 0.691589 | | 2.14E-06 | -0.61057 | 5.99E-05 | |  |
| 891 BCKDHA | | 0.691005 | | 2.20E-06 | -0.68977 | 2.33E-06 | |  |
| 892 AVIL | | 0.690461 | | 2.26E-06 | -0.60255 | 7.94E-05 | |  |
| 893 DKFZP564O0523 0.690293 | | | | 2.27E-06 | -0.46072 | 0.00411 | |  |
| 894 ZNF358 0.690233 | | | | 2.28E-06 | -0.64195 | 1.85E-05 | |  |
| 895 ZNF96 0.689927 | | | | 2.31E-06 | -0.60056 | 8.50E-05 | |  |
| 896 SLC37A3 0.689701 | | | | 2.34E-06 | -0.51136 | 0.001218 | |  |
| 897 RPS25 0.689381 | | | | 2.37E-06 | -0.52283 | 0.000901 | |  |
| 898 FOXQ1 0.689322 | | | | 2.38E-06 | -0.81503 | 8.21E-10 | |  |
| 899 EHMT1 | | 0.688942 | | 2.42E-06 | -0.71417 | 6.92E-07 | |  |
| 900 C13ORF3 | | 0.688796 | | 2.44E-06 | -0.7659 | 3.32E-08 | |  |
| 901 FLJ90757 | | 0.688273 | | 2.50E-06 | -0.51625 | 0.001072 | |  |
| 902 SYT1 | | 0.687964 | | 2.54E-06 | -0.67357 | 4.90E-06 | |  |
| 903 BRMS1 | | 0.687819 | | 2.55E-06 | -0.75562 | 6.44E-08 | |  |
| 904 ZNF543 | | 0.687527 | | 2.59E-06 | -0.51129 | 0.00122 | |  |
| 905 TAF1B | | 0.687453 | | 2.60E-06 | -0.63169 | 2.75E-05 | |  |
| 906 PPAT | | 0.686095 | | 2.77E-06 | -0.55689 | 0.000344 | |  |
| 907 RBMS3 | | 0.685731 | | 2.82E-06 | -0.48421 | 0.002392 | |  |
| 908 USP46 | | 0.684643 | | 2.96E-06 | -0.56915 | 0.000237 | |  |
| 909 SPRED2 | | 0.683419 | | 3.14E-06 | -0.50433 | 0.001458 | |  |
| 910 CARD8 | | 0.683369 | | 3.14E-06 | -0.70193 | 1.29E-06 | |  |
| 911 TMEM43 | | 0.6833 | | 3.15E-06 | -0.50119 | 0.001579 | |  |
| 912 GLT8D2 | | 0.68289 | | 3.21E-06 | -0.42628 | 0.008517 | |  |
| 913 C14ORF130 | | 0.682716 | | 3.24E-06 | -0.73314 | 2.46E-07 | |  |
| 914 HRB2 | | 0.682079 | | 3.34E-06 | -0.46232 | 0.003966 | |  |
| 915 THUMPD1 | | 0.681915 | | 3.36E-06 | -0.51381 | 0.001143 | |  |
| 916 WHDC1 | | 0.681534 | | 3.42E-06 | -0.64753 | 1.48E-05 | |  |
| 917 PELI2 | | 0.678909 | | 3.86E-06 | -0.69871 | 1.51E-06 | |  |
| 918 PACSIN2 | | 0.677599 | | 4.09E-06 | -0.40499 | 0.012901 | |  |
| 919 SRP9 | | 0.676582 | | 4.28E-06 | -0.62259 | 3.88E-05 | |  |
| 920 CAPN3 | | 0.675275 | | 4.54E-06 | -0.73556 | 2.15E-07 | |  |
| 921 NUP35 | | 0.674779 | | 4.64E-06 | -0.56515 | 0.000268 | |  |
| 922 BUB1B | | 0.674658 | | 4.67E-06 | -0.68773 | 2.57E-06 | |  |
| 923 AQR | | 0.674228 | | 4.76E-06 | -0.46864 | 0.003439 | |  |
| 924 TIFA | | 0.67376 | | 4.86E-06 | -0.61212 | 5.67E-05 | |  |
| 925 PTHLH | | 0.673545 | | 4.91E-06 | -0.52861 | 0.00077 | |  |
| 926 GAB1 | | 0.672234 | | 5.20E-06 | -0.70413 | 1.16E-06 | |  |
| 927 DPY19L2 | | 0.671949 | | 5.27E-06 | -0.62707 | 3.28E-05 | |  |
| 928 LAMC1 | | 0.671919 | | 5.27E-06 | -0.41445 | 0.010763 | |  |
| 929 ABCC1 | | 0.671119 | | 5.46E-06 | -0.60254 | 7.94E-05 | |  |
| 930 RLF | | 0.670395 | | 5.64E-06 | -0.70617 | 1.04E-06 | |  |
| 931 NQO1 | | 0.670313 | | 5.66E-06 | -0.73433 | 2.30E-07 | |  |
| 932 PHLPP | | 0.670243 | | 5.68E-06 | -0.74633 | 1.14E-07 | |  |
| 933 CARM1 | | 0.670138 | | 5.70E-06 | -0.47666 | 0.002858 | |  |
| 934 SKP2 | | 0.669663 | | 5.82E-06 | -0.59514 | 0.000102 | |  |
| 935 POLA1 | | 0.669592 | | 5.84E-06 | -0.58017 | 0.000167 | |  |
| 936 LOC652924 | | 0.669484 | | 5.87E-06 | -0.61408 | 5.29E-05 | |  |
| 937 CRSP6 | | 0.668404 | | 6.15E-06 | -0.55668 | 0.000346 | |  |
| 938 GALNAC4S-6ST | | 0.667507 | | 6.40E-06 | -0.4226 | 0.009169 | |  |
| 939 RRAGB | | 0.66737 | | 6.44E-06 | -0.45538 | 0.004624 | |  |
| 940 LOC90639 | | 0.667237 | | 6.47E-06 | -0.5827 | 0.000154 | |  |
| 941 CCS | | 0.666338 | | 6.73E-06 | -0.66643 | 6.71E-06 | |  |
| 942 MKL2 | | 0.666242 | | 6.76E-06 | -0.56211 | 0.000294 | |  |
| 943 ZNF643 | | 0.666003 | | 6.83E-06 | -0.46576 | 0.003671 | |  |
| 944 RNMTL1 | | 0.665921 | | 6.85E-06 | -0.67885 | 3.87E-06 | |  |
| 945 FXC1 | | 0.665903 | | 6.86E-06 | -0.37456 | 0.022372 | |  |
| 946 HCAP-D3 | | 0.66529 | | 7.04E-06 | -0.55844 | 0.000328 | |  |
| 947 EPHB3 | | 0.664934 | | 7.15E-06 | -0.52841 | 0.000774 | |  |
| 948 ZNF32 | | 0.664895 | | 7.16E-06 | -0.67401 | 4.81E-06 | |  |
| 949 GATS | | 0.664704 | | 7.22E-06 | -0.52354 | 0.000884 | |  |
| 950 KIAA1160 | | 0.663916 | | 7.47E-06 | -0.62077 | 4.15E-05 | |  |
| 951 SNRPD1 | | 0.663526 | | 7.60E-06 | -0.52768 | 0.00079 | |  |
| 952 HNRPUL2 | | 0.662755 | | 7.85E-06 | -0.58239 | 0.000156 | |  |
| 953 C11ORF71 | | 0.661678 | | 8.22E-06 | -0.49177 | 0.001993 | |  |
| 954 VPS4B | | 0.661461 | | 8.30E-06 | -0.687 | 2.66E-06 | |  |
| 955 TMEM69 | | 0.660965 | | 8.48E-06 | -0.45509 | 0.004653 | |  |
| 956 FLJ90396 | | 0.659805 | | 8.90E-06 | -0.66509 | 7.10E-06 | |  |
| 957 YPEL1 | | 0.659474 | | 9.03E-06 | -0.52374 | 0.000879 | |  |
| 958 ITGB3 | | 0.659324 | | 9.08E-06 | -0.63977 | 2.01E-05 | |  |
| 959 MGC20470 | | 0.658999 | | 9.21E-06 | -0.61913 | 4.40E-05 | |  |
| 960 FRS3 | | 0.657662 | | 9.74E-06 | -0.42113 | 0.00944 | |  |
| 961 ASB7 | | 0.657518 | | 9.80E-06 | -0.72395 | 4.11E-07 | |  |
| 962 ATP8B4 | | 0.657414 | | 9.84E-06 | -0.5717 | 0.000219 | |  |
| 963 ORC1L | | 0.657241 | | 9.92E-06 | -0.61591 | 4.95E-05 | |  |
| 964 FZD1 | | 0.657136 | | 9.96E-06 | -0.49788 | 0.001715 | |  |
| 965 GTPBP3 | | 0.655479 | | 1.07E-05 | -0.64686 | 1.52E-05 | |  |
| 966 AMIGO3 | | 0.655274 | | 1.08E-05 | -0.5361 | 0.000626 | |  |
| 967 LOC144404 | | 0.654634 | | 1.11E-05 | -0.56047 | 0.000309 | |  |
| 968 SPPL3 | | 0.654568 | | 1.11E-05 | -0.63187 | 2.73E-05 | |  |
| 969 DCI | | 0.653827 | | 1.14E-05 | -0.46124 | 0.004062 | |  |
| 970 RAD54L | | 0.65365 | | 1.15E-05 | -0.70276 | 1.24E-06 | |  |
| 971 ZNF6 | | 0.65363 | | 1.15E-05 | -0.47083 | 0.003271 | |  |
| 972 GBGT1 | | 0.653512 | | 1.16E-05 | -0.50636 | 0.001385 | |  |
| 973 PTPN13 | | 0.65349 | | 1.16E-05 | -0.40451 | 0.01302 | |  |
| 974 C6ORF64 | | 0.652919 | | 1.19E-05 | -0.4922 | 0.001973 | |  |
| 975 GRM2 | | 0.652291 | | 1.22E-05 | -0.6554 | 1.07E-05 | |  |
| 976 DKFZP434K1815 0.651802 | | | | 1.24E-05 | -0.41561 | 0.010521 | |  |
| 977 DHX37 0.651636 | | | | 1.25E-05 | -0.58897 | 0.000126 | |  |
| 978 RNF44 0.650304 | | | | 1.32E-05 | -0.73009 | 2.93E-07 | |  |
| 979 AADAT 0.650042 | | | | 1.33E-05 | -0.57729 | 0.000183 | |  |
| 980 NARG1L 0.650029 | | | | 1.34E-05 | -0.38473 | 0.018712 | |  |
| 981 BTBD12 0.649371 | | | | 1.37E-05 | -0.4768 | 0.002849 | |  |
| 982 C15ORF15 0.649126 | | | | 1.39E-05 | -0.44655 | 0.005597 | |  |
| 983 ACTR5 0.648074 | | | | 1.45E-05 | -0.50262 | 0.001523 | |  |
| 984 SOX12 0.647355 | | | | 1.49E-05 | -0.5795 | 0.000171 | |  |
| 985 WNT5A 0.646968 | | | | 1.51E-05 | -0.64676 | 1.53E-05 | |  |
| 986 HIST1H4J 0.646872 | | | | 1.52E-05 | -0.52911 | 0.00076 | |  |
| 987 JMJD1A 0.646871 | | | | 1.52E-05 | -0.59181 | 0.000114 | |  |
| 988 FAU 0.645106 | | | | 1.63E-05 | -0.77051 | 2.44E-08 | |  |
| 989 DGCR13 0.644699 | | | | 1.66E-05 | -0.47421 | 0.003026 | |  |
| 990 RAB11B 0.644535 | | | | 1.67E-05 | -0.70042 | 1.39E-06 | |  |
| 991 ILF2 0.643502 | | | | 1.74E-05 | -0.57603 | 0.000191 | |  |
| 992 DCLRE1B 0.643323 | | | | 1.75E-05 | -0.64916 | 1.38E-05 | |  |
| 993 C12ORF45 0.643057 | | | | 1.77E-05 | -0.5166 | 0.001063 | |  |
| 994 RNF34 0.642355 | | | | 1.82E-05 | -0.49911 | 0.001663 | |  |
| 995 MGC52057 0.642185 | | | | 1.83E-05 | -0.5084 | 0.001315 | |  |
| 996 KBTBD4 0.641038 | | | | 1.92E-05 | -0.55723 | 0.00034 | |  |
| 997 PTCD1 0.640231 | | | | 1.98E-05 | -0.37328 | 0.022872 | |  |
| 998 HP1BP3 0.6402 | | | | 1.98E-05 | -0.68735 | 2.61E-06 | |  |
| 999 ZNF614 | | | 0.639424 | | 2.04E-05 | -0.6066 | 6.90E-05 | |
| 1000 RPL18A | | | 0.639324 | | 2.05E-05 | -0.47913 | 0.002697 | |
| 1001 TPST1 | | | 0.638658 | | 2.10E-05 | -0.70085 | 1.36E-06 | |
| 1002 USP21 | | | 0.638609 | | 2.11E-05 | -0.69529 | 1.79E-06 | |
| 1003 RANBP2 | | | 0.638342 | | 2.13E-05 | -0.52808 | 0.000781 | |
| 1004 LOC51035 | | | 0.638168 | | 2.14E-05 | -0.75539 | 6.53E-08 | |
| 1005 SAP30BP | | | 0.637777 | | 2.18E-05 | -0.70114 | 1.34E-06 | |
| 1006 USP6 | | | 0.637622 | | 2.19E-05 | -0.50692 | 0.001366 | |
| 1007 REV1L | | | 0.637535 | | 2.20E-05 | -0.53618 | 0.000625 | |
| 1008 CMTM1 | | | 0.637175 | | 2.23E-05 | -0.60119 | 8.32E-05 | |
| 1009 EXOSC6 | | | 0.636589 | | 2.28E-05 | -0.74032 | 1.63E-07 | |
| 1010 CEP76 | | | 0.636419 | | 2.30E-05 | -0.49027 | 0.002068 | |
| 1011 USP48 | | | 0.636263 | | 2.31E-05 | -0.41163 | 0.011367 | |
| 1012 ADSL | | | 0.634489 | | 2.47E-05 | -0.43932 | 0.00652 | |
| 1013 PDK4 | | | 0.633089 | | 2.61E-05 | -0.70312 | 1.22E-06 | |
| 1014 GOSR1 | | | 0.631607 | | 2.76E-05 | -0.53201 | 0.000702 | |
| 1015 JMJD2A | | | 0.631025 | | 2.82E-05 | -0.53038 | 0.000734 | |
| 1016 LOC654129 | | | 0.630815 | | 2.85E-05 | -0.61441 | 5.23E-05 | |
| 1017 SART1 | | | 0.62928 | | 3.02E-05 | -0.63877 | 2.09E-05 | |
| 1018 YTHDF1 | | | 0.629116 | | 3.04E-05 | -0.61236 | 5.62E-05 | |
| 1019 ZNF16 | | | 0.628981 | | 3.05E-05 | -0.45773 | 0.004392 | |
| 1020 C17ORF56 | | | 0.628737 | | 3.08E-05 | -0.71016 | 8.52E-07 | |
| 1021 GATA3 | | | 0.626278 | | 3.38E-05 | -0.55435 | 0.000371 | |
| 1022 UBQLN2 | | | 0.625959 | | 3.42E-05 | -0.37231 | 0.023257 | |
| 1023 ARRB1 | | | 0.625458 | | 3.49E-05 | -0.55363 | 0.000379 | |
| 1024 PLEKHA5 | | | 0.625184 | | 3.52E-05 | -0.48141 | 0.002557 | |
| 1025 SPIN1 | | | 0.625136 | | 3.53E-05 | -0.55015 | 0.000419 | |
| 1026 RSC1A1 | | | 0.624903 | | 3.56E-05 | -0.52122 | 0.00094 | |
| 1027 PHLDB1 | | | 0.624869 | | 3.56E-05 | -0.35726 | 0.029953 | |
| 1028 H2AFY | | | 0.624308 | | 3.64E-05 | -0.67468 | 4.66E-06 | |
| 1029 DDX6 | | | 0.624292 | | 3.64E-05 | -0.55374 | 0.000378 | |
| 1030 UIP1 | | | 0.623175 | | 3.79E-05 | -0.53559 | 0.000635 | |
| 1031 RMI1 | | | 0.623109 | | 3.80E-05 | -0.41676 | 0.010289 | |
| 1032 GNPTG | | | 0.622192 | | 3.93E-05 | -0.4869 | 0.002243 | |
| 1033 CXORF15 | | | 0.622077 | | 3.95E-05 | -0.54717 | 0.000457 | |
| 1034 ZNF561 | | | 0.620955 | | 4.12E-05 | -0.39392 | 0.015851 | |
| 1035 C14ORF101 | | | 0.619893 | | 4.28E-05 | -0.40112 | 0.013874 | |
| 1036 MT1H | | | 0.619285 | | 4.38E-05 | -0.45517 | 0.004646 | |
| 1037 TBX1 | | | 0.618677 | | 4.48E-05 | -0.52226 | 0.000914 | |
| 1038 ZNF512 | | | 0.618598 | | 4.49E-05 | -0.58732 | 0.000133 | |
| 1039 NOL11 | | | 0.617992 | | 4.59E-05 | -0.32977 | 0.046236 | |
| 1040 BTF3L4 | | | 0.617258 | | 4.71E-05 | -0.39512 | 0.015505 | |
| 1041 WDR18 | | | 0.615962 | | 4.94E-05 | -0.42112 | 0.009443 | |
| 1042 SYF2 | | | 0.614449 | | 5.22E-05 | -0.46009 | 0.004168 | |
| 1043 ALF | | | 0.614356 | | 5.24E-05 | -0.59416 | 0.000106 | |
| 1044 CDC7 | | | 0.614044 | | 5.29E-05 | -0.5303 | 0.000735 | |
| 1045 ZNF510 | | | 0.613766 | | 5.35E-05 | -0.46504 | 0.003731 | |
| 1046 FLJ37464 | | | 0.612891 | | 5.52E-05 | -0.56791 | 0.000246 | |
| 1047 DUSP4 | | | 0.612296 | | 5.64E-05 | -0.67496 | 4.61E-06 | |
| 1048 STGC3 | | | 0.61208 | | 5.68E-05 | -0.54534 | 0.000482 | |
| 1049 NAT11 | | | 0.610801 | 5.95E-05 | | -0.62582 | 3.44E-05 | |
| 1050 UBE2O | | | 0.60963 | 6.20E-05 | | -0.48859 | 0.002153 | |
| 1051 RPN1 | | | 0.609562 | 6.21E-05 | | -0.48486 | 0.002355 | |
| 1052 LOC143543 | | | 0.609384 | 6.25E-05 | | -0.54506 | 0.000486 | |
| 1053 PGRMC1 | | | 0.609055 | 6.32E-05 | | -0.34495 | 0.036536 | |
| 1054 LOC126536 | | | 0.607231 | 6.74E-05 | | -0.49689 | 0.001758 | |
| 1055 SFRS14 | | | 0.606832 | 6.84E-05 | | -0.43693 | 0.006853 | |
| 1056 C9ORF112 | | | 0.606173 | 7.00E-05 | | -0.56508 | 0.000269 | |
| 1057 EDA2R | | | 0.605673 | 7.12E-05 | | -0.53849 | 0.000586 | |
| 1058 SP4 | | | 0.604855 | 7.33E-05 | | -0.35904 | 0.029085 | |
| 1059 REPS1 | | | 0.60472 | 7.36E-05 | | -0.44171 | 0.006201 | |
| 1060 ZNF694 | | | 0.604379 | 7.45E-05 | | -0.37691 | 0.021478 | |
| 1061 SLITRK6 | | | 0.604027 | 7.54E-05 | | -0.41501 | 0.010646 | |
| 1062 RAB34 | | | 0.603971 | 7.56E-05 | | -0.52746 | 0.000795 | |
| 1063 GANAB | | | 0.603524 | 7.68E-05 | | -0.63792 | 2.17E-05 | |
| 1064 MCC | | | 0.602566 | 7.93E-05 | | -0.43118 | 0.007713 | |
| 1065 THG1L | | | 0.600619 | 8.49E-05 | | -0.62356 | 3.74E-05 | |
| 1066 CD81 | | | 0.600046 | 8.65E-05 | | -0.37508 | 0.022171 | |
| 1067 PHF17 | | | 0.599784 | 8.73E-05 | | -0.43434 | 0.00723 | |
| 1068 PTMA | | | 0.599458 | 8.83E-05 | | -0.57051 | 0.000227 | |
| 1069 DDX27 | | | 0.599098 | 8.94E-05 | | -0.49118 | 0.002022 | |
| 1070 UPF3B | | | 0.596236 | 9.85E-05 | | -0.40703 | 0.012412 | |
| 1071 WWTR1 | | | 0.595494 | 0.000101 | | -0.56232 | 0.000292 | |
| 1072 LRPPRC | | | 0.595341 | 0.000102 | | -0.41126 | 0.011448 | |
| 1073 RPS27 | | | 0.595247 | 0.000102 | | -0.56477 | 0.000271 | |
| 1074 RPS6KA5 | | | 0.595002 | 0.000103 | | -0.63604 | 2.33E-05 | |
| 1075 PGAP1 | | | 0.593935 | 0.000106 | | -0.43821 | 0.006673 | |
| 1076 ADORA2A | | | 0.59238 | 0.000112 | | -0.6836 | 3.11E-06 | |
| 1077 FLJ14166 | | | 0.592365 | 0.000112 | | -0.63486 | 2.44E-05 | |
| 1078 LOC124751 | | | 0.592285 | 0.000113 | | -0.45993 | 0.004182 | |
| 1079 FLJ38964 | | | 0.591807 | 0.000114 | | -0.63362 | 2.56E-05 | |
| 1080 THBS4 | | | 0.590566 | 0.000119 | | -0.51405 | 0.001136 | |
| 1081 UCK1 | | | 0.590015 | 0.000121 | | -0.50744 | 0.001347 | |
| 1082 LOC653281 | | | 0.589898 | 0.000122 | | -0.42463 | 0.008805 | |
| 1083 GAS1 | | | 0.589494 | 0.000123 | | -0.44711 | 0.00553 | |
| 1084 C2ORF29 | | | 0.588882 | 0.000126 | | -0.34117 | 0.038782 | |
| 1085 CYCS | | | 0.587852 | 0.00013 | | -0.43669 | 0.006887 | |
| 1086 UBE2Q2 | | | 0.587178 | 0.000133 | | -0.53982 | 0.000564 | |
| 1087 SHPRH | | | 0.586195 | 0.000138 | | -0.34614 | 0.035854 | |
| 1088 ZNF560 | | | 0.586156 | 0.000138 | | -0.49549 | 0.001819 | |
| 1089 POLB | | | 0.585698 | 0.00014 | | -0.66495 | 7.15E-06 | |
| 1090 NOP5/NOP58 | | | 0.584254 | 0.000147 | | -0.52766 | 0.00079 | |
| 1091 CACNG7 | | | 0.583725 | 0.000149 | | -0.43569 | 0.00703 | |
| 1092 ZNF236 | | | 0.583707 | 0.000149 | | -0.5406 | 0.000552 | |
| 1093 SMARCD2 | | | 0.582629 | 0.000155 | | -0.58944 | 0.000124 | |
| 1094 TAF1 | | | 0.582305 | 0.000156 | | -0.49106 | 0.002028 | |
| 1095 HIST1H2BH | | | 0.582037 | 0.000158 | | -0.634 | 2.52E-05 | |
| 1096 GALT | | | 0.58184 | 0.000159 | | -0.47109 | 0.003252 | |
| 1097 ZNF34 | | | 0.581305 | 0.000161 | | -0.65611 | 1.04E-05 | |
| 1098 MGC10471 | | | 0.580876 | 0.000164 | | -0.41106 | 0.011491 | |
| 1099 CCDC47 | | | 0.58081 | 0.000164 | | -0.53787 | 0.000596 | |
| 1100 DOCK1 | | | 0.58075 | 0.000164 | | -0.58143 | 0.000161 | |
| 1101 HIST1H4C | | | 0.580679 | 0.000165 | | -0.47972 | 0.00266 | |
| 1102 FLCN | | | 0.580587 | 0.000165 | | -0.63604 | 2.33E-05 | |
| 1103 ARHGAP4 | | | 0.580119 | 0.000168 | | -0.4601 | 0.004167 | |
| 1104 SMPDL3A | | | 0.579265 | 0.000172 | | -0.36481 | 0.026418 | |
| 1105 SFPQ | | | 0.578163 | 0.000178 | | -0.59675 | 9.68E-05 | |
| 1106 RPL27A | | | 0.575564 | 0.000194 | | -0.419 | 0.009846 | |
| 1107 GNB4 | | | 0.57532 | 0.000195 | | -0.56005 | 0.000313 | |
| 1108 FLJ30655 | | | 0.575284 | 0.000196 | | -0.5417 | 0.000535 | |
| 1109 PRR3 | | | 0.574661 | 0.000199 | | -0.42423 | 0.008875 | |
| 1110 FBXL12 | | | 0.5745 | 0.0002 | | -0.56427 | 0.000275 | |
| 1111 IFT81 | | | 0.57402 | 0.000203 | | -0.36891 | 0.024648 | |
| 1112 XPO4 | | | 0.57359 | 0.000206 | | -0.48986 | 0.002088 | |
| 1113 RPL5 | | | 0.573494 | 0.000207 | | -0.3282 | 0.047346 | |
| 1114 PSPC1 | | | 0.573401 | 0.000207 | | -0.47825 | 0.002754 | |
| 1115 KIAA1576 | | | 0.57227 | 0.000215 | | -0.68693 | 2.66E-06 | |
| 1116 HCN3 | | | 0.571684 | 0.000219 | | -0.58062 | 0.000165 | |
| 1117 C14ORF2 | | | 0.571127 | 0.000223 | | -0.53547 | 0.000637 | |
| 1118 RAD50 | | | 0.570221 | 0.000229 | | -0.42664 | 0.008457 | |
| 1119 FZR1 | | | 0.570069 | 0.00023 | | -0.57141 | 0.000221 | |
| 1120 C9ORF64 | | | 0.569331 | 0.000236 | | -0.48179 | 0.002534 | |
| 1121 MAP3K14 | | | 0.569167 | 0.000237 | | -0.66126 | 8.37E-06 | |
| 1122 ATP8B2 | | | 0.568751 | 0.00024 | | -0.36102 | 0.028147 | |
| 1123 DDX31 | | | 0.568656 | 0.000241 | | -0.51422 | 0.001131 | |
| 1124 WASF1 | | | 0.568503 | 0.000242 | | -0.43794 | 0.006711 | |
| 1125 CARD11 | | | 0.567968 | 0.000246 | | -0.53466 | 0.000652 | |
| 1126 ITM2C | | | 0.567321 | 0.000251 | | -0.50387 | 0.001476 | |
| 1127 ZBTB26 | | | 0.567184 | 0.000252 | | -0.5833 | 0.000151 | |
| 1128 LBX2 | | | 0.566676 | 0.000256 | | -0.66606 | 6.81E-06 | |
| 1129 SNTB1 | | | 0.566032 | 0.000261 | | -0.42282 | 0.009129 | |
| 1130 RPL30 | | | 0.565172 | 0.000268 | | -0.53386 | 0.000667 | |
| 1131 ZFYVE1 | | | 0.564897 | 0.00027 | | -0.44419 | 0.005885 | |
| 1132 LDLRAD3 | | | 0.564475 | 0.000274 | | -0.34736 | 0.035165 | |
| 1133 HIST1H3H | | | 0.564464 | 0.000274 | | -0.52887 | 0.000765 | |
| 1134 TMTC2 | | | 0.562225 | 0.000293 | | -0.4013 | 0.013829 | |
| 1135 RNF168 | | | 0.56212 | 0.000294 | | -0.45505 | 0.004658 | |
| 1136 CAST1 | | | 0.561629 | 0.000298 | | -0.44253 | 0.006096 | |
| 1137 LOC390637 | | | 0.561136 | 0.000303 | | -0.35793 | 0.029624 | |
| 1138 SNRPD3 | | | 0.560564 | 0.000308 | | -0.3735 | 0.022786 | |
| 1139 RCOR3 | | | 0.558712 | 0.000326 | | -0.34923 | 0.034125 | |
| 1140 PTPN18 | | | 0.558628 | 0.000326 | | -0.43076 | 0.00778 | |
| 1141 PCNX | | | 0.558369 | 0.000329 | | -0.4486 | 0.005356 | |
| 1142 DENND4C | | | 0.5578 | 0.000335 | | -0.57389 | 0.000204 | |
| 1143 HIST1H2BG | | | 0.557716 | 0.000335 | | -0.5089 | 0.001298 | |
| 1144 AHRR | | | 0.557592 | 0.000337 | | -0.62595 | 3.42E-05 | |
| 1145 CNTN6 | | | 0.557344 | 0.000339 | | -0.526 | 0.000827 | |
| 1146 FAM48A | | | 0.556495 | 0.000348 | | -0.45745 | 0.004419 | |
| 1147 RPS15A | | | 0.556488 | 0.000348 | | -0.37295 | 0.023001 | |
| 1148 HGF | | | 0.556105 | 0.000352 | | -0.55077 | 0.000412 | |
| 1149 PHB2 | | | 0.555019 | 0.000363 | | -0.48131 | 0.002562 | |
| 1150 SOS2 | | | 0.554497 | 0.000369 | | -0.56426 | 0.000275 | |
| 1151 GSTO2 | | | 0.55376 | 0.000377 | | -0.62221 | 3.93E-05 | |
| 1152 GPIAP1 | | | 0.553515 | 0.00038 | | -0.35502 | 0.031069 | |
| 1153 FAM76B | | | 0.553476 | 0.00038 | | -0.49415 | 0.00188 | |
| 1154 NLGN2 | | | 0.552996 | 0.000386 | | -0.54879 | 0.000436 | |
| 1155 CENPO | | | 0.552908 | 0.000387 | | -0.50372 | 0.001481 | |
| 1156 G1P3 | | | 0.55286 | 0.000387 | | -0.42669 | 0.008448 | |
| 1157 TRAFD1 | | | 0.552325 | 0.000394 | | -0.53459 | 0.000653 | |
| 1158 FLJ30834 | | | 0.55198 | 0.000398 | | -0.43425 | 0.007243 | |
| 1159 GPC2 | | | 0.551005 | 0.000409 | | -0.5299 | 0.000744 | |
| 1160 ZNF212 | | | 0.54962 | 0.000426 | | -0.42434 | 0.008857 | |
| 1161 HTRA3 | | | 0.548894 | 0.000435 | | -0.52746 | 0.000795 | |
| 1162 KIAA1961 | | | 0.548551 | 0.000439 | | -0.5856 | 0.00014 | |
| 1163 TBC1D5 | | | 0.548054 | 0.000446 | | -0.61855 | 4.50E-05 | |
| 1164 ACADSB | | | 0.547455 | 0.000454 | | -0.37053 | 0.023975 | |
| 1165 MGC10911 | | | 0.546547 | 0.000466 | | -0.41007 | 0.011713 | |
| 1166 NVL | | | 0.545935 | 0.000474 | | -0.36665 | 0.025612 | |
| 1167 CDC42SE1 | | | 0.544384 | 0.000496 | | -0.61744 | 4.68E-05 | |
| 1168 ESPL1 | | | 0.542737 | 0.000519 | | -0.3918 | 0.016476 | |
| 1169 CHRNA10 | | | 0.542108 | 0.000529 | | -0.5897 | 0.000123 | |
| 1170 LIX1L | | | 0.541409 | 0.000539 | | -0.52775 | 0.000788 | |
| 1171 KIAA1875 | | | 0.540902 | 0.000547 | | -0.37365 | 0.022725 | |
| 1172 SSH2 | | | 0.540808 | 0.000549 | | -0.45192 | 0.004987 | |
| 1173 SLC24A4 | | | 0.539225 | 0.000574 | | -0.49105 | 0.002028 | |
| 1174 REXO4 | | | 0.539165 | 0.000575 | | -0.41436 | 0.010782 | |
| 1175 EPR1 | | | 0.538592 | 0.000584 | | -0.48707 | 0.002233 | |
| 1176 ERH | | | 0.538412 | 0.000587 | | -0.53152 | 0.000711 | |
| 1177 DSEL | | | 0.538343 | 0.000588 | | -0.46731 | 0.003544 | |
| 1178 IFIT5 | | | 0.538245 | 0.00059 | | -0.48468 | 0.002366 | |
| 1179 SSH1 | | | 0.538118 | 0.000592 | | -0.46499 | 0.003735 | |
| 1180 WDR5 | | | 0.537914 | 0.000595 | | -0.61697 | 4.76E-05 | |
| 1181 MASK-BP3 | | | 0.536962 | 0.000611 | | -0.50472 | 0.001444 | |
| 1182 RPS16 | | | 0.536088 | 0.000627 | | -0.39153 | 0.016557 | |
| 1183 ZBTB39 | | | 0.53302 | 0.000682 | | -0.40969 | 0.011797 | |
| 1184 MTG1 | | | 0.532442 | 0.000693 | | -0.38771 | 0.017741 | |
| 1185 ZNF713 | | | 0.531258 | 0.000716 | | -0.4645 | 0.003776 | |
| 1186 DHX8 | | | 0.529749 | 0.000747 | | -0.59656 | 9.74E-05 | |
| 1187 GTF2IRD2P | | | 0.528564 | 0.000771 | | -0.41137 | 0.011424 | |
| 1188 ADAM8 | | | 0.528338 | 0.000776 | | -0.58818 | 0.000129 | |
| 1189 ZSCAN5 | | | 0.528204 | 0.000779 | | -0.4013 | 0.013828 | |
| 1190 RNF2 | | | 0.527895 | 0.000785 | | -0.49436 | 0.00187 | |
| 1191 HSDL2 | | | 0.527606 | 0.000792 | | -0.59924 | 8.90E-05 | |
| 1192 RCC1 | | | 0.527085 | 0.000803 | | -0.55012 | 0.00042 | |
| 1193 FGFR1OP | | | 0.526727 | 0.000811 | | -0.44162 | 0.006214 | |
| 1194 ZNF184 | | | 0.526346 | 0.000819 | | -0.39189 | 0.016448 | |
| 1195 RHOBTB1 | | | 0.526308 | 0.00082 | | -0.49887 | 0.001673 | |
| 1196 ADRA1B | | | 0.524557 | 0.00086 | | -0.51083 | 0.001235 | |
| 1197 HIST1H2AM | | | 0.523893 | 0.000875 | | -0.53869 | 0.000583 | |
| 1198 GNB2L1 | | | 0.523267 | 0.00089 | | -0.44162 | 0.006212 | |
| 1199 RHBDL3 | | | 0.522321 | 0.000913 | | -0.51792 | 0.001026 | |
| 1200 NOL9 | | | 0.520481 | 0.000959 | | -0.44082 | 0.006319 | |
| 1201 PRPF40B | | | 0.519824 | 0.000976 | | -0.55832 | 0.000329 | |
| 1202 SEMA4C | | | 0.519734 | 0.000978 | | -0.46411 | 0.00381 | |
| 1203 STARD8 | | | 0.519666 | 0.00098 | | -0.47452 | 0.003004 | |
| 1204 PLA2G6 | | | 0.519164 | 0.000993 | | -0.42203 | 0.009274 | |
| 1205 FOXK2 | | | 0.518984 | 0.000998 | | -0.35605 | 0.030551 | |
| 1206 CDK5RAP1 | | | 0.516865 | 0.001055 | | -0.51728 | 0.001044 | |
| 1207 FAM72A | | | 0.515117 | 0.001105 | | -0.40221 | 0.013595 | |
| 1208 FOXI1 | | | 0.513407 | 0.001155 | | -0.40935 | 0.011876 | |
| 1209 KLK6 | | | 0.512827 | 0.001173 | | -0.50668 | 0.001374 | |
| 1210 ARHGEF9 | | | 0.51236 | 0.001187 | | -0.3596 | 0.028818 | |
| 1211 C5ORF13 | | | 0.511919 | 0.001201 | | -0.41663 | 0.010314 | |
| 1212 LOC284194 | | | 0.510922 | 0.001232 | | -0.46983 | 0.003347 | |
| 1213 KCNS1 | | | 0.509864 | 0.001266 | | -0.43037 | 0.007841 | |
| 1214 PUM2 | | | 0.506801 | 0.00137 | | -0.44049 | 0.006362 | |
| 1215 March 6 | | | 0.506401 | 0.001384 | | -0.40813 | 0.012155 | |
| 1216 KIAA0553 | | | 0.506187 | 0.001391 | | -0.43604 | 0.00698 | |
| 1217 ZNF740 | | | 0.501632 | 0.001561 | | -0.3918 | 0.016477 | |
| 1218 RABGAP1 | | | 0.499911 | 0.00163 | | -0.44841 | 0.005379 | |
| 1219 SLIT1 | | | 0.498461 | 0.00169 | | -0.41424 | 0.010805 | |
| 1220 LOC643057 | | | 0.497398 | 0.001735 | | -0.38666 | 0.018078 | |
| 1221 HTN3 | | | 0.49649 | 0.001775 | | -0.33404 | 0.043318 | |
| 1222 WISP2 | | | 0.4953 | 0.001828 | | -0.4586 | 0.004308 | |
| 1223 STOM | | | 0.494367 | 0.00187 | | -0.5214 | 0.000936 | |
| 1224 DKC1 | | | 0.49428 | 0.001874 | | -0.46414 | 0.003807 | |
| 1225 UBB | | | 0.493658 | 0.001903 | | -0.34344 | 0.037421 | |
| 1226 GNAI1 | | | 0.492611 | 0.001953 | | -0.37412 | 0.022543 | |
| 1227 JMY | | | 0.490379 | 0.002062 | | -0.52247 | 0.000909 | |
| 1228 PPP2R2D | | | 0.489481 | 0.002107 | | -0.40297 | 0.013401 | |
| 1229 LLGL1 | | | 0.487337 | 0.002219 | | -0.5181 | 0.001021 | |
| 1230 FLJ25102 | | | 0.485905 | 0.002297 | | -0.44668 | 0.005581 | |
| 1231 DNAH1 | | | 0.484373 | 0.002383 | | -0.37488 | 0.022248 | |
| 1232 LNPEP | | | 0.483701 | 0.002421 | | -0.50729 | 0.001353 | |
| 1233 CEP57 | | | 0.482993 | 0.002462 | | -0.32595 | 0.048984 | |
| 1234 RPL37 | | | 0.48242 | 0.002496 | | -0.35805 | 0.029564 | |
| 1235 PRPF38A | | | 0.481221 | 0.002568 | | -0.47746 | 0.002805 | |
| 1236 GLUL | | | 0.479338 | 0.002685 | | -0.38587 | 0.018336 | |
| 1237 GABRE | | | 0.477589 | 0.002797 | | -0.3581 | 0.029539 | |
| 1238 SSTR3 | | | 0.47707 | 0.002831 | | -0.51179 | 0.001205 | |
| 1239 OR1J1 | | | 0.476757 | 0.002852 | | -0.35846 | 0.029363 | |
| 1240 SAMD11 | | | 0.476409 | 0.002875 | | -0.37366 | 0.022722 | |
| 1241 NFKBIL1 | | | 0.475512 | 0.002936 | | -0.50053 | 0.001605 | |
| 1242 MECP2 | | | 0.475101 | 0.002964 | | -0.41516 | 0.010615 | |
| 1243 SLC27A6 | | | 0.475022 | 0.002969 | | -0.35557 | 0.030791 | |
| 1244 KIF13A | | | 0.473277 | 0.003092 | | -0.59208 | 0.000113 | |
| 1245 RPS11 | | | 0.473053 | 0.003108 | | -0.42018 | 0.00962 | |
| 1246 THRAP6 | | | 0.472048 | 0.003181 | | -0.38601 | 0.018291 | |
| 1247 FLJ20323 | | | 0.471833 | 0.003196 | | -0.33324 | 0.043857 | |
| 1248 OR5D16 | | | 0.471653 | 0.00321 | | -0.35221 | 0.032522 | |
| 1249 LOC389458 0.469435 | | | | 0.003377 | | -0.41221 | 0.011239 | |
| 1250 C9ORF90 0.46698 | | | | 0.003571 | | -0.54449 | 0.000494 | |
| 1251 CTNNBL1 0.465215 | | | | 0.003716 | | -0.45268 | 0.004905 | |
| 1252 BTNL9 0.46508 | | | | 0.003728 | | -0.50027 | 0.001616 | |
| 1253 MC1R 0.464166 | | | | 0.003805 | | -0.40974 | 0.011786 | |
| 1254 ZNF320 0.463866 | | | | 0.003831 | | -0.37014 | 0.024136 | |
| 1255 ZNF571 0.46371 | | | | 0.003844 | | -0.34351 | 0.03738 | |
| 1256 DKFZP667G2110 0.462769 | | | | 0.003926 | | -0.37043 | 0.024019 | |
| 1257 MPND | | | 0.459581 | 0.004215 | | -0.40103 | 0.013897 | |
| 1258 BRI3BP | | | 0.458959 | 0.004274 | | -0.39594 | 0.015273 | |
| 1259 LOC440686 | | | 0.458598 | 0.004308 | | -0.49688 | 0.001758 | |
| 1260 E2F4 | | | 0.455087 | 0.004654 | | -0.50675 | 0.001372 | |
| 1261 EMR2 | | | 0.4549 | 0.004673 | | -0.32695 | 0.048253 | |
| 1262 DSCR1 | | | 0.452761 | 0.004896 | | -0.34412 | 0.03702 | |
| 1263 MARK3 | | | 0.452065 | 0.004971 | | -0.38882 | 0.017391 | |
| 1264 TOB2 | | | 0.451993 | 0.004978 | | -0.40667 | 0.012498 | |
| 1265 PMP22CD | | | 0.449918 | 0.005207 | | -0.33607 | 0.041989 | |
| 1266 NPAS3 | | | 0.44975 | 0.005226 | | -0.50693 | 0.001365 | |
| 1267 SMAF1 | | | 0.448975 | 0.005313 | | -0.41417 | 0.010821 | |
| 1268 THEM4 | | | 0.446547 | 0.005597 | | -0.44076 | 0.006326 | |
| 1269 PET112L | | | 0.446385 | 0.005617 | | -0.36206 | 0.027662 | |
| 1270 ZNF673 | | | 0.443974 | 0.005912 | | -0.3805 | 0.020169 | |
| 1271 LY6G5C | | | 0.442159 | 0.006143 | | -0.50059 | 0.001603 | |
| 1272 NCK2 | | | 0.44195 | 0.00617 | | -0.33953 | 0.039792 | |
| 1273 C1ORF91 | | | 0.435476 | 0.007062 | | -0.37667 | 0.021566 | |
| 1274 SAV1 | | | 0.431585 | 0.00765 | | -0.49793 | 0.001713 | |
| 1275 PLA2G12B | | | 0.429075 | 0.008051 | | -0.41081 | 0.011547 | |
| 1276 RFX3 | | | 0.428128 | 0.008206 | | -0.49038 | 0.002062 | |
| 1277 KIAA0082 | | | 0.426849 | 0.008421 | | -0.43432 | 0.007232 | |
| 1278 ACAT2 | | | 0.426248 | 0.008524 | | -0.4534 | 0.004828 | |
| 1279 SERHL | | | 0.42608 | 0.008552 | | -0.35932 | 0.028948 | |
| 1280 C22ORF15 | | | 0.425844 | 0.008593 | | -0.32802 | 0.047481 | |
| 1281 NFASC | | | 0.42226 | 0.009231 | | -0.32968 | 0.046304 | |
| 1282 KIAA1285 | | | 0.422084 | 0.009263 | | -0.3329 | 0.044082 | |
| 1283 CCDC28A | | | 0.419604 | 0.009729 | | -0.35398 | 0.031604 | |
| 1284 UCN3 | | | 0.416446 | 0.010352 | | -0.3699 | 0.024237 | |
| 1285 SLAMF9 | | | 0.412169 | 0.011248 | | -0.40606 | 0.012642 | |
| 1286 FLJ13910 | | | 0.411159 | 0.01147 | | -0.42685 | 0.008421 | |
| 1287 ANKS1A | | | 0.405072 | 0.012882 | | -0.37394 | 0.022612 | |
| 1288 PHYHIP | | | 0.404938 | 0.012914 | | -0.34496 | 0.036529 | |
| 1289 RYR2 | | | 0.403186 | 0.013348 | | -0.33216 | 0.044583 | |
| 1290 POM121 | | | 0.394526 | 0.015676 | | -0.34852 | 0.034514 | |
| 1291 PCDHA2 | | | 0.394507 | 0.015682 | | -0.34414 | 0.03701 | |
| 1292 NOVA1 | | | 0.392562 | 0.016249 | | -0.32936 | 0.046527 | |
| 1293 DNM1L | | | 0.392554 | 0.016252 | | -0.32642 | 0.048638 | |
| 1294 METTL9 | | | 0.388306 | 0.017552 | | -0.3584 | 0.029395 | |
| 1295 COL23A1 | | | 0.385355 | 0.018505 | | -0.33724 | 0.041233 | |
| 1296 KIF27 | | | 0.384821 | 0.018682 | | -0.40064 | 0.013999 | |
| 1297 LOC388524 | | | 0.381044 | 0.019976 | | -0.37328 | 0.022869 | |
| 1298 TRIM59 | | | 0.378691 | 0.020819 | | -0.35228 | 0.032488 | |
| 1299 COPG2 | | | 0.378383 | 0.020931 | | -0.45194 | 0.004984 | |
| 1300 ZNF195 | | | 0.3761 | 0.021781 | | -0.38226 | 0.019551 | |
| 1301 RELN | | | 0.37422 | 0.022503 | | -0.32934 | 0.046538 | |
| 1302 MRPS18A | | | 0.373458 | 0.0228 | | -0.34087 | 0.038967 | |
| 1303 PCDHGB7 | | | 0.371369 | 0.023634 | | -0.40891 | 0.011974 | |
| 1304 GCDH | | | 0.370296 | 0.024072 | | -0.41382 | 0.010895 | |
| 1305 PTGER1 | | | 0.363662 | 0.026931 | | -0.39469 | 0.01563 | |
| 1306 PCSK4 | | | 0.358895 | 0.029154 | | -0.35282 | 0.032202 | |
| 1307 BRD4 | | | 0.356304 | 0.030424 | | -0.36264 | 0.027394 | |
| 1308 C20ORF14 | | | 0.344371 | 0.036875 | | -0.37601 | 0.021815 | |
| 1309 LOC161247 | | | 0.344269 | 0.036934 | | -0.33765 | 0.040973 | |
| 1310 RPL38 | | | 0.340543 | 0.039165 | | -0.37592 | 0.021851 | |
| 1311 POU2AF1 | | | 0.328788 | 0.046931 | | -0.35121 | 0.033053 | |
