# Supplementary material for: Identification of SCARA3 with potential roles in metabolic disorders
Source: Aging (Albany NY). 2020 Dec 9;13(2):2149–67. doi: 10.18632/aging.202228 (PMC7880357; doi:10.18632/aging.202228)
Supplement: Supplementary Table 3 [file aging-13-202228-s004.docx]

Supplementary Table 3: Association with adipogenic stages in green model.

| moduleGenes | MM | MMP | GS | GSP |
| --- | --- | --- | --- | --- |
| 1 ITGA11 | 0.97658 | 6.06E-25 | 0.701227 | 1.34E-06 |
| 2 APOD | 0.976073 | 8.79E-25 | 0.815203 | 8.09E-10 |
| 3 PCK2 | 0.97014 | 4.05E-23 | 0.705223 | 1.10E-06 |
| 4 EIF2B2 | 0.967511 | 1.74E-22 | 0.645026 | 1.63E-05 |
| 5 ACYP2 | 0.964495 | 8.01E-22 | 0.83761 | 1.02E-10 |
| 6 MMP11 | 0.960775 | 4.45E-21 | 0.612109 | 5.67E-05 |
| 7 TLCD1 | 0.960673 | 4.65E-21 | 0.791141 | 5.61E-09 |
| 8 SIRT4 | 0.960321 | 5.42E-21 | 0.751134 | 8.51E-08 |
| 9 TIMP1 | 0.959812 | 6.75E-21 | 0.857377 | 1.24E-11 |
| 10 PC | 0.959551 | 7.54E-21 | 0.743547 | 1.35E-07 |
| 11 TBC1D7 | 0.957503 | 1.76E-20 | 0.819347 | 5.64E-10 |
| 12 SAR1B | 0.957279 | 1.93E-20 | 0.791948 | 5.28E-09 |
| 13 DSCR5 | 0.954732 | 5.20E-20 | 0.662477 | 7.95E-06 |
| 14 LPPR2 | 0.954033 | 6.76E-20 | 0.810463 | 1.21E-09 |
| 15 DXS9879E | 0.952867 | 1.04E-19 | 0.676707 | 4.26E-06 |
| 16 PTGDS | 0.95271 | 1.10E-19 | 0.830943 | 1.95E-10 |
| 17 ZNF425 | 0.949441 | 3.45E-19 | 0.607715 | 6.63E-05 |
| 18 C15ORF24 | 0.947682 | 6.19E-19 | 0.727281 | 3.42E-07 |
| 19 ARHGEF2 | 0.947383 | 6.82E-19 | 0.826872 | 2.85E-10 |
| 20 CREG1 | 0.94561 | 1.20E-18 | 0.895285 | 7.61E-14 |
| 21 THAP6 | 0.945551 | 1.22E-18 | 0.815384 | 7.97E-10 |
| 22 SRPR | 0.945013 | 1.45E-18 | 0.809703 | 1.29E-09 |
| 23 NFE2L1 | 0.944484 | 1.70E-18 | 0.880953 | 6.39E-13 |
| 24 RUNDC1 | 0.943338 | 2.41E-18 | 0.639646 | 2.02E-05 |
| 25 SH3BP5L | 0.942691 | 2.93E-18 | 0.735345 | 2.17E-07 |
| 26 ASS | 0.94268 | 2.94E-18 | 0.898406 | 4.60E-14 |
| 27 NUPR1 | 0.940891 | 4.96E-18 | 0.827343 | 2.73E-10 |
| 28 SERINC2 | 0.940434 | 5.66E-18 | 0.74545 | 1.20E-07 |
| 29 P8 | 0.940197 | 6.05E-18 | 0.819255 | 5.68E-10 |
| 30 C9ORF16 | 0.939831 | 6.72E-18 | 0.664846 | 7.18E-06 |
| 31 NXPH4 | 0.939748 | 6.88E-18 | 0.662099 | 8.08E-06 |
| 32 UBE2D4 | 0.939471 | 7.43E-18 | 0.892634 | 1.15E-13 |
| 33 LRRC29 | 0.938882 | 8.76E-18 | 0.678743 | 3.89E-06 |
| 34 C2ORF7 | 0.93747 | 1.29E-17 | 0.839478 | 8.45E-11 |
| 35 ANKRD37 | 0.9368 | 1.55E-17 | 0.702823 | 1.24E-06 |
| 36 MYO1D | 0.936159 | 1.84E-17 | 0.866982 | 3.97E-12 |
| 37 FLJ20489 | 0.93601 | 1.91E-17 | 0.865332 | 4.85E-12 |
| 38 PEX6 | 0.935036 | 2.47E-17 | 0.742126 | 1.46E-07 |
| 39 CXXC5 | 0.935034 | 2.47E-17 | 0.582734 | 0.000154 |
| 40 TGM2 | 0.934798 | 2.63E-17 | 0.872676 | 1.93E-12 |
| 41 DYM | 0.934777 | 2.65E-17 | 0.775185 | 1.77E-08 |
| 42 ZNF18 | 0.933996 | 3.24E-17 | 0.803887 | 2.08E-09 |
| 43 KIAA0256 | 0.933617 | 3.57E-17 | 0.687984 | 2.54E-06 |
| 44 NDUFAF1 | 0.932893 | 4.29E-17 | 0.602584 | 7.93E-05 |
| 45 C14ORF143 | 0.932615 | 4.60E-17 | 0.615589 | 5.01E-05 |
| 46 NAGLU | 0.931522 | 6.04E-17 | 0.571219 | 0.000222 |
| 47 PTCD2 | 0.930685 | 7.42E-17 | 0.708045 | 9.50E-07 |
| 48 WDR20 | 0.93053 | 7.71E-17 | 0.623681 | 3.72E-05 |

| 49 GPR137 | 0.930335 | 8.09E-17 | 0.652386 | 1.21E-05 |  |
| --- | --- | --- | --- | --- | --- |
| 50 CTSD | 0.930262 | 8.23E-17 | 0.88141 | 5.99E-13 |  |
| 51 C7ORF36 | 0.929498 | 9.90E-17 | 0.722561 | 4.43E-07 |  |
| 52 EHD3 | 0.929372 | 1.02E-16 | 0.812417 | 1.03E-09 |  |
| 53 C3ORF39 | 0.928175 | 1.36E-16 | 0.827469 | 2.70E-10 |  |
| 54 ATP13A2 | 0.928069 | 1.39E-16 | 0.772009 | 2.20E-08 |  |
| 55 RPP38 | 0.927404 | 1.62E-16 | 0.648766 | 1.41E-05 |  |
| 56 ZNF419 | 0.926573 | 1.97E-16 | 0.85072 | 2.61E-11 |  |
| 57 HAX1 | 0.926491 | 2.01E-16 | 0.645685 | 1.59E-05 |  |
| 58 LARGE | 0.926079 | 2.21E-16 | 0.726989 | 3.48E-07 |  |
| 59 TMEM129 | 0.924964 | 2.84E-16 | 0.716674 | 6.07E-07 |  |
| 60 CMKLR1 | 0.924369 | 3.25E-16 | 0.790155 | 6.04E-09 |  |
| 61 BCAP31 | 0.923908 | 3.60E-16 | 0.80253 | 2.32E-09 |  |
| 62 ZNHIT2 | 0.923893 | 3.61E-16 | 0.742745 | 1.41E-07 |  |
| 63 SCAMP5 | 0.923767 | 3.71E-16 | 0.605587 | 7.14E-05 |  |
| 64 ZNF416 | 0.923366 | 4.05E-16 | 0.772845 | 2.08E-08 |  |
| 65 ASS1 | 0.923275 | 4.14E-16 | 0.902235 | 2.42E-14 |  |
| 66 FYN | 0.923247 | 4.16E-16 | 0.858431 | 1.10E-11 |  |
| 67 TMEM8 | 0.922961 | 4.43E-16 | 0.783006 | 1.02E-08 |  |
| 68 MGC12966 | 0.922188 | 5.24E-16 | 0.727953 | 3.30E-07 |  |
| 69 GGCX | 0.921541 | 6.03E-16 | 0.69909 | 1.49E-06 |  |
| 70 IGFBP7 | 0.920927 | 6.88E-16 | 0.804218 | 2.02E-09 |  |
| 71 TERF2 | 0.920798 | 7.07E-16 | 0.68341 | 3.14E-06 |  |
| 72 MAD2L1BP | 0.919924 | 8.51E-16 | 0.729284 | 3.06E-07 |  |
| 73 MARS | 0.919488 | 9.32E-16 | 0.793836 | 4.57E-09 |  |
| 74 CHPF | 0.919198 | 9.90E-16 | 0.854523 | 1.71E-11 |  |
| 75 MAN1C1 | 0.918895 | 1.05E-15 | 0.711775 | 7.84E-07 |  |
| 76 TMEM150 | 0.918625 | 1.12E-15 | 0.825587 | 3.21E-10 |  |
| 77 TM2D2 | 0.918501 | 1.14E-15 | 0.551904 | 0.000398 |  |
| 78 FAM45A | 0.917862 | 1.31E-15 | 0.743547 | 1.35E-07 |  |
| 79 FZD8 | 0.91734 | 1.45E-15 | 0.798208 | 3.26E-09 |  |
| 80 NCDN | 0.916669 | 1.66E-15 | 0.615129 | 5.09E-05 |  |
| 81 C1ORF122 | 0.916078 | 1.87E-15 | 0.774282 | 1.89E-08 |  |
| 82 LOC92270 | 0.913729 | 2.98E-15 | 0.800975 | 2.62E-09 |  |
| 83 FCGBP | 0.911192 | 4.85E-15 | 0.629315 | 3.01E-05 |  |
| 84 CTH | 0.910698 | 5.32E-15 | 0.83406 | 1.44E-10 |  |
| 85 F25965 | 0.910567 | 5.45E-15 | 0.70744 | 9.79E-07 |  |
| 86 ASPHD1 | 0.910549 | 5.47E-15 | 0.797373 | 3.48E-09 |  |
| 87 TBPL1 | 0.910149 | 5.90E-15 | 0.780219 | 1.25E-08 |  |
| 88 GLRX2 | 0.909998 | 6.07E-15 | 0.84538 | 4.61E-11 |  |
| 89 ARHGEF3 | 0.909915 | 6.16E-15 | 0.528306 | 0.000777 |  |
| 90 ABCB6 | 0.909681 | 6.43E-15 | 0.631109 | 2.82E-05 |  |
| 91 C1QDC1 | 0.909068 | 7.21E-15 | 0.715693 | 6.39E-07 |  |
| 92 PRSS15 | 0.908259 | 8.36E-15 | 0.58177 | 0.000159 |  |
| 93 ADM2 | 0.90759 | 9.44E-15 | 0.858856 | 1.05E-11 |  |
| 94 CLCN3 | 0.907453 | 9.68E-15 | 0.574825 | 0.000198 |  |
| 95 ZNF177 | 0.907283 | 9.98E-15 | 0.594537 | 0.000104 |  |
| 96 PPIC | 0.907156 | 1.02E-14 | 0.880108 | 7.18E-13 |  |
| 97 NEIL2 | 0.906494 | 1.15E-14 | 0.839588 | 8.36E-11 |  |
| 98 DOCK6 | 0.90646 | 1.16E-14 | 0.610025 | 6.11E-05 |  |
| 99 PPA1 | | 0.906409 | 1.17E-14 | 0.911378 | 4.68E-15 |
| 100 TXNL5 | | 0.906035 | 1.25E-14 | 0.711257 | 8.05E-07 |
| 101 ZNF589 | | 0.90556 | 1.36E-14 | 0.604817 | 7.34E-05 |
| 102 ABHD4 | | 0.905433 | 1.39E-14 | 0.87695 | 1.10E-12 |
| 103 MRPL40 | | 0.904691 | 1.58E-14 | 0.688649 | 2.46E-06 |
| 104 DNAJC1 | | 0.904582 | 1.61E-14 | 0.749572 | 9.37E-08 |
| 105 POP5 | | 0.90322 | 2.04E-14 | 0.774747 | 1.83E-08 |
| 106 TOR1B | | 0.902867 | 2.17E-14 | 0.836271 | 1.16E-10 |
| 107 NUDT18 | | 0.90267 | 2.25E-14 | 0.865937 | 4.51E-12 |
| 108 FLJ20422 | | 0.902507 | 2.31E-14 | 0.65699 | 1.00E-05 |
| 109 NUDT16 | | 0.901943 | 2.55E-14 | 0.811417 | 1.12E-09 |
| 110 DLL3 | | 0.90171 | 2.65E-14 | 0.769006 | 2.70E-08 |
| 111 HSPC171 | | 0.90155 | 2.72E-14 | 0.509692 | 0.001272 |
| 112 PEX11B | | 0.901469 | 2.76E-14 | 0.883895 | 4.22E-13 |
| 113 DCUN1D5 | | 0.901444 | 2.77E-14 | 0.514459 | 0.001124 |
| 114 INHBE | | 0.901185 | 2.90E-14 | 0.865923 | 4.52E-12 |
| 115 NAPB | | 0.900464 | 3.27E-14 | 0.925954 | 2.27E-16 |
| 116 SLC35E3 | | 0.900168 | 3.43E-14 | 0.612891 | 5.52E-05 |
| 117 KIAA0828 | | 0.89946 | 3.86E-14 | 0.547552 | 0.000452 |
| 118 ROBO2 | | 0.899376 | 3.92E-14 | 0.810365 | 1.22E-09 |
| 119 GRK4 | | 0.899361 | 3.93E-14 | 0.864959 | 5.08E-12 |
| 120 CYP1B1 | | 0.897734 | 5.13E-14 | 0.801877 | 2.44E-09 |
| 121 MRPL24 | | 0.897049 | 5.74E-14 | 0.681197 | 3.47E-06 |
| 122 CHAC1 | | 0.896575 | 6.19E-14 | 0.560337 | 0.00031 |
| 123 ZNF621 | | 0.895575 | 7.27E-14 | 0.831558 | 1.84E-10 |
| 124 ALPK1 | | 0.895163 | 7.76E-14 | 0.578038 | 0.000179 |
| 125 C7ORF30 | | 0.89478 | 8.24E-14 | 0.734711 | 2.25E-07 |
| 126 NPC2 | | 0.894481 | 8.64E-14 | 0.502196 | 0.001539 |
| 127 RBM21 | | 0.894284 | 8.92E-14 | 0.48201 | 0.00252 |
| 128 GPR177 | | 0.894055 | 9.24E-14 | 0.824113 | 3.68E-10 |
| 129 GPX3 | | 0.893991 | 9.34E-14 | 0.854909 | 1.64E-11 |
| 130 HEAB | | 0.893698 | 9.78E-14 | 0.507617 | 0.001341 |
| 131 OPRS1 | | 0.892432 | 1.19E-13 | 0.590018 | 0.000121 |
| 132 AQP11 | | 0.892029 | 1.27E-13 | 0.757839 | 5.60E-08 |
| 133 SERPINA3 | | 0.891897 | 1.29E-13 | 0.685914 | 2.79E-06 |
| 134 SLC1A5 | | 0.891533 | 1.37E-13 | 0.489457 | 0.002109 |
| 135 SHMT2 | | 0.891499 | 1.37E-13 | 0.485051 | 0.002344 |
| 136 PPARD | | 0.890917 | 1.50E-13 | 0.507247 | 0.001354 |
| 137 ING2 | | 0.890906 | 1.50E-13 | 0.594088 | 0.000106 |
| 138 RAMP1 | | 0.890374 | 1.63E-13 | 0.907823 | 9.05E-15 |
| 139 NPDC1 | | 0.889993 | 1.73E-13 | 0.807772 | 1.51E-09 |
| 140 REEP6 | | 0.889016 | 2.00E-13 | 0.634456 | 2.48E-05 |
| 141 CXCL14 | | 0.88813 | 2.28E-13 | 0.754843 | 6.76E-08 |
| 142 CBS | | 0.887599 | 2.47E-13 | 0.906496 | 1.15E-14 |
| 143 MYL5 | | 0.887264 | 2.59E-13 | 0.480369 | 0.00262 |
| 144 ANTXR2 | | 0.886944 | 2.72E-13 | 0.524383 | 0.000864 |
| 145 SURF6 | | 0.886831 | 2.76E-13 | 0.613265 | 5.44E-05 |
| 146 CPNE3 | | 0.886225 | 3.02E-13 | 0.545294 | 0.000483 |
| 147 HLX1 | | 0.886145 | 3.05E-13 | 0.913956 | 2.85E-15 |
| 148 GCAT | | 0.884758 | 3.73E-13 | 0.524908 | 0.000852 |
| 149 OXSM | | 0.884738 | 3.74E-13 | 0.678172 | 3.99E-06 |
| 150 CSEN | | 0.883976 | 4.17E-13 | 0.748219 | 1.02E-07 |
| 151 ARHGAP9 | | 0.88347 | 4.49E-13 | 0.680963 | 3.51E-06 |
| 152 TRIM41 | | 0.883422 | 4.52E-13 | 0.519198 | 0.000992 |
| 153 DIO2 | | 0.882593 | 5.08E-13 | 0.674239 | 4.76E-06 |
| 154 CLTCL1 | | 0.88248 | 5.16E-13 | 0.789831 | 6.19E-09 |
| 155 C22ORF16 | | 0.882306 | 5.29E-13 | 0.89907 | 4.12E-14 |
| 156 SDF2 | | 0.881763 | 5.70E-13 | 0.449991 | 0.005199 |
| 157 ARV1 | | 0.881751 | 5.71E-13 | 0.849399 | 3.01E-11 |
| 158 TUSC2 | | 0.881714 | 5.74E-13 | 0.629384 | 3.01E-05 |
| 159 PSARL | | 0.881477 | 5.94E-13 | 0.669564 | 5.85E-06 |
| 160 CDKL3 | | 0.881392 | 6.01E-13 | 0.818022 | 6.33E-10 |
| 161 ZNF79 | | 0.881277 | 6.10E-13 | 0.58485 | 0.000144 |
| 162 FUCA1 | | 0.881245 | 6.13E-13 | 0.579841 | 0.000169 |
| 163 VLDLR | | 0.881198 | 6.17E-13 | 0.744902 | 1.24E-07 |
| 164 PPP1R10 | | 0.881013 | 6.33E-13 | 0.797521 | 3.44E-09 |
| 165 ICMT | | 0.88023 | 7.06E-13 | 0.77232 | 2.16E-08 |
| 166 RRAGA | | 0.880225 | 7.06E-13 | 0.53562 | 0.000635 |
| 167 TM9SF1 | | 0.880063 | 7.22E-13 | 0.699112 | 1.48E-06 |
| 168 RASSF6 | | 0.87977 | 7.52E-13 | 0.761837 | 4.33E-08 |
| 169 DHX29 | | 0.879582 | 7.71E-13 | 0.727007 | 3.47E-07 |
| 170 MRPL50 | | 0.879002 | 8.35E-13 | 0.770615 | 2.42E-08 |
| 171 BTBD5 | | 0.878947 | 8.41E-13 | 0.851397 | 2.42E-11 |
| 172 MGC5509 | | 0.878563 | 8.87E-13 | 0.829286 | 2.28E-10 |
| 173 OSBPL2 | | 0.878249 | 9.25E-13 | 0.599874 | 8.71E-05 |
| 174 TMEM134 | | 0.878024 | 9.54E-13 | 0.912721 | 3.62E-15 |
| 175 MGC13024 | | 0.877954 | 9.63E-13 | 0.402875 | 0.013426 |
| 176 SMYD3 | | 0.877829 | 9.79E-13 | 0.603211 | 7.76E-05 |
| 177 PROSC | | 0.877711 | 9.95E-13 | 0.599789 | 8.73E-05 |
| 178 KHDRBS3 | | 0.877253 | 1.06E-12 | 0.849845 | 2.87E-11 |
| 179 PTK7 | | 0.876285 | 1.20E-12 | 0.57394 | 0.000204 |
| 180 AMMECR1 | | 0.876217 | 1.22E-12 | 0.61871 | 4.47E-05 |
| 181 PPM1E | | 0.87619 | 1.22E-12 | 0.657227 | 9.92E-06 |
| 182 HEXA | | 0.875637 | 1.31E-12 | 0.860201 | 8.95E-12 |
| 183 ZSWIM3 | | 0.87548 | 1.34E-12 | 0.778932 | 1.36E-08 |
| 184 C1ORF97 | | 0.875086 | 1.41E-12 | 0.462428 | 0.003956 |
| 185 FGFR1 | | 0.874395 | 1.55E-12 | 0.557697 | 0.000336 |
| 186 RP11-529I10.4 | | 0.874325 | 1.56E-12 | 0.845057 | 4.77E-11 |
| 187 CEECAM1 | | 0.874213 | 1.58E-12 | 0.8143 | 8.75E-10 |
| 188 RWDD2 | | 0.874126 | 1.60E-12 | 0.890408 | 1.62E-13 |
| 189 GARS | | 0.873912 | 1.65E-12 | 0.885518 | 3.34E-13 |
| 190 EIF2B4 | | 0.873597 | 1.72E-12 | 0.694898 | 1.82E-06 |
| 191 SELO | | 0.873495 | 1.74E-12 | 0.837827 | 9.97E-11 |
| 192 NUDT6 | | 0.873121 | 1.83E-12 | 0.88227 | 5.31E-13 |
| 193 NKIRAS2 | | 0.873004 | 1.85E-12 | 0.592248 | 0.000113 |
| 194 MGST2 | | 0.872394 | 2.01E-12 | 0.895206 | 7.71E-14 |
| 195 C3ORF31 | | 0.872321 | 2.02E-12 | 0.670206 | 5.69E-06 |
| 196 SERGEF | | 0.871923 | 2.13E-12 | 0.852159 | 2.23E-11 |
| 197 SLC35C1 | | 0.871605 | 2.22E-12 | 0.765017 | 3.52E-08 |
| 198 BCAS4 | | 0.87142 | 2.27E-12 | 0.776226 | 1.65E-08 |
| 199 COL15A1 | | 0.871364 | 2.29E-12 | 0.912893 | 3.50E-15 |
| 200 DNAPTP6 | | 0.871264 | 2.32E-12 | 0.810068 | 1.25E-09 |
| 201 PEX19 | | 0.871122 | 2.36E-12 | 0.899495 | 3.84E-14 |
| 202 CIAPIN1 | | 0.870163 | 2.67E-12 | 0.524887 | 0.000852 |
| 203 KIF1B | | 0.86931 | 2.97E-12 | 0.502633 | 0.001522 |
| 204 BRP44L | | 0.868235 | 3.40E-12 | 0.657421 | 9.84E-06 |
| 205 CFD | | 0.867803 | 3.58E-12 | 0.794322 | 4.41E-09 |
| 206 JOSD3 | | 0.867575 | 3.69E-12 | 0.805772 | 1.78E-09 |
| 207 MDP-1 | | 0.867512 | 3.72E-12 | 0.487891 | 0.00219 |
| 208 GPR108 | | 0.867187 | 3.87E-12 | 0.483729 | 0.00242 |
| 209 PNPLA2 | | 0.866844 | 4.03E-12 | 0.66416 | 7.39E-06 |
| 210 AIG1 | | 0.866269 | 4.33E-12 | 0.895564 | 7.28E-14 |
| 211 STIP1 | | 0.865675 | 4.66E-12 | 0.853151 | 2.00E-11 |
| 212 C1ORF53 | | 0.865353 | 4.84E-12 | 0.651285 | 1.27E-05 |
| 213 FLJ21749 | | 0.864184 | 5.58E-12 | 0.82309 | 4.03E-10 |
| 214 TMEM99 | | 0.864031 | 5.68E-12 | 0.660188 | 8.76E-06 |
| 215 G6PC3 | | 0.863149 | 6.32E-12 | 0.680662 | 3.56E-06 |
| 216 FLJ23584 | | 0.862597 | 6.75E-12 | 0.684116 | 3.04E-06 |
| 217 SPR | | 0.862542 | 6.79E-12 | 0.717402 | 5.84E-07 |
| 218 KIAA2013 | | 0.862482 | 6.84E-12 | 0.488956 | 0.002134 |
| 219 ZC3H10 | | 0.861757 | 7.45E-12 | 0.409991 | 0.01173 |
| 220 C9ORF9 | | 0.861472 | 7.71E-12 | 0.90146 | 2.76E-14 |
| 221 PCDHB10 | | 0.86091 | 8.24E-12 | 0.493427 | 0.001914 |
| 222 FOXL2 | | 0.860737 | 8.40E-12 | 0.763966 | 3.77E-08 |
| 223 TCTA | | 0.860552 | 8.59E-12 | 0.652141 | 1.23E-05 |
| 224 GEMIN6 | | 0.860442 | 8.70E-12 | 0.874844 | 1.46E-12 |
| 225 ZCCHC17 | | 0.860405 | 8.74E-12 | 0.886652 | 2.84E-13 |
| 226 DYRK4 | | 0.860185 | 8.97E-12 | 0.491311 | 0.002016 |
| 227 RBED1 | | 0.859925 | 9.24E-12 | 0.626963 | 3.29E-05 |
| 228 MIPEP | | 0.859683 | 9.51E-12 | 0.833497 | 1.52E-10 |
| 229 COQ9 | | 0.859428 | 9.79E-12 | 0.454573 | 0.004706 |
| 230 LOC129138 | | 0.859379 | 9.85E-12 | 0.478529 | 0.002736 |
| 231 C16ORF9 | | 0.859034 | 1.03E-11 | 0.456501 | 0.004512 |
| 232 C1ORF83 | | 0.858673 | 1.07E-11 | 0.773975 | 1.93E-08 |
| 233 PRPF4 | | 0.858543 | 1.08E-11 | 0.506779 | 0.00137 |
| 234 C21ORF33 | | 0.858025 | 1.15E-11 | 0.525667 | 0.000834 |
| 235 FAHD2B | | 0.857834 | 1.18E-11 | 0.858389 | 1.10E-11 |
| 236 ZDHHC24 | | 0.857809 | 1.18E-11 | 0.681147 | 3.48E-06 |
| 237 FAM86A | | 0.856961 | 1.30E-11 | 0.784456 | 9.19E-09 |
| 238 ZNF193 | | 0.856173 | 1.42E-11 | 0.672747 | 5.08E-06 |
| 239 NR2F2 | | 0.855784 | 1.49E-11 | 0.766874 | 3.11E-08 |
| 240 SLC30A7 | | 0.855624 | 1.51E-11 | 0.800644 | 2.69E-09 |
| 241 RNF185 | | 0.85499 | 1.63E-11 | 0.467553 | 0.003525 |
| 242 DNAJB2 | | 0.85496 | 1.63E-11 | 0.468855 | 0.003422 |
| 243 FAM82C | | 0.854905 | 1.64E-11 | 0.706628 | 1.02E-06 |
| 244 PIGZ | | 0.854683 | 1.68E-11 | 0.784183 | 9.38E-09 |
| 245 PEBP1 | | 0.854395 | 1.74E-11 | 0.616271 | 4.89E-05 |
| 246 SLC44A3 | | 0.854294 | 1.76E-11 | 0.631362 | 2.79E-05 |
| 247 HMGCL | | 0.854215 | 1.77E-11 | 0.696545 | 1.68E-06 |
| 248 LMO4 | | 0.854179 | 1.78E-11 | 0.497903 | 0.001714 |
| 249 SAA1 | | 0.854003 | 1.82E-11 | 0.84797 | 3.51E-11 |
| 250 ADAM23 | | 0.853101 | 2.01E-11 | 0.906555 | 1.14E-14 |
| 251 A2M | | 0.852687 | 2.10E-11 | 0.830775 | 1.98E-10 |
| 252 LONRF2 | | 0.852318 | 2.19E-11 | 0.73165 | 2.68E-07 |
| 253 ULBP1 | | 0.852146 | 2.23E-11 | 0.773221 | 2.03E-08 |
| 254 SMUG1 | | 0.851916 | 2.29E-11 | 0.755501 | 6.49E-08 |
| 255 BTD | | 0.851861 | 2.30E-11 | 0.474686 | 0.002993 |
| 256 DDR2 | | 0.851825 | 2.31E-11 | 0.629283 | 3.02E-05 |
| 257 ABLIM2 | | 0.851405 | 2.42E-11 | 0.816606 | 7.17E-10 |
| 258 CHST12 | | 0.851171 | 2.48E-11 | 0.839743 | 8.23E-11 |
| 259 ADPRHL1 | | 0.850993 | 2.53E-11 | 0.513314 | 0.001158 |
| 260 HK1 | | 0.850592 | 2.64E-11 | 0.859912 | 9.26E-12 |
| 261 UBPH | | 0.850413 | 2.70E-11 | 0.90763 | 9.37E-15 |
| 262 MTAP | | 0.850359 | 2.71E-11 | 0.8617 | 7.50E-12 |
| 263 LOC388135 | | 0.850106 | 2.79E-11 | 0.616524 | 4.84E-05 |
| 264 DOCK5 | | 0.850051 | 2.80E-11 | 0.724808 | 3.92E-07 |
| 265 ART3 | | 0.84991 | 2.85E-11 | 0.85 | 2.82E-11 |
| 266 TMED4 | | 0.849628 | 2.93E-11 | 0.659037 | 9.20E-06 |
| 267 PIP5K1C | | 0.849526 | 2.97E-11 | 0.414442 | 0.010764 |
| 268 TNPO2 | | 0.849115 | 3.10E-11 | 0.658625 | 9.36E-06 |
| 269 LRRC28 | | 0.848977 | 3.15E-11 | 0.861848 | 7.37E-12 |
| 270 VPS25 | | 0.848759 | 3.22E-11 | 0.634523 | 2.47E-05 |
| 271 MRPS28 | | 0.848749 | 3.23E-11 | 0.808854 | 1.38E-09 |
| 272 D2HGDH | | 0.848639 | 3.26E-11 | 0.533198 | 0.000679 |
| 273 IPO13 | | 0.848231 | 3.41E-11 | 0.62076 | 4.15E-05 |
| 274 NT5DC2 | | 0.847382 | 3.73E-11 | 0.767419 | 3.00E-08 |
| 275 PRMT5 | | 0.847243 | 3.79E-11 | 0.720659 | 4.91E-07 |
| 276 TM6SF1 | | 0.847007 | 3.88E-11 | 0.868763 | 3.18E-12 |
| 277 BET1L | | 0.846585 | 4.06E-11 | 0.638281 | 2.13E-05 |
| 278 KIF1C | | 0.846216 | 4.22E-11 | 0.831775 | 1.80E-10 |
| 279 CPE | | 0.846102 | 4.27E-11 | 0.878957 | 8.40E-13 |
| 280 SURF1 | | 0.846075 | 4.29E-11 | 0.414649 | 0.010721 |
| 281 PIGV | | 0.845997 | 4.32E-11 | 0.534127 | 0.000662 |
| 282 TMEM118 | | 0.845539 | 4.53E-11 | 0.724489 | 3.99E-07 |
| 283 FHL2 | | 0.844775 | 4.91E-11 | 0.86122 | 7.94E-12 |
| 284 TDP1 | | 0.844346 | 5.14E-11 | 0.707831 | 9.60E-07 |
| 285 C6ORF130 | | 0.844002 | 5.32E-11 | 0.531232 | 0.000717 |
| 286 IPP | | 0.843984 | 5.33E-11 | 0.770758 | 2.40E-08 |
| 287 C6ORF151 | | 0.843932 | 5.36E-11 | 0.81094 | 1.16E-09 |
| 288 FZD9 | | 0.843914 | 5.37E-11 | 0.535206 | 0.000642 |
| 289 MT1A | | 0.843785 | 5.45E-11 | 0.715846 | 6.34E-07 |
| 290 WDR12 | | 0.84248 | 6.23E-11 | 0.520943 | 0.000947 |
| 291 INVS | | 0.842161 | 6.44E-11 | 0.828774 | 2.39E-10 |
| 292 NAP1L3 | | 0.842118 | 6.47E-11 | 0.92194 | 5.53E-16 |
| 293 COASY | | 0.842114 | 6.47E-11 | 0.833022 | 1.60E-10 |
| 294 CRLF1 | | 0.841799 | 6.68E-11 | 0.818165 | 6.26E-10 |
| 295 CLK4 | | 0.841588 | 6.83E-11 | 0.632972 | 2.62E-05 |
| 296 ETV5 | | 0.841133 | 7.15E-11 | 0.645204 | 1.62E-05 |
| 297 GPR18 | | 0.841089 | 7.18E-11 | 0.797729 | 3.39E-09 |
| 298 SRM | | 0.841051 | 7.21E-11 | 0.769828 | 2.55E-08 |
| 299 MOCS1 | | 0.84081 | 7.39E-11 | 0.752362 | 7.89E-08 |
| 300 C7ORF25 | | 0.840476 | 7.64E-11 | 0.836174 | 1.17E-10 |
| 301 TTC5 | | 0.840102 | 7.94E-11 | 0.747589 | 1.06E-07 |
| 302 GALNT11 | | 0.839296 | 8.61E-11 | 0.85946 | 9.76E-12 |
| 303 SCRG1 | | 0.839233 | 8.66E-11 | 0.878319 | 9.16E-13 |
| 304 SLC6A8 | | 0.839071 | 8.81E-11 | 0.645289 | 1.62E-05 |
| 305 STARD7 | | 0.838895 | 8.96E-11 | 0.647919 | 1.46E-05 |
| 306 LARP6 | | 0.838114 | 9.69E-11 | 0.864144 | 5.60E-12 |
| 307 LSM10 | | 0.838068 | 9.73E-11 | 0.93967 | 7.03E-18 |
| 308 ENDOG | | 0.837567 | 1.02E-10 | 0.731469 | 2.71E-07 |
| 309 FLJ10661 | | 0.836811 | 1.10E-10 | 0.528961 | 0.000763 |
| 310 ABCD1 | | 0.836557 | 1.13E-10 | 0.789265 | 6.46E-09 |
| 311 METTL3 | | 0.836462 | 1.14E-10 | 0.856469 | 1.38E-11 |
| 312 METTL7B | | 0.836232 | 1.17E-10 | 0.634783 | 2.45E-05 |
| 313 ZNF529 | | 0.835885 | 1.21E-10 | 0.498115 | 0.001705 |
| 314 TRIM56 | | 0.834632 | 1.37E-10 | 0.614799 | 5.15E-05 |
| 315 COG5 | | 0.834312 | 1.41E-10 | 0.863871 | 5.79E-12 |
| 316 GGPS1 | | 0.834204 | 1.42E-10 | 0.865391 | 4.82E-12 |
| 317 LOC442578 | | 0.833349 | 1.55E-10 | 0.380877 | 0.020035 |
| 318 KATNAL2 | | 0.833243 | 1.56E-10 | 0.3916 | 0.016536 |
| 319 FLJ22555 | | 0.832457 | 1.69E-10 | 0.429602 | 0.007965 |
| 320 WFDC1 | | 0.83232 | 1.71E-10 | 0.876038 | 1.24E-12 |
| 321 EIF2S3 | | 0.831941 | 1.77E-10 | 0.545631 | 0.000478 |
| 322 C21ORF59 | | 0.831568 | 1.83E-10 | 0.705396 | 1.09E-06 |
| 323 ZMAT3 | | 0.831096 | 1.92E-10 | 0.630543 | 2.88E-05 |
| 324 ECD | | 0.831084 | 1.92E-10 | 0.634707 | 2.45E-05 |
| 325 NIFUN | | 0.830822 | 1.97E-10 | 0.615008 | 5.11E-05 |
| 326 PDLIM3 | | 0.830689 | 2.00E-10 | 0.846484 | 4.11E-11 |
| 327 CNOT4 | | 0.830449 | 2.04E-10 | 0.385806 | 0.018357 |
| 328 C3ORF58 | | 0.830062 | 2.12E-10 | 0.898275 | 4.70E-14 |
| 329 EPB41L5 | | 0.830021 | 2.13E-10 | 0.649609 | 1.36E-05 |
| 330 MAMDC1 | | 0.829626 | 2.21E-10 | 0.838398 | 9.42E-11 |
| 331 PLCD1 | | 0.829357 | 2.26E-10 | 0.378807 | 0.020777 |
| 332 PFDN6 | | 0.829188 | 2.30E-10 | 0.895847 | 6.96E-14 |
| 333 NSUN5 | | 0.829175 | 2.30E-10 | 0.646954 | 1.51E-05 |
| 334 MT2A | | 0.829151 | 2.31E-10 | 0.779963 | 1.27E-08 |
| 335 DPM3 | | 0.829137 | 2.31E-10 | 0.523234 | 0.000891 |
| 336 HSZFP36 | | 0.829008 | 2.34E-10 | 0.743815 | 1.33E-07 |
| 337 HOMER1 | | 0.828946 | 2.35E-10 | 0.668507 | 6.13E-06 |
| 338 C8ORF4 | | 0.82851 | 2.45E-10 | 0.635026 | 2.42E-05 |
| 339 LASS1 | | 0.828298 | 2.50E-10 | 0.761028 | 4.56E-08 |
| 340 DPM1 | | 0.828153 | 2.53E-10 | 0.599017 | 8.96E-05 |
| 341 LOC114984 | | 0.828073 | 2.55E-10 | 0.711961 | 7.76E-07 |
| 342 STOML1 | | 0.827228 | 2.76E-10 | 0.665151 | 7.09E-06 |
| 343 C9ORF37 | | 0.827176 | 2.77E-10 | 0.749635 | 9.33E-08 |
| 344 ZNF174 | | 0.827098 | 2.79E-10 | 0.558523 | 0.000327 |
| 345 K6HF | | 0.827087 | 2.80E-10 | 0.525472 | 0.000839 |
| 346 MGC45840 | | 0.826688 | 2.90E-10 | 0.539117 | 0.000576 |
| 347 HIST1H4H | | 0.826353 | 2.99E-10 | 0.51791 | 0.001026 |
| 348 MTO1 | | 0.826001 | 3.09E-10 | 0.776413 | 1.63E-08 |
| 349 OGFRL1 | | 0.825663 | 3.19E-10 | 0.564909 | 0.00027 |
| 350 NUDT9 | | 0.825592 | 3.21E-10 | 0.467614 | 0.00352 |
| 351 SPSB2 | | 0.824731 | 3.48E-10 | 0.33574 | 0.042202 |
| 352 LOC644096 | | 0.824585 | 3.52E-10 | 0.460407 | 0.004139 |
| 353 JMJD4 | | 0.824292 | 3.62E-10 | 0.436171 | 0.006961 |
| 354 FLJ11200 | | 0.823951 | 3.73E-10 | 0.77393 | 1.93E-08 |
| 355 GNRH1 | | 0.823493 | 3.89E-10 | 0.835452 | 1.26E-10 |
| 356 ITGAE | | 0.822638 | 4.20E-10 | 0.783589 | 9.79E-09 |
| 357 GUCA1B | | 0.822348 | 4.31E-10 | 0.486717 | 0.002253 |
| 358 CPT2 | | 0.822127 | 4.40E-10 | 0.544825 | 0.000489 |
| 359 POLRMT | | 0.821931 | 4.48E-10 | 0.699131 | 1.48E-06 |
| 360 MTE | | 0.821586 | 4.62E-10 | 0.597789 | 9.35E-05 |
| 361 FLJ39575 | | 0.82158 | 4.62E-10 | 0.655913 | 1.05E-05 |
| 362 DAG1 | | 0.821061 | 4.84E-10 | 0.774993 | 1.80E-08 |
| 363 FLJ12681 | | 0.821035 | 4.85E-10 | 0.733991 | 2.35E-07 |
| 364 EIF4EBP1 | | 0.820787 | 4.96E-10 | 0.524287 | 0.000866 |
| 365 C10ORF9 | | 0.820481 | 5.10E-10 | 0.861279 | 7.89E-12 |
| 366 RNF167 | | 0.820134 | 5.26E-10 | 0.743448 | 1.35E-07 |
| 367 LOC129530 | | 0.81976 | 5.43E-10 | 0.47794 | 0.002774 |
| 368 HIBADH | | 0.819307 | 5.66E-10 | 0.521761 | 0.000927 |
| 369 ARMC10 | | 0.81911 | 5.76E-10 | 0.838477 | 9.35E-11 |
| 370 ZNF696 | | 0.819 | 5.81E-10 | 0.542254 | 0.000527 |
| 371 WDR25 | | 0.818942 | 5.84E-10 | 0.531934 | 0.000703 |
| 372 BLCAP | | 0.816914 | 6.98E-10 | 0.858721 | 1.06E-11 |
| 373 QP-C | | 0.816672 | 7.13E-10 | 0.533697 | 0.00067 |
| 374 FLJ90013 | | 0.816313 | 7.35E-10 | 0.661883 | 8.15E-06 |
| 375 TNFRSF11B | | 0.816263 | 7.38E-10 | 0.736306 | 2.06E-07 |
| 376 AKR1A1 | | 0.816037 | 7.53E-10 | 0.774981 | 1.80E-08 |
| 377 MRPS36 | | 0.815604 | 7.82E-10 | 0.791296 | 5.55E-09 |
| 378 PAFAH2 | | 0.815506 | 7.88E-10 | 0.526748 | 0.00081 |
| 379 Selenoprotein 15 0.815315 | | | 8.02E-10 | 0.574997 | 0.000197 |
| 380 LEF1 0.815205 | | | 8.09E-10 | 0.564611 | 0.000272 |
| 381 SENP5 0.814025 | | | 8.96E-10 | 0.835342 | 1.27E-10 |
| 382 KRCC1 0.813984 | | | 8.99E-10 | 0.793425 | 4.72E-09 |
| 383 ENDOGL1 0.813931 | | | 9.03E-10 | 0.774412 | 1.87E-08 |
| 384 MAF1 0.813694 | | | 9.21E-10 | 0.654599 | 1.11E-05 |
| 385 SLC41A1 0.813507 | | | 9.36E-10 | 0.514194 | 0.001132 |
| 386 CCDC127 0.812751 | | | 9.98E-10 | 0.410135 | 0.011697 |
| 387 PIGG 0.812735 | | | 1.00E-09 | 0.648141 | 1.44E-05 |
| 388 MLF2 0.812383 | | | 1.03E-09 | 0.713489 | 7.17E-07 |
| 389 DIRC2 0.812368 | | | 1.03E-09 | 0.687411 | 2.60E-06 |
| 390 ZNF613 0.812167 | | | 1.05E-09 | 0.613828 | 5.34E-05 |
| 391 MGC2747 0.812125 | | | 1.05E-09 | 0.531483 | 0.000712 |
| 392 CCT7 0.812002 | | | 1.06E-09 | 0.746651 | 1.12E-07 |
| 393 CYP46A1 0.811292 | | | 1.13E-09 | 0.558258 | 0.00033 |
| 394 BTRC 0.810885 | | | 1.17E-09 | 0.814976 | 8.25E-10 |
| 395 RHBDD1 0.81049 | | | 1.21E-09 | 0.6892 | 2.39E-06 |
| 396 MICA 0.810434 | | | 1.21E-09 | 0.653638 | 1.15E-05 |
| 397 C6ORF157 0.810322 | | | 1.23E-09 | 0.530567 | 0.00073 |
| 398 NOL3 0.810129 | | | 1.25E-09 | 0.622481 | 3.89E-05 |
| 399 LRAP | | 0.809929 | 1.27E-09 | 0.60191 | 8.12E-05 |
| 400 LINS1 | | 0.809213 | 1.34E-09 | 0.633609 | 2.56E-05 |
| 401 NTF3 | | 0.808988 | 1.37E-09 | 0.903603 | 1.91E-14 |
| 402 CEBPG | | 0.808941 | 1.37E-09 | 0.546125 | 0.000471 |
| 403 SENP2 | | 0.808457 | 1.43E-09 | 0.60673 | 6.86E-05 |
| 404 MGC59937 | | 0.808216 | 1.46E-09 | 0.66868 | 6.08E-06 |
| 405 SATB2 | | 0.807881 | 1.50E-09 | 0.567539 | 0.000249 |
| 406 GOT1 | | 0.807869 | 1.50E-09 | 0.520591 | 0.000956 |
| 407 CRIPAK | | 0.807686 | 1.53E-09 | 0.686631 | 2.70E-06 |
| 408 FLJ22167 | | 0.807154 | 1.59E-09 | 0.813249 | 9.57E-10 |
| 409 ZNF514 | | 0.806304 | 1.71E-09 | 0.710701 | 8.29E-07 |
| 410 MXD1 | | 0.805194 | 1.87E-09 | 0.471833 | 0.003196 |
| 411 LOC128977 | | 0.804597 | 1.96E-09 | 0.376411 | 0.021664 |
| 412 RAB24 | | 0.803979 | 2.06E-09 | 0.494595 | 0.00186 |
| 413 PLTP | | 0.802672 | 2.29E-09 | 0.3984 | 0.014596 |
| 414 LAMB2 | | 0.801755 | 2.47E-09 | 0.758638 | 5.32E-08 |
| 415 MAN2B1 | | 0.80169 | 2.48E-09 | 0.702767 | 1.24E-06 |
| 416 MPZL1 | | 0.801187 | 2.58E-09 | 0.550122 | 0.00042 |
| 417 SYT7 | | 0.800706 | 2.68E-09 | 0.639353 | 2.05E-05 |
| 418 TRAP1 | | 0.800606 | 2.70E-09 | 0.769916 | 2.54E-08 |
| 419 CACNA2D4 | | 0.800114 | 2.81E-09 | 0.777368 | 1.52E-08 |
| 420 LOC402682 | | 0.799349 | 2.98E-09 | 0.489362 | 0.002113 |
| 421 LOC200312 | | 0.79877 | 3.12E-09 | 0.796562 | 3.71E-09 |
| 422 ZNF493 | | 0.798057 | 3.30E-09 | 0.740837 | 1.58E-07 |
| 423 SNX19 | | 0.797809 | 3.37E-09 | 0.355054 | 0.031053 |
| 424 HIBCH | | 0.797789 | 3.37E-09 | 0.77862 | 1.39E-08 |
| 425 RPAP1 | | 0.797174 | 3.54E-09 | 0.460331 | 0.004146 |
| 426 TAF10 | | 0.79659 | 3.70E-09 | 0.403369 | 0.013302 |
| 427 VPS35 | | 0.796528 | 3.72E-09 | 0.824256 | 3.63E-10 |
| 428 TCEA2 | | 0.795995 | 3.88E-09 | 0.71997 | 5.09E-07 |
| 429 C20ORF30 | | 0.794474 | 4.36E-09 | 0.650468 | 1.31E-05 |
| 430 LRP10 | | 0.794434 | 4.37E-09 | 0.726778 | 3.52E-07 |
| 431 TMEM116 | | 0.794099 | 4.48E-09 | 0.40126 | 0.013839 |
| 432 STXBP1 | | 0.793739 | 4.61E-09 | 0.816359 | 7.32E-10 |
| 433 SLC22A17 | | 0.793611 | 4.65E-09 | 0.385445 | 0.018475 |
| 434 TMEM107 | | 0.792922 | 4.90E-09 | 0.689664 | 2.34E-06 |
| 435 TTC9C | | 0.792813 | 4.94E-09 | 0.845168 | 4.71E-11 |
| 436 JOSD2 | | 0.792619 | 5.02E-09 | 0.664772 | 7.20E-06 |
| 437 ALDH1A3 | | 0.792562 | 5.04E-09 | 0.590582 | 0.000119 |
| 438 FLJ11806 | | 0.792298 | 5.14E-09 | 0.343228 | 0.037547 |
| 439 LCMT2 | | 0.791936 | 5.28E-09 | 0.381729 | 0.019736 |
| 440 ZNF558 | | 0.791901 | 5.30E-09 | 0.607909 | 6.59E-05 |
| 441 FLJ20160 | | 0.791331 | 5.53E-09 | 0.803629 | 2.12E-09 |
| 442 TPK1 | | 0.791312 | 5.54E-09 | 0.817941 | 6.38E-10 |
| 443 HHLA3 | | 0.791045 | 5.65E-09 | 0.670761 | 5.55E-06 |
| 444 UBL7 | | 0.791018 | 5.66E-09 | 0.360005 | 0.028623 |
| 445 DIRAS3 | | 0.790783 | 5.76E-09 | 0.689595 | 2.35E-06 |
| 446 KCNG1 | | 0.790755 | 5.78E-09 | 0.759061 | 5.18E-08 |
| 447 RSAD1 | | 0.790585 | 5.85E-09 | 0.397728 | 0.014779 |
| 448 ELOF1 | | 0.790489 | 5.89E-09 | 0.461439 | 0.004045 |
| 449 WDR68 | | 0.789981 | 6.12E-09 | 0.410639 | 0.011585 |
| 450 SLC35A2 | | 0.789917 | 6.15E-09 | 0.41847 | 0.009949 |
| 451 ITIH3 | | 0.789882 | 6.17E-09 | 0.417492 | 0.010142 |
| 452 NEK1 | | 0.788908 | 6.63E-09 | 0.808087 | 1.48E-09 |
| 453 C1ORF66 | | 0.788894 | 6.64E-09 | 0.347728 | 0.034957 |
| 454 CCL13 | | 0.788531 | 6.82E-09 | 0.684557 | 2.98E-06 |
| 455 NR2F1 | | 0.788139 | 7.02E-09 | 0.326751 | 0.048395 |
| 456 C6ORF148 | | 0.788014 | 7.08E-09 | 0.422346 | 0.009215 |
| 457 C8ORF41 | | 0.787461 | 7.38E-09 | 0.659822 | 8.90E-06 |
| 458 ABHD14B | | 0.787041 | 7.61E-09 | 0.410228 | 0.011677 |
| 459 PARL | | 0.78694 | 7.67E-09 | 0.716616 | 6.09E-07 |
| 460 NGRN | | 0.786233 | 8.07E-09 | 0.847392 | 3.73E-11 |
| 461 NARFL | | 0.786132 | 8.14E-09 | 0.541198 | 0.000543 |
| 462 CCDC51 | | 0.785836 | 8.31E-09 | 0.750767 | 8.70E-08 |
| 463 ZFYVE21 | | 0.785629 | 8.44E-09 | 0.624093 | 3.67E-05 |
| 464 MPI | | 0.78546 | 8.54E-09 | 0.842723 | 6.08E-11 |
| 465 MRPS10 | | 0.785351 | 8.61E-09 | 0.687031 | 2.65E-06 |
| 466 TRAPPC2 | | 0.785342 | 8.62E-09 | 0.597554 | 9.42E-05 |
| 467 MGC61571 | | 0.785316 | 8.63E-09 | 0.756716 | 6.01E-08 |
| 468 COIL | | 0.785247 | 8.68E-09 | 0.533151 | 0.00068 |
| 469 C20ORF24 | | 0.78514 | 8.75E-09 | 0.748341 | 1.01E-07 |
| 470 PEG3 | | 0.785126 | 8.75E-09 | 0.689339 | 2.38E-06 |
| 471 PABPC1 | | 0.784782 | 8.98E-09 | 0.638245 | 2.14E-05 |
| 472 ABI2 | | 0.784217 | 9.35E-09 | 0.400256 | 0.014101 |
| 473 ATP6V1G1 | | 0.784068 | 9.45E-09 | 0.580971 | 0.000163 |
| 474 LOC119710 | | 0.784055 | 9.46E-09 | 0.60469 | 7.37E-05 |
| 475 MTCP1 | | 0.783868 | 9.59E-09 | 0.366763 | 0.025561 |
| 476 PFN4 | | 0.783434 | 9.90E-09 | 0.498378 | 0.001694 |
| 477 IFNA8 | | 0.783424 | 9.90E-09 | 0.401358 | 0.013813 |
| 478 RGMB | | 0.783366 | 9.95E-09 | 0.835599 | 1.24E-10 |
| 479 ADAMTS1 | | 0.782994 | 1.02E-08 | 0.421157 | 0.009435 |
| 480 TRMT12 | | 0.782969 | 1.02E-08 | 0.576103 | 0.000191 |
| 481 PKD2 | | 0.782824 | 1.03E-08 | 0.663001 | 7.77E-06 |
| 482 TTC23 | | 0.782339 | 1.07E-08 | 0.476976 | 0.002837 |
| 483 ARMC7 | | 0.781667 | 1.12E-08 | 0.403054 | 0.013381 |
| 484 ANGEL1 | | 0.781488 | 1.14E-08 | 0.76149 | 4.43E-08 |
| 485 ZNF306 | | 0.78104 | 1.18E-08 | 0.540703 | 0.00055 |
| 486 TRIB3 | | 0.780123 | 1.25E-08 | 0.504702 | 0.001445 |
| 487 PNN | | 0.779759 | 1.29E-08 | 0.519875 | 0.000974 |
| 488 NKRF | | 0.778903 | 1.37E-08 | 0.586644 | 0.000136 |
| 489 WDR7 | | 0.77869 | 1.39E-08 | 0.630977 | 2.83E-05 |
| 490 ATP5G1 | | 0.778569 | 1.40E-08 | 0.857929 | 1.16E-11 |
| 491 PRR4 | | 0.778527 | 1.40E-08 | 0.719557 | 5.21E-07 |
| 492 ALAS1 | | 0.777855 | 1.47E-08 | 0.574609 | 0.0002 |
| 493 DNTTIP1 | | 0.776874 | 1.58E-08 | 0.650091 | 1.33E-05 |
| 494 MORG1 | | 0.776844 | 1.58E-08 | 0.523562 | 0.000883 |
| 495 ZNF132 | | 0.776614 | 1.61E-08 | 0.561676 | 0.000298 |
| 496 POLR1C | | 0.776084 | 1.67E-08 | 0.357282 | 0.02994 |
| 497 LOC116143 | | 0.775489 | 1.74E-08 | 0.653955 | 1.14E-05 |
| 498 ASAHL | | 0.775352 | 1.75E-08 | 0.599199 | 8.91E-05 |
| 499 NOL8 | | 0.774489 | 1.86E-08 | 0.444332 | 0.005867 |
| 500 SERINC1 | | 0.774203 | 1.90E-08 | 0.788672 | 6.75E-09 |
| 501 TK2 | | 0.77414 | 1.91E-08 | 0.368658 | 0.024753 |
| 502 SETD8 | | 0.773627 | 1.97E-08 | 0.766831 | 3.12E-08 |
| 503 ZDHHC14 | | 0.773506 | 1.99E-08 | 0.661847 | 8.16E-06 |
| 504 CAMK1 | | 0.773436 | 2.00E-08 | 0.716457 | 6.14E-07 |
| 505 DDX55 | | 0.773324 | 2.01E-08 | 0.384013 | 0.018953 |
| 506 CHST1 | | 0.773119 | 2.04E-08 | 0.652418 | 1.21E-05 |
| 507 KCR1 | | 0.77257 | 2.12E-08 | 0.525244 | 0.000844 |
| 508 CNIH | | 0.772174 | 2.18E-08 | 0.721345 | 4.73E-07 |
| 509 ANAPC10 | | 0.771106 | 2.34E-08 | 0.668346 | 6.17E-06 |
| 510 EXTL2 | | 0.770465 | 2.45E-08 | 0.520293 | 0.000964 |
| 511 STS | | 0.769795 | 2.56E-08 | 0.550194 | 0.000419 |
| 512 SPPL2A | | 0.769101 | 2.68E-08 | 0.811129 | 1.15E-09 |
| 513 C9ORF5 | | 0.768972 | 2.71E-08 | 0.673374 | 4.94E-06 |
| 514 PTPDC1 | | 0.768567 | 2.78E-08 | 0.61177 | 5.74E-05 |
| 515 HOXA6 | | 0.767786 | 2.93E-08 | 0.718896 | 5.39E-07 |
| 516 ARHGAP1 | | 0.767557 | 2.97E-08 | 0.59954 | 8.81E-05 |
| 517 C15ORF17 | | 0.767469 | 2.99E-08 | 0.587061 | 0.000134 |
| 518 CTSH | | 0.767451 | 3.00E-08 | 0.6661 | 6.80E-06 |
| 519 FKSG24 | | 0.767346 | 3.02E-08 | 0.431715 | 0.007629 |
| 520 ADARB1 | | 0.76734 | 3.02E-08 | 0.734652 | 2.26E-07 |
| 521 AP2S1 | | 0.767023 | 3.08E-08 | 0.593906 | 0.000107 |
| 522 TMCO1 | | 0.766867 | 3.11E-08 | 0.717839 | 5.70E-07 |
| 523 TBC1D9B | | 0.766564 | 3.18E-08 | 0.618692 | 4.47E-05 |
| 524 WARS | | 0.763917 | 3.78E-08 | 0.398354 | 0.014608 |
| 525 RP1-112K5.2 | | 0.763323 | 3.93E-08 | 0.578037 | 0.000179 |
| 526 C17ORF81 | | 0.763288 | 3.94E-08 | 0.58439 | 0.000146 |
| 527 D4S234E | | 0.762977 | 4.02E-08 | 0.393472 | 0.015982 |
| 528 ATP6V0A1 | | 0.761721 | 4.36E-08 | 0.508471 | 0.001312 |
| 529 IARS | | 0.760596 | 4.69E-08 | 0.676212 | 4.36E-06 |
| 530 YARS | | 0.760594 | 4.69E-08 | 0.641186 | 1.90E-05 |
| 531 TARS | | 0.760567 | 4.70E-08 | 0.626824 | 3.31E-05 |
| 532 BPY2IP1 | | 0.760003 | 4.87E-08 | 0.557318 | 0.000339 |
| 533 EPHB2 | | 0.759576 | 5.01E-08 | 0.396394 | 0.015147 |
| 534 GABRR1 | | 0.759341 | 5.09E-08 | 0.856562 | 1.36E-11 |
| 535 LGTN | | 0.758383 | 5.41E-08 | 0.602845 | 7.86E-05 |
| 536 ZFYVE27 | | 0.758288 | 5.44E-08 | 0.579032 | 0.000174 |
| 537 ZDHHC11 | | 0.757394 | 5.76E-08 | 0.781017 | 1.18E-08 |
| 538 LOC283377 | | 0.756762 | 5.99E-08 | 0.592145 | 0.000113 |
| 539 C12ORF60 | | 0.755794 | 6.37E-08 | 0.507219 | 0.001355 |
| 540 KLF8 | | 0.755637 | 6.43E-08 | 0.435431 | 0.007069 |
| 541 BCS1L | | 0.755527 | 6.48E-08 | 0.483415 | 0.002438 |
| 542 ZCRB1 | | 0.755029 | 6.68E-08 | 0.633758 | 2.54E-05 |
| 543 C14ORF92 | | 0.754223 | 7.03E-08 | 0.514841 | 0.001113 |
| 544 SESN1 | | 0.754034 | 7.11E-08 | 0.344766 | 0.036644 |
| 545 SCAMP4 | | 0.753469 | 7.36E-08 | 0.634234 | 2.50E-05 |
| 546 HSPA9B | | 0.753066 | 7.55E-08 | 0.731121 | 2.76E-07 |
| 547 PLAC9 | | 0.75296 | 7.60E-08 | 0.702005 | 1.29E-06 |
| 548 MRP63 | | 0.75223 | 7.95E-08 | 0.360081 | 0.028587 |
| 549 COLEC10 | | 0.751317 | 8.41E-08 | 0.425035 | 0.008734 |
| 550 KIAA0961 | | 0.751092 | 8.53E-08 | 0.364636 | 0.026494 |
| 551 FLJ22639 | | 0.750741 | 8.72E-08 | 0.760973 | 4.58E-08 |
| 552 PRDM13 | | 0.750678 | 8.75E-08 | 0.497283 | 0.00174 |
| 553 TXNDC14 | | 0.750517 | 8.84E-08 | 0.709327 | 8.89E-07 |
| 554 TIGD6 | | 0.750336 | 8.94E-08 | 0.561071 | 0.000303 |
| 555 TALDO1 | | 0.747566 | 1.06E-07 | 0.710571 | 8.34E-07 |
| 556 RAB21 | | 0.747003 | 1.09E-07 | 0.765855 | 3.33E-08 |
| 557 LRRC23 | | 0.746734 | 1.11E-07 | 0.582501 | 0.000155 |
| 558 C14ORF142 | | 0.746521 | 1.13E-07 | 0.607007 | 6.80E-05 |
| 559 PPP1R3D | | 0.745114 | 1.23E-07 | 0.672643 | 5.11E-06 |
| 560 CENTB1 | | 0.743834 | 1.32E-07 | 0.8322 | 1.73E-10 |
| 561 DPM2 | | 0.743665 | 1.34E-07 | 0.350981 | 0.033177 |
| 562 GPNMB | | 0.743382 | 1.36E-07 | 0.660151 | 8.77E-06 |
| 563 NDUFV3 | | 0.742317 | 1.45E-07 | 0.481147 | 0.002573 |
| 564 ARD1A | | 0.74224 | 1.45E-07 | 0.648676 | 1.41E-05 |
| 565 PDP2 | | 0.74173 | 1.50E-07 | 0.536941 | 0.000612 |
| 566 RAB18 | | 0.741599 | 1.51E-07 | 0.796079 | 3.85E-09 |
| 567 CRBN | | 0.740887 | 1.58E-07 | 0.461453 | 0.004043 |
| 568 ABCA2 | | 0.740559 | 1.61E-07 | 0.702738 | 1.24E-06 |
| 569 AHCY | | 0.740342 | 1.63E-07 | 0.425435 | 0.008664 |
| 570 TMEM109 | | 0.739832 | 1.68E-07 | 0.586553 | 0.000136 |
| 571 FLJ21103 | | 0.738645 | 1.80E-07 | 0.425304 | 0.008687 |
| 572 DNM3 | | 0.738395 | 1.82E-07 | 0.717631 | 5.77E-07 |
| 573 PIGX | | 0.737607 | 1.91E-07 | 0.492282 | 0.001968 |
| 574 RNF123 | | 0.736358 | 2.05E-07 | 0.591516 | 0.000115 |
| 575 ELP4 | | 0.735164 | 2.20E-07 | 0.752259 | 7.94E-08 |
| 576 SLA/LP | | 0.735092 | 2.20E-07 | 0.549985 | 0.000421 |
| 577 CYHR1 | | 0.734673 | 2.26E-07 | 0.382498 | 0.019469 |
| 578 FLJ21816 | | 0.73311 | 2.47E-07 | 0.421606 | 0.009352 |
| 579 DGCR6 | | 0.732656 | 2.53E-07 | 0.473309 | 0.003089 |
| 580 PSENEN | | 0.73171 | 2.67E-07 | 0.588331 | 0.000128 |
| 581 CCDC6 | | 0.731014 | 2.78E-07 | 0.64637 | 1.55E-05 |
| 582 LYAR | | 0.729333 | 3.05E-07 | 0.634366 | 2.49E-05 |
| 583 DSG4 | | 0.728625 | 3.17E-07 | 0.55604 | 0.000353 |
| 584 LOC654017 | | 0.72789 | 3.31E-07 | 0.665651 | 6.94E-06 |
| 585 GYS1 | | 0.727881 | 3.31E-07 | 0.587145 | 0.000133 |
| 586 CDIPT | | 0.726884 | 3.50E-07 | 0.640994 | 1.92E-05 |
| 587 DHRS7 | | 0.725528 | 3.77E-07 | 0.458403 | 0.004327 |
| 588 CRADD | | 0.724112 | 4.07E-07 | 0.649804 | 1.35E-05 |
| 589 C20ORF134 | | 0.724 | 4.10E-07 | 0.344301 | 0.036915 |
| 590 SEC61A2 | | 0.72287 | 4.35E-07 | 0.544176 | 0.000498 |
| 591 DNAJC5 | | 0.721559 | 4.67E-07 | 0.694602 | 1.85E-06 |
| 592 ATG16L1 | | 0.720082 | 5.06E-07 | 0.553607 | 0.000379 |
| 593 VPREB3 | | 0.719269 | 5.29E-07 | 0.777997 | 1.46E-08 |
| 594 CYP2U1 | | 0.718995 | 5.36E-07 | 0.631691 | 2.75E-05 |
| 595 OTUD5 | | 0.71884 | 5.41E-07 | 0.611105 | 5.88E-05 |
| 596 KIAA0649 | | 0.718504 | 5.51E-07 | 0.760383 | 4.76E-08 |
| 597 TNFSF13B | | 0.716479 | 6.13E-07 | 0.694791 | 1.83E-06 |
| 598 FLJ32771 | | 0.71567 | 6.40E-07 | 0.520009 | 0.000971 |
| 599 VPS8 | | 0.71483 | 6.69E-07 | 0.544959 | 0.000487 |
| 600 PRDM2 | | 0.712676 | 7.48E-07 | 0.40567 | 0.012737 |
| 601 PTDSS1 | | 0.712454 | 7.57E-07 | 0.372495 | 0.023182 |
| 602 CHP | | 0.711667 | 7.88E-07 | 0.49623 | 0.001786 |
| 603 TMED10P | | 0.709377 | 8.87E-07 | 0.599416 | 8.84E-05 |
| 604 FTO | | 0.709294 | 8.91E-07 | 0.577904 | 0.00018 |
| 605 C21ORF127 | | 0.709124 | 8.99E-07 | 0.532633 | 0.00069 |
| 606 IGF2R | | 0.708991 | 9.05E-07 | 0.342731 | 0.037842 |
| 607 ZNF447 | | 0.707001 | 1.00E-06 | 0.325198 | 0.049535 |
| 608 TOR1A | | 0.706786 | 1.01E-06 | 0.466097 | 0.003643 |
| 609 IMMT | | 0.706451 | 1.03E-06 | 0.583479 | 0.00015 |
| 610 CBX7 | | 0.706251 | 1.04E-06 | 0.646775 | 1.52E-05 |
| 611 MAPK8IP1 | | 0.705077 | 1.10E-06 | 0.336385 | 0.041783 |
| 612 BOLA1 | | 0.703793 | 1.18E-06 | 0.525102 | 0.000847 |
| 613 SLC23A2 | | 0.702363 | 1.26E-06 | 0.474509 | 0.003005 |
| 614 EIF2C3 | | 0.699953 | 1.42E-06 | 0.446856 | 0.00556 |
| 615 HIST1H2BK | | 0.69969 | 1.44E-06 | 0.438482 | 0.006635 |
| 616 MRPL51 | | 0.698558 | 1.53E-06 | 0.62065 | 4.16E-05 |
| 617 PSMA2 | | 0.69622 | 1.71E-06 | 0.58401 | 0.000148 |
| 618 PANK4 | | 0.696087 | 1.72E-06 | 0.560864 | 0.000305 |
| 619 C12ORF62 | | 0.695539 | 1.77E-06 | 0.441645 | 0.00621 |
| 620 PLXNA1 | | 0.695464 | 1.77E-06 | 0.610609 | 5.99E-05 |
| 621 SCDR10 | | 0.69488 | 1.82E-06 | 0.50698 | 0.001363 |
| 622 ZFAND3 | | 0.694537 | 1.86E-06 | 0.514466 | 0.001124 |
| 623 BAK1 | | 0.693943 | 1.91E-06 | 0.565217 | 0.000267 |
| 624 PHLDB3 | | 0.691421 | 2.15E-06 | 0.61494 | 5.13E-05 |
| 625 MRPS33 | | 0.690341 | 2.27E-06 | 0.477054 | 0.002832 |
| 626 LOC402055 | | 0.690238 | 2.28E-06 | 0.512899 | 0.001171 |
| 627 ADCY9 | | 0.688935 | 2.42E-06 | 0.414602 | 0.010731 |
| 628 ERGIC1 | | 0.688842 | 2.43E-06 | 0.44967 | 0.005235 |
| 629 C9ORF66 | | 0.687774 | 2.56E-06 | 0.533583 | 0.000672 |
| 630 COL4A3BP | | 0.684395 | 3.00E-06 | 0.353495 | 0.031852 |
| 631 EGFLAM | | 0.681173 | 3.48E-06 | 0.520645 | 0.000955 |
| 632 DHX36 | | 0.679833 | 3.70E-06 | 0.435257 | 0.007094 |
| 633 C18ORF17 | | 0.676527 | 4.29E-06 | 0.352807 | 0.03221 |
| 634 SLC39A9 | | 0.67493 | 4.61E-06 | 0.442077 | 0.006154 |
| 635 SMARCD3 | | 0.673939 | 4.82E-06 | 0.521676 | 0.000929 |
| 636 FLJ25143 | | 0.673532 | 4.91E-06 | 0.426821 | 0.008426 |
| 637 LHFPL1 | | 0.667325 | 6.45E-06 | 0.470226 | 0.003316 |
| 638 RIBC1 | | 0.6667 | 6.63E-06 | 0.62202 | 3.96E-05 |
| 639 ZNF222 | | 0.665091 | 7.10E-06 | 0.409632 | 0.011811 |
| 640 DISP1 | | 0.664753 | 7.21E-06 | 0.429222 | 0.008027 |
| 641 PARP2 | | 0.664529 | 7.28E-06 | 0.625375 | 3.50E-05 |
| 642 KLHDC8B | | 0.661711 | 8.21E-06 | 0.626922 | 3.30E-05 |
| 643 GREM1 | | 0.661209 | 8.39E-06 | 0.516557 | 0.001064 |
| 644 LRRC46 | | 0.658849 | 9.27E-06 | 0.422325 | 0.009219 |
| 645 SURF2 | | 0.65864 | 9.35E-06 | 0.508944 | 0.001297 |
| 646 GADD45GIP1 | | 0.655953 | 1.05E-05 | 0.537879 | 0.000596 |
| 647 ABTB2 | | 0.653751 | 1.15E-05 | 0.381686 | 0.019751 |
| 648 CETP | | 0.653138 | 1.18E-05 | 0.551264 | 0.000406 |
| 649 SAMM50 | | 0.652538 | 1.21E-05 | 0.592145 0.000113 | |
| 650 C17ORF48 | | 0.648573 | 1.42E-05 | 0.352604 0.032317 | |
| 651 NRBP1 | | 0.646994 | 1.51E-05 | 0.374711 0.022312 | |
| 652 CLEC4A | | 0.640584 | 1.95E-05 | 0.382826 0.019357 | |
| 653 DCUN1D2 | | 0.640083 | 1.99E-05 | 0.340298 0.039316 | |
| 654 ABHD6 | | 0.638895 | 2.08E-05 | 0.433848 0.007303 | |
| 655 USH3A | | 0.638438 | 2.12E-05 | 0.510407 0.001249 | |
| 656 GTPBP2 | | 0.638329 | 2.13E-05 | 0.467851 0.003501 | |
| 657 DERL3 | | 0.637932 | 2.16E-05 | 0.480697 0.0026 | |
| 658 H2BFS | | 0.637299 | 2.22E-05 | 0.417307 0.010179 | |
| 659 HIG2 | | 0.635373 | 2.39E-05 | 0.439095 0.006551 | |
| 660 MIA3 | | 0.63434 | 2.49E-05 | 0.472938 0.003116 | |
| 661 PDDC1 | | 0.632957 | 2.62E-05 | 0.593208 0.000109 | |
| 662 C19ORF23 | | 0.62975 | 2.97E-05 | 0.507205 0.001356 | |
| 663 NAT9 | | 0.629555 | 2.99E-05 | 0.492182 0.001973 | |
| 664 PECAM1 | | 0.625499 | 3.48E-05 | 0.433627 0.007336 | |
| 665 ZNF182 | | 0.62069 | 4.16E-05 | 0.481846 0.00253 | |
| 666 GALNT2 | | 0.618518 | 4.50E-05 | 0.52397 0.000873 | |
| 667 ZNF334 | | 0.613984 | 5.31E-05 | 0.38198 0.019649 | |
| 668 AAAS | | 0.61004 | 6.11E-05 | 0.481167 0.002571 | |
| 669 TTC18 | | 0.609474 | 6.23E-05 | 0.397226 0.014916 | |
| 670 CHMP7 | | 0.607732 | 6.63E-05 | 0.508801 0.001301 | |
| 671 BXDC5 | | 0.607032 | 6.79E-05 | 0.467875 0.003499 | |
| 672 ZNF42 | | 0.60313 | 7.78E-05 | 0.375311 0.022081 | |
| 673 P2RX2 | | 0.598677 | 9.07E-05 | 0.57341 0.000207 | |
| 674 PEX10 | | 0.596391 | 9.80E-05 | 0.469406 0.003379 | |
| 675 NPR1 | | 0.595111 0.000102 | | 0.551476 0.000403 | |
| 676 MRPL16 | | 0.594436 0.000105 | | 0.476707 0.002855 | |
| 677 TMEM25 | | 0.59323 0.000109 | | 0.484439 0.002379 | |
| 678 IMP5 | | 0.590252 0.00012 | | 0.46152 0.004037 | |
| 679 POLG2 | | 0.585632 0.00014 | | 0.494963 0.001843 | |
| 680 MRPL30 | | 0.583378 0.000151 | | 0.50067 0.001599 | |
| 681 LSDP5 | | 0.58251 0.000155 | | 0.573266 0.000208 | |
| 682 ARFRP1 | | 0.579541 0.000171 | | 0.540355 0.000556 | |
| 683 RGL3 | | 0.578082 0.000179 | | 0.468132 0.003479 | |
| 684 CFTR | | 0.575573 0.000194 | | 0.352725 0.032253 | |
| 685 BRWD3 | | 0.57504 0.000197 | | 0.388934 0.017354 | |
| 686 MRPL42P5 | | 0.572662 0.000212 | | 0.391539 0.016555 | |
| 687 TIMM17B | | 0.5723 0.000215 | | 0.540958 0.000546 | |
| 688 HIST1H3C | | 0.570779 0.000225 | | 0.51915 0.000993 | |
| 689 C8ORF44 | | 0.568158 0.000244 | | 0.527172 0.000801 | |
| 690 SLC2A11 | | 0.566913 0.000254 | | 0.411945 0.011297 | |
| 691 SH3BGR | | 0.566884 0.000254 | | 0.334201 0.043214 | |
| 692 ZNF607 | | 0.566804 0.000255 | | 0.349103 0.034195 | |
| 693 C9ORF98 | | 0.565492 0.000265 | | 0.517652 0.001033 | |
| 694 YWHAE | | 0.563643 0.000281 | | 0.532924 0.000684 | |
| 695 CRYGS | | 0.561783 0.000297 | | 0.469645 0.003361 | |
| 696 WT1 | | 0.557381 0.000339 | | 0.381768 0.019722 | |
| 697 MST150 | | 0.543758 0.000504 | | 0.420528 0.009554 | |
| 698 ZNF233 | | 0.542859 0.000518 | | 0.490688 0.002046 | |
| 699 LGI4 | | 0.537297 0.000606 0.40816 0.012148 | | | |
| 700 RNF43 | | 0.532575 0.000691 0.545585 0.000479 | | | |
| 701 TMEM127 | | 0.529281 0.000756 0.380573 0.020142 | | | |
| 702 MAP4K1 | | 0.528855 0.000765 0.436993 0.006844 | | | |
| 703 LRRC47 | | 0.522134 0.000917 0.353486 0.031857 | | | |
| 704 ARMCX5 | | 0.519385 0.000987 0.379075 0.020679 | | | |
| 705 C6ORF199 | | 0.516693 0.00106 0.388015 0.017644 | | | |
| 706 PIG38 | | 0.511392 0.001217 0.48615 0.002284 | | | |
| 707 FLJ21865 | | 0.510345 0.001251 0.336794 0.04152 | | | |
| 708 NBPF9 | | 0.507107 0.001359 0.356075 0.030539 | | | |
| 709 ENTPD1 | | 0.498525 0.001687 0.403025 0.013388 | | | |
| 710 NARS2 | | 0.49699 0.001753 0.380735 0.020085 | | | |
| 711 PLAC8L1 | | 0.488477 0.002159 0.358402 0.029392 | | | |
| 712 MGC35206 | | 0.479643 0.002665 0.393884 0.015862 | | | |
| 713 C8ORF31 | | 0.453081 0.004862 0.389276 0.017247 | | | |
| 714 NAPA | | 0.431795 0.007617 0.413406 0.010983 | | | |
| 715 HTR1E | | 0.417606 0.010119 0.379968 0.020358 | | | |
| 716 PRPS1L1 | | 0.395495 0.0154 0.385871 0.018335 | | | |
| 717 SERPINA4 | | 0.393287 0.016036 0.329866 0.046171 | | | |
| 718 ZNF157 | | 0.373615 0.022739 0.358416 0.029386 | | | |
| 719 GEMIN7 | | 0.355054 0.031053 0.344664 0.036704 | | | |
| 720 RNF186 | | 0.338253 0.04059 0.332256 0.044521 | | | |
