# Supplementary material for: Identification of SCARA3 with potential roles in metabolic disorders
Source: Aging (Albany NY). 2020 Dec 9;13(2):2149–67. doi: 10.18632/aging.202228 (PMC7880357; doi:10.18632/aging.202228)
Supplement: Supplementary Table 4 [file aging-13-202228-s005.docx]

Supplementary Table 4: Association with adipogenic stages in red model.

| moduleGenes | MM | | MMP | | | GS | | GSP | | |  |
| --- | --- | --- | --- | --- | --- | --- | --- | --- | --- | --- | --- |
| 1 FNDC6 | 0.983084 | | 2.15E-27 | | | 0.977868 | | 2.28E-25 | | |  |
| 2 REXO2 | 0.981152 | | 1.41E-26 | | | 0.986252 | | 5.85E-29 | | |  |
| 3 TRAK2 | 0.980168 | | 3.40E-26 | | | 0.969886 | | 4.68E-23 | | |  |
| 4 EFHD1 | 0.979861 | | 4.44E-26 | | | 0.976137 | | 8.39E-25 | | |  |
| 5 FN1 | 0.978999 | | 9.17E-26 | | | 0.946496 | | 9.08E-19 | | |  |
| 6 CCND2 | 0.973758 | | 4.34E-24 | | | 0.971175 | | 2.20E-23 | | |  |
| 7 KIAA0746 | 0.972774 | | 8.21E-24 | | | 0.950238 | | 2.63E-19 | | |  |
| 8 DNAJC12 | 0.970477 | | 3.33E-23 | | | 0.97095 | | 2.52E-23 | | |  |
| 9 SH3PXD2A | 0.969953 | | 4.51E-23 | | | 0.948512 | | 4.71E-19 | | |  |
| 10 CHRNA1 | 0.969359 | | 6.32E-23 | | | 0.980254 | | 3.15E-26 | | |  |
| 11 CHST7 | 0.968422 | | 1.06E-22 | | | 0.985945 | | 8.59E-29 | | |  |
| 12 LOX | 0.965889 | | 4.02E-22 | | | 0.964559 | | 7.76E-22 | | |  |
| 13 IDS | 0.964469 | | 8.11E-22 | | | 0.976112 | | 8.54E-25 | | |  |
| 14 PURA | 0.963682 | | 1.18E-21 | | | 0.946682 | | 8.55E-19 | | |  |
| 15 COMP | 0.963609 | | 1.22E-21 | | | 0.95534 | | 4.13E-20 | | |  |
| 16 RDH5 | 0.963025 | | 1.61E-21 | | | 0.963011 | | 1.62E-21 | | |  |
| 17 CST6 | 0.962522 | | 2.03E-21 | | | 0.978035 | | 2.00E-25 | | |  |
| 18 LOC492311 | 0.960252 | | 5.58E-21 | | | 0.947306 | | 7.00E-19 | | |  |
| 19 DYNC1LI2 | 0.959178 | | 8.83E-21 | | | 0.942162 | | 3.43E-18 | | |  |
| 20 SPOCK | 0.95866 | | 1.10E-20 | | | 0.939197 | | 8.03E-18 | | |  |
| 21 CLDN1 | 0.957184 | | 2.00E-20 | | | 0.958836 | | 1.02E-20 | | |  |
| 22 ZNF651 | 0.956863 | | 2.28E-20 | | | 0.956125 | | 3.04E-20 | | |  |
| 23 CDKN2B | 0.955489 | | 3.90E-20 | | | 0.945489 | | 1.25E-18 | | |  |
| 24 PSMB5 | 0.954414 | | 5.87E-20 | | | 0.956916 | | 2.23E-20 | | |  |
| 25 BNIP3 | 0.953309 | | 8.84E-20 | | | 0.916085 | | 1.87E-15 | | |  |
| 26 ANKRD1 | 0.952965 | | 1.00E-19 | | | 0.938574 | | 9.55E-18 | | |  |
| 27 CPA4 | 0.952021 | | 1.41E-19 | | | 0.959538 | | 7.58E-21 | | |  |
| 28 RNF14 | 0.950954 | | 2.05E-19 | | | 0.949969 | | 2.88E-19 | | |  |
| 29 PRKCQ | 0.949826 | | 3.03E-19 | | | 0.949173 | | 3.78E-19 | | |  |
| 30 RGC32 | 0.949333 | | 3.58E-19 | | | 0.957223 | | 1.97E-20 | | |  |
| 31 AGA | 0.948878 | | 4.17E-19 | | | 0.937397 | | 1.32E-17 | | |  |
| 32 CALU | 0.948623 | | 4.54E-19 | | | 0.928421 | | 1.28E-16 | | |  |
| 33 NT5E | 0.948227 | | 5.18E-19 | | | 0.931547 | | 6.00E-17 | | |  |
| 34 GOSR2 | 0.948213 | | 5.20E-19 | | | 0.943412 | | 2.36E-18 | | |  |
| 35 SORT1 | 0.947544 | | 6.48E-19 | | | 0.957919 | | 1.49E-20 | | |  |
| 36 CGNL1 | 0.945662 | | 1.18E-18 | | | 0.936539 | | 1.66E-17 | | |  |
| 37 PGK1 | 0.943942 | | 2.01E-18 | | | 0.930241 | | 8.27E-17 | | |  |
| 38 PSMD14 | 0.942944 | | 2.72E-18 | | | 0.958546 | | 1.15E-20 | | |  |
| 39 COPS7A | 0.94219 | | 3.40E-18 | | | 0.957687 | | 1.63E-20 | | |  |
| 40 CCPG1 | 0.941053 | | 4.74E-18 | | | 0.898357 | | 4.64E-14 | | |  |
| 41 PARP3 | 0.941045 | | 4.75E-18 | | | 0.920341 | | 7.79E-16 | | |  |
| 42 LOC285989 | 0.940926 | | 4.91E-18 | | | 0.942168 | | 3.42E-18 | | |  |
| 43 VKORC1L1 | 0.940814 | | 5.07E-18 | | | 0.940108 | | 6.21E-18 | | |  |
| 44 P4HA2 | 0.940703 | | 5.24E-18 | | | 0.898826 | | 4.29E-14 | | |  |
| 45 HRASLS3 | 0.939524 | | 7.32E-18 | | | 0.942872 | | 2.78E-18 | | |  |
| 46 HAGH | 0.93878 | | 9.02E-18 | | | 0.954778 | | 5.11E-20 | | |  |
| 47 ORF1-FL49 | 0.938706 | | 9.20E-18 | | | 0.961378 | | 3.41E-21 | | |  |
| 48 C7ORF10 | 0.937276 | | 1.36E-17 | | | 0.944036 | | 1.96E-18 | | |  |
| 49 UCHL1 | | 0.936473 | | | 1.69E-17 | | 0.945208 | | | 1.36E-18 |  |
| 50 QDPR | | 0.936288 | | | 1.78E-17 | | 0.942201 | | | 3.39E-18 |  |
| 51 C2ORF30 | | 0.936074 | | | 1.88E-17 | | 0.898575 | | | 4.47E-14 |  |
| 52 PCSK1N | | 0.935789 | | | 2.03E-17 | | 0.936651 | | | 1.61E-17 |  |
| 53 B4GALT4 | | 0.935648 | | | 2.11E-17 | | 0.931502 | | | 6.07E-17 |  |
| 54 CHIC2 | | 0.93562 | | | 2.12E-17 | | 0.931436 | | | 6.17E-17 |  |
| 55 MRAS | | 0.9335 | | | 3.68E-17 | | 0.909302 | | | 6.90E-15 |  |
| 56 ITPR1 | | 0.93297 | | | 4.21E-17 | | 0.906887 | | | 1.07E-14 |  |
| 57 EXTL1 | | 0.932683 | | | 4.52E-17 | | 0.931906 | | | 5.49E-17 |  |
| 58 FBXO15 | | 0.931193 | | | 6.55E-17 | | 0.937649 | | | 1.23E-17 |  |
| 59 KIAA1191 | | 0.930919 | | | 7.01E-17 | | 0.947002 | | | 7.72E-19 |  |
| 60 PTPLB | | 0.930403 | | | 7.95E-17 | | 0.902629 | | | 2.26E-14 |  |
| 61 POMGNT1 | | 0.930385 | | | 7.99E-17 | | 0.93278 | | | 4.41E-17 |  |
| 62 GLRB | | 0.929826 | | | 9.15E-17 | | 0.935231 | | | 2.35E-17 |  |
| 63 FAM84B | | 0.929747 | | | 9.32E-17 | | 0.908948 | | | 7.37E-15 |  |
| 64 C1ORF24 | | 0.92926 | | | 1.05E-16 | | 0.897963 | | | 4.94E-14 |  |
| 65 COPZ2 | | 0.929242 | | | 1.05E-16 | | 0.938534 | | | 9.65E-18 |  |
| 66 RY1 | | 0.929218 | | | 1.06E-16 | | 0.926502 | | | 2.00E-16 |  |
| 67 DUSP22 | | 0.928885 | | | 1.15E-16 | | 0.936608 | | | 1.63E-17 |  |
| 68 CRYZ | | 0.927847 | | | 1.46E-16 | | 0.900911 | | | 3.03E-14 |  |
| 69 KIAA0907 | | 0.92778 | | | 1.49E-16 | | 0.922898 | | | 4.49E-16 |  |
| 70 RBP1 | | 0.927699 | | | 1.52E-16 | | 0.959425 | | | 7.95E-21 |  |
| 71 GLS | | 0.927472 | | | 1.60E-16 | | 0.904175 | | | 1.73E-14 |  |
| 72 PCDH7 | | 0.927121 | | | 1.73E-16 | | 0.890706 | | | 1.55E-13 |  |
| 73 C9ORF32 | | 0.925559 | | | 2.48E-16 | | 0.947843 | | | 5.87E-19 |  |
| 74 ITGB1BP1 | | 0.925178 | | | 2.71E-16 | | 0.95475 | | | 5.17E-20 |  |
| 75 PARP8 | | 0.924214 | | | 3.36E-16 | | 0.932614 | | | 4.60E-17 |  |
| 76 MGP | | 0.92402 | | | 3.51E-16 | | 0.938402 | | | 1.00E-17 |  |
| 77 BCL2L2 | | 0.923952 | | | 3.56E-16 | | 0.939761 | | | 6.85E-18 |  |
| 78 UBE2E2 | | 0.923374 | | | 4.05E-16 | | 0.907403 | | | 9.77E-15 |  |
| 79 TMEM45A | | 0.922339 | | | 5.07E-16 | | 0.935492 | | | 2.19E-17 |  |
| 80 USMG5 | | 0.921699 | | | 5.83E-16 | | 0.94512 | | | 1.40E-18 |  |
| 81 DMD | | 0.921651 | | | 5.89E-16 | | 0.924821 | | | 2.93E-16 |  |
| 82 RRAS2 | | 0.921199 | | | 6.49E-16 | | 0.947263 | | | 7.10E-19 |  |
| 83 SAMD3 | | 0.921088 | | | 6.65E-16 | | 0.896223 | | | 6.55E-14 |  |
| 84 PVR | | 0.920624 | | | 7.34E-16 | | 0.913987 | | | 2.83E-15 |  |
| 85 AP1S2 | | 0.919909 | | | 8.53E-16 | | 0.924152 | | | 3.41E-16 |  |
| 86 SPG20 | | 0.919533 | | | 9.23E-16 | | 0.923817 | | | 3.67E-16 |  |
| 87 TMEM49 | | 0.918998 | | | 1.03E-15 | | 0.862967 | | | 6.45E-12 |  |
| 88 CYTL1 | | 0.918988 | | | 1.03E-15 | | 0.920185 | | | 8.05E-16 |  |
| 89 NINJ2 | | 0.918458 | | | 1.15E-15 | | 0.922685 | | | 4.71E-16 |  |
| 90 NIT2 | | 0.918192 | | | 1.22E-15 | | 0.921983 | | | 5.48E-16 |  |
| 91 NQO2 | | 0.916801 | | | 1.62E-15 | | 0.947089 | | | 7.50E-19 |  |
| 92 KBTBD11 | | 0.916433 | | | 1.74E-15 | | 0.895541 | | | 7.31E-14 |  |
| 93 IQCG | | 0.916124 | | | 1.86E-15 | | 0.930289 | | | 8.18E-17 |  |
| 94 MIMITIN | | 0.916052 | | | 1.88E-15 | | 0.939452 | | | 7.47E-18 |  |
| 95 PPFIA1 | | 0.915747 | | | 2.00E-15 | | 0.891319 | | | 1.41E-13 |  |
| 96 ATP6V1E1 | | 0.915015 | | | 2.32E-15 | | 0.9004 | | | 3.30E-14 |  |
| 97 KIAA0409 | | 0.91455 | | | 2.54E-15 | | 0.929588 | | | 9.69E-17 |  |
| 98 ATP7B | | 0.914209 | | | 2.71E-15 | | 0.950359 | | | 2.52E-19 |  |
| 99 ANKMY2 | | | 0.913654 | | | 3.02E-15 | | 0.884597 | | | 3.82E-13 |
| 100 TMCO3 | | | 0.913216 | | | 3.29E-15 | | 0.92398 | | | 3.54E-16 |
| 101 AKTIP | | | 0.91272 | | | 3.62E-15 | | 0.925279 | | | 2.65E-16 |
| 102 SCOC | | | 0.911716 | | | 4.39E-15 | | 0.86471 | | | 5.24E-12 |
| 103 HIGD1A | | | 0.911225 | | | 4.82E-15 | | 0.907176 | | | 1.02E-14 |
| 104 MTPN | | | 0.911145 | | | 4.89E-15 | | 0.891036 | | | 1.47E-13 |
| 105 CDR2L | | | 0.909101 | | | 7.16E-15 | | 0.913957 | | | 2.85E-15 |
| 106 SEC23B | | | 0.908821 | | | 7.54E-15 | | 0.895059 | | | 7.89E-14 |
| 107 SLC7A5 | | | 0.90862 | | | 7.82E-15 | | 0.884257 | | | 4.01E-13 |
| 108 POGK | | | 0.908366 | | | 8.20E-15 | | 0.907406 | | | 9.76E-15 |
| 109 DHRS7B | | | 0.908274 | | | 8.34E-15 | | 0.926658 | | | 1.93E-16 |
| 110 FAM36A | | | 0.906676 | | | 1.11E-14 | | 0.914808 | | | 2.41E-15 |
| 111 FADD | | | 0.906156 | | | 1.22E-14 | | 0.895654 | | | 7.18E-14 |
| 112 CREB3 | | | 0.906115 | | | 1.23E-14 | | 0.890289 | | | 1.65E-13 |
| 113 MGC17839 | | | 0.905084 | | | 1.48E-14 | | 0.942527 | | | 3.08E-18 |
| 114 AHSA1 | | | 0.904735 | | | 1.57E-14 | | 0.924515 | | | 3.14E-16 |
| 115 C8ORF35 | | | 0.904469 | | | 1.65E-14 | | 0.936171 | | | 1.83E-17 |
| 116 GHR | | | 0.903557 | | | 1.93E-14 | | 0.880326 | | | 6.96E-13 |
| 117 DDIT4L | | | 0.903073 | | | 2.10E-14 | | 0.886693 | | | 2.82E-13 |
| 118 BLOC1S2 | | | 0.902962 | | | 2.14E-14 | | 0.876513 | | | 1.17E-12 |
| 119 C7ORF44 | | | 0.901978 | | | 2.53E-14 | | 0.929901 | | | 8.98E-17 |
| 120 LTB4DH | | | 0.901856 | | | 2.58E-14 | | 0.894453 | | | 8.68E-14 |
| 121 FLJ12571 | | | 0.901615 | | | 2.69E-14 | | 0.89131 | | | 1.41E-13 |
| 122 ANXA7 | | | 0.901239 | | | 2.87E-14 | | 0.872886 | | | 1.88E-12 |
| 123 NKIRAS1 | | | 0.900311 | | | 3.35E-14 | | 0.875398 | | | 1.35E-12 |
| 124 CD151 | | | 0.899595 | | | 3.78E-14 | | 0.913363 | | | 3.20E-15 |
| 125 PROS1 | | | 0.899401 | | | 3.90E-14 | | 0.873405 | | | 1.76E-12 |
| 126 LDHA | | | 0.899196 | | | 4.04E-14 | | 0.900419 | | | 3.29E-14 |
| 127 HSPA12A | | | 0.897739 | | | 5.13E-14 | | 0.85544 | | | 1.55E-11 |
| 128 ITGB1 | | | 0.897162 | | | 5.63E-14 | | 0.851652 | | | 2.35E-11 |
| 129 STAMBP | | | 0.8971 | | | 5.69E-14 | | 0.840413 | | | 7.69E-11 |
| 130 SUGT1 | | | 0.896471 | | | 6.30E-14 | | 0.8618 | | | 7.42E-12 |
| 131 HLA-DMA | | | 0.895884 | | | 6.92E-14 | | 0.908543 | | | 7.94E-15 |
| 132 CD58 | | | 0.895876 | | | 6.93E-14 | | 0.926947 | | | 1.81E-16 |
| 133 PSRC2 | | | 0.895293 | | | 7.60E-14 | | 0.87284 | | | 1.89E-12 |
| 134 SLC4A7 | | | 0.894824 | | | 8.19E-14 | | 0.908503 | | | 7.99E-15 |
| 135 GPD1L | | | 0.894076 | | | 9.21E-14 | | 0.871367 | | | 2.29E-12 |
| 136 LASS5 | | | 0.893898 | | | 9.47E-14 | | 0.870823 | | | 2.45E-12 |
| 137 CYB5R1 | | | 0.893761 | | | 9.68E-14 | | 0.85802 | | | 1.15E-11 |
| 138 TRAF3IP1 | | | 0.893569 | | | 9.97E-14 | | 0.920318 | | | 7.83E-16 |
| 139 HBEGF | | | 0.893155 | | | 1.06E-13 | | 0.906194 | | | 1.21E-14 |
| 140 LOC387921 | | | 0.892958 | | | 1.10E-13 | | 0.871229 | | | 2.33E-12 |
| 141 TMBIM1 | | | 0.892739 | | | 1.13E-13 | | 0.904749 | | | 1.57E-14 |
| 142 RSHL2 | | | 0.892365 | | | 1.20E-13 | | 0.887467 | | | 2.52E-13 |
| 143 ABCA3 | | | 0.891675 | | | 1.34E-13 | | 0.8839 | | | 4.22E-13 |
| 144 ACPP | | | 0.891592 | | | 1.35E-13 | | 0.89867 | | | 4.40E-14 |
| 145 NME1-NME2 | | | 0.891574 | | | 1.36E-13 | | 0.906937 | | | 1.06E-14 |
| 146 PSMA1 | | | 0.891157 | | | 1.45E-13 | | 0.872395 | | | 2.01E-12 |
| 147 FTS | | | 0.88967 | | | 1.81E-13 | | 0.864215 | | | 5.56E-12 |
| 148 SYT12 | | | 0.888717 | | | 2.09E-13 | | 0.899313 | | | 3.96E-14 |
| 149 EIF5B | | | 0.888596 | | | 2.13E-13 | | 0.914247 | | | 2.69E-15 |
| 150 RTN3 | | | 0.888031 | | | 2.32E-13 | | 0.880925 | | | 6.41E-13 |
| 151 RINT-1 | | | 0.887972 | | | 2.34E-13 | | 0.855852 | | | 1.48E-11 |
| 152 SLC20A1 | | | 0.887331 | | | 2.57E-13 | | 0.864797 | | | 5.18E-12 |
| 153 TAF13 | | | 0.887234 | | | 2.60E-13 | | 0.910477 | | | 5.55E-15 |
| 154 TXNL2 | | | 0.887068 | | | 2.67E-13 | | 0.858237 | | | 1.12E-11 |
| 155 PTPRE | | | 0.883909 | | | 4.21E-13 | | 0.920353 | | | 7.77E-16 |
| 156 PEX7 | | | 0.883528 | | | 4.45E-13 | | 0.883057 | | | 4.76E-13 |
| 157 C10ORF83 | | | 0.882821 | | | 4.92E-13 | | 0.861975 | | | 7.26E-12 |
| 158 GABBR2 | | | 0.882415 | | | 5.21E-13 | | 0.899636 | | | 3.75E-14 |
| 159 PCMT1 | | | 0.88185 | | | 5.64E-13 | | 0.845127 | | | 4.74E-11 |
| 160 PTPRM | | | 0.881846 | | | 5.64E-13 | | 0.842339 | | | 6.32E-11 |
| 161 CTPS | | | 0.881688 | | | 5.77E-13 | | 0.854857 | | | 1.65E-11 |
| 162 PSG6 | | | 0.881391 | | | 6.01E-13 | | 0.891517 | | | 1.37E-13 |
| 163 DAD1 | | | 0.880782 | | | 6.54E-13 | | 0.905411 | | | 1.39E-14 |
| 164 SETD3 | | | 0.880412 | | | 6.88E-13 | | 0.835724 | | | 1.23E-10 |
| 165 C20ORF102 | | | 0.880142 | | | 7.14E-13 | | 0.883084 | | | 4.74E-13 |
| 166 COPB2 | | | 0.880036 | | | 7.25E-13 | | 0.877455 | | | 1.03E-12 |
| 167 ACTR10 | | | 0.879167 | | | 8.17E-13 | | 0.879532 | | | 7.77E-13 |
| 168 RRS1 | | | 0.878343 | | | 9.13E-13 | | 0.866719 | | | 4.10E-12 |
| 169 ITR | | | 0.878174 | | | 9.35E-13 | | 0.836665 | | | 1.12E-10 |
| 170 CLN8 | | | 0.877948 | | | 9.64E-13 | | 0.882999 | | | 4.79E-13 |
| 171 KIAA1715 | | | 0.877628 | | | 1.01E-12 | | 0.886581 | | | 2.87E-13 |
| 172 NPAS1 | | | 0.876225 | | | 1.21E-12 | | 0.88983 | | | 1.77E-13 |
| 173 RAB3GAP1 | | | 0.875683 | | | 1.30E-12 | | 0.829155 | | | 2.31E-10 |
| 174 OCRL | | | 0.874128 | | | 1.60E-12 | | 0.8808 | | | 6.52E-13 |
| 175 TTC4 | | | 0.874008 | | | 1.63E-12 | | 0.849637 | | | 2.93E-11 |
| 176 HSPC196 | | | 0.872842 | | | 1.89E-12 | | 0.863413 | | | 6.12E-12 |
| 177 IGFBP3 | | | 0.87252 | | | 1.97E-12 | | 0.87177 | | | 2.17E-12 |
| 178 DDAH1 | | | 0.870638 | | | 2.51E-12 | | 0.846588 | | | 4.06E-11 |
| 179 LOC283537 | | | 0.870199 | | | 2.65E-12 | | 0.883506 | | | 4.46E-13 |
| 180 MAP2K1 | | | 0.869757 | | | 2.81E-12 | | 0.91474 | | | 2.44E-15 |
| 181 DYSF | | | 0.869669 | | | 2.84E-12 | | 0.890986 | | | 1.49E-13 |
| 182 NNMT | | | 0.869579 | | | 2.87E-12 | | 0.83811 | | | 9.69E-11 |
| 183 MCEE | | | 0.869456 | | | 2.91E-12 | | 0.842994 | | | 5.91E-11 |
| 184 SH3GLB1 | | | 0.869206 | | | 3.01E-12 | | 0.859922 | | | 9.25E-12 |
| 185 HEBP1 | | | 0.868971 | | | 3.10E-12 | | 0.819659 | | | 5.48E-10 |
| 186 ERCC6 | | | 0.867912 | | | 3.53E-12 | | 0.874751 | | | 1.48E-12 |
| 187 OR10A6 | | | 0.867515 | | | 3.71E-12 | | 0.856114 | | | 1.43E-11 |
| 188 CBLN3 | | | 0.867505 | | | 3.72E-12 | | 0.849605 | | | 2.94E-11 |
| 189 KDELR2 | | | 0.867149 | | | 3.89E-12 | | 0.862588 | | | 6.75E-12 |
| 190 SOCS4 | | | 0.866331 | | | 4.30E-12 | | 0.852442 | | | 2.16E-11 |
| 191 LRRC32 | | | 0.864413 | | | 5.43E-12 | | 0.833158 | | | 1.58E-10 |
| 192 SLC29A1 | | | 0.864016 | | | 5.69E-12 | | 0.884862 | | | 3.68E-13 |
| 193 SCFD1 | | | 0.863599 | | | 5.98E-12 | | 0.858626 | | | 1.07E-11 |
| 194 CEPT1 | | | 0.862721 | | | 6.65E-12 | | 0.813892 | | | 9.06E-10 |
| 195 GBE1 | | | 0.862592 | | | 6.75E-12 | | 0.840711 | | | 7.46E-11 |
| 196 KCNMB4 | | | 0.862449 | | | 6.87E-12 | | 0.888 | | | 2.33E-13 |
| 197 STARD5 | | | 0.861512 | | | 7.67E-12 | | 0.873663 | | | 1.70E-12 |
| 198 C11ORF24 | | | 0.861028 | | | 8.12E-12 | | 0.825597 | | | 3.21E-10 |
| 199 TRAPPC4 | | | 0.860489 | | | 8.65E-12 | | 0.841543 | | | 6.86E-11 |
| 200 C10ORF7 | | | 0.860332 | | | 8.81E-12 | | 0.840926 | | | 7.30E-11 |
| 201 GOLGA7 | | | 0.860301 | | | 8.84E-12 | | 0.825864 | | | 3.13E-10 |
| 202 NDUFA4 | | | 0.860166 | | | 8.99E-12 | | 0.893734 | | | 9.72E-14 |
| 203 DUSP26 | | | 0.860034 | | | 9.13E-12 | | 0.849882 | | | 2.86E-11 |
| 204 OGFOD1 | | | 0.859338 | | | 9.90E-12 | | 0.894326 | | | 8.86E-14 |
| 205 CPXM2 | | | 0.858985 | | | 1.03E-11 | | 0.857606 | | | 1.21E-11 |
| 206 MFSD3 | | | 0.858744 | | | 1.06E-11 | | 0.843119 | | | 5.83E-11 |
| 207 SLCO2A1 | | | 0.858084 | | | 1.14E-11 | | 0.853186 | | | 1.99E-11 |
| 208 RAB2 | | | 0.85802 | | | 1.15E-11 | | 0.855629 | | | 1.51E-11 |
| 209 SPIRE1 | | | 0.856541 | | | 1.36E-11 | | 0.800794 | | | 2.66E-09 |
| 210 MRPL52 | | | 0.856127 | | | 1.43E-11 | | 0.878257 | | | 9.24E-13 |
| 211 HHAT | | | 0.856046 | | | 1.44E-11 | | 0.867131 | | | 3.89E-12 |
| 212 C21ORF34 | | | 0.855291 | | | 1.57E-11 | | 0.885143 | | | 3.53E-13 |
| 213 XKR6 | | | 0.854829 | | | 1.66E-11 | | 0.885365 | | | 3.42E-13 |
| 214 TNFSF4 | | | 0.853838 | | | 1.85E-11 | | 0.82179 | | | 4.54E-10 |
| 215 SDPR | | | 0.852901 | | | 2.05E-11 | | 0.838422 | | | 9.40E-11 |
| 216 PCBD1 | | | 0.850881 | | | 2.56E-11 | | 0.879391 | | | 7.92E-13 |
| 217 VCPIP1 | | | 0.850611 | | | 2.64E-11 | | 0.807145 | | | 1.59E-09 |
| 218 CYB5R3 | | | 0.850219 | | | 2.75E-11 | | 0.827743 | | | 2.63E-10 |
| 219 TSPAN9 | | | 0.849666 | | | 2.92E-11 | | 0.847 | | | 3.89E-11 |
| 220 SYBL1 | | | 0.849469 | | | 2.99E-11 | | 0.828361 | | | 2.48E-10 |
| 221 TOR1AIP2 | | | 0.849098 | | | 3.11E-11 | | 0.784618 | | | 9.08E-09 |
| 222 YIPF6 | | | 0.84853 | | | 3.30E-11 | | 0.786391 | | | 7.98E-09 |
| 223 TTC1 | | | 0.848246 | | | 3.40E-11 | | 0.859623 | | | 9.57E-12 |
| 224 LACTB | | | 0.848132 | | | 3.45E-11 | | 0.829456 | | | 2.24E-10 |
| 225 KLHL18 | | | 0.847885 | | | 3.54E-11 | | 0.846682 | | | 4.02E-11 |
| 226 KIAA0143 | | | 0.847391 | | | 3.73E-11 | | 0.816421 | | | 7.28E-10 |
| 227 ZNF226 | | | 0.847213 | | | 3.80E-11 | | 0.858846 | | | 1.05E-11 |
| 228 EGFL9 | | | 0.846177 | | | 4.24E-11 | | 0.822458 | | | 4.27E-10 |
| 229 SLC36A4 | | | 0.845363 | | | 4.62E-11 | | 0.858473 | | | 1.09E-11 |
| 230 C6ORF113 | | | 0.844494 | | | 5.06E-11 | | 0.823366 | | | 3.93E-10 |
| 231 CIB1 | | | 0.843321 | | | 5.71E-11 | | 0.870782 | | | 2.46E-12 |
| 232 EPHA4 | | | 0.841349 | | | 6.99E-11 | | 0.847782 | | | 3.58E-11 |
| 233 ALDOA | | | 0.841137 | | | 7.15E-11 | | 0.819506 | | | 5.56E-10 |
| 234 TLOC1 | | | 0.840795 | | | 7.40E-11 | | 0.865629 | | | 4.68E-12 |
| 235 PAPSS2 | | | 0.840794 | | | 7.40E-11 | | 0.802531 | | | 2.32E-09 |
| 236 HPS5 | | | 0.840763 | | | 7.42E-11 | | 0.852878 | | | 2.06E-11 |
| 237 NPLOC4 | | | 0.840265 | | | 7.81E-11 | | 0.888414 | | | 2.19E-13 |
| 238 LOC130576 | | | 0.840109 | | | 7.93E-11 | | 0.810076 | | | 1.25E-09 |
| 239 PPM1B | | | 0.840022 | | | 8.00E-11 | | 0.791795 | | | 5.34E-09 |
| 240 MYH4 | | | 0.839695 | | | 8.27E-11 | | 0.856996 | | | 1.30E-11 |
| 241 RAP1GDS1 | | | 0.838993 | | | 8.88E-11 | | 0.836666 | | | 1.12E-10 |
| 242 SDHB | | | 0.838739 | | | 9.10E-11 | | 0.856798 | | | 1.33E-11 |
| 243 KIF2 | | | 0.838396 | | | 9.42E-11 | | 0.838681 | | | 9.16E-11 |
| 244 LOC643940 | | | 0.836989 | | | 1.08E-10 | | 0.799245 | | | 3.01E-09 |
| 245 ABLIM1 | | | 0.836978 | | | 1.08E-10 | | 0.877144 | | | 1.07E-12 |
| 246 HSF2BP | | | 0.836591 | | | 1.13E-10 | | 0.860768 | | | 8.37E-12 |
| 247 RGS4 | | | 0.836305 | | | 1.16E-10 | | 0.837135 | | | 1.07E-10 |
| 248 JAM2 | | | 0.83603 | | | 1.19E-10 | | 0.83931 | | | 8.60E-11 |
| 249 CD14 | | | 0.835986 | | | 1.20E-10 | | 0.832845 | | | 1.62E-10 |
| 250 FBXL2 | | | 0.835774 | | | 1.22E-10 | | 0.846477 | | | 4.11E-11 |
| 251 ABI3BP | | | 0.835641 | | | 1.24E-10 | | 0.823287 | | | 3.96E-10 |
| 252 C4ORF9 | | | 0.834181 | | | 1.43E-10 | | 0.780306 | | | 1.24E-08 |
| 253 SYT17 | | | 0.832987 | | | 1.60E-10 | | 0.828483 | | | 2.46E-10 |
| 254 COG6 | | | 0.832874 | | | 1.62E-10 | | 0.774188 | | | 1.90E-08 |
| 255 CUGBP1 | | | 0.832706 | | | 1.65E-10 | | 0.796617 | | | 3.69E-09 |
| 256 ORC3L | | | 0.832451 | | | 1.69E-10 | | 0.837083 | | | 1.07E-10 |
| 257 C8ORF48 | | | 0.831648 | | | 1.82E-10 | | 0.797622 | | | 3.42E-09 |
| 258 SEDLP | | | 0.830833 | | | 1.97E-10 | | 0.848102 | | | 3.46E-11 |
| 259 RPS6KC1 | | | 0.829615 | | | 2.21E-10 | | 0.844633 | | | 4.99E-11 |
| 260 MPP6 | | | 0.829501 | | | 2.23E-10 | | 0.861238 | | | 7.92E-12 |
| 261 RPL6 | | | 0.829472 | | | 2.24E-10 | | 0.79227 | | | 5.15E-09 |
| 262 C6ORF69 | | | 0.829438 | | | 2.25E-10 | | 0.813992 | | | 8.98E-10 |
| 263 PTPN11 | | | 0.828033 | | | 2.56E-10 | | 0.774606 | | | 1.84E-08 |
| 264 NDUFA8 | | | 0.827088 | | | 2.80E-10 | | 0.809931 | | | 1.27E-09 |
| 265 AGPS | | | 0.823947 | | | 3.73E-10 | | 0.767711 | | | 2.94E-08 |
| 266 ENC1 | | | 0.82299 | | | 4.07E-10 | | 0.790836 | | | 5.74E-09 |
| 267 VAMP1 | | | 0.821313 | | | 4.73E-10 | | 0.837418 | | | 1.04E-10 |
| 268 ARSK | | | 0.820947 | | | 4.89E-10 | | 0.784685 | | | 9.04E-09 |
| 269 CFLAR | | | 0.819868 | | | 5.38E-10 | | 0.844745 | | | 4.93E-11 |
| 270 P15RS | | | 0.818899 | | | 5.86E-10 | | 0.768983 | | | 2.70E-08 |
| 271 PALLD | | | 0.818799 | | | 5.92E-10 | | 0.774737 | | | 1.83E-08 |
| 272 ARHGEF6 | | | 0.818691 | | | 5.97E-10 | | 0.836152 | | | 1.18E-10 |
| 273 FLJ32549 | | | 0.818092 | | | 6.30E-10 | | 0.801917 | | | 2.44E-09 |
| 274 RGS5 | | | 0.818073 | | | 6.31E-10 | | 0.859454 | | | 9.76E-12 |
| 275 FAM79A | | | 0.816904 | | | 6.98E-10 | | 0.760201 | | | 4.81E-08 |
| 276 ZNF622 | | | 0.816722 | | | 7.10E-10 | | 0.821689 | | | 4.58E-10 |
| 277 SNX11 | | | 0.816341 | | | 7.34E-10 | | 0.815304 | | | 8.02E-10 |
| 278 C17ORF58 | | | 0.815233 | | | 8.07E-10 | | 0.836849 | | | 1.10E-10 |
| 279 ACP1 | | | 0.814373 | | | 8.69E-10 | | 0.745022 | | | 1.23E-07 |
| 280 CRH | | | 0.81411 | | | 8.89E-10 | | 0.844948 | | | 4.82E-11 |
| 281 ALDH9A1 | | | 0.812729 | | | 1.00E-09 | | 0.74241 | | | 1.44E-07 |
| 282 PSMD10 | | | 0.812483 | | | 1.02E-09 | | 0.765891 | | | 3.32E-08 |
| 283 TFG | | | 0.811676 | | | 1.09E-09 | | 0.771809 | | | 2.23E-08 |
| 284 RNGTT | | | 0.810718 | | | 1.19E-09 | | 0.774307 | | | 1.88E-08 |
| 285 LAPTM4B | | | 0.810631 | | | 1.19E-09 | | 0.818546 | | | 6.05E-10 |
| 286 CCT4 | | | 0.810618 | | | 1.20E-09 | | 0.760156 | | | 4.83E-08 |
| 287 PDIA5 | | | 0.810352 | | | 1.22E-09 | | 0.809192 | | | 1.35E-09 |
| 288 RER1 | | | 0.809344 | | | 1.33E-09 | | 0.81047 | | | 1.21E-09 |
| 289 DIDO1 | | | 0.808879 | | | 1.38E-09 | | 0.818801 | | | 5.92E-10 |
| 290 NPTN | | | 0.808089 | | | 1.48E-09 | | 0.761496 | | | 4.43E-08 |
| 291 FLJ21901 | | | 0.807788 | | | 1.51E-09 | | 0.796067 | | | 3.85E-09 |
| 292 ATP5G3 | | | 0.80656 | | | 1.67E-09 | | 0.779823 | | | 1.28E-08 |
| 293 SMARCAL1 | | | 0.804599 | | | 1.96E-09 | | 0.793487 | | | 4.70E-09 |
| 294 ALDH5A1 | | | 0.804396 | | | 2.00E-09 | | 0.777515 | | | 1.51E-08 |
| 295 ATF6 | | | 0.80348 | | | 2.15E-09 | | 0.790923 | | | 5.70E-09 |
| 296 RPS7 | | | 0.803185 | | | 2.20E-09 | | 0.798363 | | | 3.22E-09 |
| 297 MGC3207 | | | 0.802831 | | | 2.26E-09 | | 0.782025 | | | 1.10E-08 |
| 298 SHRM | | | 0.802261 | | | 2.37E-09 | | 0.791889 | | | 5.30E-09 |
| 299 CLDND1 | | | 0.801601 | | | 2.50E-09 | | 0.759537 | | | 5.02E-08 |
| 300 SUCLA2 | | | 0.800818 | | | 2.66E-09 | | 0.743691 | | | 1.33E-07 |
| 301 CLCC1 | | | 0.799968 | | | 2.84E-09 | | 0.7817 | | | 1.12E-08 |
| 302 MAPK9 | | | 0.799467 | | | 2.96E-09 | | 0.794662 | | | 4.29E-09 |
| 303 RFWD2 | | | 0.799303 | | | 3.00E-09 | | 0.759982 | | | 4.88E-08 |
| 304 DRD1IP | | | 0.797839 | | | 3.36E-09 | | 0.798105 | | | 3.29E-09 |
| 305 FBXO22 | | | 0.795329 | | | 4.08E-09 | | 0.731076 | | | 2.77E-07 |
| 306 LOC133308 | | | 0.794615 | | | 4.31E-09 | | 0.767446 | | | 3.00E-08 |
| 307 TRAPPC3 | | | 0.794557 | | | 4.33E-09 | | 0.735652 | | | 2.13E-07 |
| 308 AFTPH | | | 0.794035 | | | 4.51E-09 | | 0.783468 | | | 9.87E-09 |
| 309 PAFAH1B2 | | | 0.793759 | | | 4.60E-09 | | 0.822228 | | | 4.36E-10 |
| 310 PRO0149 | | | 0.793152 | | | 4.82E-09 | | 0.734346 | | | 2.30E-07 |
| 311 MRPS23 | | | 0.793139 | | | 4.82E-09 | | 0.777947 | | | 1.46E-08 |
| 312 PGBD3 | | | 0.79187 | | | 5.31E-09 | | 0.753822 | | | 7.20E-08 |
| 313 IDH3A | | | 0.790357 | | | 5.95E-09 | | 0.764228 | | | 3.71E-08 |
| 314 ATPIF1 | | | 0.790181 | | | 6.03E-09 | | 0.836948 | | | 1.09E-10 |
| 315 FBXW11 | | | 0.789995 | | | 6.11E-09 | | 0.7655 | | | 3.41E-08 |
| 316 ACY1L2 | | | 0.789313 | | | 6.43E-09 | | 0.808238 | | | 1.46E-09 |
| 317 ACTR1B | | | 0.788297 | | | 6.94E-09 | | 0.809371 | | | 1.33E-09 |
| 318 ACOT8 | | | 0.787449 | | | 7.39E-09 | | 0.778712 | | | 1.39E-08 |
| 319 WDR77 | | | 0.786144 | | | 8.13E-09 | | 0.783232 | | | 1.00E-08 |
| 320 GRAMD3 | | | 0.785563 | | | 8.48E-09 | | 0.733162 | | | 2.46E-07 |
| 321 RBX1 | | | 0.785448 | | | 8.55E-09 | | 0.825442 | | | 3.26E-10 |
| 322 ZBTB8OS | | | 0.784042 | | | 9.47E-09 | | 0.805805 | | | 1.78E-09 |
| 323 HSP90AA1 | | | 0.783772 | | | 9.66E-09 | | 0.783928 | | | 9.55E-09 |
| 324 PGBD5 | | | 0.783702 | | | 9.71E-09 | | 0.813404 | | | 9.44E-10 |
| 325 SLC25A23 | | | 0.780836 | | | 1.19E-08 | | 0.833813 | | | 1.48E-10 |
| 326 LZTFL1 | | | 0.77919 | | | 1.34E-08 | | 0.757028 | | | 5.89E-08 |
| 327 GCS1 | | | 0.777839 | | | 1.47E-08 | | 0.793112 | | | 4.83E-09 |
| 328 GAL3ST2 | | | 0.77769 | | | 1.49E-08 | | 0.747753 | | | 1.05E-07 |
| 329 SEMA7A | | | 0.777153 | | | 1.55E-08 | | 0.758329 | | | 5.42E-08 |
| 330 IQGAP2 | | | 0.776593 | | | 1.61E-08 | | 0.757722 | | | 5.64E-08 |
| 331 GDAP2 | | | 0.776301 | | | 1.64E-08 | | 0.718269 | | | 5.58E-07 |
| 332 CUL5 | | | 0.773058 | | | 2.05E-08 | | 0.743864 | | | 1.32E-07 |
| 333 ST13 | | | 0.770281 | | | 2.48E-08 | | 0.698971 | | | 1.50E-06 |
| 334 TAX1BP1 | | | 0.770156 | | | 2.50E-08 | | 0.721072 | | | 4.80E-07 |
| 335 REEP2 | | | 0.76984 | | | 2.55E-08 | | 0.828293 | | | 2.50E-10 |
| 336 ADK | | | 0.769784 | | | 2.56E-08 | | 0.725444 | | | 3.78E-07 |
| 337 MPP2 | | | 0.769638 | | | 2.59E-08 | | 0.773206 | | | 2.03E-08 |
| 338 NOC3L | | | 0.769542 | | | 2.60E-08 | | 0.720399 | | | 4.98E-07 |
| 339 MRPS17 | | | 0.768154 | | | 2.86E-08 | | 0.79895 | | | 3.08E-09 |
| 340 MGC15763 | | | 0.766144 | | | 3.27E-08 | | 0.728539 | | | 3.19E-07 |
| 341 PSMD11 | | | 0.764605 | | | 3.62E-08 | | 0.780461 | | | 1.22E-08 |
| 342 LOC283219 | | | 0.764546 | | | 3.63E-08 | | 0.773695 | | | 1.96E-08 |
| 343 DDX26 | | | 0.764271 | | | 3.70E-08 | | 0.716161 | | | 6.23E-07 |
| 344 CCT3 | | | 0.762477 | | | 4.15E-08 | | 0.724088 | | | 4.08E-07 |
| 345 EDIL3 | | | 0.761738 | | | 4.36E-08 | | 0.698708 | | | 1.51E-06 |
| 346 CCT8 | | | 0.761207 | | | 4.51E-08 | | 0.710741 | | | 8.27E-07 |
| 347 RIMS3 | | | 0.760818 | | | 4.62E-08 | | 0.757317 | | | 5.78E-08 |
| 348 WDR36 | | | 0.759881 | | | 4.91E-08 | | 0.722076 | | | 4.55E-07 |
| 349 RFXDC2 | | | 0.759223 | | | 5.12E-08 | | 0.798868 | | | 3.10E-09 |
| 350 ARNTL | | | 0.758858 | | | 5.24E-08 | | 0.72832 | | | 3.23E-07 |
| 351 C1QTNF7 | | | 0.756896 | | | 5.94E-08 | | 0.758674 | | | 5.31E-08 |
| 352 LOC162073 | | | 0.755843 | | | 6.35E-08 | | 0.754714 | | | 6.81E-08 |
| 353 SLC41A2 | | | 0.755821 | | | 6.36E-08 | | 0.732342 | | | 2.58E-07 |
| 354 ZNF573 | | | 0.754366 | | | 6.96E-08 | | 0.689836 | | | 2.32E-06 |
| 355 TTL | | | 0.752497 | | | 7.82E-08 | | 0.730066 | | | 2.93E-07 |
| 356 LGALS3 | | | 0.750751 | | | 8.71E-08 | | 0.77309 | | | 2.05E-08 |
| 357 ZF | | | 0.749111 | | | 9.63E-08 | | 0.718328 | | | 5.56E-07 |
| 358 VMD2 | | | 0.748155 | | | 1.02E-07 | | 0.728186 | | | 3.25E-07 |
| 359 ICAM5 | | | 0.747953 | | | 1.03E-07 | | 0.764453 | | | 3.65E-08 |
| 360 C5ORF3 | | | 0.747229 | | | 1.08E-07 | | 0.727002 | | | 3.47E-07 |
| 361 ZHX3 | | | 0.744933 | | | 1.24E-07 | | 0.704741 | | | 1.12E-06 |
| 362 IGFL3 | | | 0.744802 | | | 1.25E-07 | | 0.712342 | | | 7.61E-07 |
| 363 TIMM23 | | | 0.742819 | | | 1.41E-07 | | 0.78882 | | | 6.67E-09 |
| 364 RAG1AP1 | | | 0.742739 | | | 1.41E-07 | | 0.766308 | | | 3.23E-08 |
| 365 FOLR3 | | | 0.742738 | | | 1.41E-07 | | 0.746655 | | | 1.12E-07 |
| 366 SPAG16 | | | 0.742537 | | | 1.43E-07 | | 0.784609 | | | 9.09E-09 |
| 367 UFD1L | | | 0.741783 | | | 1.49E-07 | | 0.763588 | | | 3.86E-08 |
| 368 LOC387856 | | | 0.741259 | | | 1.54E-07 | | 0.731291 | | | 2.73E-07 |
| 369 UBE2A | | | 0.739693 | | | 1.69E-07 | | 0.682859 | | | 3.22E-06 |
| 370 POLR3B | | | 0.739003 | | | 1.76E-07 | | 0.671398 | | | 5.40E-06 |
| 371 CTDSPL | | | 0.73875 | | | 1.79E-07 | | 0.765296 | | | 3.45E-08 |
| 372 ACOX1 | | | 0.738309 | | | 1.83E-07 | | 0.668164 | | | 6.22E-06 |
| 373 LFNG | | | 0.73807 | | | 1.86E-07 | | 0.742328 | | | 1.45E-07 |
| 374 MT1G | | | 0.737717 | | | 1.90E-07 | | 0.734674 | | | 2.26E-07 |
| 375 SLC35B3 | | | 0.737536 | | | 1.92E-07 | | 0.660835 | | | 8.52E-06 |
| 376 NECAP1 | | | 0.737246 | | | 1.95E-07 | | 0.676128 | | | 4.37E-06 |
| 377 DLG1 | | | 0.736164 | | | 2.07E-07 | | 0.743561 | | | 1.35E-07 |
| 378 RIOK2 | | | 0.735665 | | | 2.13E-07 | | 0.659536 | | | 9.00E-06 |
| 379 RPL23AP7 | | | 0.735249 | | | 2.18E-07 | | 0.750496 | | | 8.85E-08 |
| 380 ZNF484 | | | 0.734896 | | | 2.23E-07 | | 0.691114 | | | 2.19E-06 |
| 381 GAPVD1 | | | 0.73467 | | | 2.26E-07 | | 0.686853 | | | 2.67E-06 |
| 382 PTPRN | | | 0.731007 | | | 2.78E-07 | | 0.736523 | | | 2.03E-07 |
| 383 DAPK2 | | | 0.729903 | | | 2.96E-07 | | 0.731848 | | | 2.65E-07 |
| 384 DYNLL1 | | | 0.728852 | | | 3.13E-07 | | 0.720769 | | | 4.88E-07 |
| 385 ZDHHC16 | | | 0.728442 | | | 3.21E-07 | | 0.654665 | | | 1.10E-05 |
| 386 COL4A1 | | | 0.728077 | | | 3.27E-07 | | 0.667705 | | | 6.34E-06 |
| 387 LOC642090 | | | 0.727559 | | | 3.37E-07 | | 0.648363 | | | 1.43E-05 |
| 388 BCAP29 | | | 0.727173 | | | 3.44E-07 | | 0.724582 | | | 3.97E-07 |
| 389 ANKDD1A | | | 0.72656 | | | 3.56E-07 | | 0.717289 | | | 5.87E-07 |
| 390 MASA | | | 0.72577 | | | 3.72E-07 | | 0.63092 | | | 2.84E-05 |
| 391 INSIG2 | | | 0.723471 | | | 4.21E-07 | | 0.669822 | | | 5.78E-06 |
| 392 PSMC2 | | | 0.722862 | | | 4.36E-07 | | 0.695666 | | | 1.76E-06 |
| 393 IFRD2 | | | 0.721172 | | | 4.77E-07 | | 0.729151 | | | 3.08E-07 |
| 394 POLR2C | | | 0.721144 | | | 4.78E-07 | | 0.717145 | | | 5.92E-07 |
| 395 SERF2 | | | 0.720381 | | | 4.98E-07 | | 0.726436 | | | 3.58E-07 |
| 396 ZNF471 | | | 0.720006 | | | 5.08E-07 | | 0.670479 | | | 5.62E-06 |
| 397 E2F5 | | | 0.718443 | | | 5.52E-07 | | 0.712368 | | | 7.60E-07 |
| 398 TRPC1 | | | 0.717837 | | | 5.71E-07 | | 0.651351 | | | 1.27E-05 |
| 399 COX7B | | | 0.717707 | | | 5.74E-07 | | 0.744488 | | | 1.27E-07 |
| 400 2'-PDE | | | 0.717707 | | | 5.74E-07 | | 0.718734 | | | 5.44E-07 |
| 401 L3MBTL | | | 0.716619 | | | 6.09E-07 | | 0.688914 | | | 2.43E-06 |
| 402 RECQL | | | 0.716229 | | | 6.21E-07 | | 0.657488 | | | 9.81E-06 |
| 403 CSS3 | | | 0.715865 | | | 6.33E-07 | | 0.672985 | | | 5.03E-06 |
| 404 ATP6V1A | | | 0.714418 | | | 6.83E-07 | | 0.656748 | | | 1.01E-05 |
| 405 LIPG | | | 0.711285 | | | 8.04E-07 | | 0.696646 | | | 1.68E-06 |
| 406 TRIAP1 | | | 0.708961 | | | 9.06E-07 | | 0.691357 | | | 2.16E-06 |
| 407 EIF5A | | | 0.708349 | | | 9.35E-07 | | 0.751804 | | | 8.17E-08 |
| 408 RNASEH1 | | | 0.708015 | | | 9.51E-07 | | 0.674107 | | | 4.79E-06 |
| 409 NAT1 | | | 0.708015 | | | 9.51E-07 | | 0.66543 | | | 7.00E-06 |
| 410 DARS | | | 0.707949 | | | 9.54E-07 | | 0.672834 | | | 5.06E-06 |
| 411 NSUN3 | | | 0.707827 | | | 9.60E-07 | | 0.685482 | | | 2.85E-06 |
| 412 C3ORF18 | | | 0.706753 | | | 1.01E-06 | | 0.714646 | | | 6.75E-07 |
| 413 15E1.2 | | | 0.705614 | | | 1.07E-06 | | 0.664241 | | | 7.37E-06 |
| 414 TATDN3 | | | 0.705541 | | | 1.08E-06 | | 0.625417 | | | 3.49E-05 |
| 415 GSDML | | | 0.70459 | | | 1.13E-06 | | 0.708723 | | | 9.17E-07 |
| 416 DSP | | | 0.704511 | | | 1.14E-06 | | 0.679344 | | | 3.78E-06 |
| 417 EGLN1 | | | 0.703816 | | | 1.18E-06 | | 0.684278 | | | 3.01E-06 |
| 418 HIST1H1C | | | 0.703738 | | | 1.18E-06 | | 0.722697 | | | 4.40E-07 |
| 419 ATG5 | | | 0.703676 | | | 1.18E-06 | | 0.694446 | | | 1.86E-06 |
| 420 MAP6 | | | 0.703613 | | | 1.19E-06 | | 0.678733 | | | 3.89E-06 |
| 421 MTHFD2L | | | 0.702541 | | | 1.25E-06 | | 0.645323 | | | 1.62E-05 |
| 422 COPS4 | | | 0.702455 | | | 1.26E-06 | | 0.685817 | | | 2.81E-06 |
| 423 FLJ40448 | | | 0.702298 | | | 1.27E-06 | | 0.651149 | | | 1.28E-05 |
| 424 CDK5 | | | 0.702239 | | | 1.27E-06 | | 0.729389 | | | 3.04E-07 |
| 425 FLJ31438 | | | 0.702152 | | | 1.28E-06 | | 0.642958 | | | 1.78E-05 |
| 426 EDARADD | | | 0.701638 | | | 1.31E-06 | | 0.662613 | | | 7.90E-06 |
| 427 RASSF4 | | | 0.701518 | | | 1.32E-06 | | 0.701638 | | | 1.31E-06 |
| 428 ASB1 | | | 0.701467 | | | 1.32E-06 | | 0.678508 | | | 3.93E-06 |
| 429 PITX2 | | | 0.70139 | | | 1.33E-06 | | 0.725787 | | | 3.71E-07 |
| 430 FLNB | | | 0.701362 | | | 1.33E-06 | | 0.729884 | | | 2.96E-07 |
| 431 SCNM1 | | | 0.701156 | | | 1.34E-06 | | 0.672262 | | | 5.19E-06 |
| 432 LOC221955 | | | 0.699702 | | | 1.44E-06 | | 0.647896 | | | 1.46E-05 |
| 433 SCFD2 | | | 0.695014 | | | 1.81E-06 | | 0.714016 | | | 6.98E-07 |
| 434 HUWE1 | | | 0.694164 | | | 1.89E-06 | | 0.697108 | | | 1.64E-06 |
| 435 HIVEP2 | | | 0.693529 | | | 1.95E-06 | | 0.689202 | | | 2.39E-06 |
| 436 ZNF343 | | | 0.690217 | | | 2.28E-06 | | 0.655175 | | | 1.08E-05 |
| 437 SND1 | | | 0.689889 | | | 2.32E-06 | | 0.746192 | | | 1.15E-07 |
| 438 MMAA | | | 0.689517 | | | 2.36E-06 | | 0.70757 | | | 9.73E-07 |
| 439 GRPEL2 | | | 0.689025 | | | 2.41E-06 | | 0.60324 | | | 7.75E-05 |
| 440 CXORF39 | | | 0.688876 | | | 2.43E-06 | | 0.658686 | | | 9.33E-06 |
| 441 ZNF460 | | | 0.68661 | | | 2.70E-06 | | 0.6739 | | | 4.83E-06 |
| 442 SPIN2B | | | 0.685153 | | | 2.89E-06 | | 0.662265 | | | 8.02E-06 |
| 443 UBL5 | | | 0.684701 | | | 2.96E-06 | | 0.705214 | | | 1.10E-06 |
| 444 EAF2 | | | 0.684617 | | | 2.97E-06 | | 0.65117 | | | 1.27E-05 |
| 445 KIAA1468 | | | 0.682964 | | | 3.20E-06 | | 0.701579 | | | 1.31E-06 |
| 446 CHCHD8 | | | 0.682644 | | | 3.25E-06 | | 0.678891 | | | 3.86E-06 |
| 447 ACSL4 | | | 0.682527 | | | 3.27E-06 | | 0.698959 | | | 1.50E-06 |
| 448 FN5 | | | 0.682506 | | | 3.27E-06 | | 0.687594 | | | 2.58E-06 |
| 449 MIB1 | | | 0.681798 | | | 3.38E-06 | | 0.673243 | | 4.97E-06 | |
| 450 HSPG2 | | | 0.680224 | | | 3.63E-06 | | 0.663989 | | 7.45E-06 | |
| 451 BRE | | | 0.679618 | | | 3.73E-06 | | 0.651039 | | 1.28E-05 | |
| 452 HOMER2 | | | 0.678232 | | | 3.98E-06 | | 0.66418 | | 7.39E-06 | |
| 453 ATP6V1B2 | | | 0.6765 | | | 4.30E-06 | | 0.674472 | | 4.71E-06 | |
| 454 UBR2 | | | 0.676396 | | | 4.32E-06 | | 0.6485 | | 1.42E-05 | |
| 455 KRT16 | | | 0.676343 | | | 4.33E-06 | | 0.651264 | | 1.27E-05 | |
| 456 IMP4 | | | 0.676094 | | | 4.38E-06 | | 0.658454 | | 9.42E-06 | |
| 457 TRPT1 | | | 0.675827 | | | 4.43E-06 | | 0.715796 | | 6.36E-07 | |
| 458 NANS | | | 0.675777 | | | 4.44E-06 | | 0.663835 | | 7.50E-06 | |
| 459 C18ORF45 | | | 0.67529 | | | 4.54E-06 | | 0.634584 | | 2.46E-05 | |
| 460 ABCF2 | | | 0.673698 | | | 4.87E-06 | | 0.616392 | | 4.86E-05 | |
| 461 SERINC3 | | | 0.673571 | | | 4.90E-06 | | 0.654977 | | 1.09E-05 | |
| 462 ASMTL | | | 0.671543 | | | 5.36E-06 | | 0.721804 | | 4.61E-07 | |
| 463 SLC2A8 | | | 0.671088 | | | 5.47E-06 | | 0.657988 | | 9.61E-06 | |
| 464 CMKOR1 | | | 0.669454 | | | 5.88E-06 | | 0.693209 | | 1.98E-06 | |
| 465 C13ORF1 | | | 0.669305 | | | 5.92E-06 | | 0.666405 | | 6.71E-06 | |
| 466 C6ORF205 | | | 0.667935 | | | 6.28E-06 | | 0.689003 | | 2.42E-06 | |
| 467 PRPF4B | | | 0.667472 | | | 6.41E-06 | | 0.648548 | | 1.42E-05 | |
| 468 UBXD6 | | | 0.66629 | | | 6.75E-06 | | 0.662225 | | 8.03E-06 | |
| 469 COL4A2 | | | 0.666012 | | | 6.83E-06 | | 0.637425 | | 2.21E-05 | |
| 470 NEK11 | | | 0.665517 | | | 6.98E-06 | | 0.632785 | | 2.64E-05 | |
| 471 ABCE1 | | | 0.66479 | | | 7.20E-06 | | 0.606843 | | 6.84E-05 | |
| 472 VTI1B | | | 0.664719 | | | 7.22E-06 | | 0.684603 | | 2.97E-06 | |
| 473 AP3S2 | | | 0.662501 | | | 7.94E-06 | | 0.618973 | | 4.43E-05 | |
| 474 SYNPO2L | | | 0.660992 | | | 8.47E-06 | | 0.635825 | | 2.35E-05 | |
| 475 SRRP35 | | | 0.660061 | | | 8.81E-06 | | 0.68442 | | 2.99E-06 | |
| 476 SRFBP1 | | | 0.659503 | | | 9.02E-06 | | 0.626316 | | 3.37E-05 | |
| 477 STK38L | | | 0.658772 | | | 9.30E-06 | | 0.636172 | | 2.32E-05 | |
| 478 SELI | | | 0.658742 | | | 9.31E-06 | | 0.605146 | | 7.26E-05 | |
| 479 LIMK1 | | | 0.658245 | | | 9.51E-06 | | 0.691681 | | 2.13E-06 | |
| 480 RHPN1 | | | 0.657248 | | | 9.91E-06 | | 0.641241 | | 1.90E-05 | |
| 481 PCDHB16 | | | 0.656844 | | | 1.01E-05 | | 0.670071 | | 5.72E-06 | |
| 482 FGF1 | | | 0.656191 | | | 1.04E-05 | | 0.676641 | | 4.27E-06 | |
| 483 MDH1 | | | 0.655889 | | | 1.05E-05 | | 0.649673 | | 1.36E-05 | |
| 484 HOXB6 | | | 0.655759 | | | 1.05E-05 | | 0.71183 | | 7.82E-07 | |
| 485 LOC344405 | | | 0.655463 | | | 1.07E-05 | | 0.626721 | | 3.32E-05 | |
| 486 TXNDC10 | | | 0.654799 | | | 1.10E-05 | | 0.581842 | | 0.000159 | |
| 487 SAP18 | | | 0.654644 | | | 1.10E-05 | | 0.592135 | | 0.000113 | |
| 488 FOXD4 | | | 0.65395 | | | 1.14E-05 | | 0.635345 | | 2.39E-05 | |
| 489 SRXN1 | | | 0.653906 | | | 1.14E-05 | | 0.622877 | | 3.84E-05 | |
| 490 CCDC55 | | | 0.653446 | | | 1.16E-05 | | 0.609046 | | 6.33E-05 | |
| 491 MGC72080 | | | 0.653203 | | | 1.17E-05 | | 0.699568 | | 1.45E-06 | |
| 492 EXOSC1 | | | 0.651307 | | | 1.27E-05 | | 0.645152 | | 1.63E-05 | |
| 493 KCTD5 | | | 0.650926 | | | 1.29E-05 | | 0.642373 | | 1.82E-05 | |
| 494 QPCTL | | | 0.650604 | | | 1.30E-05 | | 0.62085 | | 4.13E-05 | |
| 495 SATL1 | | | 0.650563 | | | 1.31E-05 | | 0.644542 | | 1.67E-05 | |
| 496 TMCC1 | | | 0.650456 | | | 1.31E-05 | | 0.665893 | | 6.86E-06 | |
| 497 POP4 | | | 0.649536 | | | 1.36E-05 | | 0.662173 | | 8.05E-06 | |
| 498 FLJ21687 | | | 0.64929 | | | 1.38E-05 | | 0.661976 | | 8.12E-06 | |
| 499 LOC493869 | | | 0.649194 | | | 1.38E-05 | | 0.588408 | | 0.000128 | |
| 500 NPM3 | | | 0.649171 | | | 1.38E-05 | | 0.675753 | | 4.45E-06 | |
| 501 FLJ40142 | | | 0.648322 | | | 1.43E-05 | | 0.613904 | | 5.32E-05 | |
| 502 NIT1 | | | 0.64809 | | | 1.45E-05 | | 0.66072 | | 8.56E-06 | |
| 503 RNF113A | | | 0.647984 | | | 1.45E-05 | | 0.637922 | | 2.17E-05 | |
| 504 POLG | | | 0.645795 | | | 1.59E-05 | | 0.633675 | | 2.55E-05 | |
| 505 COMMD6 | | | 0.645286 | | | 1.62E-05 | | 0.628136 | | 3.15E-05 | |
| 506 C5ORF16 | | | 0.64417 | | | 1.69E-05 | | 0.657511 | | 9.80E-06 | |
| 507 KIAA1600 | | | 0.644002 | | | 1.70E-05 | | 0.614121 | | 5.28E-05 | |
| 508 ADHFE1 | | | 0.643715 | | | 1.72E-05 | | 0.624259 | | 3.64E-05 | |
| 509 C22ORF9 | | | 0.641708 | | | 1.87E-05 | | 0.671972 | | 5.26E-06 | |
| 510 C17ORF79 | | | 0.640436 | | | 1.96E-05 | | 0.656333 | | 1.03E-05 | |
| 511 LAD1 | | | 0.639112 | | | 2.07E-05 | | 0.618292 | | 4.54E-05 | |
| 512 LASS2 | | | 0.638745 | | | 2.10E-05 | | 0.583777 | | 0.000149 | |
| 513 TMEM93 | | | 0.638728 | | | 2.10E-05 | | 0.62683 | | 3.31E-05 | |
| 514 SENP3 | | | 0.638229 | | | 2.14E-05 | | 0.664495 | | 7.29E-06 | |
| 515 GATAD1 | | | 0.638208 | | | 2.14E-05 | | 0.590036 | | 0.000121 | |
| 516 C1ORF26 | | | 0.63792 | | | 2.17E-05 | | 0.621073 | | 4.10E-05 | |
| 517 ZNF121 | | | 0.63725 | | | 2.22E-05 | | 0.588899 | | 0.000126 | |
| 518 GORASP2 | | | 0.634887 | | | 2.44E-05 | | 0.540292 | | 0.000557 | |
| 519 CAMKK1 | | | 0.63441 | | | 2.48E-05 | | 0.612387 | | 5.62E-05 | |
| 520 FLJ46072 | | | 0.634308 | | | 2.49E-05 | | 0.652505 | | 1.21E-05 | |
| 521 VPS41 | | | 0.633974 | | | 2.52E-05 | | 0.583937 | | 0.000148 | |
| 522 PQLC3 | | | 0.633733 | | | 2.55E-05 | | 0.599064 | | 8.95E-05 | |
| 523 FLJ20628 | | | 0.633363 | | | 2.58E-05 | | 0.650441 | | 1.31E-05 | |
| 524 MGC33887 | | | 0.629681 | | | 2.97E-05 | | 0.634349 | | 2.49E-05 | |
| 525 C11ORF47 | | | 0.629485 | | | 2.99E-05 | | 0.614938 | | 5.13E-05 | |
| 526 DLAT | | | 0.629194 | | | 3.03E-05 | | 0.526504 | | 0.000816 | |
| 527 GALNT14 | | | 0.627022 | | | 3.29E-05 | | 0.593128 | | 0.000109 | |
| 528 FNTA | | | 0.626342 | | | 3.37E-05 | | 0.568288 | | 0.000243 | |
| 529 CDH2 | | | 0.625251 | | | 3.51E-05 | | 0.573316 | | 0.000208 | |
| 530 PRNP | | | 0.624871 | | | 3.56E-05 | | 0.546189 | | 0.00047 | |
| 531 EIF2B1 | | | 0.624825 | | | 3.57E-05 | | 0.589159 | | 0.000125 | |
| 532 SLC36A1 | | | 0.624714 | | | 3.58E-05 | | 0.578227 | | 0.000178 | |
| 533 AHSA2 | | | 0.624477 | | | 3.62E-05 | | 0.609175 | | 6.30E-05 | |
| 534 BDP1 | | | 0.624136 | | | 3.66E-05 | | 0.594148 | | 0.000106 | |
| 535 TCEB3 | | | 0.623736 | | | 3.72E-05 | | 0.579556 | | 0.000171 | |
| 536 DGKE | | | 0.623517 | | | 3.75E-05 | | 0.603471 | | 7.69E-05 | |
| 537 OR11L1 | | | 0.623099 | | | 3.80E-05 | | 0.627982 | | 3.17E-05 | |
| 538 POLR2J2 | | | 0.623019 | | | 3.82E-05 | | 0.621966 | | 3.97E-05 | |
| 539 HK2 | | | 0.622671 | | | 3.87E-05 | | 0.600615 | | 8.49E-05 | |
| 540 NDN | | | 0.620182 | | | 4.24E-05 | | 0.689154 | | 2.40E-06 | |
| 541 VPS13A | | | 0.619965 | | | 4.27E-05 | | 0.58348 | | 0.00015 | |
| 542 PAOX | | | 0.619216 | | | 4.39E-05 | | 0.604352 | | 7.46E-05 | |
| 543 LRP1 | | | 0.617928 | | | 4.60E-05 | | 0.640749 | | 1.94E-05 | |
| 544 C16ORF57 | | | 0.617792 | | | 4.62E-05 | | 0.630397 | | 2.89E-05 | |
| 545 DPAGT1 | | | 0.617399 | | | 4.69E-05 | | 0.569556 | | 0.000234 | |
| 546 EGFL7 | | | 0.617166 | | | 4.73E-05 | | 0.649815 | | 1.35E-05 | |
| 547 FLJ21106 | | | 0.616002 | | | 4.93E-05 | | 0.644741 | | 1.65E-05 | |
| 548 MCPH1 | | | 0.615937 | | | 4.95E-05 | | 0.626189 | | 3.39E-05 | |
| 549 CYP2R1 | | | 0.61567 | | 4.99E-05 | | | 0.647128 | | 1.50E-05 | |
| 550 MAP1LC3B | | | 0.615449 | | 5.03E-05 | | | 0.58658 | | 0.000136 | |
| 551 CXCL16 | | | 0.615204 | | 5.08E-05 | | | 0.637134 | | 2.23E-05 | |
| 552 DNCL2A | | | 0.612587 | | 5.58E-05 | | | 0.683493 | | 3.13E-06 | |
| 553 C20ORF23 | | | 0.611773 | | 5.74E-05 | | | 0.645621 | | 1.60E-05 | |
| 554 RNF141 | | | 0.611555 | | 5.79E-05 | | | 0.530928 | | 0.000723 | |
| 555 CEP63 | | | 0.610948 | | 5.91E-05 | | | 0.591426 | | 0.000116 | |
| 556 CST9 | | | 0.609765 | | 6.17E-05 | | | 0.585121 | | 0.000143 | |
| 557 TMSB4X | | | 0.609668 | | 6.19E-05 | | | 0.650212 | | 1.33E-05 | |
| 558 NDUFB10 | | | 0.609617 | | 6.20E-05 | | | 0.641108 | | 1.91E-05 | |
| 559 MSL3L1 | | | 0.6091 | | 6.31E-05 | | | 0.668204 | | 6.21E-06 | |
| 560 ADAM12 | | | 0.608831 | | 6.38E-05 | | | 0.63732 | | 2.22E-05 | |
| 561 C10ORF89 | | | 0.608711 | | 6.40E-05 | | | 0.638177 | | 2.14E-05 | |
| 562 CCIN | | | 0.608102 | | 6.54E-05 | | | 0.611014 | | 5.90E-05 | |
| 563 CEACAM3 | | | 0.607575 | | 6.66E-05 | | | 0.592272 | | 0.000113 | |
| 564 CHST6 | | | 0.606306 | | 6.97E-05 | | | 0.667117 | | 6.51E-06 | |
| 565 ASB3 | | | 0.605866 | | 7.07E-05 | | | 0.645644 | | 1.59E-05 | |
| 566 PHB | | | 0.605472 | | 7.17E-05 | | | 0.560676 | | 0.000307 | |
| 567 AFAP1L2 | | | 0.605451 | | 7.18E-05 | | | 0.603709 | | 7.63E-05 | |
| 568 SUCLG1 | | | 0.605124 | | 7.26E-05 | | | 0.687747 | | 2.56E-06 | |
| 569 SCAMP3 | | | 0.604722 | | 7.36E-05 | | | 0.639351 | | 2.05E-05 | |
| 570 ATG4A | | | 0.604107 | | 7.52E-05 | | | 0.625437 | | 3.49E-05 | |
| 571 SKP1A | | | 0.603646 | | 7.64E-05 | | | 0.602409 | | 7.98E-05 | |
| 572 C17ORF37 | | | 0.600811 | | 8.43E-05 | | | 0.626252 | | 3.38E-05 | |
| 573 PSMA3 | | | 0.59905 | | 8.95E-05 | | | 0.589469 | | 0.000124 | |
| 574 QSCN6 | | | 0.598776 | | 9.04E-05 | | | 0.615012 | | 5.11E-05 | |
| 575 TMEM183B | | | 0.597366 | | 9.48E-05 | | | 0.568665 | | 0.00024 | |
| 576 FLYWCH1 | | | 0.597336 | | 9.49E-05 | | | 0.59605 | | 9.92E-05 | |
| 577 SNX1 | | | 0.597006 | | 9.60E-05 | | | 0.543006 | | 0.000515 | |
| 578 C1ORF162 | | | 0.596119 | | 9.89E-05 | | | 0.594276 | | 0.000105 | |
| 579 PRX | | | 0.594786 | | 0.000103 | | | 0.581624 | | 0.00016 | |
| 580 SLC45A1 | | | 0.594613 | | 0.000104 | | | 0.542222 | | 0.000527 | |
| 581 TNFRSF13C | | | 0.592902 | | 0.00011 | | | 0.600654 | | 8.48E-05 | |
| 582 MYOCD | | | 0.592585 | | 0.000111 | | | 0.622752 | | 3.85E-05 | |
| 583 OAZ1 | | | 0.591149 | | 0.000117 | | | 0.596847 | | 9.65E-05 | |
| 584 CDC42EP5 | | | 0.588068 | | 0.000129 | | | 0.682185 | | 3.32E-06 | |
| 585 POLR3D | | | 0.587066 | | 0.000134 | | | 0.593129 | | 0.000109 | |
| 586 DDX1 | | | 0.585023 | | 0.000143 | | | 0.574241 | | 0.000202 | |
| 587 PTHR1 | | | 0.584155 | | 0.000147 | | | 0.611161 | | 5.87E-05 | |
| 588 WDR47 | | | 0.582937 | | 0.000153 | | | 0.621431 | | 4.05E-05 | |
| 589 SNAPAP | | | 0.580588 | | 0.000165 | | | 0.592223 | | 0.000113 | |
| 590 DGKI | | | 0.580329 | | 0.000166 | | | 0.481315 | | 0.002562 | |
| 591 STAP2 | | | 0.580183 | | 0.000167 | | | 0.632717 | | 2.65E-05 | |
| 592 ATP5C1 | | | 0.578173 | | 0.000178 | | | 0.615739 | | 4.98E-05 | |
| 593 RUFY2 | | | 0.576899 | | 0.000186 | | | 0.516832 | | 0.001056 | |
| 594 NAG | | | 0.576797 | | 0.000186 | | | 0.596986 | | 9.61E-05 | |
| 595 SNX25 | | | 0.574412 | | 0.000201 | | | 0.515997 | | 0.00108 | |
| 596 ROBO4 | | | 0.573816 | | 0.000205 | | | 0.534951 | | 0.000647 | |
| 597 OR2H2 | | | 0.573531 | | 0.000207 | | | 0.595659 | | 0.0001 | |
| 598 HACE1 | | | 0.571956 | | 0.000217 | | | 0.552026 | | 0.000397 | |
| 599 STON1 | | | 0.571851 0.000218 0.603206 7.76E-05 | | | | | | | | |
| 600 PLAA | | | 0.570312 0.000228 0.587341 0.000133 | | | | | | | | |
| 601 STX8 | | | 0.569894 0.000231 0.568044 0.000245 | | | | | | | | |
| 602 HSD17B1 | | | 0.569489 0.000234 0.620161 4.24E-05 | | | | | | | | |
| 603 TMCO6 | | | 0.568178 0.000244 0.595869 9.98E-05 | | | | | | | | |
| 604 ANKRD23 | | | 0.567268 0.000251 0.591621 0.000115 | | | | | | | | |
| 605 RABEP1 | | | 0.566773 0.000255 0.475631 0.002928 | | | | | | | | |
| 606 FLJ20125 | | | 0.566706 0.000255 0.531596 0.00071 | | | | | | | | |
| 607 SET7 | | | 0.566353 0.000258 0.538356 0.000588 | | | | | | | | |
| 608 KREMEN1 | | | 0.564564 0.000273 0.595634 0.000101 | | | | | | | | |
| 609 LHX5 | | | 0.564096 0.000277 0.536104 0.000626 | | | | | | | | |
| 610 ANKRD27 | | | 0.562159 0.000293 0.57242 0.000214 | | | | | | | | |
| 611 TBC1D21 | | | 0.560824 0.000306 0.514032 0.001136 | | | | | | | | |
| 612 CLCN5 | | | 0.560049 0.000313 0.548881 0.000435 | | | | | | | | |
| 613 MGC35361 | | | 0.557759 0.000335 0.5033 0.001497 | | | | | | | | |
| 614 MAPK14 | | | 0.556763 0.000345 0.541068 0.000545 | | | | | | | | |
| 615 STAC3 | | | 0.556556 0.000347 0.52883 0.000766 | | | | | | | | |
| 616 EPS15L1 | | | 0.556365 0.000349 0.586003 0.000139 | | | | | | | | |
| 617 MMP24 | | | 0.555516 0.000358 0.607285 6.73E-05 | | | | | | | | |
| 618 SLC30A9 | | | 0.554843 0.000365 0.47558 0.002931 | | | | | | | | |
| 619 NOL7 | | | 0.554832 0.000366 0.487238 0.002225 | | | | | | | | |
| 620 PDK3 | | | 0.553169 0.000384 0.553074 0.000385 | | | | | | | | |
| 621 NCK1 | | | 0.552787 0.000388 0.558278 0.00033 | | | | | | | | |
| 622 CARS | | | 0.551362 0.000405 0.489473 0.002108 | | | | | | | | |
| 623 SRP46 | | | 0.550656 0.000413 0.540316 0.000556 | | | | | | | | |
| 624 PPIL6 | | | 0.549447 0.000428 0.504143 0.001465 | | | | | | | | |
| 625 C9ORF150 | | | 0.547789 0.000449 0.504535 0.001451 | | | | | | | | |
| 626 ARVCF | | | 0.546605 0.000465 0.549863 0.000423 | | | | | | | | |
| 627 MAB21L1 | | | 0.54547 0.00048 0.498805 0.001676 | | | | | | | | |
| 628 C1ORF149 | | | 0.545446 0.000481 0.503228 0.0015 | | | | | | | | |
| 629 PCYT1A | | | 0.541891 0.000532 0.526316 0.00082 | | | | | | | | |
| 630 PPP4R1L | | | 0.541891 0.000532 0.556941 0.000343 | | | | | | | | |
| 631 C6ORF136 | | | 0.541828 0.000533 0.589898 0.000122 | | | | | | | | |
| 632 C11ORF54 | | | 0.539988 0.000562 0.536894 0.000613 | | | | | | | | |
| 633 NAB1 | | | 0.537856 0.000596 0.474621 0.002997 | | | | | | | | |
| 634 LSM12 | | | 0.537082 0.000609 0.495078 0.001838 | | | | | | | | |
| 635 UNC13B | | | 0.536409 0.000621 0.545964 0.000474 | | | | | | | | |
| 636 CRYBB2 | | | 0.535201 0.000642 0.498244 0.001699 | | | | | | | | |
| 637 TSCOT | | | 0.53501 0.000646 0.5476 0.000452 | | | | | | | | |
| 638 CCL1 | | | 0.534995 0.000646 0.531046 0.000721 | | | | | | | | |
| 639 TARDBP | | | 0.533487 0.000674 0.46833 0.003463 | | | | | | | | |
| 640 RB1CC1 | | | 0.531844 0.000705 0.536246 0.000624 | | | | | | | | |
| 641 MAK10 | | | 0.530859 0.000724 0.52257 0.000907 | | | | | | | | |
| 642 FLJ38288 | | | 0.530306 0.000735 0.524676 0.000857 | | | | | | | | |
| 643 SEMG1 | | | 0.529885 0.000744 0.539225 0.000574 | | | | | | | | |
| 644 UNQ830 | | | 0.5295 0.000752 0.511785 0.001205 | | | | | | | | |
| 645 PTPN6 | | | 0.527622 0.000791 0.512098 0.001195 | | | | | | | | |
| 646 CCL23 | | | 0.527552 0.000793 0.513798 0.001143 | | | | | | | | |
| 647 C12ORF11 | | | 0.527246 0.000799 0.518113 0.001021 | | | | | | | | |
| 648 ZNF418 | | | 0.527003 0.000805 0.567034 0.000253 | | | | | | | | |
| 649 CLIC2 | | | 0.525708 0.000833 0.502488 0.001528 | | | | | | | | |
| 650 FAHD1 | | | 0.525583 0.000836 0.504129 0.001466 | | | | | | | | |
| 651 CREBL2 | | | 0.52527 0.000843 0.50003 0.001625 | | | | | | | | |
| 652 CAMTA1 | | | 0.524124 0.00087 0.543049 0.000515 | | | | | | | | |
| 653 AMIGO2 | | | 0.523568 0.000883 0.538525 0.000585 | | | | | | | | |
| 654 LEMD2 | | | 0.522353 0.000912 0.481506 0.002551 | | | | | | | | |
| 655 EPM2A | | | 0.521456 0.000934 0.469008 0.00341 | | | | | | | | |
| 656 NSUN5B | | | 0.520472 0.000959 0.506569 0.001378 | | | | | | | | |
| 657 ADPN | | | 0.519654 0.00098 0.541112 0.000544 | | | | | | | | |
| 658 AK1 | | | 0.519609 0.000981 0.581501 0.00016 | | | | | | | | |
| 659 MASS1 | | | 0.51958 0.000982 0.530974 0.000722 | | | | | | | | |
| 660 PAX7 | | | 0.51924 0.000991 0.546484 0.000466 | | | | | | | | |
| 661 BID | | | 0.518105 0.001021 0.510533 0.001245 | | | | | | | | |
| 662 ADORA3 | | | 0.516739 0.001059 0.495926 0.0018 | | | | | | | | |
| 663 TRY1 | | | 0.516384 0.001069 0.500161 0.00162 | | | | | | | | |
| 664 NAGS | | | 0.511507 0.001214 0.524524 0.00086 | | | | | | | | |
| 665 LOC653906 | | | 0.511034 0.001229 0.487028 0.002236 | | | | | | | | |
| 666 C1ORF37 | | | 0.510677 0.00124 0.48053 0.00261 | | | | | | | | |
| 667 PAPOLG | | | 0.509454 0.00128 0.501646 0.001561 | | | | | | | | |
| 668 GSTP1 | | | 0.508857 0.001299 0.578028 0.000179 | | | | | | | | |
| 669 H2AFY2 | | | 0.506003 0.001398 0.52712 0.000802 | | | | | | | | |
| 670 FLJ20534 | | | 0.50514 0.001429 0.516355 0.001069 | | | | | | | | |
| 671 TFB1M | | | 0.504967 0.001435 0.539101 0.000576 | | | | | | | | |
| 672 OVGP1 | | | 0.504027 0.00147 0.433116 0.007414 | | | | | | | | |
| 673 SOX14 | | | 0.503717 0.001481 | | | | | 0.525414 0.00084 | | | |
| 674 PPIE | | | 0.500937 0.001589 | | | | | 0.489113 0.002126 | | | |
| 675 LOC389118 | | | 0.500497 0.001606 | | | | | 0.521148 0.000942 | | | |
| 676 MTX3 | | | 0.498673 0.001681 | | | | | 0.446496 0.005603 | | | |
| 677 WDR4 | | | 0.498557 0.001686 | | | | | 0.533374 0.000676 | | | |
| 678 EPB41L1 | | | 0.4947 0.001855 | | | | | 0.530855 0.000724 | | | |
| 679 FAM26B | | | 0.494385 0.00187 | | | | | 0.45585 0.004577 | | | |
| 680 LYSMD3 | | | 0.493031 0.001933 | | | | | 0.427698 0.008278 | | | |
| 681 NRIP2 | | | 0.491005 0.002031 | | | | | 0.511758 0.001206 | | | |
| 682 PRSS35 | | | 0.488636 0.002151 | | | | | 0.484691 0.002365 | | | |
| 683 FLJ16734 | | | 0.487004 0.002237 | | | | | 0.499322 0.001654 | | | |
| 684 AGPAT6 | | | 0.485929 0.002296 | | | | | 0.482794 0.002474 | | | |
| 685 INPP5F | | | 0.48291 0.002467 | | | | | 0.39366 0.015927 | | | |
| 686 MRPL22 | | | 0.482119 0.002514 | | | | | 0.505952 0.0014 | | | |
| 687 BAG4 | | | 0.480171 0.002632 | | | | | 0.433015 0.007429 | | | |
| 688 ESM1 | | | 0.478683 0.002726 | | | | | 0.506694 0.001373 | | | |
| 689 PPP1R11 | | | 0.477038 0.002833 | | | | | 0.463955 0.003823 | | | |
| 690 KIAA1970 | | | 0.476968 0.002838 | | | | | 0.377527 0.021247 | | | |
| 691 C1S | | | 0.476813 0.002848 | | | | | 0.492439 0.001961 | | | |
| 692 ZNF451 | | | 0.475639 0.002927 | | | | | 0.454501 0.004714 | | | |
| 693 FLJ30058 | | | 0.475608 0.002929 | | | | | 0.489752 0.002093 | | | |
| 694 DEFB1 | | | 0.47559 0.00293 0.436525 0.00691 | | | | | | | | |
| 695 CDYL | | | 0.47534 0.002948 0.396076 0.015236 | | | | | | | | |
| 696 KLK1 | | | 0.475209 0.002957 0.504719 0.001444 | | | | | | | | |
| 697 NSMAF | | | 0.474075 0.003035 0.376154 0.021761 | | | | | | | | |
| 698 LRCH2 | | | 0.472711 0.003132 0.490604 0.002051 | | | | | | | | |
| 699 ANAPC11 | | | 0.472162 0.003172 0.53884 0.00058 | | | | | | | | |
| 700 OR51T1 | | | 0.472003 0.003184 0.436533 0.006909 | | | | | | | | |
| 701 DKFZP686I15217 | | | 0.469565 0.003367 0.473671 0.003064 | | | | | | | | |
| 702 C19ORF18 | | | 0.466315 0.003625 0.424549 0.008819 | | | | | | | | |
| 703 RG9MTD2 | | | 0.463637 0.003851 0.448102 0.005414 | | | | | | | | |
| 704 FLJ22662 | | | 0.462987 0.003907 0.502371 0.001533 | | | | | | | | |
| 705 DNASE1 | | | 0.461744 0.004017 0.429705 0.007948 | | | | | | | | |
| 706 EVC | | | 0.460961 0.004088 0.451764 0.005003 | | | | | | | | |
| 707 ATP1B2 | | | 0.460396 0.00414 0.452302 0.004945 | | | | | | | | |
| 708 TNFSF8 | | | 0.459762 0.004198 0.446269 0.00563 | | | | | | | | |
| 709 S100A5 | | | 0.456688 0.004493 0.451832 0.004996 | | | | | | | | |
| 710 TRIM15 | | | 0.456645 0.004497 0.424771 0.00878 | | | | | | | | |
| 711 BECN1 | | | 0.454584 0.004705 0.396867 0.015015 | | | | | | | | |
| 712 SRA1 | | | 0.4543 0.004735 0.42646 0.008487 | | | | | | | | |
| 713 RPL39 | | | 0.452051 0.004972 0.460881 0.004095 | | | | | | | | |
| 714 VGCNL1 | | | 0.451302 0.005053 0.370942 0.023807 | | | | | | | | |
| 715 C17ORF25 | | | 0.450263 0.005168 0.442032 0.006159 | | | | | | | | |
| 716 SLC28A1 | | | 0.449707 0.00523 0.484801 0.002359 | | | | | | | | |
| 717 KCNK9 | | | 0.448989 0.005312 0.426825 0.008425 | | | | | | | | |
| 718 CLEC3B | | | 0.448518 0.005366 0.384183 0.018896 | | | | | | | | |
| 719 PLCH2 | | | 0.447715 0.005459 0.44235 0.006118 | | | | | | | | |
| 720 ST6GALNAC6 | | | 0.44759 0.005474 0.500334 0.001613 | | | | | | | | |
| 721 EXPH5 | | | 0.446935 0.005551 0.373865 0.022641 | | | | | | | | |
| 722 NPL | | | 0.44687 0.005559 0.401794 0.013701 | | | | | | | | |
| 723 NR1I2 | | | 0.445614 0.00571 0.456833 0.004479 | | | | | | | | |
| 724 FGF13 | | | 0.444205 0.005883 0.440198 0.006401 | | | | | | | | |
| 725 SRD5A2 | | | 0.439229 0.006532 0.4225 0.009187 | | | | | | | | |
| 726 CDC42 | | | 0.437743 0.006738 0.441544 0.006223 | | | | | | | | |
| 727 FLJ33718 | | | 0.43628 0.006946 0.430467 0.007826 | | | | | | | | |
| 728 C12ORF30 | | | 0.435911 0.006999 0.446615 0.005589 | | | | | | | | |
| 729 GALNT12 | | | 0.431827 0.007612 0.372933 0.023008 | | | | | | | | |
| 730 MOCOS | | | 0.431069 0.007731 0.444671 0.005825 | | | | | | | | |
| 731 ORMDL3 | | | 0.430725 0.007785 0.469009 0.00341 | | | | | | | | |
| 732 CHD2 | | | 0.4286 0.008128 0.352924 0.032149 | | | | | | | | |
| 733 C19ORF19 | | | 0.426892 0.008414 0.369724 0.024308 | | | | | | | | |
| 734 WNK3 | | | 0.424386 0.008848 0.412738 0.011125 | | | | | | | | |
| 735 CAPN7 | | | 0.423944 0.008926 0.41563 0.010518 | | | | | | | | |
| 736 FLJ36492 | | | 0.421931 0.009292 0.40997 0.011735 | | | | | | | | |
| 737 ACP6 | | | 0.419821 0.009688 0.413455 0.010972 | | | | | | | | |
| 738 KIF3A | | | 0.419166 0.009814 0.400169 0.014123 | | | | | | | | |
| 739 LOC401589 | | | 0.418949 0.009856 0.404729 0.012965 | | | | | | | | |
| 740 SLC6A7 | | | 0.418233 0.009996 0.420349 0.009587 | | | | | | | | |
| 741 UVRAG | | | 0.414341 0.010785 0.363126 0.027174 | | | | | | | | |
| 742 SCUBE2 | | | 0.411813 0.011326 0.420443 0.00957 | | | | | | | | |
| 743 MGAT5 | | | 0.411459 0.011404 0.43981 0.006454 | | | | | | | | |
| 744 MGC119295 | | | 0.409506 0.011839 0.400724 0.013978 | | | | | | | | |
| 745 ARFIP2 | | | 0.403583 0.013248 0.424971 0.008745 | | | | | | | | |
| 746 ABCC12 | | | 0.403128 0.013363 0.400076 0.014148 | | | | | | | | |
| 747 RNF39 | | | 0.400395 0.014064 0.420592 0.009542 | | | | | | | | |
| 748 POMC | | | 0.400245 0.014103 0.412946 0.011081 | | | | | | | | |
| 749 AQP9 | | | 0.399859 0.014205 0.381798 0.019712 | | | | | | | | |
| 750 C14ORF150 | | | 0.39865 0.014528 0.405535 0.012769 | | | | | | | | |
| 751 LOC644685 | | | 0.398435 0.014586 0.379695 0.020456 | | | | | | | | |
| 752 U2AF1L2 | | | 0.397947 0.014719 0.438703 0.006604 | | | | | | | | |
| 753 NFIB | | | 0.395781 0.015319 0.3703 0.02407 | | | | | | | | |
| 754 TFEB | | | 0.39539 0.015429 0.396111 0.015226 | | | | | | | | |
| 755 NKX2-8 | | | 0.395386 0.015431 0.412743 0.011124 | | | | | | | | |
| 756 C1ORF89 | | | 0.395361 0.015438 0.439676 0.006472 | | | | | | | | |
| 757 MAPK10 | | | 0.395063 0.015523 0.390084 0.016998 | | | | | | | | |
| 758 CECR1 | | | 0.391077 0.016694 0.427923 0.008241 | | | | | | | | |
| 759 SPTBN2 | | | 0.390905 0.016746 0.415177 0.010611 | | | | | | | | |
| 760 PLEK2 | | | 0.387087 0.017941 0.37158 0.023549 | | | | | | | | |
| 761 FOXD2 | | | 0.385979 0.0183 0.376428 0.021657 | | | | | | | | |
| 762 VAMP4 | | | 0.385901 0.018326 0.378307 0.020959 | | | | | | | | |
| 763 PACSIN1 | | | 0.381614 0.019776 0.42413 0.008893 | | | | | | | | |
| 764 GTF2E1 | | | 0.381203 0.01992 0.362558 0.027433 | | | | | | | | |
| 765 PIGK | | | 0.380737 0.020084 0.326803 0.048357 | | | | | | | | |
| 766 RAPGEF4 | | | 0.38047 0.020179 0.354504 0.031333 | | | | | | | | |
| 767 POLH | | | 0.379551 0.020507 0.383131 0.019252 | | | | | | | | |
| 768 FLJ45422 | | | 0.379002 0.020706 0.373718 0.022698 | | | | | | | | |
| 769 SAA4 | | | 0.378658 0.020831 0.379104 0.020669 | | | | | | | | |
| 770 DUSP13 | | | 0.378523 0.02088 0.339029 0.040103 | | | | | | | | |
| 771 OR12D2 | | | 0.37435 0.022452 0.343784 0.037218 | | | | | | | | |
| 772 TREML2P | | | 0.373981 0.022596 0.378809 0.020776 | | | | | | | | |
| 773 GPR156 | | | 0.372413 0.023214 0.353415 0.031894 | | | | | | | | |
| 774 C5AR1 | | | 0.370587 0.023953 0.382032 0.019631 | | | | | | | | |
| 775 FOXO1A | | | 0.365465 0.026127 0.354176 0.031501 | | | | | | | | |
| 776 TAOK1 | | | 0.363432 0.027035 0.385169 0.018567 | | | | | | | | |
| 777 DACT2 | | | 0.361874 0.027748 0.381796 0.019712 | | | | | | | | |
| 778 FREM1 | | | 0.361422 0.027957 0.424373 0.00885 | | | | | | | | |
| 779 BAZ2A | | | 0.361074 0.02812 0.34737 0.035157 | | | | | | | | |
| 780 PHKA1 | | | 0.359544 0.028842 0.369971 0.024206 | | | | | | | | |
| 781 ST6GALNAC2 | | | 0.359353 0.028934 0.348243 0.03467 | | | | | | | | |
| 782 HIRA | | | 0.357529 0.029818 0.368975 0.02462 | | | | | | | | |
| 783 TRIM29 | | | 0.355078 0.031041 0.373748 0.022687 | | | | | | | | |
| 784 WNT10A | | | 0.354077 0.031552 0.383266 0.019206 | | | | | | | | |
| 785 RNF36 | | | 0.353031 0.032093 0.351898 0.032689 | | | | | | | | |
| 786 C1ORF101 | | | 0.347912 0.034854 0.336148 0.041937 | | | | | | | | |
| 787 PVRL4 | | | 0.347592 0.035033 0.337984 0.04076 | | | | | | | | |
| 788 TRH | | | 0.339476 0.039824 0.382624 0.019426 | | | | | | | | |
| 789 HHLA1 | | | 0.338114 0.040678 0.337862 0.040838 | | | | | | | | |
| 790 OR4P4 | | | 0.331135 0.045289 0.399017 0.014429 | | | | | | | | |
| 791 PCTK2 | | | 0.327609 0.047773 0.330366 0.045822 | | | | | | | | |
| 792 PRKACA | | | 0.326772 0.04838 0.379489 0.02053 | | | | | | | | |
| 793 IFNK | | | 0.326487 0.048587 0.335849 0.042131 | | | | | | | | |
