# Supplementary material for: Identification of SCARA3 with potential roles in metabolic disorders
Source: Aging (Albany NY). 2020 Dec 9;13(2):2149–67. doi: 10.18632/aging.202228 (PMC7880357; doi:10.18632/aging.202228)
Supplement: Supplementary Table 5 [file aging-13-202228-s006.docx]

**Supplementary Table 5: DEGs during adipogenesis from GEO100748. (Adjust p-value <=0.05, |logFC|>1**

| GENE | logFC | | AveExpr | | t | | P.Value | | | adj.P.Val | | | B | | |
| --- | --- | --- | --- | --- | --- | --- | --- | --- | --- | --- | --- | --- | --- | --- | --- |
| AXUD1 | -4.33069 | | 10.52634 | | -56.1431 | | 1.86E-27 | | | 3.20E-23 | | | 49.87099 | | |
| ANGPTL4 | -4.6687 | | 8.143143 | | -36.1398 | | 8.18E-23 | | | 7.05E-19 | | | 41.37265 | | |
| TRIB1 | -2.93443 | | 10.16353 | | -33.7781 | | 4.17E-22 | | | 2.39E-18 | | | 39.94232 | | |
| BHLHB2 | -2.5658 | | 12.23128 | | -31.2581 | | 2.68E-21 | | | 1.16E-17 | | | 38.27212 | | |
| ID3 | -3.79137 | | 10.57164 | | -29.292 | | 1.27E-20 | | | 4.38E-17 | | | 36.85214 | | |
| JUNB | -4.14459 | | 8.182956 | | -28.827 | | 1.86E-20 | | | 5.36E-17 | | | 36.49983 | | |
| H3F3B | -2.73766 | | 8.991603 | | -28.4052 | | 2.65E-20 | | | 6.53E-17 | | | 36.17456 | | |
| CPA4 | 6.409645 | | 6.247161 | | 27.96781 | | 3.84E-20 | | | 8.27E-17 | | | 35.83127 | | |
| CBS | 2.832345 | | 8.691056 | | 27.00108 | | 8.87E-20 | | | 1.70E-16 | | | 35.05031 | | |
| PPP1R15A | -2.75575 | | 11.11101 | | -26.7598 | | 1.10E-19 | | | 1.89E-16 | | | 34.85041 | | |
| EFHD1 | 3.29162 | | 8.4696 | | 26.25782 | | 1.72E-19 | | | 2.70E-16 | | | 34.42808 | | |
| REC8L1 | -2.66205 | | 6.97136 | | -25.354 | | 3.95E-19 | | | 5.68E-16 | | | 33.6444 | | |
| ZC3H12A | -3.77579 | | 8.289965 | | -24.6937 | | 7.38E-19 | | | 9.79E-16 | | | 33.05223 | | |
| SLC25A25 | -2.26738 | | 8.816494 | | -24.2696 | | 1.11E-18 | | | 1.37E-15 | | | 32.66275 | | |
| CREG1 | 2.085429 | | 8.855997 | | 23.32775 | | 2.83E-18 | | | 3.25E-15 | | | 31.77084 | | |
| IER3 | -2.80699 | | 11.95235 | | -22.782 | | 4.93E-18 | | | 5.32E-15 | | | 31.23628 | | |
| SPOCK | 2.683831 | | 8.676689 | | 22.19182 | | 9.14E-18 | | | 8.71E-15 | | | 30.64292 | | |
| CHST7 | 1.851812 | | 8.674693 | | 22.17256 | | 9.32E-18 | | | 8.71E-15 | | | 30.62327 | | |
| CGNL1 | 2.431638 | | 6.496332 | | 22.0276 | | 1.09E-17 | | | 8.71E-15 | | | 30.47487 | | |
| KIAA0644 | -2.89539 | | 8.052624 | | -22.0116 | | 1.11E-17 | | | 8.71E-15 | | | 30.4584 | | |
| KLF9 | -2.74677 | | 10.21481 | | -21.9771 | | 1.15E-17 | | | 8.71E-15 | | | 30.42289 | | |
| LOC91461 | -2.92942 | | 5.283105 | | -21.9657 | | 1.16E-17 | | | 8.71E-15 | | | 30.41118 | | |
| CDC20 | -5.25645 | | 7.028418 | | -21.9648 | | 1.16E-17 | | | 8.71E-15 | | | 30.41023 | | |
| HEY1 | -6.26955 | | 7.936847 | | -21.4614 | | 2.00E-17 | | | 1.38E-14 | | | 29.8854 | | |
| HEYL | -7.73686 | | 6.194541 | | -26.8809 | | 1.69E-17 | | | 1.21E-14 | | | 29.82315 | | |
| PSAT1 | 3.666027 | | 8.636994 | | 21.13096 | | 2.87E-17 | | | 1.90E-14 | | | 29.53392 | | |
| CDKN2B | 2.581463 | | 7.326803 | | 21.0579 | | 3.11E-17 | | | 1.99E-14 | | | 29.45546 | | |
| TGFBR3 | -1.58345 | | 6.974537 | | -20.6507 | | 4.91E-17 | | | 3.02E-14 | | | 29.01303 | | |
| EPSTI1 | -1.7706 | | 8.005814 | | -20.2712 | | 7.55E-17 | | | 4.49E-14 | | | 28.59271 | | |
| WISP1 | -2.44144 | | 7.704887 | | -20.2036 | | 8.16E-17 | | | 4.69E-14 | | | 28.51694 | | |
| C21orf7 | 3.119921 | | 8.21063 | | 19.84885 | | 1.23E-16 | | | 6.85E-14 | | | 28.11549 | | |
| CHRNA1 | 1.998786 | | 11.33657 | | 19.77608 | | 1.34E-16 | | | 7.22E-14 | | | 28.03225 | | |
| RUNX3 | -2.40901 | | 7.791719 | | -19.593 | | 1.66E-16 | | | 8.69E-14 | | | 27.82147 | | |
| MX1 | -3.79185 | | 8.379208 | | -19.4669 | | 1.93E-16 | | | 9.79E-14 | | | 27.67517 | | |
| LOX | 2.401344 | | 10.03875 | | 19.4376 | | 2.00E-16 | | | 9.85E-14 | | | 27.64107 | | |
| AGA | 1.444788 | | 9.660763 | | 19.39261 | | 2.11E-16 | | | 1.01E-13 | | | 27.58857 | | |
| FOSB | -8.70573 | | 8.528851 | | -19.2421 | | 2.53E-16 | | | 1.18E-13 | | | 27.41203 | | |
| COLEC12 | -3.64122 | | 9.725096 | | -19.1877 | | 2.70E-16 | | | 1.22E-13 | | | 27.34792 | | |
| LOC492311 | 3.134496 | | 6.887202 | | 19.10074 | | 2.99E-16 | | | 1.32E-13 | | | 27.24506 | | |
| FNDC6 | 2.747692 | | 6.447203 | | 18.88674 | | 3.88E-16 | | | 1.67E-13 | | | 26.98991 | | |
| EFNA1 | -4.98252 | | 7.734458 | | -18.8544 | | 4.04E-16 | | | 1.70E-13 | | | 26.95108 | | |
| CLDN1 | 4.074812 | | 8.418738 | | 18.71076 | | 4.82E-16 | | | 1.98E-13 | | | 26.778 | | |
| COMP | 4.788706 | | 6.674163 | | 18.56352 | | 5.78E-16 | | | 2.31E-13 | | | 26.5992 | | |
| PER2 | -2.43406 | | 7.298148 | | -18.4315 | | 6.80E-16 | | | 2.67E-13 | | | 26.43766 | | |
| CDC2 | -4.28409 | | 5.994638 | | -18.4094 | | 6.99E-16 | | | 2.68E-13 | | | 26.41054 | | |
| KIAA0907 | 1.395038 | | 7.861214 | | 18.3279 | | 7.74E-16 | | | 2.90E-13 | | | 26.31022 | | |
| CCNL1 | -2.94293 | | 9.949116 | | -18.1488 | | 9.70E-16 | | | 3.56E-13 | | | 26.0882 | | |
| F2RL1 | -2.4081 | | 9.370574 | | -17.9507 | | 1.25E-15 | | | 4.48E-13 | | | 25.84012 | | |
| PLCB1 | | -1.59926 | | 7.336389 | | -17.7075 | | | 1.70E-15 | | | 5.99E-13 | | 25.53197 | |
| CLSTN2 | | 2.430331 | | 5.89997 | | 17.62056 | | | 1.91E-15 | | | 6.57E-13 | | 25.42088 | |
| CRELD2 | | -1.42889 | | 10.81494 | | -17.5623 | | | 2.06E-15 | | | 6.95E-13 | | 25.34611 | |
| TRAK2 | | 1.657031 | | 9.795959 | | 17.43732 | | | 2.42E-15 | | | 7.79E-13 | | 25.185 | |
| UCK2 | | 1.498175 | | 9.346854 | | 17.43681 | | | 2.42E-15 | | | 7.79E-13 | | 25.18434 | |
| IFIT1 | | -3.17979 | | 7.764866 | | -17.4312 | | | 2.44E-15 | | | 7.79E-13 | | 25.17705 | |
| NXT1 | | -1.54021 | | 9.630426 | | -17.3426 | | | 2.74E-15 | | | 8.59E-13 | | 25.06221 | |
| PPIC | | 1.042096 | | 9.986981 | | 17.32391 | | | 2.81E-15 | | | 8.64E-13 | | 25.03784 | |
| ASS1 | | 4.211855 | | 6.583709 | | 17.26072 | | | 3.05E-15 | | | 9.18E-13 | | 24.95546 | |
| NXF1 | | -1.66531 | | 9.106462 | | -17.2498 | | | 3.10E-15 | | | 9.18E-13 | | 24.94116 | |
| ASS | | 4.645631 | | 7.345543 | | 17.23886 | | | 3.14E-15 | | | 9.18E-13 | | 24.92689 | |
| ANKRD1 | | 4.758471 | | 6.699004 | | 17.15214 | | | 3.52E-15 | | | 1.01E-12 | | 24.81324 | |
| REXO2 | | 1.060259 | | 11.99252 | | 17.12991 | | | 3.63E-15 | | | 1.03E-12 | | 24.78401 | |
| IDS | | 1.524552 | | 10.36382 | | 16.86224 | | | 5.19E-15 | | | 1.44E-12 | | 24.42938 | |
| TIAM2 | | -2.24739 | | 7.907589 | | -16.791 | | | 5.71E-15 | | | 1.56E-12 | | 24.33413 | |
| CCND2 | | 2.447434 | | 10.99052 | | 16.7297 | | | 6.21E-15 | | | 1.67E-12 | | 24.25185 | |
| BRPF1 | | -1.16355 | | 9.832475 | | -16.6801 | | | 6.64E-15 | | | 1.76E-12 | | 24.18515 | |
| PTGDS | | 3.689503 | | 6.16949 | | 16.58645 | | | 7.54E-15 | | | 1.97E-12 | | 24.05853 | |
| RAMP1 | | 2.243159 | | 8.642519 | | 16.55028 | | | 7.92E-15 | | | 2.01E-12 | | 24.00947 | |
| EIF1 | | -1.06938 | | 12.00565 | | -16.5483 | | | 7.94E-15 | | | 2.01E-12 | | 24.00678 | |
| ID1 | | -3.88222 | | 9.434781 | | -16.5143 | | | 8.32E-15 | | | 2.08E-12 | | 23.96051 | |
| ING1 | | -2.61094 | | 6.663777 | | -16.4735 | | | 8.80E-15 | | | 2.17E-12 | | 23.90495 | |
| CDCA5 | | -3.45363 | | 7.109614 | | -16.4257 | | | 9.40E-15 | | | 2.28E-12 | | 23.83977 | |
| CHMP1B | | -1.37782 | | 10.00211 | | -16.4021 | | | 9.71E-15 | | | 2.32E-12 | | 23.80743 | |
| KLHL15 | | -3.63779 | | 4.280641 | | -16.9754 | | | 1.03E-14 | | | 2.44E-12 | | 23.77738 | |
| EDG2 | | -1.40396 | | 11.09848 | | -16.3013 | | | 1.12E-14 | | | 2.60E-12 | | 23.66914 | |
| EXTL1 | | 3.451297 | | 5.865904 | | 16.24043 | | | 1.21E-14 | | | 2.79E-12 | | 23.58519 | |
| ADM2 | | 1.999575 | | 7.358349 | | 16.07771 | | | 1.52E-14 | | | 3.45E-12 | | 23.35943 | |
| FSTL3 | | 2.613058 | | 7.293431 | | 16.03107 | | | 1.63E-14 | | | 3.64E-12 | | 23.29435 | |
| RGC32 | | 2.361437 | | 11.90024 | | 15.95277 | | | 1.81E-14 | | | 4.01E-12 | | 23.18468 | |
| PCSK1N | | 4.261487 | | 5.772045 | | 16.46269 | | | 2.01E-14 | | | 4.39E-12 | | 23.11479 | |
| PRR11 | | -3.51415 | | 4.390738 | | -15.8314 | | | 2.15E-14 | | | 4.61E-12 | | 23.01376 | |
| MCM7 | | -2.81944 | | 8.972086 | | -15.8272 | | | 2.17E-14 | | | 4.61E-12 | | 23.00784 | |
| PTTG1 | | -2.26037 | | 9.620277 | | -15.8139 | | | 2.21E-14 | | | 4.64E-12 | | 22.98905 | |
| CTH | | 2.017614 | | 6.317776 | | 15.78469 | | | 2.30E-14 | | | 4.78E-12 | | 22.94766 | |
| PRRX1 | | -1.94897 | | 10.14288 | | -15.6797 | | | 2.67E-14 | | | 5.49E-12 | | 22.79842 | |
| PHACTR4 | | -1.02912 | | 9.520437 | | -15.6351 | | | 2.85E-14 | | | 5.71E-12 | | 22.73488 | |
| KIAA0746 | | 2.734784 | | 8.016158 | | 15.60075 | | | 2.99E-14 | | | 5.93E-12 | | 22.68568 | |
| ATF3 | | -2.75023 | | 7.123611 | | -15.5647 | | | 3.15E-14 | | | 6.18E-12 | | 22.63395 | |
| CDKAL1 | | -2.62325 | | 6.469196 | | -15.5232 | | | 3.35E-14 | | | 6.48E-12 | | 22.57436 | |
| C14orf43 | | -1.90488 | | 8.072215 | | -15.4882 | | | 3.52E-14 | | | 6.74E-12 | | 22.52402 | |
| HBEGF | | 2.432594 | | 8.364051 | | 15.33408 | | | 4.40E-14 | | | 8.27E-12 | | 22.30097 | |
| TYMS | | -3.63193 | | 7.942741 | | -15.3323 | | | 4.41E-14 | | | 8.27E-12 | | 22.29845 | |
| PIM1 | | -4.03813 | | 7.186827 | | -15.3139 | | | 4.53E-14 | | | 8.40E-12 | | 22.27167 | |
| ZFP36 | | -4.05272 | | 9.533959 | | -15.2362 | | | 5.08E-14 | | | 9.31E-12 | | 22.15826 | |
| PRC1 | | -3.53122 | | 7.673521 | | -15.2078 | | | 5.29E-14 | | | 9.61E-12 | | 22.11678 | |
| HMFN0839 | | -2.21152 | | 6.32566 | | -15.1389 | | | 5.86E-14 | | | 1.05E-11 | | 22.01561 | |
| KBTBD2 | | -1.45511 | | 8.977788 | | -15.1213 | | | 6.01E-14 | | | 1.07E-11 | | 21.98977 | |
| OIP5 | | -3.87431 | | 5.733223 | | -15.094 | | | 6.26E-14 | | | 1.10E-11 | | 21.94963 | |
| HCAP-G | | -4.15775 | | 5.290923 | | -15.046 | | | 6.72E-14 | | | 1.17E-11 | | 21.87866 | |
| KCNK2 | | -3.11557 | | 6.389122 | | -15.0244 | | | 6.94E-14 | | | 1.18E-11 | | 21.84679 | |
| RKHD3 | | -2.38031 | | 6.959264 | | -15.024 | | | 6.94E-14 | | | 1.18E-11 | | 21.84625 | |
| GUSB | | -1.40748 | | 8.369189 | | -14.9533 | | | 7.71E-14 | | | 1.30E-11 | | 21.7413 | |
| MGLL | | 1.838848 | | 9.077331 | | 14.92225 | | | 8.07E-14 | | | 1.35E-11 | | 21.69518 | |
| OSBPL10 | | 1.349777 | | 7.83003 | | 14.88192 | | | 8.57E-14 | | | 1.42E-11 | | 21.63508 | |
| TNFRSF19 | | -1.65415 | | 11.01059 | | -14.8453 | | | 9.06E-14 | | | 1.48E-11 | | 21.58037 | |
| CD58 | | 1.536138 | | 6.373474 | | 14.83801 | | | 9.15E-14 | | | 1.48E-11 | | 21.56948 | |
| CCNB2 | | -4.25937 | | 6.726763 | | -14.8376 | | | 9.16E-14 | | | 1.48E-11 | | 21.5688 | |
| USMG5 | | 1.285577 | | 8.1669 | | 14.7703 | | | 1.01E-13 | | | 1.60E-11 | | 21.46799 | |
| ORF1-FL49 | | 2.464905 | | 7.566896 | | 14.74933 | | | 1.05E-13 | | | 1.64E-11 | | 21.43648 | |
| KIAA1754 | | -1.26983 | | 9.198441 | | -14.7281 | | | 1.08E-13 | | | 1.68E-11 | | 21.40448 | |
| NUFIP2 | | -1.17226 | | 8.527141 | | -14.716 | | | 1.10E-13 | | | 1.69E-11 | | 21.38626 | |
| RBP1 | | 3.415833 | | 6.244101 | | 14.69933 | | | 1.13E-13 | | | 1.72E-11 | | 21.3612 | |
| NFKBIZ | | -2.99464 | | 9.64095 | | -14.6936 | | | 1.14E-13 | | | 1.72E-11 | | 21.35254 | |
| MIDN | | -1.85521 | | 8.857652 | | -14.6805 | | | 1.16E-13 | | | 1.74E-11 | | 21.33271 | |
| MGC39900 | | -1.9386 | | 8.117189 | | -14.65 | | | 1.21E-13 | | | 1.80E-11 | | 21.28675 | |
| NUPR1 | | 2.288926 | | 8.301736 | | 14.58752 | | | 1.33E-13 | | | 1.93E-11 | | 21.19203 | |
| DUSP1 | | -2.36911 | | 9.667481 | | -14.5866 | | | 1.34E-13 | | | 1.93E-11 | | 21.19069 | |
| C16orf60 | | -1.62683 | | 7.643554 | | -14.5819 | | | 1.35E-13 | | | 1.93E-11 | | 21.18351 | |
| GADD45B | | -2.94631 | | 8.438895 | | -14.5819 | | | 1.35E-13 | | | 1.93E-11 | | 21.18346 | |
| CPE | | 4.145556 | | 4.264079 | | 16.84453 | | | 1.67E-13 | | | 2.34E-11 | | 21.10307 | |
| P8 | | 2.322682 | | 7.863349 | | 14.51411 | | | 1.49E-13 | | | 2.13E-11 | | 21.08038 | |
| DRD1IP | | 2.767368 | | 4.926968 | | 15.46968 | | | 1.69E-13 | | | 2.34E-11 | | 21.0375 | |
| HRASLS3 | | 1.949025 | | 8.519705 | | 14.44631 | | | 1.65E-13 | | | 2.34E-11 | | 20.97681 | |
| ZFP36L2 | | -2.11239 | | 7.498642 | | -14.4216 | | | 1.72E-13 | | | 2.37E-11 | | 20.93904 | |
| PLOD2 | | 1.367998 | | 12.3718 | | 14.36582 | | | 1.87E-13 | | | 2.56E-11 | | 20.85335 | |
| VGLL4 | | -1.35622 | | 9.337841 | | -14.3176 | | | 2.02E-13 | | | 2.74E-11 | | 20.77912 | |
| RN7SK | | -1.27868 | | 7.142639 | | -14.3103 | | | 2.04E-13 | | | 2.75E-11 | | 20.76782 | |
| C7orf10 | | 2.04399 | | 8.643767 | | 14.22863 | | | 2.31E-13 | | | 3.09E-11 | | 20.64152 | |
| PARP14 | | -1.11436 | | 7.373036 | | -14.1724 | | | 2.52E-13 | | | 3.35E-11 | | 20.55423 | |
| ELF4 | | -1.17989 | | 8.159192 | | -14.1435 | | | 2.64E-13 | | | 3.47E-11 | | 20.50921 | |
| UBE2D4 | | 1.211109 | | 8.272022 | | 14.13401 | | | 2.68E-13 | | | 3.50E-11 | | 20.49443 | |
| NPAS1 | | 3.032636 | | 5.653665 | | 14.10307 | | | 2.81E-13 | | | 3.64E-11 | | 20.44615 | |
| SMARCD1 | | -1.14189 | | 9.233873 | | -14.0949 | | | 2.85E-13 | | | 3.66E-11 | | 20.43331 | |
| NUSAP1 | | -5.2798 | | 6.199952 | | -14.0614 | | | 3.00E-13 | | | 3.83E-11 | | 20.38095 | |
| RALGDS | | -1.37124 | | 8.828368 | | -14.0092 | | | 3.25E-13 | | | 4.12E-11 | | 20.29915 | |
| POLE | | -2.40324 | | 5.115543 | | -13.9916 | | | 3.34E-13 | | | 4.21E-11 | | 20.27144 | |
| ANKRD46 | | 1.172395 | | 6.863359 | | 13.98483 | | | 3.38E-13 | | | 4.22E-11 | | 20.26083 | |
| EFNB2 | | -2.2417 | | 7.303792 | | -13.979 | | | 3.41E-13 | | | 4.23E-11 | | 20.25171 | |
| BCOR | | -2.18412 | | 7.255392 | | -13.9233 | | | 3.72E-13 | | | 4.58E-11 | | 20.16386 | |
| ASPM | | -5.27885 | | 5.126431 | | -13.9156 | | | 3.77E-13 | | | 4.61E-11 | | 20.15168 | |
| TUBA1 | | 4.341143 | | 5.458106 | | 13.88278 | | | 3.97E-13 | | | 4.82E-11 | | 20.09984 | |
| AURKB | | -5.58902 | | 4.781144 | | -14.7509 | | | 4.50E-13 | | | 5.42E-11 | | 20.05975 | |
| NAP1L3 | | 5.740414 | | 5.132435 | | 18.77217 | | | 4.57E-13 | | | 5.44E-11 | | 20.05876 | |
| SIX5 | | -1.23356 | | 8.426623 | | -13.7946 | | | 4.56E-13 | | | 5.44E-11 | | 19.95992 | |
| LOC649853 | | -1.24056 | | 10.97119 | | -13.7854 | | | 4.63E-13 | | | 5.46E-11 | | 19.94526 | |
| CEP55 | | -3.2651 | | 5.681114 | | -13.7781 | | | 4.68E-13 | | | 5.46E-11 | | 19.93367 | |
| ERN1 | | -1.75919 | | 7.166471 | | -13.7728 | | | 4.72E-13 | | | 5.46E-11 | | 19.92522 | |
| ENAH | | 1.676447 | | 7.598303 | | 13.77267 | | | 4.72E-13 | | | 5.46E-11 | | 19.92502 | |
| ARL9 | | -3.62696 | | 4.488716 | | -17.3581 | | | 6.29E-13 | | | 6.86E-11 | | 19.83324 | |
| FLJ40629 | | -7.43618 | | 4.532206 | | -16.4594 | | | 6.36E-13 | | | 6.90E-11 | | 19.81481 | |
| SERTAD1 | | -1.5072 | | 9.999871 | | -13.6996 | | | 5.31E-13 | | | 6.10E-11 | | 19.8084 | |
| CD83 | | -1.70342 | | 6.316926 | | -13.6792 | | | 5.48E-13 | | | 6.26E-11 | | 19.77564 | |
| ZNF462 | | -1.44378 | | 7.653062 | | -13.6333 | | | 5.90E-13 | | | 6.62E-11 | | 19.70217 | |
| NASP | | -2.557 | | 5.892948 | | -13.6315 | | | 5.91E-13 | | | 6.62E-11 | | 19.69928 | |
| TRIP13 | | -2.88254 | | 6.119701 | | -13.6236 | | | 5.99E-13 | | | 6.65E-11 | | 19.68644 | |
| FLJ12505 | | -2.95854 | | 6.023712 | | -13.6202 | | | 6.02E-13 | | | 6.65E-11 | | 19.68097 | |
| PCBD1 | | 1.160544 | | 8.465776 | | 13.5986 | | | 6.23E-13 | | | 6.84E-11 | | 19.64629 | |
| C18orf24 | | -2.53954 | | 4.212819 | | -13.545 | | | 6.79E-13 | | | 7.32E-11 | | 19.5599 | |
| CCL26 | | 3.475297 | | 4.50531 | | 13.53448 | | | 6.91E-13 | | | 7.40E-11 | | 19.54289 | |
| HSPB8 | | 3.770059 | | 4.662696 | | 13.91564 | | | 7.40E-13 | | | 7.78E-11 | | 19.51969 | |
| TIMELESS | | -1.62837 | | 7.277728 | | -13.5105 | | | 7.18E-13 | | | 7.64E-11 | | 19.50413 | |
| PRIM1 | | -2.90116 | | 5.031419 | | -13.4977 | | | 7.33E-13 | | | 7.75E-11 | | 19.48342 | |
| PBX3 | | -1.15453 | | 9.687681 | | -13.4785 | | | 7.56E-13 | | | 7.90E-11 | | 19.45226 | |
| RDH5 | | 2.207674 | | 6.349238 | | 13.4611 | | | 7.78E-13 | | | 8.08E-11 | | 19.42405 | |
| CTSD | | 1.127817 | | 8.981326 | | 13.45695 | | | 7.83E-13 | | | 8.08E-11 | | 19.41732 | |
| THUMPD2 | | 1.207687 | | 7.469479 | | 13.43095 | | | 8.17E-13 | | | 8.38E-11 | | 19.37508 | |
| HSF2BP | | 2.332057 | | 7.03128 | | 13.38606 | | | 8.78E-13 | | | 8.96E-11 | | 19.302 | |
| LOC90835 | | 1.332485 | | 8.261999 | | 13.35862 | | | 9.18E-13 | | | 9.31E-11 | | 19.25721 | |
| NRP1 | | -1.49809 | | 9.567522 | | -13.3053 | | | 1.00E-12 | | | 1.01E-10 | | 19.16994 | |
| RBM39 | | -1.27404 | | 9.507363 | | -13.2869 | | | 1.03E-12 | | | 1.03E-10 | | 19.13991 | |
| YRDC | | -1.03662 | | 8.780827 | | -13.2764 | | | 1.05E-12 | | | 1.05E-10 | | 19.12265 | |
| FLJ20035 | | -2.07521 | | 5.624469 | | -13.2549 | | | 1.09E-12 | | | 1.08E-10 | | 19.08731 | |
| PRKCQ | | 1.205939 | | 7.844804 | | 13.23984 | | | 1.11E-12 | | | 1.10E-10 | | 19.06254 | |
| CENTD3 | | -1.1902 | | 8.615053 | | -13.2252 | | | 1.14E-12 | | | 1.12E-10 | | 19.03848 | |
| ZNF651 | | 1.596561 | | 6.090655 | | 13.16184 | | | 1.27E-12 | | | 1.23E-10 | | 18.93392 | |
| CCNA2 | | -3.51083 | | 5.88329 | | -13.1178 | | | 1.36E-12 | | | 1.32E-10 | | 18.86101 | |
| BNIP2 | | -1.0351 | | 8.716868 | | -13.0929 | | | 1.42E-12 | | | 1.37E-10 | | 18.81981 | |
| LGMN | | 1.960726 | | 6.494501 | | 13.08097 | | | 1.45E-12 | | | 1.39E-10 | | 18.79993 | |
| TOP2A | | -4.93257 | | 6.764473 | | -13.8654 | | | 1.59E-12 | | | 1.49E-10 | | 18.79626 | |
| TK1 | | -3.91249 | | 6.463686 | | -13.0746 | | | 1.46E-12 | | | 1.39E-10 | | 18.7894 | |
| TMSL8 | | -6.20755 | | 6.605528 | | -13.8479 | | | 1.63E-12 | | | 1.51E-10 | | 18.77056 | |
| CYP1B1 | | 2.839988 | | 7.233472 | | 13.06055 | | | 1.50E-12 | | | 1.42E-10 | | 18.766 | |
| HERC5 | | -4.54877 | | 4.941049 | | -13.4175 | | | 1.59E-12 | | | 1.49E-10 | | 18.7497 | |
| FZD8 | | 2.032522 | | 7.240836 | | 13.02001 | | | 1.60E-12 | | | 1.49E-10 | | 18.69851 | |
| HSPB3 | | 3.130054 | | 5.57995 | | 12.98935 | | | 1.68E-12 | | | 1.54E-10 | | 18.64734 | |
| HAGH | | 1.207804 | | 9.912813 | | 12.97625 | | | 1.72E-12 | | | 1.57E-10 | | 18.62546 | |
| GPX3 | | 2.893988 | | 6.818165 | | 12.9717 | | | 1.73E-12 | | | 1.57E-10 | | 18.61784 | |
| EGR1 | | -3.19549 | | 10.96656 | | -12.956 | | | 1.78E-12 | | | 1.61E-10 | | 18.59156 | |
| SLC30A1 | | -1.87641 | | 5.837098 | | -12.9192 | | | 1.89E-12 | | | 1.70E-10 | | 18.52996 | |
| MAP3K8 | | -5.24606 | | 5.304157 | | -13.276 | | | 1.99E-12 | | | 1.77E-10 | | 18.5268 | |
| ERRFI1 | | 1.448094 | | 9.735048 | | 12.90277 | | | 1.94E-12 | | | 1.74E-10 | | 18.50235 | |
| Pfs2 | | -4.0366 | | 6.209466 | | -12.873 | | | 2.04E-12 | | | 1.80E-10 | | 18.45233 | |
| TXNIP | | 3.897052 | | 7.293653 | | 12.87195 | | | 2.05E-12 | | | 1.80E-10 | | 18.45056 | |
| PGK1 | | 1.072118 | | 9.120011 | | 12.86299 | | | 2.08E-12 | | | 1.81E-10 | | 18.43547 | |
| ETV3 | | -2.64429 | | 5.450357 | | -12.8604 | | | 2.09E-12 | | | 1.81E-10 | | 18.43112 | |
| MGC17839 | | 1.204164 | | 9.046209 | | 12.8588 | | | 2.09E-12 | | | 1.81E-10 | | 18.42842 | |
| CPXM2 | | 4.514932 | | 5.907293 | | 14.04506 | | | 2.51E-12 | | | 2.13E-10 | | 18.38655 | |
| C10orf59 | | 1.233997 | | 6.037694 | | 12.82235 | | | 2.22E-12 | | | 1.92E-10 | | 18.36698 | |
| TGM2 | | 1.296752 | | 11.94492 | | 12.78977 | | | 2.35E-12 | | | 2.01E-10 | | 18.31195 | |
| ARL5B | | -1.77577 | | 6.276655 | | -12.7591 | | | 2.47E-12 | | | 2.11E-10 | | 18.25999 | |
| LIG1 | | -2.41179 | | 5.558133 | | -12.746 | | | 2.53E-12 | | | 2.14E-10 | | 18.23778 | |
| HMGB3 | | -1.70126 | | 6.630982 | | -12.7329 | | | 2.59E-12 | | | 2.16E-10 | | 18.21568 | |
| HNRPM | | -1.53111 | | 10.89093 | | -12.7297 | | | 2.60E-12 | | | 2.16E-10 | | 18.21023 | |
| KIF20A | | -5.4515 | | 5.486553 | | -13.9007 | | | 3.07E-12 | | | 2.49E-10 | | 18.18289 | |
| PNRC1 | | -3.80216 | | 3.713078 | | -13.4356 | | | 3.01E-12 | | | 2.46E-10 | | 18.15773 | |
| UCHL1 | | 1.501301 | | 12.0841 | | 12.6928 | | | 2.77E-12 | | | 2.29E-10 | | 18.1475 | |
| ENPP2 | | -2.3199 | | 4.727036 | | -13.0333 | | | 2.92E-12 | | | 2.40E-10 | | 18.14013 | |
| ATP7B | | 1.586152 | | 6.128986 | | 12.68701 | | | 2.79E-12 | | | 2.30E-10 | | 18.13766 | |
| NT5E | | 2.401165 | | 8.227116 | | 12.63445 | | | 3.05E-12 | | | 2.48E-10 | | 18.04809 | |
| March 4 | | 2.065621 | | 7.218666 | | 12.62109 | | | 3.12E-12 | | | 2.52E-10 | | 18.02527 | |
| MDK | | -2.43836 | | 11.29853 | | -12.5849 | | | 3.32E-12 | | | 2.66E-10 | | 17.96333 | |
| ASNS | | 2.018978 | | 9.839068 | | 12.56267 | | | 3.45E-12 | | | 2.73E-10 | | 17.92527 | |
| CREBBP | | -1.17041 | | 8.635751 | | -12.5143 | | | 3.75E-12 | | | 2.95E-10 | | 17.84215 | |
| AUTS2 | | -3.01565 | | 5.739029 | | -12.5033 | | | 3.82E-12 | | | 2.99E-10 | | 17.82329 | |
| HES1 | | -6.49295 | | 6.988067 | | -12.8068 | | | 4.20E-12 | | | 3.26E-10 | | 17.77397 | |
| CD248 | | -1.29343 | | 11.1624 | | -12.4587 | | | 4.12E-12 | | | 3.21E-10 | | 17.74637 | |
| CDCA8 | | -3.42848 | | 5.208772 | | -12.7785 | | | 4.40E-12 | | | 3.38E-10 | | 17.72775 | |
| SLC4A7 | | 1.668108 | | 9.383808 | | 12.42137 | | | 4.39E-12 | | | 3.38E-10 | | 17.68195 | |
| STMN1 | | -1.11843 | | 8.881215 | | -12.375 | | | 4.76E-12 | | | 3.64E-10 | | 17.60159 | |
| SPC24 | | -2.6218 | | 5.870097 | | -12.3693 | | | 4.80E-12 | | | 3.66E-10 | | 17.59171 | |
| PRIC285 | | -1.3355 | | 8.784151 | | -12.319 | | | 5.24E-12 | | | 3.98E-10 | | 17.50434 | |
| IFI6 | | -2.27088 | | 9.225422 | | -12.2931 | | | 5.48E-12 | | | 4.14E-10 | | 17.45925 | |
| SVEP1 | | -1.74184 | | 11.4075 | | -12.2812 | | | 5.59E-12 | | | 4.21E-10 | | 17.43845 | |
| DDX39 | | -1.88754 | | 9.457829 | | -12.2638 | | | 5.76E-12 | | | 4.30E-10 | | 17.40799 | |
| KIF11 | | -3.79721 | | 5.059577 | | -12.5293 | | | 6.61E-12 | | | 4.89E-10 | | 17.31825 | |
| RARRES1 | | -2.84607 | | 5.537488 | | -12.2109 | | | 6.32E-12 | | | 4.69E-10 | | 17.31545 | |
| CRYAB | | 5.72518 | | 5.796715 | | 20.67388 | | | 3.37E-12 | | | 2.67E-10 | | 17.28882 | |
| COL18A1 | | -1.15998 | | 9.291764 | | -12.1741 | | | 6.73E-12 | | | 4.96E-10 | | 17.251 | |
| NAPB | | 1.406301 | | 6.928319 | | 12.14099 | | | 7.13E-12 | | | 5.23E-10 | | 17.19273 | |
| SUV39H1 | | -1.59278 | | 6.850077 | | -12.132 | | | 7.25E-12 | | | 5.25E-10 | | 17.17693 | |
| ISG20 | | -2.74215 | | 5.014736 | | -12.132 | | | 7.25E-12 | | | 5.25E-10 | | 17.17684 | |
| GEM | | -2.22255 | | 6.187099 | | -12.1235 | | | 7.35E-12 | | | 5.30E-10 | | 17.16191 | |
| TM6SF1 | | 3.495445 | | 4.993388 | | 12.11855 | | | 7.42E-12 | | | 5.33E-10 | | 17.15321 | |
| BST2 | | -2.60487 | | 6.536001 | | -12.0567 | | | 8.27E-12 | | | 5.91E-10 | | 17.04396 | |
| NOTCH3 | | 1.40199 | | 6.750262 | | 12.0312 | | | 8.65E-12 | | | 6.16E-10 | | 16.99884 | |
| TPX2 | | -4.27891 | | 4.715008 | | -12.2745 | | | 1.01E-11 | | | 7.03E-10 | | 16.89262 | |
| DACT1 | | -2.38663 | | 5.615498 | | -11.9663 | | | 9.69E-12 | | | 6.87E-10 | | 16.8835 | |
| ANXA11 | | -1.12143 | | 8.350458 | | -11.9594 | | | 9.81E-12 | | | 6.89E-10 | | 16.87124 | |
| BRD2 | | -1.27404 | | 10.37502 | | -11.9583 | | | 9.83E-12 | | | 6.89E-10 | | 16.86927 | |
| KLF6 | | -1.2404 | | 10.57955 | | -11.9361 | | | 1.02E-11 | | | 7.10E-10 | | 16.82985 | |
| PSMB9 | | -3.01513 | | 5.859233 | | -11.9115 | | | 1.07E-11 | | | 7.36E-10 | | 16.78585 | |
| CDCA3 | | -4.18751 | | 4.480852 | | -12.5316 | | | 1.21E-11 | | | 8.09E-10 | | 16.75733 | |
| FN1 | | 1.898797 | | 8.014589 | | 11.88882 | | | 1.11E-11 | | | 7.63E-10 | | 16.74539 | |
| SHC4 | | 2.223816 | | 5.77229 | | 11.87998 | | | 1.13E-11 | | | 7.72E-10 | | 16.72958 | |
| MPHOSPH1 | | -3.02068 | | 6.138541 | | -11.8614 | | | 1.17E-11 | | | 7.88E-10 | | 16.69624 | |
| NETO2 | | -3.61236 | | 5.938167 | | -11.8608 | | | 1.17E-11 | | | 7.88E-10 | | 16.6953 | |
| SIPA1L2 | | -2.0245 | | 10.68545 | | -11.8599 | | | 1.17E-11 | | | 7.88E-10 | | 16.69366 | |
| INSIG1 | | -2.3272 | | 6.084933 | | -11.8598 | | | 1.17E-11 | | | 7.88E-10 | | 16.69353 | |
| TGIF1 | | -1.09569 | | 9.299324 | | -11.8537 | | | 1.18E-11 | | | 7.93E-10 | | 16.68255 | |
| UPP1 | | -1.62889 | | 8.826491 | | -11.8304 | | | 1.23E-11 | | | 8.20E-10 | | 16.64071 | |
| SLC31A2 | | 1.617149 | | 7.819911 | | 11.79499 | | | 1.31E-11 | | | 8.70E-10 | | 16.57712 | |
| DMD | | 2.067422 | | 6.02046 | | 11.7785 | | | 1.35E-11 | | | 8.91E-10 | | 16.54745 | |
| PRKCH | | 2.009975 | | 4.016088 | | 11.77766 | | | 1.35E-11 | | | 8.91E-10 | | 16.54593 | |
| WDR51A | | -2.36881 | | 6.889189 | | -11.7513 | | | 1.42E-11 | | | 9.30E-10 | | 16.49835 | |
| TRMT5 | | -1.30529 | | 10.68742 | | -11.7195 | | | 1.50E-11 | | | 9.80E-10 | | 16.4411 | |
| C1QTNF6 | | -1.52268 | | 7.288513 | | -11.7179 | | | 1.51E-11 | | | 9.80E-10 | | 16.43811 | |
| ANKRD10 | | -1.42317 | | 7.422749 | | -11.7146 | | | 1.52E-11 | | | 9.82E-10 | | 16.43225 | |
| MPP4 | | 3.486336 | | 5.025186 | | 11.69427 | | | 1.57E-11 | | | 1.01E-09 | | 16.3954 | |
| CRISPLD2 | | -5.40047 | | 4.593996 | | -13.5745 | | | 2.06E-11 | | | 1.27E-09 | | 16.37554 | |
| AURKA | | -2.17599 | | 7.136254 | | -11.6562 | | | 1.68E-11 | | | 1.08E-09 | | 16.32646 | |
| PURA | | 1.705383 | | 6.70907 | | 11.64765 | | | 1.71E-11 | | | 1.09E-09 | | 16.31089 | |
| SAA1 | | 2.816241 | | 5.934858 | | 11.63771 | | | 1.74E-11 | | | 1.11E-09 | | 16.29285 | |
| NET1 | | -1.04903 | | 9.262426 | | -11.6138 | | | 1.82E-11 | | | 1.15E-09 | | 16.2494 | |
| IFIT3 | | -2.49112 | | 6.474917 | | -11.6071 | | | 1.84E-11 | | | 1.16E-09 | | 16.23717 | |
| CDC45L | | -5.62796 | | 5.1302 | | -12.1908 | | | 2.09E-11 | | | 1.29E-09 | | 16.20809 | |
| C18orf56 | | -2.0434 | | 6.030137 | | -11.5891 | | | 1.90E-11 | | | 1.19E-09 | | 16.20447 | |
| MCM5 | | -2.66742 | | 5.931188 | | -11.5635 | | | 1.99E-11 | | | 1.25E-09 | | 16.15766 | |
| PSCD1 | | -1.13269 | | 8.424252 | | -11.5571 | | | 2.01E-11 | | | 1.26E-09 | | 16.14605 | |
| ACOT7 | | 1.029124 | | 10.45618 | | 11.54575 | | | 2.05E-11 | | | 1.27E-09 | | 16.12531 | |
| SLC15A3 | | -1.84602 | | 7.171475 | | -11.5442 | | | 2.06E-11 | | | 1.27E-09 | | 16.12251 | |
| ID2 | | -3.38272 | | 9.037538 | | -11.497 | | | 2.24E-11 | | | 1.38E-09 | | 16.03603 | |
| DLX1 | | -1.44703 | | 9.093228 | | -11.4876 | | | 2.28E-11 | | | 1.39E-09 | | 16.01883 | |
| ANKRD34 | | 1.801358 | | 6.186083 | | 11.47555 | | | 2.33E-11 | | | 1.42E-09 | | 15.99677 | |
| LBH | | 2.116674 | | 8.446561 | | 11.47305 | | | 2.34E-11 | | | 1.42E-09 | | 15.99218 | |
| SDC1 | | -1.58431 | | 8.326223 | | -11.4564 | | | 2.42E-11 | | | 1.46E-09 | | 15.96163 | |
| FMNL2 | | -1.62167 | | 5.674299 | | -11.4546 | | | 2.42E-11 | | | 1.46E-09 | | 15.9583 | |
| SMC4 | | -1.81666 | | 8.005871 | | -11.4374 | | | 2.50E-11 | | | 1.50E-09 | | 15.92674 | |
| CARKL | | -1.22943 | | 7.503623 | | -11.4333 | | | 2.52E-11 | | | 1.51E-09 | | 15.91913 | |
| RBM4 | | -1.07319 | | 9.733843 | | -11.4289 | | | 2.54E-11 | | | 1.51E-09 | | 15.91109 | |
| COL15A1 | | 7.717086 | | 6.956628 | | 16.38024 | | | 3.14E-11 | | | 1.82E-09 | | 15.88779 | |
| FAM46A | | -1.21686 | | 9.462471 | | -11.4129 | | | 2.61E-11 | | | 1.55E-09 | | 15.88162 | |
| HIC2 | | -1.07722 | | 6.718744 | | -11.3957 | | | 2.70E-11 | | | 1.59E-09 | | 15.84983 | |
| RRAS2 | | 1.387153 | | 8.614941 | | 11.39519 | | | 2.70E-11 | | | 1.59E-09 | | 15.84894 | |
| MAF | | -3.00557 | | 4.776628 | | -11.9283 | | | 3.21E-11 | | | 1.85E-09 | | 15.77685 | |
| TMEM16A | | 2.498784 | | 8.275411 | | 11.3427 | | | 2.97E-11 | | | 1.74E-09 | | 15.75197 | |
| SDF2L1 | | -1.75248 | | 10.87712 | | -11.3413 | | | 2.98E-11 | | | 1.74E-09 | | 15.74944 | |
| TACC3 | | -2.40883 | | 4.804808 | | -11.3179 | | | 3.11E-11 | | | 1.80E-09 | | 15.70606 | |
| ECH1 | | -1.20821 | | 10.48814 | | -11.2883 | | | 3.28E-11 | | | 1.89E-09 | | 15.65112 | |
| C19orf48 | | -1.77328 | | 6.059417 | | -11.2762 | | | 3.36E-11 | | | 1.92E-09 | | 15.62864 | |
| TNNT2 | | 3.800251 | | 5.87107 | | 11.25946 | | | 3.46E-11 | | | 1.97E-09 | | 15.59751 | |
| FBXO15 | | 2.955564 | | 5.005181 | | 11.20768 | | | 3.81E-11 | | | 2.15E-09 | | 15.50104 | |
| ANLN | | -3.26227 | | 5.211722 | | -11.4592 | | | 4.07E-11 | | | 2.27E-09 | | 15.48384 | |
| ACPP | | 7.772563 | | 6.700975 | | 17.50309 | | | 3.48E-11 | | | 1.97E-09 | | 15.42806 | |
| C8orf72 | | -2.40817 | | 5.435796 | | -11.158 | | | 4.18E-11 | | | 2.32E-09 | | 15.40817 | |
| SNAI2 | | -1.83006 | | 9.837849 | | -11.1502 | | | 4.24E-11 | | | 2.35E-09 | | 15.39365 | |
| CDKN3 | | -2.71907 | | 5.929496 | | -11.1484 | | | 4.25E-11 | | | 2.35E-09 | | 15.39013 | |
| SNX26 | | -1.49628 | | 6.579173 | | -11.1317 | | | 4.38E-11 | | | 2.41E-09 | | 15.35884 | |
| CHTF18 | | -3.28843 | | 4.019001 | | -11.3798 | | | 4.68E-11 | | | 2.55E-09 | | 15.34261 | |
| S100A4 | | 1.605137 | | 12.39934 | | 11.11558 | | | 4.52E-11 | | | 2.47E-09 | | 15.32865 | |
| IER2 | | -2.51914 | | 6.466653 | | -11.0923 | | | 4.72E-11 | | | 2.56E-09 | | 15.28485 | |
| DUSP5 | | -1.35924 | | 8.972213 | | -11.0921 | | | 4.72E-11 | | | 2.56E-09 | | 15.28449 | |
| OAS2 | | -2.79978 | | 6.006966 | | -11.0884 | | | 4.75E-11 | | | 2.57E-09 | | 15.27752 | |
| PARP8 | | 1.229704 | | 8.119374 | | 11.08623 | | | 4.77E-11 | | | 2.57E-09 | | 15.27348 | |
| BCL2L12 | | -1.19423 | | 8.108541 | | -11.0842 | | | 4.79E-11 | | | 2.57E-09 | | 15.26976 | |
| KLF11 | | -2.75862 | | 7.363935 | | -11.014 | | | 5.46E-11 | | | 2.91E-09 | | 15.13738 | |
| SYT12 | | 3.269901 | | 4.02684 | | 11.52185 | | | 6.31E-11 | | | 3.26E-09 | | 15.09771 | |
| SFRS5 | | -1.1322 | | 11.28276 | | -10.9897 | | | 5.71E-11 | | | 3.02E-09 | | 15.09131 | |
| MAP1B | | 1.531275 | | 9.047376 | | 10.98864 | | | 5.72E-11 | | | 3.02E-09 | | 15.08937 | |
| SPINT2 | | 2.486077 | | 5.294195 | | 10.96082 | | | 6.03E-11 | | | 3.16E-09 | | 15.03668 | |
| GLRB | | 1.538112 | | 7.551058 | | 10.95128 | | | 6.14E-11 | | | 3.21E-09 | | 15.01859 | |
| AOX1 | | 4.409248 | | 5.938164 | | 15.33928 | | | 8.25E-11 | | | 4.09E-09 | | 15.00447 | |
| MCM4 | | -2.71052 | | 7.116135 | | -10.9391 | | | 6.28E-11 | | | 3.26E-09 | | 14.99549 | |
| C20orf100 | | -1.17542 | | 10.29531 | | -10.9304 | | | 6.38E-11 | | | 3.29E-09 | | 14.97903 | |
| COL16A1 | | -1.9152 | | 9.164836 | | -10.9211 | | | 6.50E-11 | | | 3.34E-09 | | 14.96125 | |
| ERCC6 | | 2.701527 | | 4.761388 | | 11.15583 | | | 6.97E-11 | | | 3.57E-09 | | 14.94235 | |
| FLJ46536 | | -2.75016 | | 3.876301 | | -11.7137 | | | 8.14E-11 | | | 4.04E-09 | | 14.89109 | |
| ADAM23 | | 1.856515 | | 6.115728 | | 10.88282 | | | 6.98E-11 | | | 3.57E-09 | | 14.88844 | |
| TAGLN3 | | -3.36655 | | 4.393433 | | -11.119 | | | 7.44E-11 | | | 3.77E-09 | | 14.87389 | |
| LDB2 | | -1.18482 | | 10.13487 | | -10.875 | | | 7.09E-11 | | | 3.61E-09 | | 14.87362 | |
| C21orf34 | | 1.610653 | | 5.39653 | | 10.85341 | | | 7.38E-11 | | | 3.75E-09 | | 14.83235 | |
| PLEKHH3 | | -1.43232 | | 6.448637 | | -10.8375 | | | 7.61E-11 | | | 3.83E-09 | | 14.8019 | |
| ZNF161 | | -1.20093 | | 9.857553 | | -10.8359 | | | 7.63E-11 | | | 3.83E-09 | | 14.79894 | |
| MELK | | -2.53639 | | 7.414201 | | -10.8345 | | | 7.65E-11 | | | 3.83E-09 | | 14.79632 | |
| C1orf24 | | 1.727672 | | 7.467627 | | 10.82304 | | | 7.82E-11 | | | 3.91E-09 | | 14.77432 | |
| FLJ43339 | | 1.716654 | | 9.046571 | | 10.81221 | | | 7.98E-11 | | | 3.97E-09 | | 14.75362 | |
| NFKB1 | | -1.10141 | | 11.06063 | | -10.7832 | | | 8.43E-11 | | | 4.16E-09 | | 14.69805 | |
| SERPINE1 | | 2.908117 | | 9.403801 | | 10.73748 | | | 9.19E-11 | | | 4.53E-09 | | 14.61026 | |
| BTBD11 | | -2.7392 | | 6.509596 | | -10.7359 | | | 9.22E-11 | | | 4.53E-09 | | 14.60723 | |
| C9orf9 | | 1.614028 | | 6.004571 | | 10.70878 | | | 9.71E-11 | | | 4.75E-09 | | 14.55501 | |
| JOSD3 | | 1.377525 | | 5.920594 | | 10.69898 | | | 9.89E-11 | | | 4.83E-09 | | 14.53613 | |
| C20orf111 | | -1.16005 | | 9.709497 | | -10.6769 | | | 1.03E-10 | | | 5.01E-09 | | 14.4935 | |
| ZNF508 | | -1.08915 | | 7.578707 | | -10.6585 | | | 1.07E-10 | | | 5.17E-09 | | 14.45793 | |
| CYBA | | -1.17715 | | 9.130527 | | -10.6396 | | | 1.11E-10 | | | 5.35E-09 | | 14.42152 | |
| SH3BGRL2 | | 1.825532 | | 7.957831 | | 10.60361 | | | 1.19E-10 | | | 5.71E-09 | | 14.35173 | |
| SRF | | -1.41404 | | 10.30753 | | -10.589 | | | 1.22E-10 | | | 5.84E-09 | | 14.32334 | |
| PPAPDC1A | | -2.90306 | | 6.079197 | | -10.5856 | | | 1.23E-10 | | | 5.87E-09 | | 14.31675 | |
| ARS2 | | -1.02506 | | 10.59118 | | -10.5399 | | | 1.34E-10 | | | 6.39E-09 | | 14.22802 | |
| GABBR2 | | 2.401352 | | 6.88827 | | 10.53678 | | | 1.35E-10 | | | 6.41E-09 | | 14.22187 | |
| LOC285989 | | 1.543425 | | 6.568911 | | 10.53484 | | | 1.35E-10 | | | 6.41E-09 | | 14.21808 | |
| LRRC32 | | 2.032807 | | 7.373637 | | 10.53324 | | | 1.36E-10 | | | 6.42E-09 | | 14.21496 | |
| RBMX | | -1.06995 | | 8.719376 | | -10.5236 | | | 1.38E-10 | | | 6.51E-09 | | 14.19614 | |
| SMOX | | -1.18101 | | 6.808321 | | -10.5232 | | | 1.39E-10 | | | 6.51E-09 | | 14.19538 | |
| TP53BP2 | | -1.38696 | | 7.742221 | | -10.5193 | | | 1.40E-10 | | | 6.54E-09 | | 14.18779 | |
| TIA1 | | -1.33002 | | 8.887244 | | -10.5162 | | | 1.40E-10 | | | 6.56E-09 | | 14.18167 | |
| ADFP | | -1.45994 | | 9.985893 | | -10.4997 | | | 1.45E-10 | | | 6.73E-09 | | 14.14954 | |
| BCL2L2 | | 1.288855 | | 8.836535 | | 10.48218 | | | 1.50E-10 | | | 6.93E-09 | | 14.11534 | |
| PHF19 | | -1.64472 | | 6.153047 | | -10.4642 | | | 1.55E-10 | | | 7.15E-09 | | 14.08026 | |
| BNC2 | | -1.9531 | | 7.056081 | | -10.4433 | | | 1.62E-10 | | | 7.43E-09 | | 14.03931 | |
| HERC6 | | -2.29378 | | 6.135301 | | -10.441 | | | 1.62E-10 | | | 7.44E-09 | | 14.03478 | |
| CRSP7 | | -1.80592 | | 5.534791 | | -10.4285 | | | 1.66E-10 | | | 7.58E-09 | | 14.01028 | |
| BAMBI | | -1.64654 | | 10.01696 | | -10.4246 | | | 1.68E-10 | | | 7.62E-09 | | 14.00259 | |
| KLF10 | | -5.47606 | | 6.695565 | | -10.4208 | | | 1.69E-10 | | | 7.66E-09 | | 13.99518 | |
| TRA2A | | -1.13327 | | 8.118307 | | -10.4145 | | | 1.71E-10 | | | 7.73E-09 | | 13.98286 | |
| WFDC1 | | 7.58667 | | 5.251398 | | 11.15193 | | | 2.03E-10 | | | 8.88E-09 | | 13.97239 | |
| UBE2E2 | | 1.823634 | | 6.579794 | | 10.406 | | | 1.74E-10 | | | 7.83E-09 | | 13.9661 | |
| ZCCHC14 | | -1.12109 | | 7.37028 | | -10.4052 | | | 1.74E-10 | | | 7.83E-09 | | 13.96446 | |
| NUP62 | | -1.03106 | | 10.33281 | | -10.3911 | | | 1.79E-10 | | | 8.03E-09 | | 13.93691 | |
| FLJ10374 | | -1.47289 | | 5.291368 | | -10.3744 | | | 1.85E-10 | | | 8.22E-09 | | 13.90394 | |
| ITGBL1 | | 2.16602 | | 5.218038 | | 10.37379 | | | 1.85E-10 | | | 8.22E-09 | | 13.90277 | |
| PSD3 | | -1.36355 | | 8.407084 | | -10.3734 | | | 1.85E-10 | | | 8.22E-09 | | 13.90198 | |
| FKSG14 | | -3.09526 | | 5.177197 | | -10.5874 | | | 1.96E-10 | | | 8.58E-09 | | 13.89334 | |
| TOX | | -2.4185 | | 5.790542 | | -10.3638 | | | 1.89E-10 | | | 8.36E-09 | | 13.88309 | |
| SIRT1 | | -1.4688 | | 7.100072 | | -10.3551 | | | 1.92E-10 | | | 8.48E-09 | | 13.86588 | |
| MRAS | | 1.984817 | | 6.875012 | | 10.35221 | | | 1.93E-10 | | | 8.50E-09 | | 13.86028 | |
| MCM2 | | -2.73996 | | 5.599056 | | -10.3488 | | | 1.94E-10 | | | 8.54E-09 | | 13.85357 | |
| LRRCC1 | | -1.57544 | | 5.955146 | | -10.3459 | | | 1.95E-10 | | | 8.56E-09 | | 13.84778 | |
| MAB21L2 | | -1.28517 | | 10.25469 | | -10.3223 | | | 2.04E-10 | | | 8.90E-09 | | 13.80121 | |
| WDR58 | | -1.38365 | | 7.009036 | | -10.3062 | | | 2.11E-10 | | | 9.14E-09 | | 13.76943 | |
| POLD1 | | -1.39542 | | 4.884519 | | -10.2494 | | | 2.36E-10 | | | 1.01E-08 | | 13.65695 | |
| ABLIM1 | | 1.746942 | | 7.734169 | | 10.2438 | | | 2.38E-10 | | | 1.02E-08 | | 13.64592 | |
| CEP1 | | -2.3765 | | 4.013326 | | -10.6793 | | | 2.71E-10 | | | 1.13E-08 | | 13.61977 | |
| ZNF564 | | 2.04853 | | 5.102543 | | 10.22879 | | | 2.45E-10 | | | 1.05E-08 | | 13.61612 | |
| SLC25A4 | | 1.411048 | | 9.067775 | | 10.22847 | | | 2.46E-10 | | | 1.05E-08 | | 13.61549 | |
| KLF13 | | -1.01862 | | 8.58048 | | -10.2032 | | | 2.58E-10 | | | 1.09E-08 | | 13.56518 | |
| VPREB3 | | 2.703603 | | 3.727518 | | 10.90705 | | | 3.06E-10 | | | 1.27E-08 | | 13.55856 | |
| PTPRE | | 2.018385 | | 6.206174 | | 10.19575 | | | 2.62E-10 | | | 1.10E-08 | | 13.55042 | |
| BUB1 | | -4.48662 | | 4.890324 | | -12.9023 | | | 4.61E-10 | | | 1.81E-08 | | 13.42927 | |
| TEAD2 | | -1.14557 | | 10.74652 | | -10.1189 | | | 3.05E-10 | | | 1.27E-08 | | 13.39701 | |
| IER5 | | -1.17747 | | 8.897266 | | -10.118 | | | 3.05E-10 | | | 1.27E-08 | | 13.3954 | |
| CDC25A | | -3.13687 | | 4.7183 | | -10.5313 | | | 3.53E-10 | | | 1.43E-08 | | 13.35222 | |
| TAF13 | | 1.46001 | | 6.029587 | | 10.07214 | | | 3.34E-10 | | | 1.38E-08 | | 13.30344 | |
| MGST2 | | 1.092986 | | 9.942437 | | 10.07197 | | | 3.34E-10 | | | 1.38E-08 | | 13.30311 | |
| NR1D2 | | -2.90845 | | 5.150658 | | -10.0574 | | | 3.44E-10 | | | 1.41E-08 | | 13.27385 | |
| INMT | | 2.183086 | | 7.661272 | | 10.05502 | | | 3.46E-10 | | | 1.41E-08 | | 13.26908 | |
| CHPF | | 1.250101 | | 9.299841 | | 10.04944 | | | 3.50E-10 | | | 1.42E-08 | | 13.25788 | |
| THAP6 | | 1.51079 | | 5.642778 | | 10.03114 | | | 3.63E-10 | | | 1.47E-08 | | 13.22109 | |
| SLCO2A1 | | 4.355878 | | 4.147897 | | 11.69355 | | | 5.30E-10 | | | 2.02E-08 | | 13.19241 | |
| CEP135 | | -1.50039 | | 7.017576 | | -9.98997 | | | 3.94E-10 | | | 1.58E-08 | | 13.1382 | |
| FKBP4 | | 1.007369 | | 7.081755 | | 9.966706 | | | 4.12E-10 | | | 1.65E-08 | | 13.09124 | |
| VAPA | | -2.4062 | | 4.721494 | | -9.95957 | | | 4.18E-10 | | | 1.67E-08 | | 13.07683 | |
| TIMP1 | | 1.158319 | | 14.20278 | | 9.950447 | | | 4.26E-10 | | | 1.69E-08 | | 13.05839 | |
| COPZ2 | | 1.265142 | | 8.440556 | | 9.949918 | | | 4.26E-10 | | | 1.69E-08 | | 13.05732 | |
| TMEM35 | | -1.71366 | | 5.762267 | | -9.92744 | | | 4.46E-10 | | | 1.76E-08 | | 13.01186 | |
| CCNF | | -3.29363 | | 5.796278 | | -9.89429 | | | 4.76E-10 | | | 1.87E-08 | | 12.94466 | |
| CXYorf3 | | -1.03768 | | 9.308663 | | -9.88781 | | | 4.83E-10 | | | 1.88E-08 | | 12.93152 | |
| NME1-NME2 | | 1.175983 | | 7.275215 | | 9.886085 | | | 4.84E-10 | | | 1.88E-08 | | 12.92802 | |
| PIM3 | | -2.33599 | | 6.044489 | | -9.8811 | | | 4.89E-10 | | | 1.89E-08 | | 12.91789 | |
| RNASEH2A | | -1.6289 | | 5.880239 | | -9.86397 | | | 5.06E-10 | | | 1.95E-08 | | 12.8831 | |
| PYCARD | | -1.60211 | | 8.87397 | | -9.8525 | | | 5.18E-10 | | | 1.99E-08 | | 12.85977 | |
| ALPL | | 2.322295 | | 7.652763 | | 9.851549 | | | 5.19E-10 | | | 1.99E-08 | | 12.85784 | |
| LOC387921 | | 1.034424 | | 7.127953 | | 9.849677 | | | 5.21E-10 | | | 2.00E-08 | | 12.85403 | |
| ATOH8 | | -3.4525 | | 4.634067 | | -10.7794 | | | 6.48E-10 | | | 2.35E-08 | | 12.85398 | |
| ZNF586 | | -1.86374 | | 7.05795 | | -9.83836 | | | 5.33E-10 | | | 2.03E-08 | | 12.83099 | |
| TBL1X | | -1.04913 | | 9.064196 | | -9.83668 | | | 5.35E-10 | | | 2.03E-08 | | 12.82758 | |
| MEF2D | | -1.5645 | | 8.851293 | | -9.834 | | | 5.38E-10 | | | 2.04E-08 | | 12.82213 | |
| ISG20L1 | | -1.01125 | | 9.446584 | | -9.83386 | | | 5.38E-10 | | | 2.04E-08 | | 12.82184 | |
| ROR2 | | 2.577088 | | 4.565544 | | 10.02483 | | | 5.67E-10 | | | 2.12E-08 | | 12.82046 | |
| BAZ1A | | -1.07658 | | 7.995462 | | -9.83171 | | | 5.40E-10 | | | 2.04E-08 | | 12.81747 | |
| CNN1 | | 2.089164 | | 7.295862 | | 9.825097 | | | 5.47E-10 | | | 2.06E-08 | | 12.80399 | |
| KIF23 | | -2.38317 | | 5.785186 | | -9.80766 | | | 5.67E-10 | | | 2.12E-08 | | 12.76844 | |
| EXOSC9 | | -1.02924 | | 7.206081 | | -9.80282 | | | 5.73E-10 | | | 2.13E-08 | | 12.75856 | |
| SLC7A5 | | 1.901815 | | 10.47971 | | 9.80232 | | | 5.73E-10 | | | 2.13E-08 | | 12.75754 | |
| CENPF | | -6.867 | | 5.646595 | | -11.8154 | | | 8.68E-10 | | | 3.00E-08 | | 12.74924 | |
| HMGB2 | | -2.94394 | | 6.851273 | | -9.79118 | | | 5.86E-10 | | | 2.18E-08 | | 12.7348 | |
| DYSF | | 3.354728 | | 4.912863 | | 9.790008 | | | 5.88E-10 | | | 2.18E-08 | | 12.73241 | |
| GARS | | 1.065414 | | 11.74657 | | 9.788356 | | | 5.89E-10 | | | 2.18E-08 | | 12.72904 | |
| MYL4 | | 4.333747 | | 5.82263 | | 9.776892 | | | 6.03E-10 | | | 2.22E-08 | | 12.70561 | |
| GLI2 | | -1.68866 | | 6.190056 | | -9.77454 | | | 6.06E-10 | | | 2.23E-08 | | 12.70081 | |
| TFAM | | -1.24926 | | 7.524219 | | -9.77372 | | | 6.07E-10 | | | 2.23E-08 | | 12.69913 | |
| TTC14 | | 1.057123 | | 6.935192 | | 9.770607 | | | 6.11E-10 | | | 2.24E-08 | | 12.69277 | |
| HLX1 | | 2.996176 | | 6.324816 | | 9.74301 | | | 6.46E-10 | | | 2.35E-08 | | 12.63629 | |
| GSDMDC1 | | -1.14682 | | 7.356572 | | -9.74107 | | | 6.49E-10 | | | 2.35E-08 | | 12.63231 | |
| IQCG | | 1.577239 | | 6.083842 | | 9.724682 | | | 6.70E-10 | | | 2.42E-08 | | 12.59873 | |
| ADAMTSL5 | | 1.817896 | | 5.265655 | | 9.724614 | | | 6.71E-10 | | | 2.42E-08 | | 12.59859 | |
| GRK4 | | 1.772678 | | 5.547888 | | 9.714492 | | | 6.84E-10 | | | 2.46E-08 | | 12.57783 | |
| PENK | | 5.785917 | | 7.271245 | | 11.23903 | | | 1.01E-09 | | | 3.36E-08 | | 12.54183 | |
| KBTBD11 | | 2.217396 | | 6.458208 | | 9.682036 | | | 7.31E-10 | | | 2.61E-08 | | 12.51116 | |
| HIC1 | | -2.36831 | | 4.612841 | | -9.86212 | | | 7.77E-10 | | | 2.74E-08 | | 12.5 | |
| CLN8 | | 2.029859 | | 4.930738 | | 9.672312 | | | 7.46E-10 | | | 2.65E-08 | | 12.49116 | |
| FOS | | -6.88653 | | 7.271776 | | -10.2971 | | | 8.75E-10 | | | 3.01E-08 | | 12.49057 | |
| NUDT6 | | 1.055566 | | 6.964911 | | 9.655359 | | | 7.72E-10 | | | 2.73E-08 | | 12.45626 | |
| SERTAD3 | | -1.08478 | | 8.479902 | | -9.65147 | | | 7.78E-10 | | | 2.74E-08 | | 12.44824 | |
| PKIA | | -1.0451 | | 6.775061 | | -9.64895 | | | 7.82E-10 | | | 2.74E-08 | | 12.44307 | |
| SLC16A4 | | 1.843889 | | 5.99242 | | 9.648513 | | | 7.82E-10 | | | 2.74E-08 | | 12.44216 | |
| CEBPB | | -1.29538 | | 11.87683 | | -9.62982 | | | 8.13E-10 | | | 2.84E-08 | | 12.40363 | |
| CCPG1 | | 1.278091 | | 10.2264 | | 9.620141 | | | 8.29E-10 | | | 2.89E-08 | | 12.38365 | |
| ZNF254 | | -1.40918 | | 4.403837 | | -9.61653 | | | 8.35E-10 | | | 2.91E-08 | | 12.37619 | |
| ISYNA1 | | -1.17585 | | 6.6147 | | -9.59249 | | | 8.77E-10 | | | 3.01E-08 | | 12.32651 | |
| EPC1 | | -1.86549 | | 4.928623 | | -9.59187 | | | 8.78E-10 | | | 3.01E-08 | | 12.32523 | |
| RNF144 | | -2.68417 | | 4.960731 | | -9.5888 | | | 8.84E-10 | | | 3.02E-08 | | 12.31889 | |
| PPME1 | | 1.191793 | | 9.633204 | | 9.583966 | | | 8.92E-10 | | | 3.05E-08 | | 12.30889 | |
| INHBE | | 5.137185 | | 5.661199 | | 10.43905 | | | 1.14E-09 | | | 3.73E-08 | | 12.29469 | |
| PSMD14 | | 1.171514 | | 9.835472 | | 9.57535 | | | 9.08E-10 | | | 3.09E-08 | | 12.29106 | |
| IMPA2 | | -1.64975 | | 6.09665 | | -9.57103 | | | 9.16E-10 | | | 3.12E-08 | | 12.28211 | |
| DDIT4L | | 2.469841 | | 6.174944 | | 9.56858 | | | 9.21E-10 | | | 3.12E-08 | | 12.27704 | |
| UBE2C | | -4.9211 | | 6.903805 | | -9.56683 | | | 9.24E-10 | | | 3.13E-08 | | 12.27343 | |
| CBX2 | | -1.37158 | | 7.406166 | | -9.56506 | | | 9.28E-10 | | | 3.14E-08 | | 12.26975 | |
| BNIP3 | | 1.439251 | | 10.35889 | | 9.550869 | | | 9.55E-10 | | | 3.22E-08 | | 12.24034 | |
| HSPA5 | | -1.28179 | | 10.56831 | | -9.53278 | | | 9.91E-10 | | | 3.31E-08 | | 12.20282 | |
| AZI1 | | -1.37438 | | 7.704519 | | -9.53256 | | | 9.91E-10 | | | 3.31E-08 | | 12.20236 | |
| UBE2T | | -1.61998 | | 6.613581 | | -9.5297 | | | 9.97E-10 | | | 3.32E-08 | | 12.19642 | |
| APOD | | 3.996441 | | 6.160522 | | 9.503455 | | | 1.05E-09 | | | 3.48E-08 | | 12.14189 | |
| ZMYM5 | | -1.22461 | | 6.524005 | | -9.49084 | | | 1.08E-09 | | | 3.57E-08 | | 12.11566 | |
| TRAF3IP2 | | -1.37685 | | 8.615655 | | -9.47985 | | | 1.10E-09 | | | 3.64E-08 | | 12.09278 | |
| SCML1 | | -1.17339 | | 7.137922 | | -9.47924 | | | 1.11E-09 | | | 3.64E-08 | | 12.0915 | |
| GLS | | 1.740677 | | 8.849284 | | 9.474084 | | | 1.12E-09 | | | 3.67E-08 | | 12.08076 | |
| CENPA | | -4.76886 | | 4.826068 | | -9.61179 | | | 1.27E-09 | | | 4.12E-08 | | 12.00361 | |
| LOC92270 | | 1.383771 | | 4.59974 | | 9.425377 | | | 1.24E-09 | | | 4.03E-08 | | 11.97914 | |
| CABLES2 | | -1.86238 | | 5.29527 | | -9.41482 | | | 1.26E-09 | | | 4.11E-08 | | 11.95707 | |
| NRIP3 | | 1.004827 | | 8.639765 | | 9.407268 | | | 1.28E-09 | | | 4.15E-08 | | 11.94127 | |
| DUSP22 | | 1.122021 | | 7.583415 | | 9.401624 | | | 1.30E-09 | | | 4.19E-08 | | 11.92946 | |
| TRERF1 | | -1.22435 | | 5.647699 | | -9.38997 | | | 1.33E-09 | | | 4.28E-08 | | 11.90507 | |
| PLK4 | | -4.2955 | | 4.994864 | | -9.54898 | | | 1.44E-09 | | | 4.59E-08 | | 11.8778 | |
| MARCKSL1 | | -2.19234 | | 10.33857 | | -9.37018 | | | 1.39E-09 | | | 4.45E-08 | | 11.86358 | |
| HDAC1 | | -1.01234 | | 10.6389 | | -9.36791 | | | 1.39E-09 | | | 4.46E-08 | | 11.85884 | |
| KIAA1794 | | -2.49613 | | 4.442404 | | -9.53285 | | | 1.48E-09 | | | 4.72E-08 | | 11.84541 | |
| COX17 | | 1.163569 | | 10.7932 | | 9.338953 | | | 1.48E-09 | | | 4.72E-08 | | 11.79805 | |
| DKFZp762E131 -3.25767 | | | | 4.677047 | | -9.33419 | | | 1.49E-09 | | | 4.75E-08 | | 11.78805 | |
| C16orf30 | | -3.02427 | | 4.685476 | | -9.31944 | | | 1.54E-09 | | | 4.88E-08 | | 11.75702 | |
| CDH15 | | -1.42494 | | 6.812008 | | -9.30791 | | | 1.58E-09 | | | 4.98E-08 | | 11.73275 | |
| FBXL10 | | -1.06134 | | 9.167971 | | -9.30012 | | | 1.60E-09 | | | 5.05E-08 | | 11.71636 | |
| TROAP | | -6.4229 | | 4.814806 | | -12.016 | | | 2.78E-09 | | | 8.13E-08 | | 11.69694 | |
| FLJ13909 | | -2.25696 | | 4.663383 | | -9.29052 | | | 1.63E-09 | | | 5.11E-08 | | 11.69613 | |
| EFNB3 | | -2.60797 | | 6.348849 | | -9.29019 | | | 1.64E-09 | | | 5.11E-08 | | 11.69543 | |
| APP | | -1.17933 | | 10.82407 | | -9.28569 | | | 1.65E-09 | | | 5.15E-08 | | 11.68594 | |
| RGL1 | | -1.00675 | | 10.85959 | | -9.28326 | | | 1.66E-09 | | | 5.17E-08 | | 11.68082 | |
| PAQR8 | | -1.96903 | | 4.516673 | | -9.2696 | | | 1.71E-09 | | | 5.31E-08 | | 11.65201 | |
| TGIF2 | | -1.16019 | | 6.327805 | | -9.26343 | | | 1.73E-09 | | | 5.36E-08 | | 11.63899 | |
| AXIIR | | -2.10614 | | 5.942728 | | -9.26305 | | | 1.73E-09 | | | 5.36E-08 | | 11.63819 | |
| SFRS2 | | -1.08521 | | 11.35194 | | -9.2529 | | | 1.77E-09 | | | 5.46E-08 | | 11.61675 | |
| NFATC4 | | -1.35605 | | 5.653751 | | -9.23823 | | | 1.82E-09 | | | 5.61E-08 | | 11.58576 | |
| FLJ20489 | | 1.353269 | | 7.163045 | | 9.227673 | | | 1.86E-09 | | | 5.72E-08 | | 11.56342 | |
| PTPRB | | 2.700935 | | 3.986157 | | 10.27685 | | | 2.51E-09 | | | 7.40E-08 | | 11.56288 | |
| JUN | | -1.54381 | | 10.32602 | | -9.22514 | | | 1.87E-09 | | | 5.74E-08 | | 11.55806 | |
| TRAF4 | | -1.27466 | | 5.047675 | | -9.21831 | | | 1.90E-09 | | | 5.82E-08 | | 11.54361 | |
| SUMO2 | | -1.14593 | | 10.48453 | | -9.21038 | | | 1.93E-09 | | | 5.90E-08 | | 11.5268 | |
| MSX1 | | -1.23441 | | 10.36914 | | -9.20433 | | | 1.96E-09 | | | 5.97E-08 | | 11.51399 | |
| KNTC1 | | -1.99171 | | 6.276305 | | -9.18523 | | | 2.04E-09 | | | 6.20E-08 | | 11.4735 | |
| ARHGEF2 | | 1.039385 | | 9.947479 | | 9.154721 | | | 2.17E-09 | | | 6.57E-08 | | 11.40872 | |
| PSMB8 | | -1.24239 | | 9.040162 | | -9.1526 | | | 2.18E-09 | | | 6.59E-08 | | 11.40422 | |
| TLE4 | | -1.05719 | | 8.978816 | | -9.14502 | | | 2.21E-09 | | | 6.68E-08 | | 11.3881 | |
| ZNF416 | | 1.19262 | | 5.340645 | | 9.137303 | | | 2.25E-09 | | | 6.78E-08 | | 11.37168 | |
| ZNF606 | | 1.785095 | | 4.222705 | | 9.132112 | | | 2.28E-09 | | | 6.83E-08 | | 11.36063 | |
| C9orf25 | | 1.799995 | | 5.522818 | | 9.124667 | | | 2.31E-09 | | | 6.92E-08 | | 11.34478 | |
| KIAA1434 | | -1.55039 | | 6.039008 | | -9.1141 | | | 2.36E-09 | | | 7.06E-08 | | 11.32228 | |
| HLA-H | | -1.19554 | | 11.06607 | | -9.11289 | | | 2.37E-09 | | | 7.06E-08 | | 11.3197 | |
| KRT18 | | -1.38212 | | 5.796482 | | -9.09626 | | | 2.45E-09 | | | 7.28E-08 | | 11.28424 | |
| PLA2G4C | | 1.803772 | | 7.281776 | | 9.094891 | | | 2.46E-09 | | | 7.29E-08 | | 11.28132 | |
| HNRPC | | -1.18883 | | 8.640013 | | -9.08378 | | | 2.52E-09 | | | 7.42E-08 | | 11.25762 | |
| FTS | | 1.429957 | | 6.424522 | | 9.082251 | | | 2.53E-09 | | | 7.43E-08 | | 11.25434 | |
| CCDC45 | | -1.28232 | | 7.194229 | | -9.0725 | | | 2.58E-09 | | | 7.57E-08 | | 11.23351 | |
| GLI3 | | -1.99926 | | 4.747949 | | -9.0483 | | | 2.71E-09 | | | 7.96E-08 | | 11.18177 | |
| SRPX | | -1.09024 | | 10.55554 | | -9.03118 | | | 2.81E-09 | | | 8.21E-08 | | 11.14514 | |
| SH3PXD2A | | 1.245198 | | 11.93983 | | 9.028841 | | | 2.83E-09 | | | 8.23E-08 | | 11.14012 | |
| RAD51AP1 | | -4.70734 | | 4.719258 | | -9.17546 | | | 3.03E-09 | | | 8.80E-08 | | 11.11894 | |
| C9orf95 | | 1.003575 | | 6.628458 | | 9.006531 | | | 2.97E-09 | | | 8.62E-08 | | 11.0923 | |
| POLQ | | -4.5741 | | 4.18637 | | -9.50808 | | | 3.63E-09 | | | 1.02E-07 | | 11.04979 | |
| TAPBPL | | -1.88719 | | 4.841576 | | -8.98518 | | | 3.10E-09 | | | 8.96E-08 | | 11.04646 | |
| CDC42EP4 | | -1.24529 | | 9.180462 | | -8.98336 | | | 3.11E-09 | | | 8.98E-08 | | 11.04255 | |
| ART3 | | 2.620789 | | 3.490523 | | 9.129392 | | | 3.33E-09 | | | 9.51E-08 | | 11.02643 | |
| CBLN2 | | 2.034504 | | 5.648231 | | 8.971944 | | | 3.19E-09 | | | 9.18E-08 | | 11.01803 | |
| AKR1B10 | | 3.055209 | | 5.015801 | | 9.120661 | | | 3.39E-09 | | | 9.64E-08 | | 11.00842 | |
| AIG1 | | 1.00859 | | 7.517089 | | 8.954926 | | | 3.31E-09 | | | 9.47E-08 | | 10.98142 | |
| PSG9 | | 2.56461 | | 6.034056 | | 8.954164 | | | 3.31E-09 | | | 9.47E-08 | | 10.97978 | |
| PCF11 | | -1.22533 | | 6.712801 | | -8.95404 | | | 3.31E-09 | | | 9.47E-08 | | 10.97953 | |
| NQO2 | | 1.243246 | | 7.509229 | | 8.950118 | | | 3.34E-09 | | | 9.52E-08 | | 10.97108 | |
| CTSC | | -1.03492 | | 9.629449 | | -8.93806 | | | 3.43E-09 | | | 9.72E-08 | | 10.94511 | |
| PDGFC | | 1.094519 | | 8.328572 | | 8.935528 | | | 3.45E-09 | | | 9.76E-08 | | 10.93965 | |
| PSG6 | | 2.048274 | | 5.830949 | | 8.934522 | | | 3.45E-09 | | | 9.76E-08 | | 10.93749 | |
| ZFHX4 | | -2.29759 | | 5.820496 | | -8.93125 | | | 3.48E-09 | | | 9.81E-08 | | 10.93044 | |
| MOXD1 | | -1.30426 | | 10.01708 | | -8.92682 | | | 3.51E-09 | | | 9.89E-08 | | 10.92089 | |
| SHBG | | 1.319091 | | 5.187421 | | 8.922088 | | | 3.55E-09 | | | 9.97E-08 | | 10.91068 | |
| ZNF548 | | -1.30162 | | 6.787845 | | -8.90916 | | | 3.65E-09 | | | 1.02E-07 | | 10.88279 | |
| CDKN2A | | 2.141881 | | 7.488485 | | 8.902355 | | | 3.70E-09 | | | 1.03E-07 | | 10.86811 | |
| PBK | | -5.95533 | | 4.283604 | | -9.38778 | | | 4.53E-09 | | | 1.24E-07 | | 10.82322 | |
| C3orf58 | | 1.115498 | | 6.561818 | | 8.876334 | | | 3.91E-09 | | | 1.09E-07 | | 10.81188 | |
| PFKFB3 | | -1.03001 | | 10.82984 | | -8.86626 | | | 4.00E-09 | | | 1.11E-07 | | 10.79009 | |
| C14orf149 | | 1.012225 | | 8.107092 | | 8.861146 | | | 4.04E-09 | | | 1.12E-07 | | 10.77903 | |
| SFRS6 | | -1.59121 | | 11.14739 | | -8.85158 | | | 4.12E-09 | | | 1.14E-07 | | 10.7583 | |
| PAFAH1B3 | | -1.71711 | | 9.157238 | | -8.84305 | | | 4.20E-09 | | | 1.16E-07 | | 10.73983 | |
| CMKLR1 | | 3.047407 | | 4.190661 | | 10.36535 | | | 6.58E-09 | | | 1.72E-07 | | 10.73388 | |
| LYPLAL1 | | 1.081785 | | 8.739014 | | 8.83165 | | | 4.30E-09 | | | 1.18E-07 | | 10.71512 | |
| NAPRT1 | | -1.14499 | | 8.345165 | | -8.81958 | | | 4.41E-09 | | | 1.21E-07 | | 10.68895 | |
| NOLC1 | | -1.25918 | | 6.224409 | | -8.78646 | | | 4.74E-09 | | | 1.29E-07 | | 10.617 | |
| MIF4GD | | 1.121356 | | 5.837594 | | 8.77944 | | | 4.81E-09 | | | 1.31E-07 | | 10.60173 | |
| ZNF608 | | -2.17328 | | 4.998084 | | -8.77882 | | | 4.82E-09 | | | 1.31E-07 | | 10.60038 | |
| RARRES2 | | 3.526563 | | 11.29595 | | 8.767195 | | | 4.94E-09 | | | 1.34E-07 | | 10.57508 | |
| COL9A2 | | -2.67236 | | 5.040805 | | -8.75641 | | | 5.06E-09 | | | 1.36E-07 | | 10.5516 | |
| KNTC2 | | -2.91124 | | 4.490745 | | -8.89914 | | | 5.34E-09 | | | 1.42E-07 | | 10.54573 | |
| RICS | | -1.46229 | | 6.1058 | | -8.75363 | | | 5.09E-09 | | | 1.37E-07 | | 10.54554 | |
| LSMD1 | | 1.11443 | | 8.711897 | | 8.752423 | | | 5.10E-09 | | | 1.37E-07 | | 10.54291 | |
| PCOLCE | | -1.39651 | | 11.24931 | | -8.75153 | | | 5.11E-09 | | | 1.37E-07 | | 10.54095 | |
| FJX1 | | -1.16976 | | 8.388245 | | -8.74173 | | | 5.22E-09 | | | 1.40E-07 | | 10.51959 | |
| RAB6B | | 1.010895 | | 6.768712 | | 8.738622 | | | 5.25E-09 | | | 1.40E-07 | | 10.51282 | |
| ITGB1BP1 | | 1.016489 | | 9.660336 | | 8.720736 | | | 5.46E-09 | | | 1.45E-07 | | 10.47379 | |
| POLE2 | | -2.90444 | | 4.919799 | | -8.85979 | | | 5.79E-09 | | | 1.53E-07 | | 10.46327 | |
| RPS6KA2 | | 1.189888 | | 7.685235 | | 8.70936 | | | 5.59E-09 | | | 1.49E-07 | | 10.44895 | |
| FAM46C | | -1.91453 | | 6.14654 | | -8.70795 | | | 5.61E-09 | | | 1.49E-07 | | 10.44587 | |
| C17orf53 | | -3.13645 | | 5.385731 | | -8.69062 | | | 5.83E-09 | | | 1.54E-07 | | 10.40799 | |
| SNORD56 | | -1.90867 | | 4.320149 | | -8.68248 | | | 5.93E-09 | | | 1.56E-07 | | 10.39017 | |
| KRCC1 | | 1.017622 | | 6.976564 | | 8.678152 | | | 5.98E-09 | | | 1.57E-07 | | 10.3807 | |
| DNAJC12 | | 1.516202 | | 5.241229 | | 8.66898 | | | 6.10E-09 | | | 1.60E-07 | | 10.36062 | |
| HHAT | | 1.41269 | | 5.928308 | | 8.659833 | | | 6.23E-09 | | | 1.63E-07 | | 10.34058 | |
| KIF14 | | -3.35082 | | 4.331604 | | -8.7837 | | | 6.78E-09 | | | 1.76E-07 | | 10.30324 | |
| IRS2 | | -1.30731 | | 7.503252 | | -8.63171 | | | 6.62E-09 | | | 1.72E-07 | | 10.27889 | |
| MGC13170 | | -1.13875 | | 8.200432 | | -8.62706 | | | 6.68E-09 | | | 1.74E-07 | | 10.26869 | |
| STIL | | -2.20175 | | 6.656852 | | -8.62308 | | | 6.74E-09 | | | 1.75E-07 | | 10.25995 | |
| SMAD6 | | -2.12728 | | 5.44076 | | -8.61723 | | | 6.83E-09 | | | 1.76E-07 | | 10.2471 | |
| PINK1 | | 1.21913 | | 7.330384 | | 8.611392 | | | 6.91E-09 | | | 1.78E-07 | | 10.23427 | |
| PPFIBP1 | | 1.050547 | | 6.570632 | | 8.584682 | | | 7.33E-09 | | | 1.88E-07 | | 10.17552 | |
| EPHA5 | | -3.10202 | | 4.001159 | | -10.3319 | | | 1.24E-08 | | | 2.95E-07 | | 10.15271 | |
| PTPLA | | 1.821319 | | 9.599985 | | 8.569962 | | | 7.57E-09 | | | 1.93E-07 | | 10.1431 | |
| IFITM1 | | -1.98294 | | 9.178622 | | -8.56797 | | | 7.60E-09 | | | 1.94E-07 | | 10.1387 | |
| CKS2 | | -1.42003 | | 9.654046 | | -8.56363 | | | 7.67E-09 | | | 1.96E-07 | | 10.12915 | |
| CAMK2N1 | | 1.49164 | | 10.05228 | | 8.562788 | | | 7.68E-09 | | | 1.96E-07 | | 10.12729 | |
| MYOZ1 | | 2.672829 | | 4.728372 | | 8.544599 | | | 7.99E-09 | | | 2.03E-07 | | 10.08717 | |
| FAM64A | | -5.88206 | | 5.050589 | | -9.92804 | | | 1.26E-08 | | | 3.00E-07 | | 10.0581 | |
| ZNF10 | | -1.31184 | | 5.667829 | | -8.51895 | | | 8.45E-09 | | | 2.13E-07 | | 10.03052 | |
| TNS3 | | -1.00811 | | 12.43541 | | -8.51871 | | | 8.46E-09 | | | 2.13E-07 | | 10.03 | |
| C9orf140 | | -5.85428 | | 4.499239 | | -11.1533 | | | 1.59E-08 | | | 3.66E-07 | | 10.0235 | |
| EPHA2 | | -1.13894 | | 7.646114 | | -8.49538 | | | 8.90E-09 | | | 2.22E-07 | | 9.978401 | |
| RASD1 | | -1.85151 | | 4.450943 | | -8.48361 | | | 9.13E-09 | | | 2.28E-07 | | 9.952331 | |
| TMEM16K | | 1.410239 | | 8.196412 | | 8.483355 | | | 9.14E-09 | | | 2.28E-07 | | 9.951769 | |
| SNAI1 | | -6.29076 | | 5.058062 | | -11.7661 | | | 1.69E-08 | | | 3.86E-07 | | 9.92153 | |
| AMPD3 | | -1.60299 | | 5.262587 | | -8.46226 | | | 9.57E-09 | | | 2.37E-07 | | 9.905014 | |
| GAS6 | | 1.256951 | | 11.62397 | | 8.460389 | | | 9.61E-09 | | | 2.38E-07 | | 9.900865 | |
| ZNF322A | | 1.399537 | | 5.297641 | | 8.4478 | | | 9.88E-09 | | | 2.44E-07 | | 9.872932 | |
| FADS3 | | 1.593437 | | 4.458434 | | 8.446169 | | | 9.91E-09 | | | 2.44E-07 | | 9.869311 | |
| MAFB | | -2.37846 | | 3.430807 | | -8.88197 | | | 1.18E-08 | | | 2.84E-07 | | 9.850059 | |
| BTG2 | | -1.01508 | | 7.914739 | | -8.43376 | | | 1.02E-08 | | | 2.51E-07 | | 9.841759 | |
| RAB11FIP1 | | 1.183238 | | 8.543444 | | 8.433254 | | | 1.02E-08 | | | 2.51E-07 | | 9.84063 | |
| ILF3 | | -1.27398 | | 8.735842 | | -8.42698 | | | 1.03E-08 | | | 2.54E-07 | | 9.826683 | |
| SESTD1 | | -1.19536 | | 6.775454 | | -8.42504 | | | 1.04E-08 | | | 2.55E-07 | | 9.822377 | |
| RBM22 | | -1.39843 | | 10.64545 | | -8.4236 | | | 1.04E-08 | | | 2.55E-07 | | 9.819176 | |
| IFI44 | | -1.18696 | | 8.561435 | | -8.3944 | | | 1.11E-08 | | | 2.70E-07 | | 9.75421 | |
| ITPR3 | | -1.62839 | | 7.716654 | | -8.38604 | | | 1.13E-08 | | | 2.74E-07 | | 9.735581 | |
| SLC29A1 | | 1.5931 | | 6.223963 | | 8.381692 | | | 1.14E-08 | | | 2.76E-07 | | 9.725898 | |
| SPTY2D1 | | -1.37586 | | 7.384656 | | -8.37949 | | | 1.15E-08 | | | 2.77E-07 | | 9.720996 | |
| RGMB | | 1.611264 | | 8.530824 | | 8.371676 | | | 1.17E-08 | | | 2.81E-07 | | 9.70357 | |
| ADC | | 1.028039 | | 6.194057 | | 8.365698 | | | 1.18E-08 | | | 2.84E-07 | | 9.690237 | |
| SNRK | | -1.0013 | | 8.362847 | | -8.36469 | | | 1.19E-08 | | | 2.84E-07 | | 9.687993 | |
| USP6NL | | -1.08133 | | 6.517992 | | -8.3551 | | | 1.21E-08 | | | 2.89E-07 | | 9.666578 | |
| MEIS2 | | -1.38446 | | 8.910406 | | -8.34828 | | | 1.23E-08 | | | 2.93E-07 | | 9.651357 | |
| NINJ2 | | 4.767429 | | 5.166464 | | 8.60119 | | | 1.41E-08 | | | 3.32E-07 | | 9.616198 | |
| FLJ38159 | | 2.245461 | | 4.88457 | | 8.320202 | | | 1.31E-08 | | | 3.09E-07 | | 9.588611 | |
| EVI1 | | -2.06735 | | 8.851797 | | -8.29382 | | | 1.39E-08 | | | 3.26E-07 | | 9.529546 | |
| MYO1D | | 3.28381 | | 5.207702 | | 8.280058 | | | 1.43E-08 | | | 3.35E-07 | | 9.498712 | |
| IGFBP3 | | 3.358308 | | 10.05443 | | 8.27376 | | | 1.45E-08 | | | 3.39E-07 | | 9.484589 | |
| RRAD | | -3.29423 | | 4.358326 | | -8.39152 | | | 1.55E-08 | | | 3.59E-07 | | 9.46616 | |
| ZNF616 | | 1.236735 | | 5.623903 | | 8.262902 | | | 1.48E-08 | | | 3.47E-07 | | 9.460226 | |
| MXD3 | | -1.53823 | | 5.098388 | | -8.25987 | | | 1.49E-08 | | | 3.49E-07 | | 9.453414 | |
| RGS5 | | 2.483707 | | 6.636413 | | 8.250641 | | | 1.53E-08 | | | 3.56E-07 | | 9.432697 | |
| CPT1A | | -3.02354 | | 5.916884 | | -8.23347 | | | 1.58E-08 | | | 3.66E-07 | | 9.394105 | |
| EGR2 | | -1.44077 | | 10.52108 | | -8.23184 | | | 1.59E-08 | | | 3.66E-07 | | 9.390439 | |
| MKI67 | | -3.03369 | | 2.693893 | | -8.81971 | | | 1.99E-08 | | | 4.43E-07 | | 9.385984 | |
| FBXL2 | | 1.077333 | | 6.873819 | | 8.22633 | | | 1.61E-08 | | | 3.70E-07 | | 9.378053 | |
| CD14 | | 2.542718 | | 6.855588 | | 8.224303 | | | 1.62E-08 | | | 3.72E-07 | | 9.373495 | |
| NDUFB1 | | 1.330141 | | 5.843501 | | 8.22085 | | | 1.63E-08 | | | 3.74E-07 | | 9.365726 | |
| PTDSR | | -1.15698 | | 6.264426 | | -8.21994 | | | 1.63E-08 | | | 3.74E-07 | | 9.363681 | |
| HMMR | | -5.30559 | | 4.661659 | | -9.46962 | | | 2.56E-08 | | | 5.50E-07 | | 9.344368 | |
| CST1 | | -3.51664 | | 3.961474 | | -8.61961 | | | 1.97E-08 | | | 4.40E-07 | | 9.332001 | |
| FLJ21986 | | 1.301424 | | 9.419904 | | 8.204719 | | | 1.69E-08 | | | 3.86E-07 | | 9.329413 | |
| ATP9A | | -1.0713 | | 10.90699 | | -8.20303 | | | 1.70E-08 | | | 3.86E-07 | | 9.325616 | |
| ZSWIM3 | | 1.888739 | | 4.562293 | | 8.199997 | | | 1.71E-08 | | | 3.88E-07 | | 9.318777 | |
| ADK | | 1.752276 | | 5.263006 | | 8.19427 | | | 1.73E-08 | | | 3.93E-07 | | 9.305875 | |
| TPD52L1 | | 1.969392 | | 4.53093 | | 8.190028 | | | 1.74E-08 | | | 3.96E-07 | | 9.296314 | |
| PC | | 2.403007 | | 6.163751 | | 8.187393 | | | 1.76E-08 | | | 3.97E-07 | | 9.290374 | |
| CHAF1B | | -2.82153 | | 5.204543 | | -8.16364 | | | 1.85E-08 | | | 4.18E-07 | | 9.236778 | |
| EZH2 | | -2.13178 | | 4.911627 | | -8.16333 | | | 1.85E-08 | | | 4.18E-07 | | 9.236089 | |
| MYLIP | | -1.06081 | | 9.501744 | | -8.16198 | | | 1.86E-08 | | | 4.18E-07 | | 9.233038 | |
| NME2 | | 1.134906 | | 7.418342 | | 8.153849 | | | 1.89E-08 | | | 4.26E-07 | | 9.214674 | |
| CCNB1 | | -3.10316 | | 4.144042 | | -8.14405 | | | 1.93E-08 | | | 4.34E-07 | | 9.192528 | |
| IL6 | | -1.56156 | | 7.724446 | | -8.14319 | | | 1.94E-08 | | | 4.35E-07 | | 9.190581 | |
| FAM38B | | -2.27789 | | 7.347071 | | -8.14109 | | | 1.95E-08 | | | 4.36E-07 | | 9.185838 | |
| GZMH | | -1.70865 | | 5.442938 | | -8.13754 | | | 1.96E-08 | | | 4.39E-07 | | 9.177818 | |
| KIAA1706 | | -2.38525 | | 3.614158 | | -8.38486 | | | 2.20E-08 | | | 4.86E-07 | | 9.161161 | |
| CDT1 | | -5.99542 | | 5.468259 | | -9.98605 | | | 3.62E-08 | | | 7.42E-07 | | 9.153284 | |
| NTF3 | | 4.288354 | | 3.634526 | | 9.611376 | | | 3.49E-08 | | | 7.21E-07 | | 9.136629 | |
| KRT17 | | 1.694228 | | 6.336114 | | 8.115298 | | | 2.06E-08 | | | 4.59E-07 | | 9.127491 | |
| HIP1 | | -1.22458 | | 6.182159 | | -8.09463 | | | 2.16E-08 | | | 4.78E-07 | | 9.080663 | |
| ACYP2 | | 2.160752 | | 6.892721 | | 8.073102 | | | 2.27E-08 | | | 4.97E-07 | | 9.03184 | |
| C10orf85 | | -2.71704 | | 3.824455 | | -8.32037 | | | 2.52E-08 | | | 5.43E-07 | | 9.025863 | |
| MAPK6 | | -1.05674 | | 9.802098 | | -8.06877 | | | 2.29E-08 | | | 5.00E-07 | | 9.021998 | |
| GLTSCR2 | | -1.0421 | | 11.64987 | | -8.05837 | | | 2.34E-08 | | | 5.11E-07 | | 8.998394 | |
| HLA-DMA | | 1.291473 | | 6.403092 | | 8.056076 | | | 2.35E-08 | | | 5.13E-07 | | 8.993178 | |
| CST6 | | 4.259858 | | 4.923657 | | 9.852294 | | | 4.35E-08 | | | 8.58E-07 | | 8.989421 | |
| TPST2 | | 1.35903 | | 10.52563 | | 8.047729 | | | 2.40E-08 | | | 5.22E-07 | | 8.974209 | |
| IL7R | | 2.554837 | | 5.564852 | | 8.044893 | | | 2.41E-08 | | | 5.25E-07 | | 8.967762 | |
| PLA2G4B | | -1.30769 | | 6.554961 | | -8.04384 | | | 2.42E-08 | | | 5.25E-07 | | 8.965365 | |
| RCOR2 | | -1.54875 | | 6.462733 | | -8.03505 | | | 2.47E-08 | | | 5.34E-07 | | 8.945378 | |
| CENPM | | -5.4691 | | 4.555063 | | -8.42649 | | | 2.89E-08 | | | 6.12E-07 | | 8.944805 | |
| GMNN | | -1.31656 | | 6.617379 | | -8.0214 | | | 2.54E-08 | | | 5.48E-07 | | 8.914327 | |
| IRF7 | | -1.06383 | | 6.937489 | | -8.01592 | | | 2.58E-08 | | | 5.53E-07 | | 8.901835 | |
| FAM84B | | 2.332818 | | 8.86489 | | 8.012311 | | | 2.60E-08 | | | 5.57E-07 | | 8.893621 | |
| C20orf129 | | -5.54188 | | 4.764631 | | -9.77288 | | | 4.85E-08 | | | 9.41E-07 | | 8.861077 | |
| PSG4 | | 2.491342 | | 6.513967 | | 7.992912 | | | 2.71E-08 | | | 5.80E-07 | | 8.849411 | |
| PRO0628 | | -2.16686 | | 4.711392 | | -7.98577 | | | 2.76E-08 | | | 5.88E-07 | | 8.833133 | |
| CSRP1 | | 1.158668 | | 9.668186 | | 7.977081 | | | 2.81E-08 | | | 5.99E-07 | | 8.813296 | |
| LRRC8C | | -4.84752 | | 4.027545 | | -9.72004 | | | 5.22E-08 | | | 1.00E-06 | | 8.787846 | |
| RASL11A | | -3.04277 | | 3.260119 | | -8.50551 | | | 3.59E-08 | | | 7.37E-07 | | 8.784397 | |
| KRTHA4 | | 3.552846 | | 4.423218 | | 9.093108 | | | 4.65E-08 | | | 9.07E-07 | | 8.764306 | |
| MFAP5 | | 2.911795 | | 4.135593 | | 8.324294 | | | 3.54E-08 | | | 7.29E-07 | | 8.7467 | |
| C1orf73 | | -1.39041 | | 4.930888 | | -7.94461 | | | 3.02E-08 | | | 6.37E-07 | | 8.739124 | |
| SH2D4A | | -1.93233 | | 6.130236 | | -7.92813 | | | 3.14E-08 | | | 6.58E-07 | | 8.701414 | |
| FAM36A | | 1.166194 | | 6.979182 | | 7.902225 | | | 3.33E-08 | | | 6.95E-07 | | 8.642078 | |
| PTTG3 | | -1.83014 | | 8.313949 | | -7.90188 | | | 3.33E-08 | | | 6.95E-07 | | 8.641278 | |
| C1orf74 | | 1.017412 | | 5.240836 | | 7.894556 | | | 3.39E-08 | | | 7.06E-07 | | 8.624496 | |
| EIF4E3 | | 1.590514 | | 5.732959 | | 7.891458 | | | 3.41E-08 | | | 7.09E-07 | | 8.617389 | |
| ITPR1 | | 1.561857 | | 6.372558 | | 7.89136 | | | 3.41E-08 | | | 7.09E-07 | | 8.617165 | |
| ETS2 | | -1.44244 | | 8.103147 | | -7.88668 | | | 3.45E-08 | | | 7.15E-07 | | 8.606428 | |
| C19orf4 | | 2.746631 | | 5.816842 | | 7.881332 | | | 3.49E-08 | | | 7.21E-07 | | 8.594159 | |
| SFRS16 | | -1.68237 | | 4.76271 | | -7.87649 | | | 3.53E-08 | | | 7.28E-07 | | 8.583035 | |
| CNAP1 | | -1.1759 | | 7.468363 | | -7.87481 | | | 3.54E-08 | | | 7.29E-07 | | 8.579189 | |
| FEN1 | | -1.7038 | | 8.374046 | | -7.86708 | | | 3.60E-08 | | | 7.39E-07 | | 8.561439 | |
| AKAP8 | | -1.00215 | | 7.345335 | | -7.86229 | | | 3.64E-08 | | | 7.45E-07 | | 8.55044 | |
| PAX9 | | 2.239407 | | 4.727912 | | 7.85442 | | | 3.71E-08 | | | 7.56E-07 | | 8.53235 | |
| KRT8 | | -1.42535 | | 6.513585 | | -7.85237 | | | 3.73E-08 | | | 7.58E-07 | | 8.527643 | |
| LY6H | | 1.81552 | | 5.242752 | | 7.850342 | | | 3.74E-08 | | | 7.61E-07 | | 8.522974 | |
| LOC139886 | | -1.81166 | | 6.56079 | | -7.84647 | | | 3.78E-08 | | | 7.65E-07 | | 8.514062 | |
| LARGE | | 2.224541 | | 5.186723 | | 7.844557 | | | 3.79E-08 | | | 7.67E-07 | | 8.509671 | |
| NNAT | | -1.53566 | | 7.760861 | | -7.84001 | | | 3.83E-08 | | | 7.73E-07 | | 8.499203 | |
| NES | | 1.584792 | | 9.765125 | | 7.837564 | | | 3.85E-08 | | | 7.76E-07 | | 8.493586 | |
| NINJ1 | | -1.10602 | | 9.850331 | | -7.82073 | | | 4.00E-08 | | | 8.01E-07 | | 8.454845 | |
| PSRC1 | | -1.29827 | | 6.014384 | | -7.81447 | | | 4.06E-08 | | | 8.11E-07 | | 8.44042 | |
| BTBD5 | | 1.018064 | | 7.326052 | | 7.811943 | | | 4.08E-08 | | | 8.14E-07 | | 8.434598 | |
| LY96 | | 2.51833 | | 5.668838 | | 7.810802 | | | 4.10E-08 | | | 8.15E-07 | | 8.431968 | |
| CLCF1 | | -1.54662 | | 6.574594 | | -7.8031 | | | 4.17E-08 | | | 8.28E-07 | | 8.414227 | |
| TTK | | -5.73334 | | 4.912904 | | -9.15073 | | | 6.97E-08 | | | 1.28E-06 | | 8.41386 | |
| SMAD7 | | -2.76524 | | 6.114535 | | -7.90954 | | | 4.39E-08 | | | 8.64E-07 | | 8.409185 | |
| C22orf18 | | -5.56027 | | 4.687664 | | -9.43769 | | | 7.76E-08 | | | 1.41E-06 | | 8.391057 | |
| ZCCHC8 | | -1.04673 | | 6.545713 | | -7.78496 | | | 4.34E-08 | | | 8.58E-07 | | 8.372369 | |
| CMTM8 | | 1.755885 | | 7.333203 | | 7.774737 | | | 4.45E-08 | | | 8.71E-07 | | 8.34878 | |
| TMEM45A | | 1.405178 | | 9.862296 | | 7.767418 | | | 4.52E-08 | | | 8.85E-07 | | 8.331878 | |
| KIAA0922 | | -1.37103 | | 4.917262 | | -7.74749 | | | 4.73E-08 | | | 9.20E-07 | | 8.285816 | |
| TBX3 | | -1.30012 | | 6.212366 | | -7.73721 | | | 4.84E-08 | | | 9.40E-07 | | 8.262052 | |
| VPS37A | | 1.025435 | | 8.39357 | | 7.724652 | | | 4.98E-08 | | | 9.64E-07 | | 8.232975 | |
| TNFAIP3 | | -1.94788 | | 7.602751 | | -7.72103 | | | 5.03E-08 | | | 9.69E-07 | | 8.224582 | |
| FLJ13236 | | -2.03537 | | 4.197192 | | -7.70828 | | | 5.18E-08 | | | 9.96E-07 | | 8.195038 | |
| TGIF | | -1.87483 | | 3.851796 | | -7.70279 | | | 5.24E-08 | | | 1.00E-06 | | 8.18233 | |
| CENTA1 | | 2.162885 | | 4.730574 | | 7.702722 | | | 5.24E-08 | | | 1.00E-06 | | 8.182166 | |
| DLG7 | | -3.58024 | | 5.255022 | | -7.9134 | | | 5.92E-08 | | | 1.11E-06 | | 8.159241 | |
| C22orf16 | | 1.564698 | | 9.920837 | | 7.691578 | | | 5.38E-08 | | | 1.03E-06 | | 8.156322 | |
| CYP2S1 | | -3.83491 | | 4.060102 | | -7.69064 | | | 5.39E-08 | | | 1.03E-06 | | 8.154142 | |
| MCM10 | | -3.16615 | | 5.008787 | | -7.68124 | | | 5.51E-08 | | | 1.05E-06 | | 8.132323 | |
| TWIST1 | | -1.02999 | | 8.924882 | | -7.67682 | | | 5.56E-08 | | | 1.05E-06 | | 8.122083 | |
| CDCA4 | | -1.36785 | | 7.42628 | | -7.67618 | | | 5.57E-08 | | | 1.05E-06 | | 8.120586 | |
| LOC283537 | | 1.176193 | | 7.811772 | | 7.675864 | | | 5.57E-08 | | | 1.05E-06 | | 8.119853 | |
| KIAA1618 | | -1.31677 | | 7.131401 | | -7.673 | | | 5.61E-08 | | | 1.06E-06 | | 8.113192 | |
| TMTC4 | | -1.36207 | | 4.084964 | | -7.6658 | | | 5.70E-08 | | | 1.08E-06 | | 8.096475 | |
| D15Wsu75e | | 1.243955 | | 8.916522 | | 7.657511 | | | 5.81E-08 | | | 1.10E-06 | | 8.077219 | |
| PPM1E | | 1.468392 | | 4.976668 | | 7.652886 | | | 5.88E-08 | | | 1.10E-06 | | 8.066468 | |
| IFI44L | | -2.9922 | | 5.000059 | | -7.75573 | | | 6.17E-08 | | | 1.15E-06 | | 8.065377 | |
| RRS1 | | 1.132442 | | 7.19826 | | 7.633993 | | | 6.14E-08 | | | 1.15E-06 | | 8.022525 | |
| HMOX1 | | -2.46362 | | 6.579039 | | -7.62642 | | | 6.24E-08 | | | 1.16E-06 | | 8.004905 | |
| GNG2 | | -1.34173 | | 5.489904 | | -7.62108 | | | 6.32E-08 | | | 1.18E-06 | | 7.992467 | |
| FNDC5 | | -1.72846 | | 4.803902 | | -7.60812 | | | 6.51E-08 | | | 1.21E-06 | | 7.962278 | |
| LOC130576 | | 2.191138 | | 4.353773 | | 7.60189 | | | 6.61E-08 | | | 1.22E-06 | | 7.947747 | |
| KCNG1 | | 1.839315 | | 5.147933 | | 7.58382 | | | 6.89E-08 | | | 1.27E-06 | | 7.905598 | |
| PCDH7 | | 1.954567 | | 7.008035 | | 7.561822 | | | 7.25E-08 | | | 1.33E-06 | | 7.854231 | |
| PRDM1 | | -1.58261 | | 9.250906 | | -7.55939 | | | 7.29E-08 | | | 1.34E-06 | | 7.848538 | |
| BRCA1 | | -1.73044 | | 4.319371 | | -7.5506 | | | 7.44E-08 | | | 1.36E-06 | | 7.828003 | |
| KIF2C | | -4.5215 | | 4.506398 | | -7.65034 | | | 7.79E-08 | | | 1.41E-06 | | 7.827976 | |
| DIAPH3 | | -1.59714 | | 4.875722 | | -7.55052 | | | 7.44E-08 | | | 1.36E-06 | | 7.827817 | |
| NCOA7 | | -1.28826 | | 10.2922 | | -7.54979 | | | 7.45E-08 | | | 1.36E-06 | | 7.826116 | |
| DHRS3 | | -2.63011 | | 6.676781 | | -7.53295 | | | 7.75E-08 | | | 1.41E-06 | | 7.786723 | |
| SLC25A34 | | -3.80634 | | 3.62042 | | -7.99262 | | | 9.71E-08 | | | 1.71E-06 | | 7.773988 | |
| TMEM51 | | -1.07583 | | 9.807451 | | -7.52077 | | | 7.97E-08 | | | 1.44E-06 | | 7.758194 | |
| THY1 | | 1.417116 | | 10.29632 | | 7.517372 | | | 8.03E-08 | | | 1.45E-06 | | 7.750244 | |
| BARD1 | | -3.19023 | | 4.885581 | | -7.51564 | | | 8.06E-08 | | | 1.45E-06 | | 7.746192 | |
| MLLT11 | | 1.375439 | | 12.39173 | | 7.505098 | | | 8.26E-08 | | | 1.48E-06 | | 7.721488 | |
| GJA1 | | -1.21323 | | 11.0072 | | -7.50495 | | | 8.26E-08 | | | 1.48E-06 | | 7.721131 | |
| NEK2 | | -4.62758 | | 5.357143 | | -8.70238 | | | 1.40E-07 | | | 2.35E-06 | | 7.709748 | |
| FOXF2 | | -1.72263 | | 6.868862 | | -7.49499 | | | 8.46E-08 | | | 1.51E-06 | | 7.697778 | |
| COP1 | | -1.25294 | | 6.589939 | | -7.48964 | | | 8.56E-08 | | | 1.52E-06 | | 7.68523 | |
| A2M | | 4.135572 | | 4.736858 | | 8.233027 | | | 1.30E-07 | | | 2.21E-06 | | 7.643958 | |
| CBX4 | | -1.16168 | | 7.256651 | | -7.46984 | | | 8.96E-08 | | | 1.59E-06 | | 7.638779 | |
| GPR162 | | -1.10358 | | 7.734872 | | -7.45195 | | | 9.34E-08 | | | 1.66E-06 | | 7.596749 | |
| SYT17 | | 2.497382 | | 3.797429 | | 7.649828 | | | 1.04E-07 | | | 1.82E-06 | | 7.5919 | |
| APCDD1 | | -1.11279 | | 7.151174 | | -7.44523 | | | 9.49E-08 | | | 1.68E-06 | | 7.580937 | |
| CICE | | 1.816432 | | 5.235442 | | 7.435532 | | | 9.71E-08 | | | 1.71E-06 | | 7.558129 | |
| C6orf204 | | -1.57303 | | 5.228053 | | -7.42289 | | | 1.00E-07 | | | 1.76E-06 | | 7.528386 | |
| SUSD2 | | -4.29102 | | 4.035496 | | -8.36609 | | | 1.55E-07 | | | 2.57E-06 | | 7.519365 | |
| NNMT | | 1.887343 | | 9.597596 | | 7.413518 | | | 1.02E-07 | | | 1.79E-06 | | 7.506308 | |
| SLC9A7 | | 1.451689 | | 5.141352 | | 7.409122 | | | 1.03E-07 | | | 1.81E-06 | | 7.495952 | |
| DDAH1 | | 1.750376 | | 8.617728 | | 7.404655 | | | 1.04E-07 | | | 1.82E-06 | | 7.485428 | |
| AKT3 | | 1.198725 | | 4.181106 | | 7.391589 | | | 1.08E-07 | | | 1.88E-06 | | 7.454625 | |
| GTSE1 | | -2.20437 | | 4.379405 | | -7.48242 | | | 1.14E-07 | | | 1.96E-06 | | 7.446737 | |
| MLLT6 | | -1.13291 | | 6.398505 | | -7.38447 | | | 1.09E-07 | | | 1.90E-06 | | 7.43784 | |
| AXIN2 | | -1.45246 | | 7.002335 | | -7.37843 | | | 1.11E-07 | | | 1.92E-06 | | 7.423579 | |
| TRIM55 | | 4.326242 | | 5.331088 | | 7.378312 | | | 1.11E-07 | | | 1.92E-06 | | 7.423304 | |
| POPDC3 | | 1.225718 | | 6.899717 | | 7.369459 | | | 1.13E-07 | | | 1.95E-06 | | 7.402406 | |
| ZNF559 | | -1.376 | | 5.272892 | | -7.36345 | | | 1.15E-07 | | | 1.98E-06 | | 7.388229 | |
| CRH | | 1.468383 | | 4.410261 | | 7.361559 | | | 1.15E-07 | | | 1.98E-06 | | 7.383752 | |
| SH3MD2 | | 1.483227 | | 4.832748 | | 7.355267 | | | 1.17E-07 | | | 2.01E-06 | | 7.368887 | |
| FABP3 | | 2.12803 | | 4.987564 | | 7.351573 | | | 1.18E-07 | | | 2.02E-06 | | 7.360159 | |
| RCL1 | | -1.64507 | | 5.082171 | | -7.32151 | | | 1.27E-07 | | | 2.16E-06 | | 7.289049 | |
| ABCA3 | | 3.627609 | | 3.880389 | | 8.636159 | | | 2.51E-07 | | | 3.85E-06 | | 7.250059 | |
| SPRY2 | | -1.157 | | 8.924351 | | -7.29221 | | | 1.36E-07 | | | 2.30E-06 | | 7.219644 | |
| KIAA0683 | | -1.374 | | 5.646052 | | -7.2899 | | | 1.36E-07 | | | 2.31E-06 | | 7.214181 | |
| LRIG2 | | -1.07294 | | 5.135093 | | -7.28933 | | | 1.37E-07 | | | 2.31E-06 | | 7.212825 | |
| COL27A1 | | -3.00242 | | 4.644741 | | -7.28401 | | | 1.38E-07 | | | 2.34E-06 | | 7.200201 | |
| RACGAP1 | | -1.3201 | | 6.999775 | | -7.283 | | | 1.39E-07 | | | 2.34E-06 | | 7.197814 | |
| SLC35D2 | | -1.21102 | | 6.534035 | | -7.2793 | | | 1.40E-07 | | | 2.35E-06 | | 7.189026 | |
| CRLF1 | | 2.652683 | | 3.544248 | | 7.367602 | | | 1.47E-07 | | | 2.47E-06 | | 7.186684 | |
| ZNF493 | | 1.937016 | | 3.420158 | | 7.270376 | | | 1.43E-07 | | | 2.40E-06 | | 7.167856 | |
| LOC653170 | | -1.25039 | | 5.195336 | | -7.26127 | | | 1.46E-07 | | | 2.45E-06 | | 7.146248 | |
| KDELR3 | | 1.19547 | | 11.3804 | | 7.251345 | | | 1.49E-07 | | | 2.49E-06 | | 7.122667 | |
| FIGF | | 1.735468 | | 6.802219 | | 7.250386 | | | 1.50E-07 | | | 2.50E-06 | | 7.120389 | |
| FUT4 | | -1.00982 | | 6.311032 | | -7.24791 | | | 1.51E-07 | | | 2.51E-06 | | 7.114511 | |
| CHST1 | | 1.828743 | | 3.446476 | | 7.228845 | | | 1.57E-07 | | | 2.61E-06 | | 7.06918 | |
| EHD3 | | 1.375372 | | 5.797323 | | 7.220157 | | | 1.61E-07 | | | 2.65E-06 | | 7.048511 | |
| TNF | | -6.49561 | | 4.391971 | | -8.80583 | | | 3.26E-07 | | | 4.84E-06 | | 7.043118 | |
| ZNF537 | | -1.04087 | | 8.363742 | | -7.21732 | | | 1.62E-07 | | | 2.66E-06 | | 7.041753 | |
| LRRC17 | | 1.02083 | | 12.18063 | | 7.213046 | | | 1.63E-07 | | | 2.68E-06 | | 7.031588 | |
| CTPS | | 1.315855 | | 9.281298 | | 7.193072 | | | 1.71E-07 | | | 2.80E-06 | | 6.984013 | |
| TKT | | -1.01836 | | 11.10339 | | -7.18838 | | | 1.73E-07 | | | 2.82E-06 | | 6.972821 | |
| LOC387758 | | -1.63605 | | 5.719308 | | -7.18271 | | | 1.75E-07 | | | 2.85E-06 | | 6.959305 | |
| C10orf125 | | 1.122405 | | 6.483226 | | 7.178544 | | | 1.77E-07 | | | 2.88E-06 | | 6.949381 | |
| FAM46B | | -2.68821 | | 4.288077 | | -7.35936 | | | 1.96E-07 | | | 3.12E-06 | | 6.943834 | |
| SPRY4 | | -1.16412 | | 9.415536 | | -7.17592 | | | 1.78E-07 | | | 2.89E-06 | | 6.943125 | |
| MCM3 | | -1.7327 | | 9.101018 | | -7.17464 | | | 1.79E-07 | | | 2.90E-06 | | 6.94006 | |
| HMGB1 | | -1.96852 | | 5.491781 | | -7.17396 | | | 1.79E-07 | | | 2.90E-06 | | 6.938451 | |
| TSC22D3 | | 1.146514 | | 8.554761 | | 7.171007 | | | 1.80E-07 | | | 2.92E-06 | | 6.931405 | |
| PCK2 | | 1.932739 | | 8.444218 | | 7.167219 | | | 1.82E-07 | | | 2.94E-06 | | 6.922367 | |
| LACTB | | 1.125525 | | 8.53394 | | 7.163344 | | | 1.84E-07 | | | 2.96E-06 | | 6.913118 | |
| BTNL2 | | -2.62059 | | 3.140912 | | -7.70093 | | | 2.42E-07 | | | 3.74E-06 | | 6.912548 | |
| PSRC2 | | 1.667184 | | 5.392231 | | 7.15651 | | | 1.87E-07 | | | 3.01E-06 | | 6.896807 | |
| HMGCS1 | | -1.05955 | | 9.787877 | | -7.14855 | | | 1.90E-07 | | | 3.06E-06 | | 6.877797 | |
| FGFR1 | | 1.052002 | | 5.297648 | | 7.147119 | | | 1.91E-07 | | | 3.06E-06 | | 6.874381 | |
| MCL1 | | -1.15588 | | 9.154348 | | -7.12338 | | | 2.02E-07 | | | 3.20E-06 | | 6.817639 | |
| ENO2 | | 1.679681 | | 6.45884 | | 7.121307 | | | 2.03E-07 | | | 3.22E-06 | | 6.812685 | |
| LRP8 | | 1.093476 | | 6.570165 | | 7.116282 | | | 2.05E-07 | | | 3.24E-06 | | 6.800664 | |
| HES4 | | -1.17606 | | 9.085972 | | -7.10573 | | | 2.10E-07 | | | 3.32E-06 | | 6.775416 | |
| SIRT4 | | 1.006472 | | 5.436627 | | 7.101954 | | | 2.12E-07 | | | 3.35E-06 | | 6.766375 | |
| C1orf63 | | 1.572275 | | 5.847388 | | 7.101207 | | | 2.13E-07 | | | 3.35E-06 | | 6.764588 | |
| GLRX2 | | 1.610147 | | 7.763918 | | 7.099345 | | | 2.14E-07 | | | 3.36E-06 | | 6.760129 | |
| DNAJC9 | | -1.03533 | | 9.293433 | | -7.0926 | | | 2.17E-07 | | | 3.41E-06 | | 6.74398 | |
| SCARA3 | | -2.7564 | | 4.128888 | | -7.75889 | | | 3.08E-07 | | | 4.61E-06 | | 6.741729 | |
| PGBD3 | | 1.078304 | | 5.250596 | | 7.085411 | | | 2.21E-07 | | | 3.46E-06 | | 6.726754 | |
| S100A16 | | 1.09414 | | 10.87904 | | 7.074581 | | | 2.27E-07 | | | 3.54E-06 | | 6.700797 | |
| KIFC1 | | -5.60401 | | 6.710312 | | -8.89246 | | | 5.00E-07 | | | 6.92E-06 | | 6.691868 | |
| MGC59937 | | 1.186962 | | 4.358061 | | 7.070397 | | | 2.29E-07 | | | 3.57E-06 | | 6.690765 | |
| ANXA1 | | 1.051298 | | 12.0071 | | 7.06426 | | | 2.32E-07 | | | 3.62E-06 | | 6.676046 | |
| FYN | | 1.13962 | | 7.962571 | | 7.058602 | | | 2.35E-07 | | | 3.65E-06 | | 6.662474 | |
| COG5 | | 1.260965 | | 6.987527 | | 7.057922 | | | 2.36E-07 | | | 3.66E-06 | | 6.660843 | |
| RECK | | 1.478477 | | 7.838027 | | 7.052804 | | | 2.39E-07 | | | 3.69E-06 | | 6.64856 | |
| TMEM97 | | -2.41492 | | 9.647215 | | -7.05071 | | | 2.40E-07 | | | 3.71E-06 | | 6.643545 | |
| PGBD5 | | 1.770634 | | 5.314005 | | 7.038302 | | | 2.47E-07 | | | 3.80E-06 | | 6.613742 | |
| STS-1 | | 1.406508 | | 8.087358 | | 7.026258 | | | 2.54E-07 | | | 3.89E-06 | | 6.584805 | |
| MGP | | 7.052578 | | 4.389945 | | 7.653517 | | | 3.74E-07 | | | 5.45E-06 | | 6.567245 | |
| PRTFDC1 | | -1.00211 | | 6.70877 | | -7.01044 | | | 2.64E-07 | | | 4.02E-06 | | 6.546767 | |
| MICAL1 | | -1.26004 | | 7.690542 | | -7.00838 | | | 2.65E-07 | | | 4.04E-06 | | 6.541835 | |
| SERPINB7 | | 1.10607 | | 6.016454 | | 6.995363 | | | 2.73E-07 | | | 4.16E-06 | | 6.510503 | |
| SPAG5 | | -1.87654 | | 4.144076 | | -6.98317 | | | 2.82E-07 | | | 4.27E-06 | | 6.481153 | |
| IFI27 | | -2.78802 | | 6.448911 | | -6.98297 | | | 2.82E-07 | | | 4.27E-06 | | 6.48067 | |
| C6orf128 | | 1.845402 | | 4.107427 | | 6.973028 | | | 2.88E-07 | | | 4.36E-06 | | 6.456714 | |
| ATHL1 | | -1.33113 | | 5.749547 | | -6.97001 | | | 2.90E-07 | | | 4.39E-06 | | 6.449452 | |
| CASP1 | | -1.05802 | | 8.716138 | | -6.95702 | | | 3.00E-07 | | | 4.51E-06 | | 6.418131 | |
| PDLIM3 | | 1.406595 | | 7.045125 | | 6.950474 | | | 3.04E-07 | | | 4.57E-06 | | 6.402339 | |
| FNDC4 | | 1.2548 | | 6.556966 | | 6.948616 | | | 3.06E-07 | | | 4.59E-06 | | 6.397859 | |
| EXO1 | | -3.97268 | | 4.063968 | | -7.02032 | | | 3.26E-07 | | | 4.84E-06 | | 6.378887 | |
| DNMT1 | | -1.16667 | | 10.21253 | | -6.93776 | | | 3.14E-07 | | | 4.68E-06 | | 6.371671 | |
| JAM2 | | 1.451694 | | 7.440113 | | 6.933098 | | | 3.17E-07 | | | 4.73E-06 | | 6.360407 | |
| WNT5B | | 1.146868 | | 7.811553 | | 6.929372 | | | 3.20E-07 | | | 4.76E-06 | | 6.351411 | |
| CDCA2 | | -3.25481 | | 4.182786 | | -7.4183 | | | 4.21E-07 | | | 5.99E-06 | | 6.350222 | |
| ZNF395 | | -1.00793 | | 6.262142 | | -6.91813 | | | 3.29E-07 | | | 4.88E-06 | | 6.324254 | |
| CMAH | | 3.350451 | | 4.196931 | | 7.171386 | | | 3.88E-07 | | | 5.61E-06 | | 6.315895 | |
| CD55 | | 1.05442 | | 5.649351 | | 6.914466 | | | 3.32E-07 | | | 4.91E-06 | | 6.315404 | |
| GLTSCR1 | | -1.00279 | | 7.031015 | | -6.90994 | | | 3.35E-07 | | | 4.95E-06 | | 6.304465 | |
| MGC2654 | | 1.044078 | | 9.573694 | | 6.907663 | | | 3.37E-07 | | | 4.97E-06 | | 6.298962 | |
| LEPR | | 1.542618 | | 6.491873 | | 6.904956 | | | 3.39E-07 | | | 5.00E-06 | | 6.292418 | |
| MYH10 | | -1.23522 | | 11.43625 | | -6.89576 | | | 3.47E-07 | | | 5.09E-06 | | 6.270171 | |
| MYL9 | | 1.561033 | | 10.50026 | | 6.889285 | | | 3.52E-07 | | | 5.16E-06 | | 6.254518 | |
| GPSM2 | | -1.7227 | | 5.679784 | | -6.88841 | | | 3.53E-07 | | | 5.17E-06 | | 6.252404 | |
| FAM129B | | 1.045547 | | 9.103777 | | 6.877365 | | | 3.62E-07 | | | 5.30E-06 | | 6.22567 | |
| C8orf61 | | 1.49698 | | 6.361975 | | 6.876786 | | | 3.63E-07 | | | 5.30E-06 | | 6.22427 | |
| MAMDC1 | | 1.524494 | | 4.802109 | | 6.874924 | | | 3.65E-07 | | | 5.32E-06 | | 6.219763 | |
| ULBP1 | | 1.320669 | | 6.561456 | | 6.868678 | | | 3.70E-07 | | | 5.40E-06 | | 6.204638 | |
| IL32 | | 1.573682 | | 6.913943 | | 6.863644 | | | 3.75E-07 | | | 5.45E-06 | | 6.192445 | |
| NFIL3 | | -1.45444 | | 6.860733 | | -6.86096 | | | 3.77E-07 | | | 5.48E-06 | | 6.185932 | |
| C6orf84 | | -1.25538 | | 4.598614 | | -6.85238 | | | 3.85E-07 | | | 5.58E-06 | | 6.165144 | |
| CHKB | | -1.02876 | | 7.744994 | | -6.85173 | | | 3.85E-07 | | | 5.58E-06 | | 6.163582 | |
| PLSCR1 | | -2.57624 | | 4.516351 | | -6.91814 | | | 4.13E-07 | | | 5.89E-06 | | 6.139176 | |
| C3orf39 | | 1.107323 | | 6.372092 | | 6.8346 | | | 4.02E-07 | | | 5.75E-06 | | 6.122041 | |
| TNFSF4 | | 1.449718 | | 9.787485 | | 6.833228 | | | 4.03E-07 | | | 5.76E-06 | | 6.118713 | |
| ARHGEF6 | | 3.401077 | | 3.890557 | | 7.178871 | | | 5.06E-07 | | | 6.99E-06 | | 6.112213 | |
| AMT | | -1.94523 | | 6.379841 | | -6.81183 | | | 4.24E-07 | | | 6.03E-06 | | 6.066785 | |
| NCAPG2 | | -4.26222 | | 3.855652 | | -6.88726 | | | 4.44E-07 | | | 6.27E-06 | | 6.066488 | |
| SEMA7A | | 1.240535 | | 4.896358 | | 6.808239 | | | 4.28E-07 | | | 6.08E-06 | | 6.058057 | |
| NR4A2 | | -6.8228 | | 6.038018 | | -7.68822 | | | 7.40E-07 | | | 9.77E-06 | | 6.020663 | |
| PAFAH1B2 | | 1.092722 | | 4.561541 | | 6.782324 | | | 4.55E-07 | | | 6.41E-06 | | 5.995077 | |
| ABI3BP | | 2.144094 | | 5.894569 | | 6.781339 | | | 4.56E-07 | | | 6.42E-06 | | 5.992681 | |
| IFNA10 | | 2.57605 | | 3.419244 | | 6.85324 | | | 4.80E-07 | | | 6.69E-06 | | 5.989093 | |
| PXMP2 | | -1.15487 | | 8.217862 | | -6.77054 | | | 4.68E-07 | | | 6.57E-06 | | 5.966419 | |
| FUS | | -1.37456 | | 5.801374 | | -6.76788 | | | 4.72E-07 | | | 6.61E-06 | | 5.959943 | |
| NAB2 | | -1.27052 | | 6.164469 | | -6.76335 | | | 4.77E-07 | | | 6.66E-06 | | 5.948912 | |
| MAP1A | | 1.577303 | | 9.447505 | | 6.759247 | | | 4.81E-07 | | | 6.70E-06 | | 5.93893 | |
| ABHD5 | | 1.415165 | | 6.454239 | | 6.758635 | | | 4.82E-07 | | | 6.71E-06 | | 5.93744 | |
| TSPYL2 | | -1.32421 | | 7.351194 | | -6.75149 | | | 4.91E-07 | | | 6.82E-06 | | 5.920037 | |
| SLC25A29 | | -1.4327 | | 6.385064 | | -6.72411 | | | 5.24E-07 | | | 7.20E-06 | | 5.853332 | |
| IHPK2 | | -1.03048 | | 7.764131 | | -6.72128 | | | 5.28E-07 | | | 7.25E-06 | | 5.846426 | |
| PLEKHG4 | | -1.05534 | | 5.731286 | | -6.71032 | | | 5.42E-07 | | | 7.42E-06 | | 5.819682 | |
| C20orf102 | | 3.919158 | | 3.427809 | | 6.936043 | | | 6.47E-07 | | | 8.66E-06 | | 5.798001 | |
| RAB15 | | 1.372783 | | 5.984476 | | 6.700769 | | | 5.54E-07 | | | 7.58E-06 | | 5.796383 | |
| ABCC13 | | 1.411325 | | 4.055925 | | 7.135403 | | | 7.40E-07 | | | 9.77E-06 | | 5.796335 | |
| C10orf35 | | 2.996336 | | 4.42639 | | 6.700448 | | | 5.55E-07 | | | 7.58E-06 | | 5.795601 | |
| AK3L1 | | 2.142295 | | 4.857553 | | 6.699299 | | | 5.56E-07 | | | 7.58E-06 | | 5.792795 | |
| ATXN7L2 | | -2.09454 | | 4.526988 | | -6.68813 | | | 5.72E-07 | | | 7.76E-06 | | 5.765526 | |
| ZNF519 | | -1.8388 | | 4.249863 | | -6.68604 | | | 5.74E-07 | | | 7.79E-06 | | 5.760425 | |
| BMF | | -2.51109 | | 5.333788 | | -6.67982 | | | 5.83E-07 | | | 7.89E-06 | | 5.745216 | |
| TIMP3 | | 1.310509 | | 11.15997 | | 6.679441 | | | 5.84E-07 | | | 7.89E-06 | | 5.744297 | |
| TGFB2 | | 1.969992 | | 7.192931 | | 6.676488 | | | 5.88E-07 | | | 7.94E-06 | | 5.737083 | |
| STXBP6 | | 2.081617 | | 5.640949 | | 6.673408 | | | 5.92E-07 | | | 7.99E-06 | | 5.729557 | |
| MND1 | | -4.43177 | | 4.615287 | | -8.59754 | | | 1.29E-06 | | | 1.58E-05 | | 5.727809 | |
| KIAA0773 | | 1.699463 | | 4.534176 | | 6.6683 | | | 6.00E-07 | | | 8.08E-06 | | 5.71707 | |
| C6orf151 | | 1.020368 | | 4.994232 | | 6.663767 | | | 6.06E-07 | | | 8.16E-06 | | 5.705988 | |
| ZNF597 | | -1.88249 | | 4.25984 | | -6.65164 | | | 6.24E-07 | | | 8.38E-06 | | 5.676333 | |
| TFAP2C | | -1.21991 | | 9.499742 | | -6.64841 | | | 6.29E-07 | | | 8.43E-06 | | 5.668425 | |
| SHRM | | 1.405802 | | 8.823394 | | 6.641826 | | | 6.39E-07 | | | 8.56E-06 | | 5.652317 | |
| KLF4 | | -1.77384 | | 3.114944 | | -6.71057 | | | 6.71E-07 | | | 8.94E-06 | | 5.648345 | |
| CNNM2 | | -2.2412 | | 4.246788 | | -6.70074 | | | 6.87E-07 | | | 9.11E-06 | | 5.624966 | |
| RNF138 | | -1.92442 | | 4.448684 | | -6.862 | | | 7.61E-07 | | | 9.98E-06 | | 5.623568 | |
| Septin 4 | | -2.4633 | | 4.356676 | | -6.69897 | | | 6.90E-07 | | | 9.14E-06 | | 5.62077 | |
| TSPAN33 | | 1.756154 | | 4.192512 | | 6.621926 | | | 6.71E-07 | | | 8.94E-06 | | 5.603593 | |
| CA8 | | -4.0758 | | 3.465004 | | -6.94564 | | | 8.25E-07 | | | 1.07E-05 | | 5.601169 | |
| C6orf173 | | -2.36724 | | 6.312159 | | -6.61954 | | | 6.75E-07 | | | 8.97E-06 | | 5.597739 | |
| GCHFR | | -1.18716 | | 6.472569 | | -6.61462 | | | 6.83E-07 | | | 9.07E-06 | | 5.585699 | |
| CFD | | 1.743641 | | 3.749983 | | 6.613917 | | | 6.84E-07 | | | 9.08E-06 | | 5.583971 | |
| LPPR2 | | 1.001164 | | 7.669787 | | 6.609505 | | | 6.92E-07 | | | 9.15E-06 | | 5.573157 | |
| ABLIM2 | | 4.950134 | | 3.37593 | | 7.382701 | | | 1.25E-06 | | | 1.54E-05 | | 5.528165 | |
| EPHA4 | | 1.837581 | | 5.318196 | | 6.590371 | | | 7.24E-07 | | | 9.58E-06 | | 5.526243 | |
| TNFRSF14 | | -3.88337 | | 1.699677 | | -9.33901 | | | 1.98E-06 | | | 2.27E-05 | | 5.510949 | |
| CDH18 | | -3.70084 | | 4.461952 | | -7.24637 | | | 1.13E-06 | | | 1.40E-05 | | 5.503117 | |
| KCNK10 | | -1.36782 | | 4.585485 | | -6.57806 | | | 7.46E-07 | | | 9.82E-06 | | 5.496037 | |
| SLIT2 | | -2.11381 | | 6.185627 | | -6.57758 | | | 7.47E-07 | | | 9.82E-06 | | 5.494847 | |
| FES | | -3.57725 | | 3.30779 | | -6.79063 | | | 8.91E-07 | | | 1.15E-05 | | 5.464382 | |
| SIX4 | | 1.117722 | | 9.131712 | | 6.564891 | | | 7.71E-07 | | | 1.01E-05 | | 5.463703 | |
| TLCD1 | | 1.221709 | | 6.431129 | | 6.564645 | | | 7.71E-07 | | | 1.01E-05 | | 5.463101 | |
| PEAR1 | | -2.51933 | | 5.386256 | | -6.56215 | | | 7.76E-07 | | | 1.01E-05 | | 5.456984 | |
| OLR1 | | 2.101343 | | 8.602914 | | 6.553183 | | | 7.93E-07 | | | 1.04E-05 | | 5.434946 | |
| MICAL2 | | 1.469796 | | 5.165731 | | 6.552434 | | | 7.94E-07 | | | 1.04E-05 | | 5.433105 | |
| CDK2 | | -1.44429 | | 7.837889 | | -6.5413 | | | 8.16E-07 | | | 1.06E-05 | | 5.405751 | |
| ADAMTSL2 | | -1.51119 | | 4.264805 | | -6.53728 | | | 8.24E-07 | | | 1.07E-05 | | 5.39586 | |
| ACTA1 | | -5.93802 | | 2.421952 | | -8.28089 | | | 1.93E-06 | | | 2.23E-05 | | 5.387422 | |
| KIF4A | | -4.8689 | | 4.387083 | | -7.49006 | | | 1.53E-06 | | | 1.82E-05 | | 5.38371 | |
| LOC55565 | | -1.09251 | | 6.499979 | | -6.51717 | | | 8.66E-07 | | | 1.12E-05 | | 5.346388 | |
| CDKN2C | | -1.58457 | | 5.475742 | | -6.51691 | | | 8.66E-07 | | | 1.12E-05 | | 5.345744 | |
| PPM1D | | -1.11044 | | 7.175185 | | -6.5141 | | | 8.72E-07 | | | 1.13E-05 | | 5.33883 | |
| IRF1 | | -1.09328 | | 8.902532 | | -6.50796 | | | 8.85E-07 | | | 1.14E-05 | | 5.323728 | |
| DBNDD1 | | 1.403493 | | 7.422941 | | 6.506684 | | | 8.88E-07 | | | 1.14E-05 | | 5.320582 | |
| CTCF | | -1.04702 | | 6.887124 | | -6.50473 | | | 8.92E-07 | | | 1.15E-05 | | 5.31576 | |
| PARD6G | | -1.06017 | | 6.634918 | | -6.50277 | | | 8.97E-07 | | | 1.15E-05 | | 5.310953 | |
| KRTAP1-3 | | 2.612348 | | 3.936044 | | 6.556943 | | | 9.64E-07 | | | 1.23E-05 | | 5.284726 | |
| FAM20A | | 4.072369 | | 3.200026 | | 6.985495 | | | 1.34E-06 | | | 1.64E-05 | | 5.269084 | |
| AEBP1 | | -1.30149 | | 9.698531 | | -6.47826 | | | 9.52E-07 | | | 1.22E-05 | | 5.250552 | |
| LOC653098 | | 1.674677 | | 5.071569 | | 6.476158 | | | 9.57E-07 | | | 1.22E-05 | | 5.24538 | |
| ZNF671 | | 1.083294 | | 6.526609 | | 6.463445 | | | 9.87E-07 | | | 1.25E-05 | | 5.214033 | |
| CLDN23 | | -2.89874 | | 3.680143 | | -6.52502 | | | 1.04E-06 | | | 1.31E-05 | | 5.205342 | |
| MERTK | | 2.314783 | | 4.888948 | | 6.458163 | | | 1.00E-06 | | | 1.27E-05 | | 5.201003 | |
| ASF1B | | -4.78178 | | 3.67401 | | -7.08344 | | | 1.52E-06 | | | 1.82E-05 | | 5.195441 | |
| OR10A6 | | 3.20292 | | 3.631667 | | 6.663529 | | | 1.18E-06 | | | 1.46E-05 | | 5.189494 | |
| OSR1 | | -1.77478 | | 5.443103 | | -6.45141 | | | 1.02E-06 | | | 1.29E-05 | | 5.18435 | |
| KCNMB4 | | 3.372529 | | 3.76421 | | 6.505143 | | | 1.09E-06 | | | 1.36E-05 | | 5.160558 | |
| DIDO1 | | 1.281354 | | 5.321193 | | 6.438848 | | | 1.05E-06 | | | 1.32E-05 | | 5.153333 | |
| UTX | | -1.01396 | | 5.498735 | | -6.43801 | | | 1.05E-06 | | | 1.32E-05 | | 5.151256 | |
| ASPHD1 | | 2.331639 | | 5.198717 | | 6.436229 | | | 1.05E-06 | | | 1.33E-05 | | 5.146867 | |
| SSX2IP | | -1.52375 | | 5.986483 | | -6.43152 | | | 1.07E-06 | | | 1.34E-05 | | 5.13523 | |
| CASP2 | | -1.09319 | | 9.57077 | | -6.42628 | | | 1.08E-06 | | | 1.36E-05 | | 5.122289 | |
| IFIT2 | | -1.0665 | | 6.524052 | | -6.42352 | | | 1.09E-06 | | | 1.36E-05 | | 5.115488 | |
| PIK3R2 | | -1.0373 | | 11.49785 | | -6.4208 | | | 1.10E-06 | | | 1.37E-05 | | 5.108761 | |
| OXSM | | 1.209902 | | 4.785828 | | 6.416465 | | | 1.11E-06 | | | 1.38E-05 | | 5.098046 | |
| LOC116236 | | 1.136101 | | 5.169792 | | 6.416023 | | | 1.11E-06 | | | 1.38E-05 | | 5.096953 | |
| EIF2S2 | | 1.201206 | | 5.407432 | | 6.413361 | | | 1.12E-06 | | | 1.39E-05 | | 5.090373 | |
| PLEKHG3 | | -1.12094 | | 5.950159 | | -6.40768 | | | 1.13E-06 | | | 1.41E-05 | | 5.076329 | |
| PPL | | -2.58047 | | 3.131527 | | -6.46935 | | | 1.19E-06 | | | 1.47E-05 | | 5.071682 | |
| ADA | | -1.23899 | | 8.137441 | | -6.38667 | | | 1.19E-06 | | | 1.47E-05 | | 5.024363 | |
| CEECAM1 | | 1.070071 | | 9.456475 | | 6.386289 | | | 1.19E-06 | | | 1.48E-05 | | 5.023426 | |
| C1orf135 | | -2.13477 | | 4.430357 | | -6.59326 | | | 1.38E-06 | | | 1.67E-05 | | 5.020867 | |
| GJB2 | | -2.26316 | | 3.604887 | | -6.44679 | | | 1.25E-06 | | | 1.54E-05 | | 5.017423 | |
| TNFAIP2 | | -1.31998 | | 5.149017 | | -6.37868 | | | 1.21E-06 | | | 1.50E-05 | | 5.004607 | |
| NFIA | | -1.72592 | | 4.436092 | | -6.35182 | | | 1.30E-06 | | | 1.59E-05 | | 4.938068 | |
| CDCA7 | | -4.32076 | | 4.997903 | | -6.54835 | | | 1.53E-06 | | | 1.82E-05 | | 4.919287 | |
| PBX4 | | -2.58887 | | 3.215951 | | -6.39525 | | | 1.42E-06 | | | 1.71E-05 | | 4.89326 | |
| RP11-19J3.3 | | -2.03618 | | 3.483156 | | -6.39416 | | | 1.42E-06 | | | 1.72E-05 | | 4.890629 | |
| ZNF425 | | 1.276928 | | 5.93326 | | 6.324777 | | | 1.39E-06 | | | 1.68E-05 | | 4.871034 | |
| LANCL2 | | -1.19656 | | 6.641228 | | -6.32281 | | | 1.39E-06 | | | 1.69E-05 | | 4.866152 | |
| FLJ37440 | | -2.6303 | | 3.823598 | | -6.32149 | | | 1.40E-06 | | | 1.69E-05 | | 4.862877 | |
| GIMAP8 | | 1.871034 | | 3.933808 | | 6.305564 | | | 1.45E-06 | | | 1.75E-05 | | 4.82336 | |
| AQP11 | | 1.026785 | | 5.544545 | | 6.299588 | | | 1.48E-06 | | | 1.77E-05 | | 4.808523 | |
| PBX1 | | -1.10561 | | 5.498264 | | -6.29782 | | | 1.48E-06 | | | 1.78E-05 | | 4.804121 | |
| SYTL4 | | -1.26643 | | 5.721966 | | -6.29744 | | | 1.48E-06 | | | 1.78E-05 | | 4.803197 | |
| LFNG | | 2.049228 | | 7.248492 | | 6.295307 | | | 1.49E-06 | | | 1.79E-05 | | 4.797893 | |
| MNT | | -1.07233 | | 8.037951 | | -6.29528 | | | 1.49E-06 | | | 1.79E-05 | | 4.797814 | |
| FILIP1L | | 1.099594 | | 8.499325 | | 6.291099 | | | 1.51E-06 | | | 1.80E-05 | | 4.787442 | |
| ITPR2 | | -1.04214 | | 4.904144 | | -6.28654 | | | 1.52E-06 | | | 1.82E-05 | | 4.776121 | |
| ZNF695 | | -4.90332 | | 2.407147 | | -7.80303 | | | 3.64E-06 | | | 3.84E-05 | | 4.769507 | |
| THEM2 | | 1.023715 | | 10.09365 | | 6.279043 | | | 1.55E-06 | | | 1.84E-05 | | 4.757492 | |
| ATP8B1 | | 1.495935 | | 5.777702 | | 6.276639 | | | 1.56E-06 | | | 1.85E-05 | | 4.751516 | |
| RFC4 | | -1.21906 | | 7.50644 | | -6.27044 | | | 1.59E-06 | | | 1.88E-05 | | 4.736101 | |
| SUV420H1 | | -1.02058 | | 6.489272 | | -6.26975 | | | 1.59E-06 | | | 1.88E-05 | | 4.734399 | |
| PEG3 | | 1.310357 | | 3.806242 | | 6.266841 | | | 1.60E-06 | | | 1.89E-05 | | 4.727163 | |
| CACNA2D4 | | 1.174895 | | 5.339501 | | 6.266501 | | | 1.60E-06 | | | 1.89E-05 | | 4.726319 | |
| KRTAP4-12 | | 2.683872 | | 2.226758 | | 6.385606 | | | 1.78E-06 | | | 2.07E-05 | | 4.718848 | |
| LOC51255 | | 1.049943 | | 11.26852 | | 6.260849 | | | 1.62E-06 | | | 1.91E-05 | | 4.712263 | |
| ISG15 | | -1.01607 | | 9.71761 | | -6.25681 | | | 1.64E-06 | | | 1.93E-05 | | 4.70223 | |
| XAF1 | | -3.06514 | | 4.645093 | | -6.43408 | | | 1.97E-06 | | | 2.26E-05 | | 4.65972 | |
| GNRH1 | | 1.210615 | | 5.059667 | | 6.236447 | | | 1.72E-06 | | | 2.02E-05 | | 4.651557 | |
| DUT | | -1.17921 | | 6.103499 | | -6.22785 | | | 1.76E-06 | | | 2.06E-05 | | 4.630165 | |
| CYB5R1 | | 1.008891 | | 9.116514 | | 6.219864 | | | 1.80E-06 | | | 2.09E-05 | | 4.610268 | |
| EXOSC2 | | -1.05422 | | 6.624649 | | -6.21842 | | | 1.80E-06 | | | 2.10E-05 | | 4.606672 | |
| PPP1R14A | | 1.68973 | | 5.516672 | | 6.218326 | | | 1.80E-06 | | | 2.10E-05 | | 4.606438 | |
| HSPA12A | | 1.441357 | | 7.303285 | | 6.212918 | | | 1.83E-06 | | | 2.12E-05 | | 4.592966 | |
| DOC1 | | 1.291201 | | 9.660883 | | 6.208801 | | | 1.85E-06 | | | 2.14E-05 | | 4.58271 | |
| GPT2 | | 1.079361 | | 8.22824 | | 6.200691 | | | 1.88E-06 | | | 2.18E-05 | | 4.562499 | |
| TSC22D1 | | -1.33681 | | 7.841981 | | -6.19822 | | | 1.89E-06 | | | 2.19E-05 | | 4.556337 | |
| IGSF4 | | -1.11023 | | 8.843697 | | -6.19586 | | | 1.91E-06 | | | 2.20E-05 | | 4.550466 | |
| CLTCL1 | | 1.046766 | | 4.307224 | | 6.181829 | | | 1.97E-06 | | | 2.26E-05 | | 4.515472 | |
| H1FX | | -1.07487 | | 7.210187 | | -6.17373 | | | 2.01E-06 | | | 2.30E-05 | | 4.495281 | |
| FLJ43374 | | 3.30486 | | 2.954875 | | 6.693185 | | | 3.19E-06 | | | 3.43E-05 | | 4.480421 | |
| DLX2 | | -3.51319 | | 2.82409 | | -6.22002 | | | 2.16E-06 | | | 2.44E-05 | | 4.468998 | |
| TNNC1 | | 3.869449 | | 4.54989 | | 6.158531 | | | 2.09E-06 | | | 2.38E-05 | | 4.45734 | |
| ZNF480 | | -1.04678 | | 5.95697 | | -6.1483 | | | 2.14E-06 | | | 2.43E-05 | | 4.431807 | |
| ATAD2 | | -2.458 | | 5.111917 | | -6.14627 | | | 2.15E-06 | | | 2.44E-05 | | 4.426728 | |
| SLC7A14 | | -2.92671 | | 3.86513 | | -6.79275 | | | 3.60E-06 | | | 3.81E-05 | | 4.412935 | |
| KIAA1904 | | 1.212422 | | 6.815121 | | 6.139752 | | | 2.19E-06 | | | 2.48E-05 | | 4.410447 | |
| FLJ25416 | | -3.20905 | | 3.916948 | | -6.4708 | | | 2.91E-06 | | | 3.16E-05 | | 4.386715 | |
| POLE3 | | -1.02073 | | 10.43262 | | -6.1139 | | | 2.33E-06 | | | 2.62E-05 | | 4.345829 | |
| PARP12 | | -1.28714 | | 6.136454 | | -6.10933 | | | 2.36E-06 | | | 2.64E-05 | | 4.334401 | |
| SLC7A7 | | 1.195904 | | 6.008991 | | 6.107413 | | | 2.37E-06 | | | 2.65E-05 | | 4.329619 | |
| LOC644376 | | 3.440826 | | 4.761397 | | 6.155501 | | | 2.52E-06 | | | 2.80E-05 | | 4.314967 | |
| EDN3 | | -3.97536 | | 3.132379 | | -6.52321 | | | 3.38E-06 | | | 3.61E-05 | | 4.307005 | |
| FLJ22639 | | 1.765327 | | 3.623505 | | 6.152175 | | | 2.54E-06 | | | 2.81E-05 | | 4.306862 | |
| PPP2R5B | | 1.125209 | | 6.58047 | | 6.096553 | | | 2.44E-06 | | | 2.72E-05 | | 4.302455 | |
| RGS20 | | -1.50247 | | 5.169284 | | -6.08502 | | | 2.51E-06 | | | 2.79E-05 | | 4.273606 | |
| NME4 | | 1.009141 | | 11.51599 | | 6.079382 | | | 2.54E-06 | | | 2.82E-05 | | 4.259484 | |
| CSEN | | 1.528649 | | 4.872989 | | 6.068376 | | | 2.61E-06 | | | 2.89E-05 | | 4.231926 | |
| RFXDC2 | | 1.383665 | | 4.623281 | | 6.067485 | | | 2.62E-06 | | | 2.89E-05 | | 4.229696 | |
| PARP10 | | -1.33488 | | 5.074323 | | -6.06327 | | | 2.65E-06 | | | 2.92E-05 | | 4.219133 | |
| OSTM1 | | 1.712889 | | 6.392622 | | 6.061509 | | | 2.66E-06 | | | 2.93E-05 | | 4.21473 | |
| ACSL1 | | 1.446422 | | 6.518774 | | 6.054419 | | | 2.71E-06 | | | 2.98E-05 | | 4.196967 | |
| TMCO4 | | -1.06213 | | 4.81707 | | -6.05053 | | | 2.73E-06 | | | 3.00E-05 | | 4.187226 | |
| CXCL2 | | -4.33377 | | 4.574329 | | -6.2216 | | | 3.19E-06 | | | 3.43E-05 | | 4.173074 | |
| KIAA1199 | | -2.20511 | | 8.618775 | | -6.0389 | | | 2.81E-06 | | | 3.08E-05 | | 4.158071 | |
| GAS2L3 | | -4.08258 | | 3.871704 | | -6.0881 | | | 2.97E-06 | | | 3.22E-05 | | 4.147564 | |
| ACVRL1 | | -3.9378 | | 3.202892 | | -6.76083 | | | 5.29E-06 | | | 5.33E-05 | | 4.126678 | |
| SNORD36C | | -1.42662 | | 5.350592 | | -6.0258 | | | 2.91E-06 | | | 3.16E-05 | | 4.125226 | |
| CABC1 | | -1.41764 | | 4.637397 | | -6.02574 | | | 2.91E-06 | | | 3.16E-05 | | 4.125086 | |
| CNP | | -2.80105 | | 2.397455 | | -6.2662 | | | 3.57E-06 | | | 3.79E-05 | | 4.114381 | |
| BEX1 | | -2.32493 | | 4.829128 | | -6.07302 | | | 3.08E-06 | | | 3.32E-05 | | 4.110718 | |
| MRS2L | | 1.264029 | | 7.144251 | | 6.009882 | | | 3.02E-06 | | 3.27E-05 | | | 4.085296 | |
| SEC14L2 | | 1.073593 | | 5.275351 | | 6.004457 | | | 3.07E-06 | | 3.31E-05 | | | 4.071682 | |
| RRM2 | | -4.68116 | | 4.32284 | | -6.40809 | | | 4.26E-06 | | 4.43E-05 | | | 4.069749 | |
| MPP2 | | 2.235922 | | 3.291276 | | 6.05027 | | | 3.25E-06 | | 3.49E-05 | | | 4.058071 | |
| SLC5A3 | | 1.069654 | | 4.69896 | | 6.048093 | | | 3.27E-06 | | 3.50E-05 | | | 4.052744 | |
| C1QTNF7 | | 2.255472 | | 5.769083 | | 5.996883 | | | 3.12E-06 | | 3.37E-05 | | | 4.052671 | |
| C12orf48 | | -1.09986 | | 5.957948 | | -5.99318 | | | 3.15E-06 | | 3.39E-05 | | | 4.043385 | |
| SH3BGRL3 | | 1.364077 | | 10.87639 | | 5.977836 | | | 3.28E-06 | | 3.51E-05 | | | 4.004843 | |
| C13orf16 | | 2.168696 | | 3.940241 | | 6.025964 | | | 3.45E-06 | | 3.67E-05 | | | 3.998586 | |
| KRTAP1-1 | | 3.559542 | | 5.652953 | | 6.079942 | | | 3.63E-06 | | 3.84E-05 | | | 3.99578 | |
| B4GALNT4 | | -1.91887 | | 5.055946 | | -6.02229 | | | 3.48E-06 | | 3.70E-05 | | | 3.986602 | |
| GADD45G | | -2.39615 | | 3.422646 | | -5.96369 | | | 3.39E-06 | | 3.63E-05 | | | 3.96929 | |
| ITGA8 | | -2.77125 | | 6.221315 | | -5.9584 | | | 3.44E-06 | | 3.66E-05 | | | 3.955994 | |
| NPDC1 | | 1.155701 | | 6.886393 | | 5.95592 | | | 3.46E-06 | | 3.68E-05 | | | 3.94977 | |
| NTSR1 | | -3.38846 | | 3.584775 | | -6.33853 | | | 4.92E-06 | | 5.02E-05 | | | 3.925547 | |
| TMEM107 | | 1.413736 | | 4.65309 | | 5.944444 | | | 3.56E-06 | | 3.78E-05 | | | 3.920919 | |
| FANCG | | -1.17129 | | 6.778777 | | -5.93736 | | | 3.62E-06 | | 3.83E-05 | | | 3.903114 | |
| TSPAN10 | | 1.052581 | | 6.744004 | | 5.937242 | | | 3.63E-06 | | 3.83E-05 | | | 3.902807 | |
| HCP5 | | -2.09871 | | 4.229607 | | -5.93066 | | | 3.69E-06 | | 3.88E-05 | | | 3.886255 | |
| LARP4 | | 1.16683 | | 7.34689 | | 5.925671 | | | 3.73E-06 | | 3.93E-05 | | | 3.873697 | |
| MEOX1 | | 1.179322 | | 5.469265 | | 5.922887 | | | 3.76E-06 | | 3.95E-05 | | | 3.866691 | |
| KIAA1683 | | -1.97368 | | 4.979912 | | -5.97096 | | | 3.94E-06 | | 4.13E-05 | | | 3.860785 | |
| PIGZ | | 2.194614 | | 3.159649 | | 6.210991 | | | 5.05E-06 | | 5.14E-05 | | | 3.849479 | |
| HSPB1 | | 1.076744 | | 12.42075 | | 5.908446 | | | 3.90E-06 | | 4.08E-05 | | | 3.830345 | |
| REL | | -2.04045 | | 3.313167 | | -6.20815 | | | 5.08E-06 | | 5.17E-05 | | | 3.821852 | |
| NUP153 | | -1.08356 | | 6.755778 | | -5.89744 | | | 4.01E-06 | | 4.19E-05 | | | 3.802623 | |
| RCN3 | | 1.455369 | | 8.28832 | | 5.889058 | | | 4.09E-06 | | 4.27E-05 | | | 3.781521 | |
| OAS1 | | -2.02869 | | 4.435362 | | -5.88096 | | | 4.17E-06 | | 4.34E-05 | | | 3.761125 | |
| CDCA1 | | -4.6711 | | 3.824926 | | -8.236 | | | 1.21E-05 | | 0.000109 | | | 3.747392 | |
| HOXB3 | | -1.38585 | | 4.894882 | | -5.86037 | | | 4.39E-06 | | 4.55E-05 | | | 3.709214 | |
| SDPR | | 1.564763 | | 6.907389 | | 5.857835 | | | 4.42E-06 | | 4.58E-05 | | | 3.702832 | |
| PLAC9 | | 1.276186 | | 4.97569 | | 5.855714 | | | 4.45E-06 | | 4.60E-05 | | | 3.697484 | |
| BLM | | -3.83834 | | 2.785008 | | -6.22129 | | | 6.25E-06 | | 6.17E-05 | | | 3.681125 | |
| FLJ23514 | | 2.698553 | | 4.480901 | | 5.847864 | | | 4.53E-06 | | 4.67E-05 | | | 3.677686 | |
| VDR | | 1.421349 | | 4.632362 | | 5.838288 | | | 4.65E-06 | | 4.77E-05 | | | 3.653531 | |
| MAN1C1 | | 2.948493 | | 3.95813 | | 5.935481 | | | 5.12E-06 | | 5.20E-05 | | | 3.650735 | |
| VIPR1 | | 2.340615 | | 6.493817 | | 5.835732 | | | 4.67E-06 | | 4.80E-05 | | | 3.647084 | |
| DDEFL1 | | -1.03519 | | 7.858464 | | -5.83398 | | | 4.70E-06 | | 4.81E-05 | | | 3.642656 | |
| C3orf62 | | -1.2223 | | 4.476343 | | -5.83364 | | | 4.70E-06 | | 4.81E-05 | | | 3.641817 | |
| FRZB | | -1.81873 | | 10.70972 | | -5.82584 | | | 4.79E-06 | | 4.91E-05 | | | 3.622115 | |
| ZNF226 | | 1.456811 | | 6.610901 | | 5.824335 | | | 4.81E-06 | | 4.92E-05 | | | 3.618322 | |
| CXCL14 | | 2.502708 | | 4.092804 | | 5.969314 | | | 5.68E-06 | | 5.68E-05 | | | 3.599428 | |
| LOC197336 | | -1.14225 | | 5.078841 | | -5.81503 | | | 4.92E-06 | | 5.03E-05 | | | 3.594832 | |
| C3orf30 | | -2.03939 | | 2.58488 | | -5.85268 | | | 5.26E-06 | | 5.33E-05 | | | 3.569987 | |
| GPR3 | | -1.40067 | | 4.7219 | | -5.80178 | | | 5.09E-06 | | 5.18E-05 | | | 3.56139 | |
| SH3BP5L | | 1.02664 | | 6.95718 | | 5.796906 | | | 5.15E-06 | | 5.23E-05 | | | 3.549066 | |
| RFC2 | | -1.66744 | | 4.791079 | | -5.7918 | | | 5.22E-06 | | 5.29E-05 | | | 3.536176 | |
| FLJ20674 | | -1.62823 | | 4.41558 | | -5.78759 | | | 5.28E-06 | | 5.33E-05 | | | 3.525535 | |
| MYCN | | -2.64085 | | 5.557162 | | -5.78407 | | | 5.32E-06 | | 5.36E-05 | | | 3.51663 | |
| TNFRSF11B | | 1.239629 | | 10.5959 | | 5.782959 | | | 5.34E-06 | | 5.37E-05 | | | 3.513833 | |
| GABRR1 | | 4.196486 | | 4.395361 | | 6.481923 | | | 1.19E-05 | | 0.000108 | | | 3.505695 | |
| MGC14376 | | -1.18175 | | 9.32912 | | -5.77799 | | | 5.40E-06 | | 5.43E-05 | | | 3.501265 | |
| RGS7 | | 3.546931 | | 3.487952 | | 6.047644 | | | 7.17E-06 | | 6.93E-05 | | | 3.494287 | |
| CSDC2 | | 3.309406 | | 4.25836 | | 5.922322 | | | 6.33E-06 | | 6.24E-05 | | | 3.489894 | |
| NPAL3 | | 1.238991 | | 8.519217 | | 5.771008 | | | 5.50E-06 | | 5.51E-05 | | | 3.483627 | |
| C1R | | -1.8123 | | 6.816112 | | -5.76678 | | | 5.56E-06 | | 5.56E-05 | | | 3.472933 | |
| IL1RAP | | 2.6824 | | 5.048613 | | 5.766121 | | | 5.57E-06 | | 5.57E-05 | | | 3.471274 | |
| C1orf51 | | -2.13817 | | 4.573134 | | -5.80139 | | | 5.97E-06 | | 5.93E-05 | | | 3.443504 | |
| NLGN1 | | -1.89053 | | 3.455111 | | -5.79496 | | | 6.06E-06 | | 6.01E-05 | | | 3.427638 | |
| FLJ13912 | | -1.56723 | | 5.465709 | | -5.74592 | | | 5.86E-06 | | 5.84E-05 | | | 3.420186 | |
| DIRAS3 | | 1.765637 | | 3.348718 | | 5.788004 | | | 6.17E-06 | | 6.10E-05 | | | 3.413486 | |
| KIAA0649 | | 2.666015 | | 2.852994 | | 5.834417 | | | 6.51E-06 | | 6.39E-05 | | | 3.408176 | |
| HNRPA3 | | -1.0339 | | 6.59232 | | -5.73832 | | | 5.97E-06 | | 5.94E-05 | | | 3.400956 | |
| HELLS | | -4.01743 | | 3.910293 | | -6.08454 | | | 8.30E-06 | | 7.87E-05 | | | 3.393865 | |
| SLC43A2 | | -1.0486 | | 6.857545 | | -5.7262 | | | 6.16E-06 | | 6.10E-05 | | | 3.370295 | |
| SLC26A11 | | -1.0114 | | 6.596339 | | -5.72361 | | | 6.20E-06 | | 6.13E-05 | | | 3.363741 | |
| TMEM119 | | -1.33921 | | 12.21081 | | -5.72312 | | | 6.20E-06 | | 6.13E-05 | | | 3.362491 | |
| CDKN1C | | -1.91644 | | 5.718868 | | -5.72033 | | | 6.25E-06 | | 6.16E-05 | | | 3.355429 | |
| RPS6KA1 | | -1.10814 | | 4.369105 | | -5.71828 | | | 6.28E-06 | | 6.19E-05 | | | 3.35026 | |
| ATP13A2 | | 1.247865 | | 5.990989 | | 5.709921 | | | 6.41E-06 | | 6.31E-05 | | | 3.329088 | |
| CCL5 | | -3.3792 | | 5.025399 | | -5.69643 | | | 6.64E-06 | | 6.49E-05 | | | 3.294916 | |
| TMEM70 | | -2.59813 | | 4.121762 | | -5.729 | | | 7.13E-06 | | 6.90E-05 | | | 3.264651 | |
| C15orf48 | | 1.690689 | | 6.868473 | | 5.682456 | | | 6.87E-06 | | 6.71E-05 | | | 3.259521 | |
| TTC10 | | -1.11996 | | 5.760569 | | -5.6824 | | | 6.88E-06 | | 6.71E-05 | | | 3.25937 | |
| SSR3 | | 1.190642 | | 6.494384 | | 5.676696 | | | 6.97E-06 | | 6.79E-05 | | | 3.244926 | |
| RFC3 | | -2.61099 | | 4.338695 | | -5.67287 | | | 7.04E-06 | | 6.83E-05 | | | 3.235228 | |
| SIM2 | | -1.00873 | | 5.653708 | | -5.67222 | | | 7.05E-06 | | 6.84E-05 | | | 3.23359 | |
| ZF | | 1.055798 | | 5.483288 | | 5.670538 | | | 7.08E-06 | | 6.87E-05 | | | 3.229322 | |
| CYP39A1 | | -1.01606 | | 5.409121 | | -5.66989 | | | 7.10E-06 | | 6.87E-05 | | | 3.227669 | |
| CYP26B1 | | -1.22747 | | 5.492593 | | -5.66618 | | | 7.16E-06 | | 6.93E-05 | | | 3.218264 | |
| LOC90355 | | -1.11868 | | 6.330602 | | -5.66413 | | | 7.20E-06 | | 6.94E-05 | | | 3.213069 | |
| C21orf66 | | -1.11376 | | 8.078746 | | -5.66396 | | | 7.20E-06 | | 6.94E-05 | | | 3.212646 | |
| MXRA5 | | -1.06542 | | 10.64347 | | -5.65415 | | | 7.38E-06 | | 7.11E-05 | | | 3.187771 | |
| HNRPA1 | | -1.05656 | | 6.542069 | | -5.63538 | | | 7.74E-06 | | 7.40E-05 | | | 3.140176 | |
| MORC4 | | 1.187928 | | 5.383322 | | 5.632882 | | | 7.79E-06 | | 7.44E-05 | | | 3.133836 | |
| C6orf150 | | -1.74379 | | 3.972005 | | -5.63113 | | | 7.83E-06 | | 7.47E-05 | | | 3.129387 | |
| KHDRBS3 | | 3.481225 | | 4.38405 | | 5.714837 | | | 8.66E-06 | | 8.15E-05 | | | 3.120015 | |
| CCL13 | | 1.171751 | | 4.375792 | | 5.62562 | | | 7.93E-06 | | 7.57E-05 | | | 3.115412 | |
| SERPINA9 | | 4.142242 | | 2.939158 | | 6.076913 | | | 1.38E-05 | | 0.000122 | | | 3.100457 | |
| ACACB | | -1.41572 | | 4.926383 | | -5.61169 | | | 8.22E-06 | | 7.80E-05 | | | 3.080072 | |
| FBXO2 | | 1.678623 | | 5.006641 | | 5.611404 | | | 8.23E-06 | | 7.80E-05 | | | 3.079336 | |
| PROS1 | | 1.070011 | | 8.079058 | | 5.607957 | | | 8.30E-06 | | 7.87E-05 | | | 3.070587 | |
| TUBB3 | | -1.24207 | | 10.49533 | | -5.60205 | | | 8.42E-06 | | 7.96E-05 | | | 3.0556 | |
| EGFL7 | | 1.270849 | | 3.619417 | | 5.586919 | | | 8.75E-06 | | 8.22E-05 | | | 3.017172 | |
| NUAK2 | | -1.43958 | | 5.578852 | | -5.58435 | | | 8.81E-06 | | 8.27E-05 | | | 3.01064 | |
| TNFSF18 | | 2.158413 | | 10.01126 | | 5.58429 | | | 8.81E-06 | | 8.27E-05 | | | 3.010493 | |
| SGOL1 | | -3.96277 | | 4.260606 | | -6.35353 | | | 2.07E-05 | | 0.000174 | | | 3.008617 | |
| HLA-F | | -1.03799 | | 9.255517 | | -5.58143 | | | 8.87E-06 | | 8.33E-05 | | | 3.003229 | |
| CXXC6 | | -2.17373 | | 3.042706 | | -5.82971 | | | 1.15E-05 | | 0.000105 | | | 2.993529 | |
| ACY1L2 | | 3.090562 | | 3.622187 | | 5.755001 | | | 1.12E-05 | | 0.000102 | | | 2.975783 | |
| AVEN | | 1.073505 | | 7.364082 | | 5.568918 | | | 9.16E-06 | | 8.56E-05 | | | 2.971446 | |
| CCL8 | | 3.343121 | | 2.786759 | | 5.652797 | | | 1.01E-05 | | 9.33E-05 | | | 2.970044 | |
| CCDC49 | | -1.19307 | | 6.767634 | | -5.56508 | | | 9.25E-06 | | 8.63E-05 | | | 2.961698 | |
| FOXM1 | | -4.85978 | | 3.462962 | | -5.68971 | | | 1.09E-05 | | 9.98E-05 | | | 2.933722 | |
| TMEM129 | | 1.017254 | | 5.340195 | | 5.550868 | | | 9.59E-06 | | 8.93E-05 | | | 2.925577 | |
| LIMS2 | | 1.643983 | | 6.071163 | | 5.528542 | | | 1.01E-05 | | 9.40E-05 | | | 2.86882 | |
| RIMS3 | | 1.498574 | | 4.855742 | | 5.527354 | | | 1.02E-05 | | 9.41E-05 | | | 2.865798 | |
| HSD17B6 | | 4.830718 | | 5.321314 | | 6.200503 | | | 2.66E-05 | | 0.000218 | | | 2.847339 | |
| DIO2 | | 1.748579 | | 8.002955 | | 5.514458 | | | 1.05E-05 | | 9.68E-05 | | | 2.832999 | |
| TRIM5 | | -1.21916 | | 7.866908 | | -5.50894 | | | 1.07E-05 | | 9.80E-05 | | | 2.81897 | |
| XRCC3 | | -2.12897 | | 3.622096 | | -5.50746 | | | 1.07E-05 | | 9.83E-05 | | | 2.815189 | |
| REV3L | | -1.58725 | | 5.607416 | | -5.48567 | | | 1.13E-05 | | 0.000103 | | | 2.759739 | |
| LRRC56 | | -1.02524 | | 3.796902 | | -5.52284 | | | 1.19E-05 | | 0.000108 | | | 2.753223 | |
| C20orf55 | | -1.64385 | | 5.029168 | | -5.48089 | | | 1.14E-05 | | 0.000105 | | | 2.747581 | |
| ALDH5A1 | | 1.061765 | | 5.83234 | | 5.479166 | | | 1.15E-05 | | 0.000105 | | | 2.743195 | |
| BIN3 | | -1.36727 | | 4.572627 | | -5.47589 | | | 1.16E-05 | | 0.000106 | | | 2.734849 | |
| C10orf54 | | -1.12646 | | 5.375232 | | -5.47422 | | | 1.16E-05 | | 0.000106 | | | 2.730593 | |
| SCRG1 | | 5.485703 | | 4.63809 | | 6.024429 | | | 2.64E-05 | | 0.000216 | | | 2.714175 | |
| GBP2 | | -1.40043 | | 6.822914 | | -5.46397 | | | 1.19E-05 | | 0.000108 | | | 2.704515 | |
| P4HA1 | | 1.216825 | | 9.082097 | | 5.45636 | | | 1.22E-05 | | 0.00011 | | | 2.685129 | |
| B3GALT4 | | -1.62269 | | 5.620408 | | -5.44713 | | | 1.25E-05 | | 0.000112 | | | 2.661616 | |
| C15orf39 | | -1.99232 | | 6.019719 | | -5.44322 | | | 1.26E-05 | | 0.000113 | | | 2.651662 | |
| DNM3 | | 1.540496 | | 4.341885 | | 5.437451 | | | 1.28E-05 | | 0.000114 | | | 2.636967 | |
| GPNMB | | 1.631189 | | 8.068295 | | 5.435512 | | | 1.28E-05 | | 0.000115 | | | 2.632027 | |
| PTGS2 | | -1.35017 | | 3.967112 | | -5.51346 | | | 1.41E-05 | | 0.000124 | | | 2.625492 | |
| TNFRSF6B | | -1.64536 | | 4.310784 | | -5.42976 | | | 1.30E-05 | | 0.000116 | | | 2.617374 | |
| SAMD4A | | 1.163722 | | 6.427235 | | 5.421005 | | | 1.33E-05 | | 0.000119 | | | 2.595064 | |
| ANGPT2 | | -2.37302 | | 3.394238 | | -5.54378 | | | 1.53E-05 | | 0.000133 | | | 2.589045 | |
| CEP70 | | -1.4294 | | 4.296498 | | -5.41455 | | | 1.35E-05 | | 0.00012 | | | 2.578619 | |
| TNFRSF25 | | -1.04771 | | 5.993309 | | -5.41396 | | | 1.36E-05 | | 0.00012 | | | 2.577098 | |
| TBC1D19 | | 1.020181 | | 8.199969 | | 5.407571 | | | 1.38E-05 | | 0.000122 | | | 2.560825 | |
| AP3M2 | | 1.007691 | | 8.112633 | | 5.406156 | | | 1.38E-05 | | 0.000122 | | | 2.557219 | |
| DLL3 | | 1.628739 | | 5.836423 | | 5.405735 | | | 1.39E-05 | | 0.000122 | | | 2.556145 | |
| NID1 | | -1.12509 | | 6.047987 | | -5.40225 | | | 1.40E-05 | | 0.000123 | | | 2.547261 | |
| ST6GAL2 | | -2.98995 | | 3.868677 | | -6.24274 | | | 3.50E-05 | | 0.000276 | | | 2.54103 | |
| SMC2L1 | | -1.2236 | | 4.677354 | | -5.39574 | | | 1.42E-05 | | 0.000125 | | | 2.530668 | |
| TIGD2 | | -1.10518 | | 6.338173 | | -5.39477 | | | 1.42E-05 | | 0.000126 | | | 2.528191 | |
| REEP6 | | 1.098975 | | 5.167348 | | 5.39196 | | | 1.43E-05 | | 0.000126 | | | 2.521029 | |
| SULF2 | | -1.32654 | | 8.81466 | | -5.39195 | | | 1.44E-05 | | 0.000126 | | | 2.520995 | |
| CCL7 | | 4.518514 | | 3.951126 | | 6.6888 | | | 4.18E-05 | | 0.000324 | | | 2.503929 | |
| HCST | | 1.243181 | | 5.754534 | | 5.383547 | | | 1.47E-05 | | 0.000129 | | | 2.499577 | |
| OLFML2B | | -2.57631 | | 4.177035 | | -5.42091 | | | 1.53E-05 | | 0.000133 | | | 2.499345 | |
| FOXP4 | | -3.1693 | | 4.503639 | | -5.38328 | | | 1.47E-05 | | 0.000129 | | | 2.498886 | |
| VWA1 | | -1.10577 | | 3.968202 | | -5.38254 | | | 1.47E-05 | | 0.000129 | | | 2.497 | |
| TXNL4B | | -1.78061 | | 2.950346 | | -5.41972 | | | 1.53E-05 | | 0.000133 | | | 2.496396 | |
| DEK | | -1.39322 | | 9.424861 | | -5.37839 | | | 1.49E-05 | | 0.00013 | | | 2.486415 | |
| SBDSP | | 1.039544 | | 7.918636 | | 5.371611 | | | 1.51E-05 | | 0.000132 | | | 2.469138 | |
| LOC201725 | | -2.07168 | | 4.75241 | | -5.36875 | | | 1.52E-05 | | 0.000133 | | | 2.46183 | |
| PGM2L1 | | 1.539069 | | 7.329473 | | 5.365737 | | | 1.53E-05 | | 0.000134 | | | 2.454155 | |
| TGM4 | | -6.79218 | | -2.31578 | | -8.06764 | | | 5.64E-05 | | 0.000421 | | | 2.451252 | |
| RBBP6 | | -1.93157 | | 3.720961 | | -5.39972 | | | 1.61E-05 | | 0.00014 | | | 2.44649 | |
| C5orf5 | | -1.25666 | | 7.348121 | | -5.36229 | | | 1.55E-05 | | 0.000135 | | | 2.445354 | |
| C5 | | -1.71227 | | 5.995703 | | -5.3593 | | | 1.56E-05 | | 0.000136 | | | 2.437734 | |
| DNAJC1 | | 1.411705 | | 4.959938 | | 5.354574 | | | 1.58E-05 | | 0.000137 | | | 2.425681 | |
| TUBB2A | | 1.032068 | | 8.774496 | | 5.339028 | | | 1.64E-05 | | 0.000142 | | | 2.386015 | |
| SNORD48 | | -2.21052 | | 3.312023 | | -5.4944 | | | 2.03E-05 | | 0.000171 | | | 2.361128 | |
| CENPE | | -3.96014 | | 3.447117 | | -5.74484 | | | 2.63E-05 | | 0.000216 | | | 2.354502 | |
| DNAJB4 | | 1.979405 | | 6.658232 | | 5.321318 | | | 1.72E-05 | | 0.000148 | | | 2.340816 | |
| JMJD3 | | -2.68838 | | 3.308312 | | -5.39617 | | | 1.87E-05 | | 0.00016 | | | 2.339953 | |
| CDC25C | | -4.7874 | | 4.350358 | | -6.04612 | | | 4.77E-05 | | 0.000363 | | | 2.328525 | |
| LOC643298 | | -2.16615 | | 3.207609 | | -5.42333 | | | 2.03E-05 | | 0.000172 | | | 2.303259 | |
| LASS1 | | 1.292603 | | 4.291403 | | 5.306137 | | | 1.79E-05 | | 0.000153 | | | 2.302062 | |
| TP53INP1 | | -1.02521 | | 7.319134 | | -5.30348 | | | 1.80E-05 | | 0.000154 | | | 2.295282 | |
| OLFML3 | | -1.5001 | | 7.735726 | | -5.29956 | | | 1.82E-05 | | 0.000155 | | | 2.285267 | |
| ZNF336 | | -2.60459 | | 3.916943 | | -5.28915 | | | 1.86E-05 | | 0.000159 | | | 2.258678 | |
| GHR | | 4.21006 | | 3.725259 | | 5.591373 | | | 2.88E-05 | | 0.000233 | | | 2.253394 | |
| ACTL7B | | -2.06215 | | 3.05324 | | -5.61993 | | | 2.71E-05 | | 0.000221 | | | 2.244752 | |
| DUS3L | | -1.01718 | | 5.810077 | | -5.28241 | | | 1.90E-05 | | 0.000161 | | | 2.24148 | |
| UBE1L | | -1.97933 | | 6.59742 | | -5.27839 | | | 1.92E-05 | | 0.000163 | | | 2.231198 | |
| LONRF2 | | 3.11747 | | 3.870526 | | 5.562215 | | | 3.06E-05 | | 0.000245 | | | 2.192426 | |
| RAD9A | | -1.27483 | | 5.597499 | | -5.25904 | | | 2.01E-05 | | 0.00017 | | | 2.18178 | |
| MAPRE3 | | 1.503658 | | 7.601088 | | 5.256945 | | | 2.02E-05 | | 0.000171 | | | 2.176429 | |
| ZNF350 | | -1.62207 | | 4.513195 | | -5.25477 | | | 2.04E-05 | | 0.000172 | | | 2.170883 | |
| F12 | | 1.186614 | | 3.953705 | | 5.250605 | | | 2.06E-05 | | 0.000173 | | | 2.160229 | |
| ZNF342 | | -1.78204 | | 2.8635 | | -5.32213 | | | 2.24E-05 | | 0.000186 | | | 2.15926 | |
| PRRT2 | | -1.15262 | | 6.639319 | | -5.2496 | | | 2.06E-05 | | 0.000173 | | | 2.157664 | |
| RARRES3 | | -2.72015 | | 3.731886 | | -5.32082 | | | 2.24E-05 | | 0.000187 | | | 2.15607 | |
| NFRKB | | -1.28711 | | 3.419951 | | -5.23845 | | | 2.12E-05 | | 0.000178 | | | 2.129164 | |
| CDC25B | | -1.4729 | | 9.173562 | | -5.23768 | | | 2.13E-05 | | 0.000178 | | | 2.127193 | |
| ITGA6 | | -1.88473 | | 4.864541 | | -5.22716 | | | 2.18E-05 | | 0.000183 | | | 2.100331 | |
| BLVRB | | 1.743602 | | 7.804073 | | 5.224112 | | | 2.20E-05 | | 0.000184 | | | 2.092529 | |
| CENTB1 | | 3.265671 | | 2.830701 | | 5.448166 | | | 3.20E-05 | | 0.000255 | | | 2.059102 | |
| HSD17B1 | | 1.447123 | | 4.093799 | | 5.242534 | | | 2.38E-05 | | 0.000197 | | | 2.056852 | |
| ZCSL2 | | 1.026871 | | 8.185716 | | 5.206573 | | | 2.30E-05 | | 0.000191 | | | 2.047699 | |
| NXPH4 | | 3.537661 | | 3.39287 | | 5.351508 | | | 2.81E-05 | | 0.000228 | | | 2.046577 | |
| MYST4 | | -1.34073 | | 4.346067 | | -5.20199 | | | 2.33E-05 | | 0.000193 | | | 2.03598 | |
| CAPN3 | | -1.6998 | | 3.238097 | | -5.19164 | | | 2.39E-05 | | 0.000198 | | | 2.009515 | |
| RNF175 | | -2.04426 | | 3.215803 | | -5.32764 | | | 2.97E-05 | | 0.000239 | | | 1.974876 | |
| FOXQ1 | | -3.32163 | | 4.978436 | | -5.24554 | | | 2.70E-05 | | 0.00022 | | | 1.972023 | |
| RNPC1 | | -1.71956 | | 3.729819 | | -5.1743 | | | 2.50E-05 | | 0.000206 | | | 1.965184 | |
| DTL | | -4.88329 | | 3.744773 | | -5.52735 | | | 4.06E-05 | | 0.000316 | | | 1.954646 | |
| SPIRE2 | | -2.84153 | | 3.448211 | | -5.2677 | | | 2.94E-05 | | 0.000237 | | | 1.932459 | |
| RGS11 | | 1.41782 | | 5.400917 | | 5.15975 | | | 2.59E-05 | | 0.000213 | | | 1.927977 | |
| LOC91689 | | -1.07142 | | 4.323362 | | -5.18553 | | | 2.74E-05 | | 0.000224 | | | 1.911069 | |
| DOCK5 | | 1.003535 | | 5.643322 | | 5.136588 | | | 2.75E-05 | | 0.000224 | | | 1.868733 | |
| LYPD1 | | -1.63264 | | 7.554678 | | -5.13324 | | | 2.78E-05 | | 0.000226 | | | 1.860176 | |
| CREM | | 2.719945 | | 2.824376 | | 5.801152 | | | 5.21E-05 | | 0.000392 | | | 1.85675 | |
| TMEM54 | | 1.165563 | | 7.997232 | | 5.119102 | | | 2.88E-05 | | 0.000233 | | | 1.824001 | |
| ANKRD37 | | 1.616606 | | 5.851664 | | 5.114962 | | | 2.91E-05 | | 0.000235 | | | 1.813407 | |
| MMP11 | | 1.172136 | | 10.75051 | | 5.093623 | | | 3.07E-05 | | 0.000246 | | | 1.758809 | |
| F25965 | | 1.158684 | | 5.03192 | | 5.091159 | | 3.09E-05 | | | 0.000247 | | | 1.752504 | |
| OXCT2 | | -1.06561 | | 4.223727 | | -5.08304 | | 3.16E-05 | | | 0.000252 | | | 1.731734 | |
| SERPINA3 | | 2.036194 | | 5.279041 | | 5.110303 | | 3.31E-05 | | | 0.000262 | | | 1.725681 | |
| SOCS2 | | -1.9868 | | 6.447941 | | -5.07761 | | 3.20E-05 | | | 0.000255 | | | 1.717829 | |
| SET7 | | 2.524049 | | 1.87164 | | 5.563448 | | 6.04E-05 | | | 0.000447 | | | 1.710672 | |
| PASK | | -2.56613 | | 2.26396 | | -5.36212 | | 4.64E-05 | | | 0.000354 | | | 1.701841 | |
| ZNF589 | | 1.199786 | | 4.942361 | | 5.069426 | | 3.27E-05 | | | 0.00026 | | | 1.696885 | |
| ABCD1 | | 1.58081 | | 5.928006 | | 5.067312 | | 3.29E-05 | | | 0.000261 | | | 1.691476 | |
| C10orf10 | | 1.936072 | | 7.399185 | | 5.065097 | | 3.31E-05 | | | 0.000262 | | | 1.685807 | |
| LOC340156 | | -1.14313 | | 4.885072 | | -5.05444 | | 3.40E-05 | | | 0.000268 | | | 1.658534 | |
| BRCC2 | | 2.064553 | | 3.412135 | | 5.183407 | | 4.15E-05 | | | 0.000322 | | | 1.655556 | |
| PPFIA4 | | 3.879686 | | 3.576781 | | 5.069218 | | 3.67E-05 | | | 0.000288 | | | 1.622673 | |
| PLXNA3 | | 1.004185 | | 7.842721 | | 5.031333 | | 3.60E-05 | | | 0.000284 | | | 1.599384 | |
| PSCD2 | | -1.00074 | | 6.779173 | | -5.03084 | | 3.61E-05 | | | 0.000284 | | | 1.59812 | |
| SENP7 | | -1.26101 | | 6.807979 | | -5.0196 | | 3.71E-05 | | | 0.000291 | | | 1.569339 | |
| RAPH1 | | 2.421169 | | 4.443068 | | 5.018262 | | 3.73E-05 | | | 0.000292 | | | 1.565926 | |
| TAGLN | | 1.48297 | | 13.77626 | | 5.017427 | | 3.74E-05 | | | 0.000293 | | | 1.563789 | |
| TNNT1 | | -2.02373 | | 2.537701 | | -5.26203 | | 5.72E-05 | | | 0.000426 | | | 1.517293 | |
| TSPAN13 | | 1.662636 | | 5.328433 | | 4.994355 | | 3.96E-05 | | | 0.000309 | | | 1.504723 | |
| RAB38 | | -1.47907 | | 5.175336 | | -4.98531 | | 4.06E-05 | | | 0.000316 | | | 1.481561 | |
| IGFL3 | | 2.800697 | | 3.691307 | | 5.011098 | | 4.25E-05 | | | 0.000329 | | | 1.476879 | |
| FLJ38964 | | -1.5659 | | 3.155643 | | -5.15244 | | 5.18E-05 | | | 0.00039 | | | 1.4742 | |
| FIBCD1 | | 1.375761 | | 5.781621 | | 4.982143 | | 4.09E-05 | | | 0.000318 | | | 1.473456 | |
| PITPNC1 | | -2.09464 | | 6.25753 | | -5.00368 | | 4.33E-05 | | | 0.000334 | | | 1.455131 | |
| GPR68 | | 2.597067 | | 4.762928 | | 4.970121 | | 4.22E-05 | | | 0.000327 | | | 1.442679 | |
| INTS6 | | -1.39299 | | 3.129146 | | -4.99862 | | 4.38E-05 | | | 0.000338 | | | 1.442436 | |
| ETS1 | | -1.13236 | | 8.274168 | | -4.96533 | | 4.27E-05 | | | 0.00033 | | | 1.4304 | |
| RCHY1 | | -1.3348 | | 4.332917 | | -4.95768 | | 4.35E-05 | | | 0.000336 | | | 1.410821 | |
| SERINC2 | | 1.13792 | | 8.231933 | | 4.95584 | | 4.37E-05 | | | 0.000337 | | | 1.406113 | |
| DDN | | -2.62694 | | 2.146306 | | -5.25672 | | 6.99E-05 | | | 0.000509 | | | 1.402859 | |
| OXTR | | 1.103462 | | 10.35615 | | 4.951964 | | 4.42E-05 | | | 0.00034 | | | 1.396189 | |
| HHEX | | -1.48301 | | 8.350626 | | -4.9438 | | 4.51E-05 | | | 0.000347 | | | 1.375282 | |
| ABHD3 | | 1.059121 | | 5.607797 | | 4.938441 | | 4.57E-05 | | | 0.000351 | | | 1.361565 | |
| MGC99813 | | -1.22818 | | 4.108019 | | -4.93143 | | 4.66E-05 | | | 0.000356 | | | 1.343604 | |
| NUF2 | | -3.25797 | | 2.459065 | | -5.26874 | | 8.37E-05 | | | 0.000593 | | | 1.326218 | |
| RAP1GA1 | | 2.394982 | | 4.386662 | | 4.921022 | | 4.78E-05 | | | 0.000364 | | | 1.316965 | |
| NRP2 | | -1.11718 | | 5.18645 | | -4.91983 | | 4.80E-05 | | | 0.000365 | | | 1.313915 | |
| APOBEC3B | | -4.83823 | | 5.521066 | | -5.39218 | | 0.000138 | | | 0.000923 | | | 1.299123 | |
| ABCA4 | | 1.4543 | | 4.990255 | | 4.912453 | | 4.89E-05 | | | 0.000371 | | | 1.295025 | |
| C6orf203 | | 1.269643 | | 4.85415 | | 4.908974 | | 4.93E-05 | | | 0.000374 | | | 1.286116 | |
| GBP1 | | -1.00079 | | 8.333757 | | -4.89949 | | 5.06E-05 | | | 0.000382 | | | 1.261842 | |
| ENDOGL1 | | 1.135911 | | 8.77416 | | 4.89742 | | 5.08E-05 | | | 0.000384 | | | 1.256535 | |
| ZBTB2 | | -2.13423 | | 4.527151 | | -4.92319 | | 5.30E-05 | | | 0.000398 | | | 1.253091 | |
| WDR62 | | -3.656 | | 2.287523 | | -5.25228 | | 8.64E-05 | | | 0.00061 | | | 1.239877 | |
| FLJ45032 | | -1.63095 | | 3.078997 | | -4.97485 | | 5.91E-05 | | | 0.000438 | | | 1.230941 | |
| RAB7B | | -1.40933 | | 3.6214 | | -4.9313 | | 5.82E-05 | | | 0.000432 | | | 1.201109 | |
| PCNA | | -1.04531 | | 9.057796 | | -4.86882 | | 5.47E-05 | | | 0.00041 | | | 1.183299 | |
| C8orf13 | | -1.16805 | | 5.165862 | | -4.86647 | | 5.50E-05 | | | 0.000412 | | | 1.17729 | |
| LOXL4 | | 1.324654 | | 10.78942 | | 4.860904 | | 5.58E-05 | | | 0.000417 | | | 1.163043 | |
| CCDC85A | | -3.55732 | | 1.696087 | | -6.21351 | | 0.000193 | | | 0.001226 | | | 1.141772 | |
| TNFRSF10D | | 1.162454 | | 7.556266 | | 4.851518 | | 5.72E-05 | | | 0.000426 | | | 1.139011 | |
| LOC654146 | | -3.3079 | | 0.789324 | | -5.77344 | | 0.000145 | | | 0.000962 | | | 1.116048 | |
| GDPD2 | | 1.970266 | | 3.443474 | | 4.839793 | | 5.89E-05 | | | 0.000437 | | | 1.108995 | |
| MYH4 | | 3.121001 | | 4.118052 | | 4.835186 | | 5.96E-05 | | | 0.000442 | | | 1.0972 | |
| MAP6 | | 1.345495 | | 4.69622 | | 4.806375 | | 6.42E-05 | | | 0.000472 | | | 1.023447 | |
| CPXM | | -2.82062 | | 3.280622 | | -4.85409 | | 7.04E-05 | | | 0.000511 | | | 1.01124 | |
| PDGFA | | 1.514578 | | 6.607322 | | 4.79221 | | 6.66E-05 | | | 0.000488 | | | 0.987191 | |
| NRM | | -1.20535 | | 5.770538 | | -4.78833 | | 6.73E-05 | | | 0.000493 | | | 0.97725 | |
| KIF18A | | -5.12076 | | 4.269719 | | -5.1665 | | 0.000158 | | | 0.001035 | | | 0.971888 | |
| BMP7 | | -1.7114 | | 5.333644 | | -4.78479 | | 6.79E-05 | | | 0.000496 | | | 0.968199 | |
| PPP1R13L | | 1.029522 | | 6.000434 | | 4.781331 | | 6.85E-05 | | | 0.0005 | | | 0.959345 | |
| CHST6 | | 1.665299 | | 4.273446 | | 4.778322 | | 6.90E-05 | | | 0.000503 | | | 0.951646 | |
| CENPJ | | -1.34344 | | 5.074774 | | -4.77044 | | 7.04E-05 | | | 0.000512 | | | 0.931465 | |
| MT1F | | -1.07805 | | 5.641817 | | -4.77001 | | 7.05E-05 | | | 0.000512 | | | 0.930367 | |
| RPS6KC1 | | 1.005921 | | 6.323641 | | 4.766956 | | 7.11E-05 | | | 0.000516 | | | 0.922558 | |
| CORO2B | | 1.26971 | | 4.964071 | | 4.759416 | | 7.25E-05 | | | 0.000524 | | | 0.903261 | |
| COQ7 | | -1.27519 | | 4.588582 | | -4.75883 | | 7.26E-05 | | | 0.000524 | | | 0.901769 | |
| AGTRAP | | 1.147181 | | 6.488248 | | 4.750495 | | 7.41E-05 | | | 0.000534 | | | 0.880435 | |
| CMA1 | | -3.0176 | | 0.837662 | | -7.76928 | | 0.000345 | | | 0.002023 | | | 0.873809 | |
| KRT16 | | 2.901282 | | 2.851285 | | 4.790045 | | 8.24E-05 | | | 0.000584 | | | 0.860546 | |
| SNN | | -1.03094 | | 4.000214 | | -4.74098 | | 7.60E-05 | | | 0.000546 | | | 0.856082 | |
| CAMK1 | | 1.003696 | | 6.491203 | | 4.737432 | | 7.67E-05 | | | 0.000549 | | | 0.847009 | |
| ADAM18 | | -7.58022 | | 0.455272 | | -16.0889 | | 0.000187 | | | 0.001194 | | | 0.84654 | |
| S100A3 | | 1.150176 | | 7.329713 | | 4.731198 | | 7.79E-05 | | | 0.000557 | | | 0.831061 | |
| HMGN2 | | -1.05477 | | 9.941939 | | -4.72996 | | 7.82E-05 | | | 0.000558 | | | 0.827885 | |
| USP1 | | -1.23035 | | 6.867569 | | -4.72954 | | 7.82E-05 | | | 0.000558 | | | 0.82682 | |
| HIPK2 | | -1.02562 | | 7.400513 | | -4.72678 | | 7.88E-05 | | | 0.000562 | | | 0.819745 | |
| ASB7 | | -1.0405 | | 4.839999 | | -4.70027 | | 8.44E-05 | | | 0.000597 | | | 0.751952 | |
| SLC41A2 | | 1.739313 | | 4.33901 | | 4.720886 | | 8.82E-05 | | | 0.00062 | | | 0.748278 | |
| MYOZ3 | | -2.36933 | | 3.571338 | | -4.72114 | | 8.81E-05 | | | 0.00062 | | | 0.745744 | |
| NOTCH1 | | -1.51093 | | 6.801154 | | -4.69655 | | 8.52E-05 | | | 0.000603 | | | 0.742429 | |
| GRAMD3 | | 1.205191 | | 9.472892 | | 4.694825 | | 8.55E-05 | | | 0.000605 | | | 0.738017 | |
| LRRC45 | | -1.51733 | | 4.597563 | | -4.68835 | | 8.70E-05 | | | 0.000614 | | | 0.721449 | |
| LRRC23 | | 1.023827 | | 4.717578 | | 4.685297 | | 8.77E-05 | | | 0.000618 | | | 0.713652 | |
| RAP2B | | -2.03108 | | 1.67001 | | -5.15691 | | 0.000161 | | | 0.00105 | | | 0.702006 | |
| ARG1 | | -3.86971 | | -0.63941 | | -7.41605 | | 0.000438 | | | 0.002489 | | | 0.691847 | |
| TMEM89 | | -1.58252 | | 2.710356 | | -5.11051 | | 0.000175 | | | 0.00113 | | | 0.689272 | |
| TRIB3 | | 1.257888 | | 8.841564 | | 4.666635 | | 9.20E-05 | | | 0.000643 | | | 0.665931 | |
| C20orf23 | | 1.188529 | | 4.801984 | | 4.659048 | | 9.38E-05 | | | 0.000655 | | | 0.646535 | |
| ZNF767 | | 1.067487 | | 5.274986 | | 4.651321 | | 9.57E-05 | | | 0.000667 | | | 0.62678 | |
| ABCA2 | | 1.114921 | | 5.105152 | | 4.65031 | | 9.59E-05 | | | 0.000668 | | | 0.624197 | |
| EGFL9 | | 1.326646 | | 4.613972 | | 4.645782 | | 9.70E-05 | | | 0.000675 | | | 0.61262 | |
| FLJ32063 | | 1.137791 | | 4.342566 | | 4.644177 | | 9.74E-05 | | | 0.000678 | | | 0.608519 | |
| FRMPD4 | | -1.59783 | | 3.178961 | | -4.66387 | | 0.000102 | | | 0.000704 | | | 0.601985 | |
| TNS1 | | 1.275892 | | 6.06555 | | 4.628545 | | 0.000101 | | | 0.000702 | | | 0.568567 | |
| ZNF71 | | -1.49783 | | 3.088639 | | -4.66477 | | 0.000112 | | | 0.000766 | | | 0.545368 | |
| ENC1 | | 1.405741 | | 8.03584 | | 4.617739 | | 0.000104 | | | 0.00072 | | | 0.540952 | |
| MFHAS1 | | -1.07712 | | 3.683537 | | -4.61489 | | 0.000105 | | | 0.000725 | | | 0.533683 | |
| UNG2 | | 1.323471 | | 4.639211 | | 4.609957 | | 0.000106 | | | 0.000732 | | | 0.521068 | |
| MTUS1 | | -2.24667 | | 5.454133 | | -4.60647 | | 0.000107 | | | 0.000738 | | | 0.512148 | |
| VMD2 | | 2.167002 | | 3.630151 | | 4.622576 | | 0.000113 | | | 0.000771 | | | 0.501537 | |
| EDARADD | | 1.30498 | | 4.937633 | | 4.601678 | | 0.000109 | | | 0.000746 | | | 0.499918 | |
| LOC56901 | | 2.267268 | | 3.055811 | | 4.71481 | | 0.00014 | | | 0.000934 | | | 0.49747 | |
| SCG2 | | -2.39453 | | 7.578339 | | -4.59858 | | 0.00011 | | | 0.000752 | | | 0.492015 | |
| KAZALD1 | | 1.157317 | | 5.63727 | | 4.594708 | | 0.000111 | | | 0.000758 | | | 0.482113 | |
| XPNPEP2 | | -1.42778 | | 3.745571 | | -4.59344 | | 0.000111 | | | 0.000759 | | | 0.478866 | |
| CREG2 | | 3.19068 | | 1.02151 | | 5.704056 | | 0.000352 | | | 0.002064 | | | 0.459404 | |
| HOMER2 | | 2.016867 | | 2.965565 | | 4.581959 | | 0.000114 | | | 0.00078 | | | 0.449551 | |
| ZCSL3 | | 1.349326 | | 7.353691 | | 4.574923 | | 0.000116 | | | 0.000794 | | | 0.431585 | |
| C22orf25 | | 1.031078 | | 6.961428 | | 4.568021 | | 0.000119 | | | 0.000808 | | | 0.413961 | |
| CNTN1 | | 2.163716 | | 5.971051 | | 4.565164 | | 0.000119 | | | 0.000813 | | | 0.406666 | |
| SERPINF1 | | -1.0341 | | 8.054578 | | -4.5637 | | 0.00012 | | | 0.000815 | | | 0.402934 | |
| CSS3 | | 1.046435 | | 4.891148 | | 4.554632 | | 0.000123 | | | 0.00083 | | | 0.379778 | |
| NFYA | | -1.10459 | | 5.050219 | | -4.5543 | | 0.000123 | | | 0.000831 | | | 0.378932 | |
| TLE3 | | -1.30325 | | 4.37445 | | -4.57454 | | 0.000127 | | | 0.000859 | | | 0.377864 | |
| KIAA1914 | | 1.295374 | | 3.236691 | | 4.551979 | | 0.000124 | | | 0.000835 | | | 0.373006 | |
| ANKRD33 | | 1.017277 | | 7.981889 | | 4.548553 | | 0.000125 | | | 0.000841 | | | 0.364263 | |
| LOC91431 | | -1.29506 | | 3.291395 | | -4.54814 | | 0.000125 | | | 0.000842 | | | 0.363216 | |
| CT45-4 | | 2.828236 | | 2.694688 | | 4.744677 | | 0.000236 | | | 0.001459 | | | 0.360282 | |
| GSG2 | | -3.33604 | | 2.950842 | | -4.8057 | | 0.000251 | | | 0.001538 | | | 0.346725 | |
| C6orf188 | | 3.509606 | | 3.260294 | | 4.742377 | | 0.000283 | | | 0.001711 | | | 0.342756 | |
| LAG3 | | -2.15011 | | 2.73939 | | -4.55651 | | 0.000133 | | | 0.000895 | | | 0.332645 | |
| BST1 | | -1.56205 | | 4.042548 | | -4.53167 | | 0.00013 | | | 0.000875 | | | 0.321168 | |
| LIF | | 1.877712 | | 5.676592 | | 4.525253 | | 0.000132 | | | 0.000889 | | | 0.304803 | |
| REEP2 | | 1.48554 | | 6.940491 | | 4.523647 | | 0.000133 | | | 0.000892 | | | 0.300706 | |
| SNORA32 | | 1.688059 | | 3.547331 | | 4.528723 | | 0.000143 | | | 0.000952 | | | 0.266187 | |
| NDST1 | | -1.11183 | | 6.88716 | | -4.48816 | | 0.000146 | | | 0.000964 | | | 0.210207 | |
| C6orf79 | | 1.018028 | | 8.513569 | | 4.483647 | | 0.000147 | | | 0.000974 | | | 0.198696 | |
| GPR18 | | 4.056507 | | 3.845397 | | 4.664219 | | 0.000278 | | | 0.001683 | | | 0.197626 | |
| TENC1 | | -1.23535 | | 3.617799 | | -4.48293 | | 0.000148 | | | 0.000975 | | | 0.196855 | |
| PDLIM4 | | 1.272248 | | 5.40713 | | 4.481343 | | 0.000148 | | | 0.000978 | | | 0.192821 | |
| UNQ9433 | | 2.653405 | | 2.199571 | | 5.706037 | | 0.000554 | | | 0.003041 | | | 0.179268 | |
| DPP4 | | -2.48124 | | 3.519543 | | -4.47461 | | 0.000151 | | | 0.000993 | | | 0.175655 | |
| CKB | | 1.719281 | | 5.977927 | | 4.468963 | | 0.000153 | | | 0.001006 | | | 0.161268 | |
| HSPA2 | | 1.129135 | | 7.315841 | | 4.461518 | | 0.000156 | | | 0.001022 | | | 0.142296 | |
| ZNHIT2 | | 1.18254 | | 5.529603 | | 4.459852 | | 0.000157 | | | 0.001026 | | | 0.138051 | |
| FBLN1 | | -1.20406 | | 11.00896 | | -4.45899 | | 0.000157 | | | 0.001027 | | | 0.135845 | |
| TUB | | 1.113386 | | 6.092683 | | 4.458326 | | 0.000157 | | | 0.001028 | | | 0.134162 | |
| FLJ10292 | | -3.1601 | | 3.222983 | | -4.66748 | | 0.000276 | | | 0.001673 | | | 0.120897 | |
| KYNU | | -2.04474 | | 5.252108 | | -4.45094 | | 0.00016 | | | 0.001045 | | | 0.115348 | |
| IFIH1 | | -1.04075 | | 5.819156 | | -4.45092 | | 0.00016 | | | 0.001045 | | | 0.115285 | |
| TNFRSF21 | | -1.03242 | | 12.29285 | | -4.44732 | | 0.000162 | | | 0.001053 | | | 0.106118 | |
| SOX5 | | -3.38112 | | 0.981103 | | -4.90333 | | 0.000318 | | | 0.001892 | | | 0.103447 | |
| CYP46A1 | | 1.490788 | | 3.669266 | | 4.494902 | | 0.000188 | | | 0.0012 | | | 0.086902 | |
| EAF2 | | 1.487357 | | 2.389551 | | 4.492214 | | 0.000189 | | | 0.001207 | | | 0.080429 | |
| CHPT1 | | 1.277927 | | 6.949276 | | 4.434071 | | 0.000167 | | | 0.001087 | | | 0.072382 | |
| MYC | | -1.5216 | | 6.497148 | | -4.42037 | | 0.000173 | | | 0.00112 | | | 0.037491 | |
| ANKRD41 | | -1.76016 | | 2.550303 | | -4.41463 | | 0.000176 | | | 0.001134 | | | 0.022895 | |
| APH1B | | 1.006856 | | 8.1508 | | 4.409741 | | 0.000178 | | | 0.001146 | | | 0.010447 | |
| RP13-15M17.2 -1.84312 | | | | 3.054704 | | -4.49623 | | 0.000232 | | | 0.001435 | | | -0.02886 | |
| ZNF521 | | -1.17345 | | 6.195563 | | -4.39316 | | 0.000186 | | | 0.001188 | | | | -0.03175 |
| DOCK6 | | 1.09803 | | 6.86822 | | 4.389671 | | 0.000187 | | | 0.001197 | | | | -0.04062 |
| MASTL | | -1.71116 | | 3.752407 | | -4.4055 | | 0.000195 | | | 0.001241 | | | | -0.04553 |
| E2F2 | | -5.065 | | 5.095903 | | -4.60135 | | 0.000373 | | | 0.002164 | | | | -0.05154 |
| MDFIC | | -1.27781 | | 6.408892 | | -4.38442 | | 0.00019 | | | 0.00121 | | | | -0.05396 |
| FLJ20105 | | -4.99804 | | 2.873421 | | -4.80327 | | 0.000617 | | | 0.003345 | | | | -0.05672 |
| ADCY4 | | -2.86069 | | 3.903331 | | -4.5255 | | 0.000277 | | | 0.001678 | | | | -0.07201 |
| KIF1C | | 1.027102 | | 6.655302 | | 4.376031 | | 0.000194 | | | 0.001235 | | | | -0.07531 |
| CSPG5 | | -1.98099 | | 1.994173 | | -4.42823 | | 0.000221 | | | 0.001379 | | | | -0.08895 |
| MRVI1 | | -1.01249 | | 4.514559 | | -4.36855 | | 0.000198 | | | 0.001255 | | | | -0.09433 |
| COL8A2 | | 2.076775 | | 7.351951 | | 4.367763 | | 0.000198 | | | 0.001257 | | | | -0.09633 |
| CISH | | -3.46699 | | 1.945568 | | -4.55644 | | 0.000587 | | | 0.003198 | | | | -0.10887 |
| IL5 | | -2.00215 | | 0.868639 | | -7.20995 | | 0.001168 | | | 0.005717 | | | | -0.12081 |
| RECQL4 | | -1.8902 | | 3.021358 | | -4.41242 | | 0.00023 | | | 0.001425 | | | | -0.12322 |
| FLJ23356 | | 1.794986 | | 2.168632 | | 4.694607 | | 0.000375 | | | 0.002173 | | | | -0.13171 |
| C8orf4 | | 2.462737 | | 2.73123 | | 4.415403 | | 0.000251 | | | 0.001542 | | | | -0.14818 |
| MGC15763 | | 1.049207 | | 4.671495 | | 4.340274 | | 0.000213 | | | 0.001336 | | | | -0.16618 |
| PAQR4 | | -1.07823 | | 6.932362 | | -4.3374 | | 0.000214 | | | 0.001344 | | | | -0.17349 |
| F13A1 | | -4.12331 | | 0.20649 | | -9.40909 | | 0.001217 | | | 0.005926 | | | | -0.18819 |
| KIAA1324 | | 1.343232 | | 4.179007 | | 4.33055 | | 0.000218 | | | 0.001365 | | | | -0.19088 |
| DCDC2 | | 2.134549 | | 4.38599 | | 4.394796 | | 0.000264 | | | 0.001609 | | | | -0.19672 |
| SARM1 | | -1.07421 | | 5.994676 | | -4.32771 | | 0.00022 | | | 0.001372 | | | | -0.19809 |
| C1orf21 | | -1.93066 | | 2.889122 | | -4.33808 | | 0.000231 | | | 0.001434 | | | | -0.21402 |
| EREG | | 1.276756 | | 5.386438 | | 4.319494 | | 0.000225 | | | 0.001398 | | | | -0.21895 |
| LOC63920 | | 1.483673 | | 4.643688 | | 4.314299 | | 0.000228 | | | 0.001415 | | | | -0.23213 |
| AADAT | | -9.58157 | | -1.75662 | | -9.23194 | | 0.001299 | | | 0.006255 | | | | -0.23537 |
| PLA2G12A | | 1.31462 | | 4.338664 | | 4.301476 | | 0.000235 | | | 0.001453 | | | | -0.26467 |
| PCDHA4 | | -1.20918 | | 3.92324 | | -4.29721 | | 0.000238 | | | 0.001466 | | | | -0.27549 |
| C1orf188 | | -1.51485 | | 4.857399 | | -4.29653 | | 0.000238 | | | 0.001468 | | | | -0.27723 |
| LOC728643 | | -1.01837 | | 8.812808 | | -4.28862 | | 0.000243 | | | 0.001495 | | | | -0.29728 |
| ZNF335 | | -1.40195 | | 3.306322 | | -4.2932 | | 0.000259 | | | 0.001584 | | | | -0.32604 |
| VWCE | | -1.48479 | | 4.894611 | | -4.27096 | | 0.000254 | | | 0.001558 | | | | -0.34206 |
| MX2 | | -3.67777 | | 1.999431 | | -5.55806 | | 0.001062 | | | 0.005292 | | | | -0.35715 |
| PAXIP1 | | -1.13294 | | 6.939864 | | -4.26417 | | 0.000259 | | | 0.001582 | | | | -0.35926 |
| SMC6L1 | | -1.19594 | | 4.638477 | | -4.26108 | | 0.000261 | | | 0.001593 | | | | -0.36708 |
| CYP3A43 | | 2.016391 | | 2.022635 | | 5.096926 | | 0.000756 | | | 0.003962 | | | | -0.36882 |
| CPNE2 | | -1.49555 | | 3.36647 | | -4.25774 | | 0.000263 | | | 0.001605 | | | | -0.37557 |
| FLJ14834 | | -4.16274 | | 1.014 | | -4.81695 | | 0.000801 | | | 0.004152 | | | | -0.40296 |
| PDE7B | | -2.51183 | | 4.174074 | | -4.29183 | | 0.000307 | | | 0.001834 | | | | -0.41331 |
| STK38L | | 2.046564 | | 3.383947 | | 4.247319 | | 0.000291 | | | 0.001749 | | | | -0.43719 |
| FN5 | | 1.045515 | | 6.00513 | | 4.227629 | | 0.000284 | | | 0.001715 | | | | -0.45181 |
| SLC7A10 | | -2.5911 | | 2.028184 | | -4.538 | | 0.000502 | | | 0.002798 | | | | -0.45552 |
| C13orf3 | | -2.20567 | | 3.527128 | | -4.2409 | | 0.000296 | | | 0.001774 | | | | -0.45639 |
| FLJ40235 | | 5.274571 | | -0.99027 | | 8.462385 | | 0.001752 | | | 0.008048 | | | | -0.45982 |
| ROBO2 | | 2.014037 | | 4.445947 | | 4.326901 | | 0.000638 | | | 0.003445 | | | | -0.46627 |
| S100A5 | | 1.189234 | | 3.910477 | | 4.245214 | | 0.000316 | | | 0.001881 | | | | -0.48506 |
| RRM2B | | 1.131914 | | 6.919293 | | 4.213711 | | 0.000295 | | | 0.001768 | | | | -0.48702 |
| MGC3101 | | 1.905062 | | 5.503466 | | 4.20745 | | 0.000299 | | | 0.001792 | | | | -0.50286 |
| KIAA1727 | | -1.16788 | | 4.128266 | | -4.20486 | | 0.000301 | | | 0.001802 | | | | -0.50941 |
| FLJ31568 | | -2.56532 | | 2.871754 | | -4.33453 | | 0.00042 | | | 0.002403 | | | | -0.51906 |
| CASP14 | | 10.2691 | | -1.3188 | | 18.20347 | | 0.000885 | | | 0.004525 | | | | -0.5804 |
| BUB1B | | -1.71342 | | 3.357228 | | -4.18316 | | 0.000342 | | | 0.002011 | | | | -0.60009 |
| MCM6 | | -1.23774 | | 9.205422 | | -4.16841 | | 0.000331 | | | 0.001957 | | | | -0.60151 |
| DPF1 | | -1.68727 | | 2.95968 | | -4.17083 | | 0.000353 | | | 0.002066 | | | | -0.63072 |
| KLHL3 | | 2.459529 | | 1.254737 | | 4.571614 | | 0.000705 | | | 0.003744 | | | | -0.6309 |
| PPIF | | -1.41378 | | 3.359729 | | -4.14568 | | 0.000351 | | | 0.002057 | | | | -0.65887 |
| DEF6 | | -1.84463 | | 3.457471 | | -4.1451 | | 0.000351 | | | 0.002059 | | | | -0.66035 |
| ARHGAP9 | | 2.761125 | | 3.33902 | | 4.231829 | | 0.000526 | | | 0.002917 | | | | -0.66889 |
| C14orf49 | | -1.74763 | | 1.824772 | | -4.20054 | | 0.000418 | | | 0.002392 | | | | -0.67114 |
| LOC441282 | | 2.842765 | | 2.340891 | | 4.25114 | | 0.000505 | | | 0.002808 | | | | -0.67178 |
| PELI2 | | -2.37574 | | 3.251143 | | -4.16313 | | 0.000388 | | | 0.002238 | | | | -0.68573 |
| WNT2 | | -2.43948 | | 6.244924 | | -4.13111 | | 0.000364 | | | 0.002121 | | | | -0.69564 |
| GPR179 | | -1.68797 | | 2.254183 | | -4.15873 | | 0.000392 | | | 0.002258 | | | | -0.69647 |
| SCG3 | | 1.266472 | | 3.447773 | | 4.128473 | | 0.000366 | | | 0.002132 | | | | -0.70228 |
| NPTX1 | | -3.93934 | | 2.380735 | | -5.22984 | | 0.001484 | | | 0.006981 | | | | -0.70291 |
| RPL23AP13 | | 1.020214 | | 6.402591 | | 4.120357 | | 0.000374 | | | 0.002171 | | | | -0.72274 |
| HIST1H2AE | | -2.54401 | | 1.8271 | | -4.37455 | | 0.000683 | | | 0.003642 | | | | -0.74019 |
| CACNA1A | | -4.99043 | | 1.712038 | | -4.62314 | | 0.001424 | | | 0.006746 | | | | -0.74583 |
| SLC35F1 | | -2.241 | | 2.425636 | | -4.18051 | | 0.00048 | | | 0.002697 | | | | -0.75574 |
| COL10A1 | | -1.80669 | | 7.008856 | | -4.1064 | | 0.000388 | | | 0.002238 | | | | -0.7579 |
| ANXA10 | | 2.136084 | | 2.230001 | | 4.168972 | | 0.000493 | | | 0.002758 | | | | -0.758 |
| CCDC54 | | 7.648099 | | -1.66257 | | 14.40771 | | 0.001604 | | | 0.007475 | | | | -0.76408 |
| WSB2 | | 1.149695 | | 7.412323 | | 4.102566 | | 0.000391 | | | 0.002257 | | | | -0.76755 |
| NDST3 | | -3.21027 | | 3.555446 | | -4.14647 | | 0.000475 | | | 0.002671 | | | | -0.79823 |
| MT1G | | 1.575987 | | 6.026823 | | 4.081334 | | 0.000413 | | | 0.002368 | | | | -0.82099 |
| DCLRE1B | | -1.53556 | | 3.064166 | | -4.09334 | | 0.000429 | | | 0.002447 | | | | -0.82301 |
| TSPAN12 | | 6.196861 | | 0.892436 | | 13.5535 | | 0.001873 | | | 0.008531 | | | | -0.82345 |
| TNFSF13B | | 1.288094 | | 3.70347 | | 4.090953 | | 0.000431 | | | 0.002459 | | | | -0.82569 |
| ProSAPiP1 | | 2.040246 | | 5.46165 | | 4.074354 | | 0.000421 | | | 0.002405 | | | | -0.83855 |
| PLAC1 | | 1.173082 | | 2.706719 | | 4.114796 | | 0.000472 | | | 0.002655 | | | | -0.84187 |
| NUDT12 | | 1.039449 | | 3.728603 | | 4.062726 | | 0.000433 | | | 0.00247 | | | | -0.86779 |
| U2AF1L4 | | -1.30124 | | 3.32725 | | -4.05338 | | 0.000444 | | | 0.002517 | | | | -0.89127 |
| FLJ40504 | | -1.02666 | | 8.57772 | | -4.04175 | | 0.000457 | | | 0.002584 | | | | -0.92048 |
| LOC401410 | | 6.623794 | | -0.34371 | | 7.107638 | | 0.003169 | | | 0.013334 | | | | -0.94745 |
| GNB3 | | -1.32995 | | 3.167644 | | -4.06311 | | 0.000535 | | | 0.002954 | | | | -0.96169 |
| NEUROD2 | | -3.51209 | | 0.776441 | | -5.34108 | | 0.002228 | | | 0.009883 | | | | -0.9621 |
| LMOD1 | | 1.58249 | | 4.908668 | | 4.017574 | | 0.000486 | | | 0.002723 | | | | -0.98116 |
| ARL4D | | -1.10523 | | 3.544721 | | -4.01624 | | 0.000488 | | | 0.002731 | | | | -0.9845 |
| LGALS3 | | 1.496691 | | 8.556879 | | 4.015773 | | 0.000489 | | | 0.002733 | | | | -0.98567 |
| TMEM88 | | -3.127 | | 3.07557 | | -4.05278 | | 0.000548 | | | 0.003019 | | | | -0.98637 |
| C22orf8 | | -1.91707 | | 3.395093 | | -4.0121 | | 0.000493 | | | 0.002758 | | | | -0.99488 |
| IFT88 | | -1.29077 | | 4.325339 | | -4.00847 | | 0.000498 | | | 0.002778 | | | | -1.00398 |
| PRAMEF6 | | -5.53472 | | -0.4057 | | -6.96352 | | 0.003396 | | | 0.014152 | | | | -1.0076 |
| RENBP | | 2.546815 | | 4.557718 | | 4.046531 | | 0.000602 | | | 0.003276 | | | | -1.01539 |
| PYCR1 | | 1.324162 | | 3.07 | | 4.071563 | | 0.000618 | | | 0.003351 | | | | -1.02025 |
| CD7 | | -2.74807 | | 3.177654 | | -4.1033 | | 0.000778 | | | 0.004057 | | | | -1.02048 |
| OR1N1 | | -1.99236 | | 0.931894 | | -4.1885 | | 0.000972 | | | 0.004907 | | | | -1.04758 |
| RNF150 | | -1.02423 | | 9.279569 | | -3.99063 | | 0.000521 | | | 0.00289 | | | | -1.04867 |
| SYNPO2L | | 1.929228 | | 3.324533 | | 4.041629 | | 0.000663 | | | 0.003556 | | | | -1.05047 |
| MGC33486 | | 2.453478 | | 3.649856 | | 4.060555 | | 0.000695 | | | 0.003698 | | | | -1.06166 |
| MLF1IP -2.16649 | | | | 3.195043 | | -4.00716 | | 0.000569 | | | 0.003116 | | | | -1.06559 |
| UCP3 -1.64488 | | | | 3.46475 | | -3.97848 | | 0.000537 | | | 0.002969 | | | | -1.0791 |
| TUBA2 2.560185 | | | | 1.804059 | | 4.109915 | | 0.000864 | | | 0.004435 | | | | -1.08209 |
| MGC23909 1.086577 | | | | 7.575276 | | 3.975512 | | 0.000541 | | | 0.002989 | | | | -1.08653 |
| GLYCTK -1.62845 | | | | 4.265433 | | -3.97416 | | 0.000543 | | | 0.002997 | | | | -1.08991 |
| OSAP -1.15882 | | | | 9.164838 | | -3.9736 | | 0.000544 | | | 0.003 | | | | -1.09133 |
| RBPMS 1.031595 | | | | 7.757205 | | 3.973109 | | 0.000545 | | | 0.003002 | | | | -1.09255 |
| CHIA 4.818272 | | | | 0.825419 | | 10.72042 | | 0.003388 | | | 0.014129 | | | | -1.10133 |
| KIAA1912 -2.69419 | | | | 4.131304 | | -3.96922 | | 0.00055 | | | 0.003029 | | | | -1.10227 |
| PBEF1 -1.73185 | | | | 6.125044 | | -3.96868 | | 0.000551 | | | 0.003031 | | | | -1.10364 |
| RASGRP1 1.245399 | | | | 3.888686 | | 3.967789 | | 0.000552 | | | 0.003035 | | | | -1.10586 |
| COL5A3 -1.21835 | | | | 4.520085 | | -3.97663 | | 0.000575 | | | 0.003141 | | | | -1.11147 |
| ABT1 -1.51735 | | | | 3.911998 | | -3.97623 | | 0.000575 | | | 0.003143 | | | | -1.11247 |
| FLJ32569 -4.69273 | | | | -0.34173 | | -5.61193 | | 0.003316 | | | 0.013867 | | | | -1.1256 |
| DKFZp686D097 4.867432 | | | | -0.74595 | | 10.49567 | | 0.003574 | | | 0.014791 | | | | -1.13057 |
| C14orf145 | | -6.21281 | | 2.17362 | | -4.15144 | | 0.001221 | | | 0.005943 | | | | -1.14506 |
| NFE2 | | -1.58613 | | 2.979586 | | -3.97747 | | 0.000658 | | | 0.003531 | | | | -1.16611 |
| CABP1 | | -5.04743 | | -1.09729 | | -10.193 | | 0.003847 | | | 0.015704 | | | | -1.17211 |
| ADORA2A | | -2.01127 | | 3.120949 | | -3.95055 | | 0.000613 | | | 0.00333 | | | | -1.17572 |
| PGA3 | | 4.510708 | | 0.151753 | | 10.15332 | | 0.003885 | | | 0.015829 | | | | -1.17775 |
| PROC | | -4.45809 | | 0.156266 | | -5.11361 | | 0.002739 | | | 0.011796 | | | | -1.17775 |
| MGC26963 | | 2.707417 | | 3.01669 | | 3.982812 | | 0.000759 | | | 0.003974 | | | | -1.18532 |
| APOC1 | | -1.46951 | | 3.442765 | | -3.94652 | | 0.00062 | | | 0.003358 | | | | -1.18564 |
| SLITRK6 | | -1.67503 | | 2.391001 | | -4.03735 | | 0.000807 | | | 0.004181 | | | | -1.18754 |
| KIAA0980 | | -1.22206 | | 4.22146 | | -3.93266 | | 0.000604 | | | 0.003285 | | | | -1.19366 |
| PIK3CG | | -2.12259 | | 2.845463 | | -3.9643 | | 0.000679 | | | 0.003624 | | | | -1.19749 |
| TJP2 | | -2.82379 | | 2.461407 | | -4.05112 | | 0.00087 | | | 0.004463 | | | | -1.20043 |
| KLHL13 | | 1.253934 | | 2.651484 | | 4.012118 | | 0.000775 | | | 0.004047 | | | | -1.20182 |
| MRGPRF | | -1.26161 | | 4.079214 | | -3.93611 | | 0.000636 | | | 0.003434 | | | | -1.21125 |
| SLCO2B1 | | 4.272317 | | 2.440475 | | 4.18591 | | 0.00166 | | | 0.007689 | | | | -1.21818 |
| DCHS1 | | -1.16043 | | 7.006415 | | -3.92119 | | 0.000622 | | | 0.003367 | | | | -1.22227 |
| HTR2B | | -1.12624 | | 7.245531 | | -3.92028 | | 0.000623 | | | 0.003372 | | | | -1.22455 |
| OR56A1 | | 3.120788 | | 0.649426 | | 6.470969 | | 0.004341 | | | 0.017376 | | | | -1.22728 |
| FLJ32771 | | 1.146552 | | 3.217657 | | 3.916582 | | 0.000629 | | | 0.0034 | | | | -1.23377 |
| HIST1H2BH | | -1.221 | | 3.272724 | | -3.91187 | | 0.000637 | | | 0.003437 | | | | -1.24552 |
| HDAC5 | | -1.28947 | | 3.650583 | | -3.90078 | | 0.000655 | | | 0.003519 | | | | -1.27318 |
| GABARAPL2 | | 1.643035 | | 6.934262 | | 3.896038 | | 0.000663 | | | 0.003556 | | | | -1.28499 |
| UNQ5783 | | -4.22098 | | -0.46674 | | -9.44238 | | 0.004661 | | | 0.018512 | | | | -1.28708 |
| INA | | 1.090212 | | 7.345006 | | 3.886237 | | 0.00068 | | | 0.003627 | | | | -1.3094 |
| NUP210L | | 2.558695 | | 0.503385 | | 5.005616 | | 0.003028 | | | 0.01285 | | | | -1.31559 |
| DPT | | 1.603585 | | 2.449322 | | 3.905315 | | 0.000783 | | | 0.004069 | | | | -1.33785 |
| PLEKHA6 | | -1.00393 | | 4.046337 | | -3.86761 | | 0.000713 | | | 0.003773 | | | | -1.35574 |
| TRPC6 | | -1.40078 | | 2.771691 | | -3.87704 | | 0.000737 | | | 0.003874 | | | | -1.35636 |
| EDIL3 | | 2.538622 | | 4.350639 | | 3.864653 | | 0.000718 | | | 0.003798 | | | | -1.36311 |
| CDC6 | | -1.87626 | | 2.260803 | | -4.02414 | | 0.001166 | | | 0.005714 | | | | -1.36908 |
| HIF1A | | 1.139864 | | 8.244856 | | 3.852227 | | 0.000741 | | | 0.003892 | | | | -1.39399 |
| NOXA1 | | -1.31362 | | 2.538416 | | -3.89926 | | 0.000921 | | | 0.004687 | | | | -1.4153 |
| SLC12A2 | | 1.182293 | | 6.566934 | | 3.839479 | | 0.000765 | | | 0.004002 | | | | -1.42566 |
| TMCO5 | | 1.703671 | | 4.164449 | | 3.878539 | | 0.000896 | | | 0.004573 | | | | -1.43398 |
| METTL7B | | 2.781877 | | 2.581796 | | 3.874772 | | 0.001269 | | | 0.006132 | | | | -1.44394 |
| UBXD6 | | 1.432223 | | 4.409129 | | 3.831545 | | 0.000781 | | | 0.004063 | | | | -1.44535 |
| KIAA1553 | | -2.47292 | | 2.693007 | | -3.83057 | | 0.000783 | | | 0.004069 | | | | -1.44778 |
| PDE3A | | -2.50117 | | 1.536038 | | -3.96547 | | 0.001165 | | | 0.005714 | | | | -1.44889 |
| LBR | | -1.95713 | | 4.852042 | | -3.82219 | | 0.0008 | | | 0.00415 | | | | -1.46857 |
| SLC1A1 | | 1.510173 | | 4.759929 | | 3.818528 | | 0.000807 | | | 0.004181 | | | | -1.47764 |
| CDK3 | | -2.18888 | | 2.761819 | | -3.8304 | | 0.00088 | | | 0.004505 | | | | -1.49315 |
| PDK3 | | 1.099547 | | 2.706919 | | 3.806869 | | 0.000831 | | | 0.004288 | | | | -1.50653 |
| GSTO2 | | -1.12491 | | 3.066062 | | -3.83991 | | 0.000982 | | | 0.00495 | | | | -1.51545 |
| CNIH2 | | -2.85759 | | 2.111562 | | -3.82626 | | 0.000947 | | | 0.004794 | | | | -1.52541 |
| BDP1 | | 1.162715 | | 3.353322 | | 3.793687 | | 0.000859 | | | 0.004414 | | | | -1.53917 |
| ELAC1 | | -1.01082 | | 3.873275 | | -3.79148 | | 0.000864 | | | 0.004436 | | | | -1.54464 |
| OR8D4 | | 5.220343 | | -0.54438 | | 8.090695 | | 0.006856 | | | 0.025634 | | | | -1.54732 |
| BIRC5 | | -3.32672 | | 3.485713 | | -3.85922 | | 0.002095 | | | 0.009382 | | | | -1.56212 |
| APOL3 | | -1.47648 | | 4.430295 | | -3.79251 | | 0.00091 | | | 0.004637 | | | | -1.56314 |
| CYP27B1 | | -2.28684 | | 2.744042 | | -3.80874 | | 0.000988 | | | 0.004975 | | | | -1.56689 |
| ARHGAP28 | | 3.379862 | | 0.845689 | | 3.974651 | | 0.002357 | | | 0.010366 | | | | -1.57183 |
| CDH24 | | -1.47271 | | 3.510309 | | -3.77521 | | 0.000901 | | | 0.004594 | | | | -1.58487 |
| NEK8 | | -1.7452 | | 3.721142 | | -3.7816 | | 0.000936 | | | 0.004745 | | | | -1.58974 |
| HORMAD2 | | 3.68396 | | 1.12735 | | 4.989381 | | 0.005319 | | | 0.020652 | | | | -1.5933 |
| RAB11B | | -1.06128 | | 4.057642 | | -3.77069 | | 0.000911 | | | 0.004637 | | | | -1.59606 |
| KRT19 | | -2.99428 | | 3.020095 | | -3.82103 | | 0.001194 | | | 0.005826 | | | | -1.59817 |
| IQGAP2 | | 1.655649 | | 3.187674 | | 3.806509 | | 0.001342 | | | 0.006413 | | | | -1.59838 |
| C18orf14 | | 3.526496 | | -2.73092 | | 7.840465 | | 0.007413 | | | 0.027401 | | | | -1.60472 |
| DRP2 | | 5.266289 | | 2.63494 | | 4.569398 | | 0.00461 | | | 0.018335 | | | | -1.60671 |
| ICAM5 | | 2.771177 | | 3.983029 | | 3.779441 | | 0.000997 | | | 0.005015 | | | | -1.60854 |
| KIAA0286 | | -1.21683 | | 4.334847 | | -3.76512 | | 0.000924 | | | 0.004697 | | | | -1.60981 |
| C17orf56 | | -1.77736 | | 3.005712 | | -3.78597 | | 0.001115 | | | 0.005508 | | | | -1.64086 |
| ZNF569 | | 5.979583 | | -0.67295 | | 7.599752 | | 0.008009 | | | 0.029246 | | | | -1.66314 |
| NRGN | | -1.29969 | | 3.69376 | | -3.78368 | | 0.001204 | | | 0.005865 | | | | -1.66495 |
| CHAC1 | | 1.351607 | | 5.11196 | | 3.740932 | | 0.000982 | | | 0.004951 | | | | -1.66952 |
| KIAA1539 | | 1.048958 | | 8.370318 | | 3.734794 | | 0.000997 | | | 0.005018 | | | | -1.68465 |
| RAD54L | | -1.23602 | | 3.941772 | | -3.75042 | | 0.00107 | | | 0.005324 | | | | -1.68533 |
| SPANX-N3 | | 3.183137 | | 0.756247 | | 4.865816 | | 0.005872 | | | 0.022413 | | | | -1.69292 |
| GALNTL4 | | 2.598133 | | 4.222787 | | 3.761665 | | 0.001366 | | | 0.006514 | | | | -1.69668 |
| AIM1L | | -2.69696 | | 0.618334 | | -4.85777 | | 0.005911 | | | 0.022544 | | | | -1.69949 |
| SOX9 | | -1.01489 | | 6.927194 | | -3.72612 | | 0.001019 | | | 0.005111 | | | | -1.70602 |
| RSN | | -2.01751 | | 2.55715 | | -3.76495 | | 0.001257 | | | 0.006087 | | | | -1.7076 |
| FOXR2 | | -6.09292 | | -0.91672 | | -5.53351 | | 0.007272 | | | 0.026949 | | | | -1.71387 |
| DUSP23 | | -1.33336 | | 2.535836 | | -3.72253 | | 0.001029 | | | 0.00515 | | | | -1.71486 |
| C6orf105 | | 1.498538 | | 3.090518 | | 3.752257 | | 0.001512 | | | 0.007096 | | | | -1.71655 |
| VIT | | -1.20581 | | 4.57092 | | -3.71691 | | 0.001043 | | | 0.00521 | | | | -1.7287 |
| THBS1 | | 1.084829 | | 12.22487 | | 3.710558 | | 0.00106 | | | 0.005285 | | | | -1.74434 |
| CYP2R1 | | 1.497902 | | 2.924802 | | 3.732289 | | 0.001266 | | | 0.00612 | | | | -1.74778 |
| TF | | -1.93045 | | 1.485781 | | -3.97159 | | 0.002369 | | | 0.010406 | | | | -1.75445 |
| KLF15 | | -1.25884 | | 4.04666 | | -3.7036 | | 0.001079 | | | 0.005359 | | | | -1.76146 |
| TDRD10 | | 4.918908 | | -0.93185 | | 7.22008 | | 0.009091 | | | 0.032483 | | | | -1.76221 |
| FLJ40172 | | -3.40254 | | 1.424374 | | -7.18189 | | 0.009211 | | | 0.032834 | | | | -1.77267 |
| TBXAS1 | | -1.70699 | | 1.498632 | | -4.3467 | | 0.003921 | | | 0.015937 | | | | -1.77863 |
| STS | | 1.752555 | | 3.00032 | | 3.701676 | | 0.001141 | | | 0.005622 | | | | -1.78085 |
| ZNF507 | | -1.94727 | | 2.475761 | | -3.70796 | | 0.001187 | | | 0.005801 | | | | -1.78698 |
| STAP2 | | 1.110558 | | 3.651828 | | 3.692807 | | 0.001109 | | | 0.005481 | | | | -1.78799 |
| EPR1 | | -2.59577 | | 2.789176 | | -3.76202 | | 0.00148 | | | 0.00697 | | | | -1.78988 |
| MAL | | 4.69443 | | -0.37533 | | 5.394881 | | 0.007897 | | | 0.028886 | | | | -1.79447 |
| SORCS2 | | 1.204921 | | 6.276719 | | 3.687171 | | 0.001125 | | | 0.005553 | | | | -1.80184 |
| CUBN | | -3.14391 | | 1.544175 | | -4.73149 | | 0.006553 | | | 0.02464 | | | | -1.8039 |
| KLRG1 | | 4.278584 | | 0.085632 | | 4.366763 | | 0.005655 | | | 0.021752 | | | | -1.8137 |
| SOX11 | | -4.406 | | 3.209994 | | -3.71282 | | 0.00218 | | | 0.009693 | | | | -1.81715 |
| ACADS | | -1.89725 | | 2.827558 | | -3.68739 | | 0.001183 | | | 0.005782 | | | | -1.81878 |
| RGMA | | -1.40188 | | 7.266508 | | -3.6799 | | 0.001145 | | | 0.005636 | | | | -1.81969 |
| GUCA1B | | 1.01206 | | 3.568411 | | 3.675295 | | 0.001159 | | | 0.005695 | | | | -1.831 |
| SN | | -1.80748 | | 0.575459 | | -3.8668 | | 0.002395 | | | 0.010503 | | | | -1.83884 |
| C17orf69 | | 1.375988 | | 3.354951 | | 3.673299 | | 0.001225 | | | 0.005952 | | | | -1.8496 |
| CD96 | | -2.71178 | | 2.687814 | | -3.75709 | | 0.001797 | | | 0.008228 | | | | -1.8521 |
| ABCC6 | | -1.07907 | | 4.219949 | | -3.66371 | | 0.001193 | | | 0.005823 | | | | -1.85941 |
| GDF10 | | -4.41763 | | 0.531934 | | -5.27795 | | 0.008477 | | | 0.030683 | | | | -1.86437 |
| DOK5 | | 2.713645 | | -0.35153 | | 5.26226 | | 0.008559 | | | 0.030947 | | | | -1.87389 |
| TDP1 | | 1.342364 | | 8.947652 | | 3.656498 | | 0.001215 | | | 0.005919 | | | | -1.87709 |
| FOLR3 | | 1.738007 | | 2.614137 | | 3.681069 | | 0.002325 | | | 0.010255 | | | | -1.88069 |
| MMAA | | 2.18153 | | 3.285161 | | 3.663013 | | 0.001325 | | | 0.006351 | | | | -1.8871 |
| KIF1A | | 2.85373 | | 4.472437 | | 3.522247 | | 0.003534 | | | 0.014654 | | | | -1.89121 |
| TH | | -3.6099 | | 2.196043 | | -3.69899 | | 0.001573 | | | 0.00736 | | | | -1.89171 |
| RAG2 | | -3.46357 | | 0.17399 | | -4.03161 | | 0.003285 | | | 0.013759 | | | | -1.89635 |
| HSPG2 | | 1.166684 | | 3.635822 | | 3.646845 | | 0.001245 | | | 0.006039 | | | | -1.90073 |
| FGD6 | | -1.31326 | | 4.119952 | | -3.65273 | | 0.001289 | | | 0.00622 | | | | -1.90263 |
| LHX4 | | -1.00283 | | 3.798803 | | -3.65189 | | 0.001291 | | | 0.006231 | | | | -1.90468 |
| MGC34647 | | 2.629644 | | 3.365703 | | 3.636719 | | 0.002309 | | | 0.010204 | | | | -1.91001 |
| CEP152 | | -1.99777 | | 3.507032 | | -3.65416 | | 0.001354 | | | 0.006463 | | | | -1.91539 |
| LHX3 | | -3.15361 | | -1.85297 | | -6.67772 | | 0.011018 | | | 0.038296 | | | | -1.92013 |
| BMP2K | | -1.01029 | | 6.035855 | | -3.63889 | | 0.00127 | | | 0.006135 | | | | -1.92021 |
| C10orf53 | | 3.114331 | | 1.163779 | | 6.667566 | | 0.011059 | | | 0.038408 | | | | -1.92329 |
| SLC25A18 | | -3.01842 | | 1.479982 | | -6.66413 | | 0.011073 | | | 0.038425 | | | | -1.92436 |
| LOC728215 | | -4.36924 | | -0.05569 | | -4.67846 | | 0.006848 | | | 0.025618 | | | | -1.93845 |
| RNF152 | | -1.03053 | | 5.231857 | | -3.63064 | | 0.001297 | | | 0.006248 | | | | -1.94037 |
| TNFAIP8 | | -1.09282 | | 6.498472 | | -3.62741 | | 0.001307 | | | 0.006285 | | | | -1.94828 |
| ARL13B | | 1.285105 | | 3.959985 | | 3.626485 | | 0.00131 | | | 0.006296 | | | | -1.95054 |
| HEPACAM | | -1.80697 | | 1.046167 | | -4.34958 | | 0.005755 | | | 0.022064 | | | | -1.9602 |
| SLC9A8 | | -1.02891 | | 4.857284 | | -3.62131 | | 0.001327 | | | 0.006362 | | | | -1.96318 |
| C11orf47 | | 1.208603 | | 3.760554 | | 3.619951 | | 0.001332 | | | 0.006376 | | | | -1.96651 |
| PDGFD | | -1.05795 | | 9.308777 | | -3.6199 | | 0.001332 | | | 0.006376 | | | | -1.96663 |
| CHRNA4 | | -1.23919 | | 2.166561 | | -3.66858 | | 0.001685 | | | 0.007777 | | | | -1.96975 |
| RCC1 | | -1.52661 | | 2.316648 | | -3.6488 | | 0.001643 | | | 0.007624 | | | | -1.97131 |
| ITGB7 | | -3.12937 | | 0.96618 | | -3.99298 | | 0.004472 | | | 0.01785 | | | | -1.97626 |
| KIAA0802 | | -1.2649 | | 4.937371 | | -3.61463 | | 0.00135 | | | 0.006446 | | | | -1.97951 |
| SLC16A1 | | 1.853345 | | 1.313944 | | 3.64579 | | 0.001655 | | | 0.007671 | | | | -1.99245 |
| AGPAT3 | | -1.43845 | | 3.624927 | | -3.60628 | | 0.001378 | | | 0.006559 | | | | -1.9999 |
| LOC338799 | | 1.0127 | | 4.287313 | | 3.605289 | | 0.001382 | | | 0.006572 | | | | -2.00231 |
| PSIP1 | | -1.14681 | | 7.184769 | | -3.60438 | | 0.001385 | | | 0.00658 | | | | -2.00453 |
| EFEMP1 | | 1.547171 | | 9.047473 | | 3.603281 | | 0.001389 | | | 0.006596 | | | | -2.00721 |
| LBX2 | | -3.11945 | | 2.575936 | | -3.68211 | | 0.002101 | | | 0.009408 | | | | -2.00745 |
| FLT3LG | | -2.04412 | | 3.862218 | | -3.63461 | | 0.001698 | | | 0.007831 | | | | -2.01775 |
| JOSD2 1.080825 | | | | 7.20686 | | 3.598752 | | 0.001405 | | | 0.006668 | | | | -2.01826 |
| DEPDC2 -3.77992 | | | | -0.17114 | | -5.08758 | | 0.009541 | | | 0.033844 | | | | -2.02217 |
| OR2D3 -3.09136 | | | | -0.17746 | | -4.32666 | | 0.005892 | | | 0.022484 | | | | -2.02391 |
| DLG1 1.212412 | | | | 3.82686 | | 3.605494 | | 0.001609 | | | 0.007493 | | | | -2.03319 |
| FZD1 -1.25018 | | | | 3.541462 | | -3.58907 | | 0.001508 | | | 0.007082 | | | | -2.05611 |
| C20orf106 -3.43282 | | | | 2.094389 | | -3.62226 | | 0.002907 | | | 0.012409 | | | | -2.06198 |
| DKK4 -2.26294 | | | | 3.505685 | | -3.6198 | | 0.002921 | | | 0.012463 | | | | -2.06675 |
| C10orf119 -1.20383 | | | | 4.069231 | | -3.57589 | | 0.001487 | | | 0.006995 | | | | -2.07396 |
| CMKOR1 2.881562 | | | | 3.693367 | | 3.608914 | | 0.002983 | | | 0.012688 | | | | -2.08788 |
| ZNF141 2.005359 | | | | 1.025533 | | 4.973894 | | 0.010257 | | | 0.036064 | | | | -2.09855 |
| FBXL14 1.222286 | | | | 3.165505 | | 3.564991 | | 0.001529 | | | 0.007169 | | | | -2.10047 |
| DKFZp686I1521 1.58631 | | | | 2.903962 | | 3.564548 | | 0.00153 | | | 0.007173 | | | | -2.10155 |
| FLJ32745 | | -1.30763 | | 3.86197 | | -3.56414 | | 0.001532 | | | 0.007177 | | | | -2.10255 |
| LRRC16 | | 1.640813 | | 4.244689 | | 3.568646 | | 0.001667 | | | 0.007716 | | | | -2.11129 |
| PNPLA2 | | 1.049764 | | 6.162334 | | 3.558223 | | 0.001555 | | | 0.007282 | | | | -2.11692 |
| WFIKKN2 | | -2.30425 | | 0.488791 | | -4.85872 | | 0.011051 | | | 0.038388 | | | | -2.13011 |
| ASB17 | | -3.78086 | | -0.12818 | | -6.05137 | | 0.014015 | | | 0.046662 | | | | -2.13034 |
| CCDC64 | | -2.8932 | | 1.222632 | | -4.45884 | | 0.008245 | | | 0.029995 | | | | -2.13703 |
| BCAS3 | | 1.128785 | | 3.942167 | | 3.546624 | | 0.001601 | | | 0.007462 | | | | -2.1451 |
| RP1-32F7.2 | | 1.582726 | | 3.156515 | | 3.559482 | | 0.002018 | | | 0.009098 | | | | -2.14815 |
| LOC153364 | | 1.299829 | | 4.33709 | | 3.543229 | | 0.001614 | | | 0.007513 | | | | -2.15334 |
| TAGAP | | 3.526217 | | -0.84882 | | 5.943257 | | 0.014643 | | | 0.048442 | | | | -2.17004 |
| SPRN | | 1.665861 | | 3.132788 | | 3.539376 | | 0.001705 | | | 0.007859 | | | | -2.17209 |
| CASP12 | | -3.27871 | | 1.35251 | | -3.70979 | | 0.006582 | | | 0.024733 | | | | -2.18898 |
| LGI3 | | 1.928839 | | 0.508946 | | 4.173787 | | 0.006907 | | | 0.025786 | | | | -2.19352 |
| FAM7A2 | | -3.34636 | | 2.309128 | | -3.56009 | | 0.002152 | | | 0.009597 | | | | -2.19987 |
| NOTCH4 | | 2.648466 | | 0.329864 | | 4.388433 | | 0.008763 | | | 0.031559 | | | | -2.20231 |
| OR5A1 | | -3.70522 | | -1.86074 | | -5.85262 | | 0.015199 | | | 0.04993 | | | | -2.20416 |
| SLC6A18 | | 1.03403 | | 3.564318 | | 3.521257 | | 0.001705 | | | 0.007859 | | | | -2.20661 |
| PTGIS | | -3.39896 | | 2.735904 | | -3.6589 | | 0.004736 | | | 0.018775 | | | | -2.20978 |
| YPEL1 | | -1.25513 | | 3.607773 | | -3.52181 | | 0.001781 | | | 0.008158 | | | | -2.21742 |
| PSKH2 | | 1.524523 | | 1.107235 | | 4.796941 | | 0.011509 | | | 0.039651 | | | | -2.22102 |
| GPC3 | | 1.794166 | | 1.776976 | | 4.368253 | | 0.008918 | | | 0.031985 | | | | -2.22117 |
| PRR8 | | -1.20368 | | 3.457374 | | -3.50532 | | 0.001774 | | | 0.008134 | | | | -2.24519 |
| HAVCR1 | | 12.28321 | | -4.49038 | | 18.83823 | | 0.007882 | | | 0.028838 | | | | -2.26021 |
| MEI1 | | 1.357148 | | 2.72425 | | 3.487461 | | 0.001855 | | | 0.008468 | | | | -2.28834 |
| CART1 | | -1.72877 | | 4.396888 | | -3.49188 | | 0.001917 | | | 0.008698 | | | | -2.28892 |
| ATPBD1C | | 1.885974 | | 0.483068 | | 4.04524 | | 0.007916 | | | 0.028937 | | | | -2.29797 |
| C21orf100 | | -2.61761 | | 1.272418 | | -4.60019 | | 0.013134 | | | 0.044189 | | | | -2.30654 |
| TBC1D8B | | 2.702176 | | 2.444889 | | 3.815588 | | 0.005689 | | | 0.021864 | | | | -2.31146 |
| KIAA1279 | | 1.600877 | | 4.690899 | | 3.476265 | | 0.001908 | | | 0.008673 | | | | -2.31536 |
| CCL1 | | 2.198279 | | 2.559979 | | 3.521259 | | 0.003945 | | | 0.016015 | | | | -2.32071 |
| ZNF300 | | -2.65785 | | 2.127097 | | -3.4912 | | 0.002361 | | | 0.010382 | | | | -2.32661 |
| ZNF132 | | 1.024149 | | 3.715681 | | 3.467884 | | 0.001948 | | | 0.008819 | | | | -2.33556 |
| TMSB4X | | 2.383422 | | 3.147381 | | 3.473905 | | 0.002202 | | | 0.009775 | | | | -2.33938 |
| ALS2CR12 | | 1.258668 | | 2.487034 | | 3.486738 | | 0.002538 | | | 0.011045 | | | | -2.34009 |
| MGAT5B | | -1.15704 | | 3.457027 | | -3.46611 | | 0.002042 | | | 0.009179 | | | | -2.35031 |
| GRHL2 | | -8.73515 | | -3.29867 | | -13.3967 | | 0.013396 | | | 0.044948 | | | | -2.36542 |
| MASS1 | | 1.910043 | | 1.623553 | | 3.456378 | | 0.002188 | | | 0.009721 | | | | -2.37587 |
| AMHR2 | | 2.548114 | | 0.957316 | | 3.999554 | | 0.008314 | | | 0.030199 | | | | -2.39146 |
| PARVB | | 1.070268 | | 7.390005 | | 3.443488 | | 0.00207 | | | 0.009287 | | | | -2.39428 |
| HNRPA1L-2 | | -1.02281 | | 8.253728 | | -3.44247 | | 0.002075 | | | 0.009308 | | | | -2.39672 |
| C10orf114 | | 1.303319 | | 3.400309 | | 3.448097 | | 0.002232 | | | 0.009897 | | | | -2.40252 |
| FAM62C | | 3.591932 | | 1.719102 | | 4.056243 | | 0.011788 | | | 0.040465 | | | | -2.40654 |
| OR10X1 | | 4.083838 | | 1.136557 | | 3.812294 | | 0.010198 | | | 0.035887 | | | | -2.41591 |
| LRP10 | | 1.024278 | | 9.474342 | | 3.425266 | | 0.002165 | | | 0.009647 | | | | -2.43804 |
| LGR5 | | -5.7462 | | -0.29611 | | -4.41543 | | 0.014928 | | | 0.049254 | | | | -2.4389 |
| PAK6 | | -2.08948 | | 1.382856 | | -3.55514 | | 0.004184 | | | 0.016845 | | | | -2.4413 |
| ADAMTS19 | | 1.394316 | | 3.170296 | | 3.429666 | | 0.002333 | | | 0.010283 | | | | -2.44569 |
| FUT5 | | -1.70394 | | 1.854564 | | -3.61237 | | 0.005106 | | | 0.019964 | | | | -2.45339 |
| C1QL1 | | -1.76495 | | 2.660511 | | -3.4317 | | 0.002562 | | | 0.011134 | | | | -2.45544 |
| MMP24 | | 1.109658 | | 3.9033 | | 3.412401 | | 0.002329 | | | 0.010268 | | | | -2.47447 |
| TMEM100 | | 1.578144 | | 2.921278 | | 3.418833 | | 0.00251 | | | 0.010953 | | | | -2.47856 |
| AMOT | | -1.25984 | | 5.158326 | | -3.40441 | | 0.00228 | | | 0.010093 | | | | -2.48803 |
| HCRTR1 | | -2.79702 | | 0.309529 | | -3.64292 | | 0.005846 | | | 0.022331 | | | | -2.49217 |
| FAM80B | | -1.01463 | | 3.791158 | | -3.4004 | | 0.002303 | | | 0.010181 | | | | -2.49764 |
| C1orf112 | | -1.13155 | | 5.208661 | | -3.39856 | | 0.002314 | | | 0.010217 | | | | -2.50205 |
| ACADSB | | -1.65199 | | 2.96306 | | -3.41307 | | 0.002676 | | | 0.011547 | | | | -2.50638 |
| RPS6KA5 | | -1.23821 | | 3.138403 | | -3.4052 | | 0.002593 | | | 0.011242 | | | | -2.50996 |
| TEK | | 2.222741 | | 3.416546 | | 3.396938 | | 0.002644 | | | 0.011439 | | | | -2.51701 |
| DTX3L | | -2.4821 | | 3.371478 | | -3.39486 | | 0.002537 | | | 0.011045 | | | | -2.52702 |
| SSBP3 | | -1.60854 | | 4.388203 | | -3.38611 | | 0.002386 | | | 0.01047 | | | | -2.53181 |
| USP19 | | -1.69302 | | 2.049734 | | -3.40748 | | 0.003032 | | | 0.012864 | | | | -2.53592 |
| ITGB8 | | -1.36386 | | 3.08933 | | -3.38063 | | 0.002419 | | | 0.010596 | | | | -2.54491 |
| CD8B | | -2.15789 | | 1.381355 | | -4.01734 | | 0.012217 | | | 0.041647 | | | | -2.55985 |
| LYL1 | | -1.8449 | | 2.66456 | | -3.39511 | | 0.003317 | | | 0.013867 | | | | -2.56724 |
| BCAS2 | | 1.122025 | | 3.814486 | | 3.374979 | | 0.002662 | | | 0.01151 | | | | -2.57335 |
| MGC13057 | | -1.22467 | | 4.47587 | | -3.36889 | | 0.00259 | | | 0.011232 | | | | -2.58057 |
| KIF15 | | -3.28092 | | 5.960312 | | -3.17648 | | 0.007572 | | | 0.027905 | | | | -2.59252 |
| HRSP12 | | 1.555107 | | 4.990432 | | 3.357003 | | 0.002666 | | | 0.011522 | | | | -2.60525 |
| ARVCF | | 1.323411 | | 3.258182 | | 3.356656 | | 0.002782 | | | 0.011947 | | | | -2.60872 |
| ATP10D | | 1.026741 | | 3.917627 | | 3.353387 | | 0.002587 | | | 0.011223 | | | | -2.60987 |
| DEPDC1B | | -4.79299 | | 2.029322 | | -3.38379 | | 0.004242 | | | 0.017039 | | | | -2.62163 |
| TFEB | | 1.329725 | | 1.948601 | | 3.351972 | | 0.002941 | | | 0.01254 | | | | -2.63223 |
| SOS2 | | -1.50553 | | 3.318554 | | -3.34613 | | 0.002737 | | | 0.011792 | | | | -2.63414 |
| C16orf54 | | 1.328244 | | 3.286288 | | 3.343331 | | 0.002756 | | | 0.011849 | | | | -2.64073 |
| PTGES | | -1.00719 | | 4.070771 | | -3.33988 | | 0.002779 | | | 0.01194 | | | | -2.64883 |
| LIN9 | | -1.62856 | | 3.347131 | | -3.3599 | | 0.004858 | | | 0.01915 | | | | -2.65599 |
| NFIB | | 1.396158 | | 1.819765 | | 3.341166 | | 0.003017 | | | 0.012814 | | | | -2.65698 |
| LOC652924 | | -1.09595 | | 3.494727 | | -3.33217 | | 0.002726 | | | 0.011749 | | | | -2.66035 |
| TM4SF11 | | -1.73654 | | 3.755373 | | -3.34116 | | 0.003329 | | | 0.013901 | | | | -2.66151 |
| PPP2CB | | 1.245873 | | 7.199964 | | 3.328684 | | 0.002749 | | | 0.01183 | | | | -2.66862 |
| LOC150084 | | -2.01545 | | 2.934667 | | -3.34259 | | 0.003972 | | | 0.01611 | | | | -2.67729 |
| GREM1 | | 1.376144 | | 8.7283 | | 3.320707 | | 0.002804 | | | 0.012025 | | | | -2.68756 |
| LOH3CR2A | | -3.12993 | | 3.43946 | | -3.39003 | | 0.00564 | | | 0.021719 | | | | -2.70144 |
| C9orf48 | | -1.65456 | | 2.521599 | | -3.31145 | | 0.003236 | | | 0.013584 | | | | -2.7249 |
| FCN3 | | -2.80038 | | 0.955631 | | -3.55502 | | 0.010329 | | | 0.03626 | | | | -2.72783 |
| SALL4 | | -1.1887 | | 3.113035 | | -3.30763 | | 0.003128 | | | 0.013177 | | | | -2.72963 |
| HOMER1 | | 2.326553 | | 2.501554 | | 3.301156 | | 0.003471 | | | 0.014412 | | | | -2.73266 |
| TRIM16 | | -1.07761 | | 4.243158 | | -3.30054 | | 0.002947 | | | 0.012562 | | | | -2.73536 |
| SH3BP2 | | -3.48609 | | 2.319307 | | -3.29979 | | 0.004087 | | | 0.016522 | | | | -2.74004 |
| LOC260341 | | 3.272415 | | -0.32571 | | 3.545718 | | 0.010454 | | | 0.036651 | | | | -2.74028 |
| VEZT | | 4.179455 | | 1.35676 | | 3.385328 | | 0.01287 | | | 0.043463 | | | | -2.77123 |
| SPRR2A | | -2.10557 | | 0.668374 | | -3.6385 | | 0.012387 | | | 0.042145 | | | | -2.77311 |
| DYNLT3 | | 3.417943 | | 1.858741 | | 3.378361 | | 0.005761 | | | 0.02208 | | | | -2.77536 |
| SBDS | | 1.014735 | | 8.825121 | | 3.277404 | | 0.003119 | | | 0.01315 | | | | -2.79004 |
| PGA5 | | -1.93693 | | 2.445766 | | -3.32971 | | 0.007083 | | | 0.026352 | | | | -2.7974 |
| AP3B2 | | -1.74751 | | 1.504744 | | -3.65115 | | 0.012211 | | | 0.041645 | | | | -2.80204 |
| FBXO32 | | 1.194686 | | 9.843691 | | 3.272024 | | 0.00316 | | | 0.013299 | | | | -2.80274 |
| AGBL2 | | -1.37773 | | 0.41212 | | -3.49374 | | 0.008909 | | | 0.031965 | | | | -2.80417 |
| ZNF124 | | -3.3953 | | 1.709952 | | -3.19713 | | 0.011596 | | | 0.039918 | | | | -2.82631 |
| TMCC1 | | 1.944647 | | 3.080933 | | 3.257379 | | 0.003527 | | | 0.014633 | | | | -2.83828 |
| RSAD2 | | -2.553 | | 1.485379 | | -3.24123 | | 0.006697 | | | 0.025133 | | | | -2.84588 |
| IL1A | | -1.32388 | | 6.539554 | | -3.25368 | | 0.003306 | | | 0.013839 | | | | -2.84598 |
| SPESP1 | | -1.91266 | | 1.407277 | | -3.27007 | | 0.00534 | | | 0.020712 | | | | -2.84861 |
| FBXO5 | | -2.07341 | | 2.690866 | | -3.26052 | | 0.004734 | | | 0.018769 | | | | -2.8489 |
| PKP2 | | 1.419726 | | 7.282701 | | 3.251106 | | 0.003327 | | | 0.013896 | | | | -2.85203 |
| C5orf16 | | 1.302793 | | 4.132557 | | 3.248913 | | 0.003465 | | | 0.014392 | | | | -2.85822 |
| SCYE1 | | 3.08624 | | 1.028678 | | 3.296211 | | 0.005504 | | | 0.021275 | | | | -2.86244 |
| TES | | -1.73055 | | 4.673405 | | -3.24446 | | 0.003381 | | | 0.014107 | | | | -2.86766 |
| JRK | | -1.22379 | | 2.822497 | | -3.23557 | | 0.004452 | | | 0.017775 | | | | -2.88852 |
| PKM2 | | 1.006706 | | 11.13178 | | 3.234791 | | 0.003462 | | | 0.014382 | | | | -2.89039 |
| MUT | | 1.886028 | | 1.465619 | | 3.307507 | | 0.007362 | | | 0.027235 | | | | -2.90378 |
| B3GALT2 | | 1.273323 | | 6.311699 | | 3.228594 | | 0.003515 | | | 0.014588 | | | | -2.90494 |
| RCV1 | | -1.18472 | | 3.239901 | | -3.22987 | | 0.003766 | | | 0.015434 | | | | -2.90874 |
| RNF43 | | 2.192896 | | 2.431378 | | 3.231316 | | 0.004747 | | | 0.018799 | | | | -2.91716 |
| ZNF326 | | -1.00533 | | 4.654676 | | -3.2222 | | 0.00357 | | | 0.014782 | | | | -2.91994 |
| MANEAL | | -4.85265 | | -0.37895 | | -3.32919 | | 0.011268 | | | 0.038983 | | | | -2.92815 |
| TNFSF8 | | 1.882529 | | 1.674786 | | 3.218791 | | 0.005174 | | | 0.020182 | | | | -2.93582 |
| THBS2 | | 1.095226 | | 11.75589 | | 3.212829 | | 0.003653 | | | 0.015066 | | | | -2.9419 |
| CRYGN | | 1.351419 | | 3.304467 | | 3.215172 | | 0.0039 | | | 0.015882 | | | | -2.94241 |
| ADAMTS8 | | 1.195453 | | 2.404115 | | 3.210008 | | 0.004485 | | | 0.017887 | | | | -2.95099 |
| EPHB6 | | -4.08365 | | 1.828922 | | -3.10515 | | 0.008669 | | | 0.031284 | | | | -2.96482 |
| CCDC62 | | 1.167176 | | 3.36306 | | 3.200597 | | 0.003763 | | | 0.015431 | | | | -2.97053 |
| PDE4DIP | | -1.30928 | | 5.466828 | | -3.19908 | | 0.003777 | | | 0.015469 | | | | -2.97408 |
| HHLA1 | | 2.425086 | | 0.711481 | | 3.224128 | | 0.005458 | | | 0.021126 | | | | -2.9796 |
| SRrp35 | | 2.141544 | | 2.240244 | | 3.174122 | | 0.004865 | | | 0.01917 | | | | -3.00434 |
| SLC2A12 | | -1.00824 | | 4.787649 | | -3.18281 | | 0.00393 | | | 0.015969 | | | | -3.01207 |
| HYI | | -1.05499 | | 4.992277 | | -3.18125 | | 0.003945 | | | 0.016015 | | | | -3.01572 |
| LOC387911 | | 1.415318 | | 3.285965 | | 3.18985 | | 0.005861 | | | 0.02238 | | | | -3.01932 |
| LAMA3 | | -1.32645 | | 0.611061 | | -3.46923 | | 0.015038 | | | 0.049516 | | | | -3.02411 |
| KLF5 | | 1.404492 | | 3.571524 | | 3.202464 | | 0.006123 | | | 0.023241 | | | | -3.03543 |
| ITGB3 | | -1.55849 | | 3.156228 | | -3.16932 | | 0.004349 | | | 0.017396 | | | | -3.04711 |
| GALR2 | | 1.721274 | | 2.005571 | | 3.243334 | | 0.012755 | | | 0.043132 | | | | -3.05508 |
| SLC7A4 | | -1.45457 | | 3.37475 | | -3.1624 | | 0.004268 | | | 0.017132 | | | | -3.06175 |
| NOX1 | | -1.21174 | | 2.673313 | | -3.15166 | | 0.004239 | | | 0.017032 | | | | -3.0846 |
| FSCN3 | | 3.322204 | | 1.408254 | | 3.133957 | | 0.007566 | | | 0.02789 | | | | -3.09294 |
| PTPRN | | 2.057105 | | 4.197948 | | 3.132228 | | 0.005348 | | | 0.020736 | | | | -3.09572 |
| METTL7A | | -1.53345 | | 3.378541 | | -3.14139 | | 0.004346 | | | 0.017393 | | | | -3.10846 |
| LIPG | | 2.765611 | | 3.388713 | | 3.124789 | | 0.006709 | | | 0.025165 | | | | -3.11084 |
| OTX2 | | -1.85593 | | 0.906783 | | -3.15508 | | 0.006739 | | | 0.025249 | | | | -3.12949 |
| LOC283174 | | -2.70343 | | 1.704347 | | -3.15499 | | 0.00674 | | | 0.025249 | | | | -3.12967 |
| FAM47B | | 2.652369 | | 0.314375 | | 3.164808 | | 0.010636 | | | 0.037134 | | | | -3.13975 |
| CYP8B1 | | 1.743883 | | 1.42138 | | 3.147517 | | 0.007368 | | | 0.027251 | | | | -3.15044 |
| KRTAP6-3 | | -1.87512 | | 0.348899 | | -3.29617 | | 0.014471 | | | 0.047949 | | | | -3.15295 |
| C9orf126 | | 2.400686 | | 2.485578 | | 3.120835 | | 0.00526 | | | 0.020437 | | | | -3.16091 |
| C21orf63 | | -2.34535 | | 1.478299 | | -3.11306 | | 0.005354 | | | 0.020757 | | | | -3.16932 |
| LOC144404 | | -1.44343 | | 2.891184 | | -3.10979 | | 0.004692 | | | 0.018629 | | | | -3.18162 |
| WNT7B | | -1.40771 | | 3.472568 | | -3.10791 | | 0.004863 | | | 0.019167 | | | | -3.18342 |
| MGC34830 | | -1.0568 | | 4.107148 | | -3.10792 | | 0.004713 | | | 0.018696 | | | | -3.18594 |
| PDE1A | | -2.0916 | | 3.532661 | | -3.10431 | | 0.005463 | | | 0.021139 | | | | -3.18859 |
| C6orf168 | | -1.67096 | | 4.702251 | | -3.10532 | | 0.004743 | | | 0.018791 | | | | -3.19194 |
| BOLA1 | | 1.256888 | | 3.746187 | | 3.104271 | | 0.004755 | | | 0.018823 | | | | -3.19437 |
| ZWINT | | -3.1617 | | 1.686638 | | -3.04822 | | 0.009657 | | | 0.03419 | | | | -3.20617 |
| DAPK2 | | 2.427552 | | 3.299128 | | 3.047391 | | 0.007078 | | | 0.026343 | | | | -3.22531 |
| DLGAP3 | | 1.658364 | | 1.216799 | | 3.176425 | | 0.011978 | | | 0.041011 | | | | -3.22746 |
| FLJ26175 | | -1.19595 | | 3.55453 | | -3.08526 | | 0.004978 | | | 0.019545 | | | | -3.2382 |
| ZNF702 | | 1.029012 | | 5.426581 | | 3.08108 | | 0.005029 | | | 0.019699 | | | | -3.24782 |
| OR1A1 | | 1.531749 | | 0.753914 | | 3.194919 | | 0.013684 | | | 0.045762 | | | | -3.24807 |
| GIF | | -1.23597 | | 1.537418 | | -3.09754 | | 0.008126 | | | 0.029637 | | | | -3.25639 |
| CASD1 | | -1.624 | | 4.342261 | | -3.07538 | | 0.005255 | | | 0.020427 | | | | -3.2576 |
| ADRA1D | | -1.76855 | | 2.533338 | | -3.07775 | | 0.006318 | | | 0.023886 | | | | -3.26009 |
| SATL1 | | 1.207883 | | 3.642588 | | 3.069897 | | 0.005324 | | | 0.020663 | | | | -3.27008 |
| HMP19 | | 1.560295 | | 3.157165 | | 3.070261 | | 0.005905 | | | 0.022527 | | | | -3.27218 |
| ECM2 | | -1.52487 | | 4.711583 | | -3.06304 | | 0.005252 | | | 0.020423 | | | | -3.28929 |
| LOC253012 | | -2.25852 | | 0.870704 | | -3.1179 | | 0.011499 | | | 0.039639 | | | | -3.29546 |
| PPCDC | | 1.587302 | | 2.004935 | | 3.056794 | | 0.009501 | | | 0.033709 | | | | -3.32076 |
| ACAT2 | | -1.32435 | | 2.331119 | | -3.04709 | | 0.006002 | | | 0.022841 | | | | -3.32384 |
| CCL11 | | 2.159057 | | 3.461041 | | 3.038987 | | 0.006115 | | | 0.023216 | | | | -3.32571 |
| PSPH | | 1.192105 | | 5.163833 | | 3.045307 | | 0.005481 | | | 0.021195 | | | | -3.32993 |
| EHMT1 | | -1.88066 | | 2.732268 | | -3.03575 | | 0.006388 | | | 0.024134 | | | | -3.33897 |
| KLK1 | | 1.421967 | | 3.173026 | | 3.034787 | | 0.005971 | | | 0.022734 | | | | -3.34359 |
| NUDCD2 | | 1.152047 | | 5.848415 | | 3.033426 | | 0.005639 | | | 0.021719 | | | | -3.3571 |
| LYPD3 | | -1.06695 | | 3.371677 | | -3.02544 | | 0.005748 | | | 0.022057 | | | | -3.37534 |
| EHD2 | | 1.000011 | | 7.491255 | | 3.02503 | | 0.005754 | | | 0.022064 | | | | -3.37628 |
| CRIP1 | | -1.28813 | | 3.423489 | | -3.02416 | | 0.005766 | | | 0.022095 | | | | -3.37827 |
| TGFBR1 | | -1.92668 | | 1.777315 | | -3.01997 | | 0.008851 | | | 0.031788 | | | | -3.37922 |
| GOLGA | | -1.27199 | | 4.797606 | | -3.02329 | | 0.005778 | | | 0.022126 | | | | -3.38024 |
| VSIG1 | | 1.32851 | | 4.072815 | | 3.016673 | | 0.006041 | | | 0.02298 | | | | -3.39065 |
| PUNC | | -2.54787 | | 2.758902 | | -2.92968 | | 0.011287 | | | 0.039033 | | | | -3.39624 |
| HIST1H2AM | | -1.96853 | | 2.673386 | | -3.00987 | | 0.00654 | | | 0.024598 | | | | -3.40212 |
| DSP | | 2.898507 | | 2.484198 | | 2.962187 | | 0.008506 | | | 0.030771 | | | | -3.40234 |
| KRTHB1 | | -1.25626 | | 2.602532 | | -3.00532 | | 0.007111 | | | 0.02644 | | | | -3.41005 |
| CLEC14A | | 3.882645 | | 0.33808 | | 3.060685 | | 0.014364 | | | 0.047666 | | | | -3.41217 |
| PLA2R1 | | 1.118957 | | 4.766735 | | 3.001377 | | 0.006089 | | | 0.023144 | | | | -3.43017 |
| SOX14 | | 1.098104 | | 3.211596 | | 3.000459 | | 0.006103 | | | 0.023189 | | | | -3.43225 |
| FOXL2 | | 1.454931 | | 4.53121 | | 2.962128 | | 0.008148 | | | 0.029703 | | | | -3.43725 |
| CHAF1A | | -2.76706 | | 2.643867 | | -2.97872 | | 0.007857 | | | 0.028764 | | | | -3.43736 |
| CCR3 | | 1.975246 | | 0.631231 | | 3.0101 | | 0.012375 | | | 0.042113 | | | | -3.45476 |
| ZNF385 | | -1.43747 | | 3.048982 | | -2.988 | | 0.006661 | | | 0.025007 | | | | -3.45528 |
| DEFB106B -1.47014 | | | | 1.807595 | | -2.98471 | | 0.008964 | | | 0.032113 | | | | -3.46354 |
| C9orf98 1.00619 | | | | 2.844537 | | 2.971458 | | 0.00767 | | | 0.028208 | | | | -3.46794 |
| PLA2G7 -1.55781 | | | | 3.846886 | | -2.97198 | | 0.007384 | | | 0.027305 | | | | -3.47771 |
| ADAMTS1 1.589837 | | | | 2.850617 | | 2.979582 | | 0.006415 | | | 0.024191 | | | | -3.47965 |
| FLJ30655 -1.02165 | | | | 3.200822 | | -2.97759 | | 0.006445 | | | 0.024277 | | | | -3.48415 |
| BTNL9 -1.74519 | | | | 1.10667 | | -2.96049 | | 0.008949 | | | 0.03208 | | | | -3.49533 |
| TMEM151 3.482337 | | | | 3.018051 | | 2.924723 | | 0.009649 | | | 0.03417 | | | | -3.49548 |
| LOC441931 -1.47431 | | | | 1.923853 | | -2.96973 | | 0.010437 | | | 0.036601 | | | | -3.50172 |
| TSPAN32 -2.62363 | | | | 2.128612 | | -2.9055 | | 0.013649 | | | 0.04569 | | | | -3.5019 |
| YKT6 1.791205 | | | | 3.746677 | | 2.963785 | | 0.007272 | | | 0.026949 | | | | -3.50739 |
| COLEC10 1.400826 | | | | 3.853111 | | 2.962473 | | 0.006682 | | | 0.02508 | | | | -3.51837 |
| KRT7 -2.31689 | | | | 2.084936 | | -2.94581 | | 0.008444 | | | 0.030609 | | | | -3.5287 |
| LDLRAD1 1.830921 | | | | 0.870669 | | 2.926832 | | 0.013127 | | | 0.044174 | | | | -3.54067 |
| TPK1 1.386591 | | | | 4.251477 | | 2.885158 | | 0.010035 | | | 0.03537 | | | | -3.56097 |
| DKFZp761E198 -1.03738 | | | | 3.731071 | | -2.94326 | | 0.006994 | | | 0.026082 | | | | -3.56172 |
| FLJ14054 | | 1.220346 | | 4.96944 | | 2.94037 | | 0.007232 | | | 0.026825 | | | | -3.56186 |
| DCTN6 | | 1.237699 | | 4.803563 | | 2.939401 | | 0.007059 | | | 0.026289 | | | | -3.57043 |
| KIAA1632 | | 1.100486 | | 3.779916 | | 2.938737 | | 0.00707 | | | 0.026319 | | | | -3.57192 |
| DNASE1 | | 1.167173 | | 2.616467 | | 2.919697 | | 0.008607 | | | 0.031093 | | | | -3.57821 |
| GGTL3 | | -1.63678 | | 3.376717 | | -2.9348 | | 0.007136 | | | 0.026526 | | | | -3.58078 |
| RALGPS1 | | 1.166128 | | 3.385448 | | 2.93361 | | 0.007156 | | | 0.02659 | | | | -3.58346 |
| IRX4 | | 1.542509 | | 2.071888 | | 2.924134 | | 0.008854 | | | 0.031794 | | | | -3.58487 |
| raptor | | -3.32197 | | 0.248966 | | -2.91267 | | 0.009896 | | | 0.034939 | | | | -3.59242 |
| MLN | | 1.315241 | | 2.827923 | | 2.918692 | | 0.008626 | | | 0.031156 | | | | -3.59502 |
| ITIH3 | | 1.123487 | | 4.053575 | | 2.918985 | | 0.00782 | | | 0.02867 | | | | -3.60062 |
| HIST1H4J | | -1.1896 | | 2.936901 | | -2.92168 | | 0.007362 | | | 0.027235 | | | | -3.61027 |
| HOXA6 | | 1.434984 | | 3.386236 | | 2.913809 | | 0.007914 | | | 0.028937 | | | | -3.612 |
| MAML2 | | -1.72501 | | 3.531465 | | -2.91015 | | 0.008791 | | | 0.031633 | | | | -3.61315 |
| BMP6 | | 1.152025 | | 3.506224 | | 2.908647 | | 0.009159 | | | 0.032677 | | | | -3.61728 |
| POLH | | 1.404641 | | 1.688756 | | 2.896265 | | 0.010243 | | | 0.036032 | | | | -3.62563 |
| BACH2 | | -1.111 | | 2.610776 | | -2.90593 | | 0.008302 | | | 0.03017 | | | | -3.62963 |
| HAMP | | -2.24806 | | 2.34402 | | -2.86346 | | 0.011504 | | | 0.03965 | | | | -3.6336 |
| CDH4 | | -2.23155 | | 0.778593 | | -2.88768 | | 0.01309 | | | 0.044066 | | | | -3.63538 |
| NSUN5C | | 1.865617 | | 3.874993 | | 2.900829 | | 0.007734 | | | 0.028407 | | | | -3.65702 |
| OR2A9P | | -1.02251 | | 3.68764 | | -2.90031 | | 0.007744 | | | 0.02843 | | | | -3.65819 |
| ZNF628 | | -1.58299 | | 2.662508 | | -2.88972 | | 0.00889 | | | 0.03191 | | | | -3.6637 |
| TGM1 | | 1.592447 | | 2.036941 | | 2.887838 | | 0.008928 | | | 0.032013 | | | | -3.66774 |
| ITGA7 | | 1.075194 | | 2.670305 | | 2.883412 | | 0.008264 | | | 0.030058 | | | | -3.68835 |
| ANKRD23 | | 1.381418 | | 3.288333 | | 2.875408 | | 0.008648 | | | 0.03123 | | | | -3.69619 |
| SENP3 | | 1.218683 | | 4.401351 | | 2.879538 | | 0.008133 | | | 0.029657 | | | | -3.70457 |
| AVIL | | -1.12298 | | 2.955009 | | -2.87493 | | 0.008429 | | | 0.030568 | | | | -3.71044 |
| TSPAN16 | | 1.636106 | | 1.311409 | | 2.858183 | | 0.012249 | | | 0.04175 | | | | -3.71209 |
| HSPA12B | | -1.91277 | | 1.899704 | | -2.86387 | | 0.009422 | | | 0.03347 | | | | -3.71898 |
| MMP3 | | 1.209525 | | 5.397182 | | 2.871574 | | 0.008288 | | | 0.030123 | | | | -3.72232 |
| PCDHA2 | | -1.5934 | | 1.459184 | | -2.8431 | | 0.012625 | | | 0.04281 | | | | -3.72419 |
| CASC5 | | -2.22658 | | 2.688474 | | -2.86177 | | 0.009181 | | | 0.032749 | | | | -3.72525 |
| EBI2 | | 1.359416 | | 1.965136 | | 2.827302 | | 0.01303 | | | 0.043934 | | | | -3.77186 |
| KLRA1 | | -1.7131 | | 1.743251 | | -2.83008 | | 0.010867 | | | 0.037845 | | | | -3.78074 |
| LIN28B | | 2.15699 | | 2.433119 | | 2.824173 | | 0.011431 | | | 0.039459 | | | | -3.78386 |
| DUSP10 | | -1.00896 | | 4.751687 | | -2.84061 | | 0.008914 | | | 0.031975 | | | | -3.79109 |
| LMO6 | | -1.76859 | | 2.498482 | | -2.8174 | | 0.012643 | | | 0.042851 | | | | -3.79585 |
| PCDHB16 | | 1.813597 | | 3.087337 | | 2.783091 | | 0.01203 | | | 0.041164 | | | | -3.80943 |
| HIST1H3C | | 1.275552 | | 3.276052 | | 2.809478 | | 0.010981 | | | 0.038197 | | | | -3.81044 |
| KIAA1193 | | 1.029207 | | 5.292433 | | 2.831312 | | 0.00911 | | | 0.032531 | | | | -3.81165 |
| LUM | | 1.269941 | | 7.821186 | | 2.823894 | | 0.00927 | | | 0.033013 | | | | -3.82804 |
| ZNF28 | | 1.816072 | | 2.596446 | | 2.8051 | | 0.011471 | | | 0.039558 | | | | -3.83234 |
| LOC442582 | | 1.342974 | | 4.662554 | | 2.802026 | | 0.010819 | | | 0.037688 | | | | -3.85033 |
| TXN2 | | 1.324991 | | 4.874664 | | 2.798802 | | 0.010311 | | | 0.03621 | | | | -3.86993 |
| FLJ10260 | | -1.05549 | | 4.061928 | | -2.80336 | | 0.009727 | | | 0.034403 | | | | -3.8733 |
| C12orf30 | | 1.782278 | | 2.790532 | | 2.786144 | | 0.011209 | | | 0.038834 | | | | -3.87505 |
| FLJ46688 | | 1.677927 | | 1.76652 | | 2.791596 | | 0.010482 | | | 0.036714 | | | | -3.87811 |
| CILP2 | | -1.49236 | | 1.759238 | | -2.77673 | | 0.012196 | | | 0.041618 | | | | -3.89072 |
| ADPRHL1 | | 1.678248 | | 3.162726 | | 2.764795 | | 0.012112 | | | 0.041393 | | | | -3.90352 |
| FLJ21687 | | 2.330311 | | 2.482559 | | 2.77246 | | 0.01095 | | | 0.038105 | | | | -3.91929 |
| C6orf26 | | -1.42697 | | 2.540606 | | -2.77943 | | 0.01052 | | | 0.03683 | | | | -3.91953 |
| NRF1 | | -1.25644 | | 2.558229 | | -2.77173 | | 0.011255 | | | 0.038955 | | | | -3.92213 |
| MGC48628 | | 1.687753 | | 1.115718 | | 2.749135 | | 0.014533 | | | 0.048125 | | | | -3.92224 |
| TMEM125 | | 1.436286 | | 1.529214 | | 2.757698 | | 0.012707 | | | 0.043002 | | | | -3.92975 |
| NDP | | -1.62173 | | 1.899201 | | -2.75346 | | 0.013284 | | | 0.044657 | | | | -3.9332 |
| HSPA1L | | 1.040454 | | 4.310225 | | 2.775269 | | 0.010385 | | | 0.036427 | | | | -3.93493 |
| CYP11B2 | | 1.957666 | | 1.944974 | | 2.765915 | | 0.011114 | | | 0.038537 | | | | -3.9407 |
| ZNF23 | | 1.01319 | | 4.865479 | | 2.770614 | | 0.010498 | | | 0.036764 | | | | -3.94511 |
| AGPAT5 | | -1.14278 | | 5.08823 | | -2.76658 | | 0.010597 | | | 0.037041 | | | | -3.95393 |
| SMCR7 | | 1.043574 | | 2.181705 | | 2.749746 | | 0.012153 | | | 0.041487 | | | | -3.96038 |
| ZNF181 | | 1.563439 | | 2.657065 | | 2.756368 | | 0.011093 | | | 0.038487 | | | | -3.96614 |
| GADD45GIP1 | | 1.407288 | | 3.896635 | | 2.745702 | | 0.011934 | | | 0.0409 | | | | -3.97337 |
| PIF1 | | -1.09572 | | 3.88292 | | -2.75485 | | 0.01089 | | | 0.037913 | | | | -3.97952 |
| GPR30 | | -1.29527 | | 6.515776 | | -2.75365 | | 0.010921 | | | 0.038011 | | | | -3.98215 |
| TNP1 | | -1.6489 | | 2.262656 | | -2.71684 | | 0.014353 | | | 0.047649 | | | | -3.98273 |
| EID3 | | -1.31707 | | 3.890332 | | -2.73911 | | 0.011295 | | | 0.039047 | | | | -4.01378 |
| PLEKHH2 | | -1.50225 | | 3.246837 | | -2.70843 | | 0.013698 | | | 0.045793 | | | | -4.02001 |
| UCK1 | | -1.05569 | | 3.919039 | | -2.73249 | | 0.01147 | | | 0.039558 | | | | -4.02816 |
| FLJ13984 | | 1.067729 | | 2.764984 | | 2.725958 | | 0.011895 | | | 0.040801 | | | | -4.03169 |
| SBK1 | | -2.94104 | | 3.199988 | | -2.72758 | | 0.011601 | | | 0.03992 | | | | -4.03882 |
| CCDC117 | | 1.434987 | | 2.162545 | | 2.694031 | | 0.015061 | | | 0.049559 | | | | -4.05262 |
| C20orf107 | | -1.10689 | | 3.374258 | | -2.7148 | | 0.012203 | | | 0.041633 | | | | -4.05902 |
| OR4F4 | | 1.287306 | | 2.47672 | | 2.701373 | | 0.013525 | | | 0.045309 | | | | -4.06134 |
| CEP170 | | -1.01654 | | 4.494784 | | -2.70799 | | 0.012138 | | | 0.041462 | | | | -4.08121 |
| BEX2 | | 1.273276 | | 3.583532 | | 2.699799 | | 0.012628 | | | 0.042813 | | | | -4.08779 |
| FOXO1A | | 1.033412 | | 3.42695 | | 2.683845 | | 0.014057 | | | 0.046793 | | | | -4.08889 |
| FRS2 | | -1.42957 | | 2.568391 | | -2.68785 | | 0.013934 | | | 0.046454 | | | | -4.08943 |
| RCCD1 | | -1.08306 | | 3.748388 | | -2.70256 | | 0.012291 | | | 0.041872 | | | | -4.09294 |
| AOC2 | | 1.99773 | | 2.13701 | | 2.682903 | | 0.013733 | | | 0.045881 | | | | -4.09336 |
| ATP12A | | 1.272613 | | 2.417617 | | 2.691729 | | 0.012863 | | | 0.043446 | | | | -4.1084 |
| FLJ34503 | | 1.785299 | | 1.961994 | | 2.677719 | | 0.014248 | | | 0.047345 | | | | -4.11041 |
| XDH | | 1.088303 | | 3.713593 | | 2.691596 | | 0.012606 | | | 0.042762 | | | | -4.11657 |
| CLIC5 | | -1.21481 | | 2.83242 | | -2.68158 | | 0.013164 | | | 0.044263 | | | | -4.13006 |
| C2orf3 | | 1.207205 | | 3.818281 | | 2.671474 | | 0.014087 | | | 0.046873 | | | | -4.13347 |
| JMJD2A | | -1.14545 | | 3.346298 | | -2.67659 | | 0.013048 | | | 0.043961 | | | | -4.14883 |
| CCDC104 | | 1.264863 | | 4.183714 | | 2.667502 | | 0.013324 | | | 0.044735 | | | | -4.16832 |
| SLC6A7 | | 1.538707 | | 3.22234 | | 2.658519 | | 0.01417 | | | 0.047123 | | | | -4.1689 |
| THTPA | | 1.150341 | | 2.90755 | | 2.643158 | | 0.015001 | | | 0.049419 | | | | -4.19246 |
| HIST1H3H | | -1.06225 | | 4.349024 | | -2.64635 | | 0.013985 | | | 0.046583 | | | | -4.21355 |
| MGC20470 | | -1.79478 | | 2.425236 | | -2.63094 | | 0.015074 | | | 0.049593 | | | | -4.22674 |
| SART1 | | -1.14445 | | 2.920691 | | -2.62854 | | 0.015155 | | | 0.049832 | | | | -4.23177 |
| ADI1 | | 1.182835 | | 5.92496 | | 2.629953 | | 0.014519 | | | 0.048089 | | | | -4.24846 |
| STRN | | -1.00518 | | 3.03259 | | -2.61452 | | 0.015039 | | | 0.049516 | | | | -4.28121 |
