# Supplementary material for: Identification of SCARA3 with potential roles in metabolic disorders
Source: Aging (Albany NY). 2020 Dec 9;13(2):2149–67. doi: 10.18632/aging.202228 (PMC7880357; doi:10.18632/aging.202228)
Supplement: Supplementary Table 6 [file aging-13-202228-s007.docx]

**Supplementary Table 6: DEGs during adipogenesis from GSE80614. (Adjust p-value <=0.05, |logFC|>1.**

| GENE | logFC | | | adj.P.Val | | P.Value | | | t | | | B | |
| --- | --- | --- | --- | --- | --- | --- | --- | --- | --- | --- | --- | --- | --- |
| HSD11B1 | 5.861248 | | | 1.69E-08 | | 3.47E-13 | | | 160.8472 | | | 16.96403 | |
| MAOA | 5.814996 | | | 3.90E-07 | | 2.40E-10 | | | 60.10634 | | | 14.19154 | |
| ADH1A | 5.750115 | | | 2.07E-07 | | 8.89E-11 | | | 69.80098 | | | 14.8345 | |
| FABP4 | 5.710834 | | | 1.45E-06 | | 1.72E-09 | | | 44.67263 | | | 12.68149 | |
| CFD | 5.609211 | | | 4.95E-07 | | 3.55E-10 | | | 56.65815 | | | 13.91483 | |
| CIDEC | 5.504896 | | | 3.79E-08 | | 3.88E-12 | | | 111.8321 | | | 16.31516 | |
| LPL | 5.346442 | | | 2.61E-07 | | 1.28E-10 | | | 66.04623 | | | 14.6065 | |
| IGFBP2 | 5.164513 | | | 1.17E-05 | | 5.84E-08 | | | 26.21203 | | | 9.36989 | |
| PLIN1 | 4.755945 | | | 3.79E-08 | | 2.12E-12 | | | 122.528 | | | 16.51304 | |
| G0S2 | 4.708962 | | | 2.31E-06 | | 3.36E-09 | | | 40.37073 | | | 12.10325 | |
| CEBPA | 4.6977 | | | 3.79E-08 | | 2.49E-12 | | | 119.5739 | | | 16.46269 | |
| VCAM1 | 4.601748 | | | 5.36E-08 | | 6.58E-12 | | | 103.2895 | | | 16.12124 | |
| TIMP4 | 4.593743 | | | 2.27E-07 | | 1.02E-10 | | | 68.33632 | | | 14.74842 | |
| ACACB | 4.266389 | | | 1.36E-07 | | 5.00E-11 | | | 76.12145 | | | 15.1686 | |
| LEPR | 4.215336 | | | 4.08E-06 | | 8.44E-09 | | | 35.12622 | | | 11.26481 | |
| FKBP5 | 4.117188 | | | 5.84E-07 | | 5.00E-10 | | | 53.81619 | | | 13.66373 | |
| S1PR3 | 4.028616 | | | 3.79E-08 | | 3.66E-12 | | | 112.8538 | | | 16.33603 | |
| FABP5 | 3.880416 | | | 6.88E-06 | | 2.38E-08 | | | 30.02838 | | | 10.26875 | |
| COL11A1 | 3.874177 | | | 1.68E-05 | | 9.89E-08 | | | 24.19822 | | | 8.828895 | |
| PPP1R14A 3.861178 | | | | 2.64E-06 | | 4.06E-09 | | | 39.22852 | | | 11.93426 | |
| MME | | 3.852541 | | 8.51E-08 | | 2.00E-11 | | | 87.40295 | | | 15.64238 | |
| PPARG | | 3.819079 | | 4.47E-07 | | 3.02E-10 | | | 58.05821 | | | 14.03068 | |
| GPX3 | | 3.644466 | | 7.16E-06 | | 2.63E-08 | | | 29.58755 | | | 10.17239 | |
| ACTG2 | | 3.587488 | | 1.38E-06 | | 1.56E-09 | | | 45.31008 | | | 12.76005 | |
| C1QTNF1 | | 3.506042 | | 4.00E-06 | | 8.11E-09 | | | 35.34199 | | | 11.30268 | |
| HP | | 3.45764 | | 4.47E-07 | | 3.01E-10 | | | 58.08373 | | | 14.03275 | |
| FAM89A | | 3.400066 | | 2.07E-07 | | 8.48E-11 | | | 70.2984 | | | 14.86294 | |
| RAP2A | | 3.395101 | | 6.06E-06 | | 1.85E-08 | | | 31.19093 | | | 10.51443 | |
| IGFBP5 | | 3.388271 | | 6.75E-06 | | 2.26E-08 | | | 30.26591 | | | 10.31992 | |
| MMD | | 3.361086 | | 3.45E-06 | | 6.34E-09 | | | 36.68463 | | | 11.53138 | |
| SLC7A10 | | 3.320838 | | 1.66E-05 | | 9.61E-08 | | | 24.30298 | | | 8.858334 | |
| SUSD2 | | 3.300632 | | 5.57E-06 | | 1.52E-08 | | | 32.15205 | | | 10.7088 | |
| ALPL | | 3.300117 | | 2.60E-06 | | 3.94E-09 | | | 39.41973 | | | 11.96304 | |
| FMO3 | | 3.238929 | | 4.63E-06 | | 1.04E-08 | | | 34.02033 | | | 11.06567 | |
| GPD1L | | 3.22388 | | 1.34E-06 | | 1.45E-09 | | | 45.82361 | | | 12.82211 | |
| MYH11 | | 3.220928 | | 4.34E-06 | | 9.38E-09 | | | 34.57453 | | | 11.16654 | |
| ANGPT1 | | 3.186043 | | 8.51E-08 | | 2.19E-11 | | | 86.18681 | | | 15.59761 | |
| OLFML3 | | 3.18234 | | 3.23E-07 | | 1.92E-10 | | | 62.15501 | | | 14.34288 | |
| RASD1 | | 3.173052 | | 1.82E-05 | | 1.13E-07 | | | 23.72693 | | | 8.694587 | |
| COMP | | 3.156869 | | 5.23E-05 | | 5.08E-07 | | | 18.85709 | | | 7.098363 | |
| ZBTB16 | | 3.156474 | | 6.38E-05 | | 7.00E-07 | | | 17.95263 | | | 6.751772 | |
| APOE | | 3.136106 | | 4.68E-05 | | 4.38E-07 | | | 19.2919 | | | 7.258628 | |
| FZD4 | | 3.110775 | | 9.90E-07 | | 9.74E-10 | | | 48.66511 | | | 13.14681 | |
| IGF2 | | 3.105265 | | 0.000185 | | 3.14E-06 | | | 14.24992 | | | 5.110444 | |
| BRINP1 | | 3.088742 | | 1.28E-05 | | 6.62E-08 | | | 25.71921 | | | 9.242187 | |
| TMEM64 | | 3.016803 | | 6.06E-06 | | 1.85E-08 | | | 31.2057 | | | 10.51748 | |
| SRPX | | 3.006871 | | 1.88E-05 | | 1.18E-07 | | | 23.56345 | | | 8.647272 | |
| SCD | | 2.987187 | | 2.72E-07 | | 1.45E-10 | | | 64.8606 | | | 14.5293 | |
| AKR1C2 | | 2.983455 | | | 4.02E-05 | | | 3.51E-07 | | 19.95374 | | | 7.495134 |
| STOM | | 2.929978 | | | 0.000299 | | | 6.19E-06 | | 12.82738 | | | 4.36063 |
| DHRS3 | | 2.919394 | | | 1.17E-05 | | | 5.85E-08 | | 26.20484 | | | 9.368048 |
| GHR | | 2.910961 | | | 1.43E-05 | | | 7.83E-08 | | 25.06969 | | | 9.069338 |
| C10orf54 | | 2.866873 | | | 6.91E-06 | | | 2.42E-08 | | 29.95485 | | | 10.2528 |
| CAT | | 2.863433 | | | 8.21E-06 | | | 3.11E-08 | | 28.83737 | | | 10.00419 |
| GAS1 | | 2.861135 | | | 4.13E-06 | | | 8.62E-09 | | 35.01592 | | | 11.24533 |
| SVEP1 | | 2.856942 | | | 0.000144 | | | 2.23E-06 | | 15.02099 | | | 5.486029 |
| DDIT4L | | 2.839509 | | | 2.23E-05 | | | 1.51E-07 | | 22.68407 | | | 8.38605 |
| PCSK5 | | 2.819867 | | | 9.59E-06 | | | 4.05E-08 | | 27.71031 | | | 9.740948 |
| PLA2G16 | | 2.79196 | | | 6.06E-06 | | | 1.88E-08 | | 31.13445 | | | 10.50277 |
| PLPP1 | | 2.776749 | | | 1.46E-05 | | | 8.13E-08 | | 24.92767 | | | 9.030833 |
| TSC22D3 | | 2.758899 | | | 3.91E-06 | | | 7.86E-09 | | 35.50893 | | | 11.33176 |
| MRAP | | 2.726691 | | | 4.84E-06 | | | 1.16E-08 | | 33.46489 | | | 10.96234 |
| ADH1B | | 2.679752 | | | 2.20E-06 | | | 3.11E-09 | | 40.85109 | | | 12.17226 |
| MARC1 | | 2.67059 | | | 4.84E-06 | | | 1.17E-08 | | 33.4327 | | | 10.95628 |
| ADAMTS9 | | 2.663165 | | | 2.65E-05 | | | 1.96E-07 | | 21.80727 | | | 8.113761 |
| C10orf10 | | 2.638188 | | | 0.000121 | | | 1.74E-06 | | 15.60647 | | | 5.758289 |
| SLC19A3 | | 2.637071 | | | 9.07E-06 | | | 3.69E-08 | | 28.09973 | | | 9.83338 |
| CES1 | | 2.60876 | | | 3.06E-05 | | | 2.41E-07 | | 21.13394 | | | 7.896087 |
| SLC40A1 | | 2.579722 | | | 0.000292 | | | 5.97E-06 | | 12.8979 | | | 4.399703 |
| FHL1 | | 2.576744 | | | 5.48E-05 | | | 5.61E-07 | | 18.57066 | | | 6.990581 |
| CHI3L2 | | 2.564239 | | | 5.49E-06 | | | 1.44E-08 | | 32.40407 | | | 10.75852 |
| EPHX1 | | 2.546683 | | | 1.07E-05 | | | 4.73E-08 | | 27.05996 | | | 9.582962 |
| SAA1 | | 2.545408 | | | 2.29E-07 | | | 1.08E-10 | | 67.7764 | | | 14.71456 |
| PNPLA2 | | 2.514871 | | | 0.00036 | | | 7.98E-06 | | 12.32816 | | | 4.077886 |
| TSPAN18 | | 2.510316 | | | 0.000179 | | | 3.01E-06 | | 14.34488 | | | 5.1578 |
| CD36 | | 2.507092 | | | 2.81E-07 | | | 1.61E-10 | | 63.82946 | | | 14.45998 |
| ALDH4A1 | | 2.48697 | | | 1.15E-05 | | | 5.38E-08 | | 26.54274 | | | 9.453973 |
| CRISPLD2 | | 2.486517 | | | 1.78E-05 | | | 1.08E-07 | | 23.89043 | | | 8.741531 |
| ANGPTL2 | | 2.474024 | | | 9.62E-05 | | | 1.29E-06 | | 16.34761 | | | 6.088176 |
| PGD | | 2.455015 | | | 0.001421 | | | 4.98E-05 | | 9.225152 | | | 2.030338 |
| ACKR3 | | 2.443302 | | | 6.75E-06 | | | 2.19E-08 | | 30.41642 | | | 10.35209 |
| TNMD | | 2.426016 | | | 4.49E-05 | | | 4.15E-07 | | 19.44573 | | | 7.314382 |
| RETSAT | | 2.387088 | | | 0.000768 | | | 2.19E-05 | | 10.52094 | | | 2.953254 |
| ALDH1L1 | | 2.373151 | | | 5.04E-06 | | | 1.24E-08 | | 33.14498 | | | 10.90178 |
| SULF2 | | 2.353721 | | | 6.75E-06 | | | 2.29E-08 | | 30.21219 | | | 10.30839 |
| AGTR1 | | 2.346739 | | | 1.05E-05 | | | 4.55E-08 | | 27.21895 | | | 9.62201 |
| CDO1 | | 2.342982 | | | 3.05E-05 | | | 2.38E-07 | | 21.17279 | | | 7.908859 |
| LEP | | 2.329178 | | | 1.99E-05 | | | 1.29E-07 | | 23.25079 | | | 8.555704 |
| LMOD1 | | 2.324681 | | | 1.53E-05 | | | 8.62E-08 | | 24.70648 | | | 8.970345 |
| CXCL8 | | 2.321243 | | | 8.42E-06 | | | 3.24E-08 | | 28.66539 | | | 9.964857 |
| ACSL1 | | 2.319289 | | | 0.000291 | | | 5.92E-06 | | 12.91705 | | | 4.410274 |
| SLC2A12 | | 2.314519 | | | 7.67E-07 | | | 7.23E-10 | | 50.9012 | | | 13.38177 |
| NPR3 | | 2.29171 | | | 5.64E-05 | | | 5.83E-07 | | 18.46619 | | | 6.950818 |
| APCDD1 | | 2.27546 | | | 3.75E-06 | | | 7.20E-09 | | 35.97986 | | | 11.41279 |
| LRRC8B | | 2.266762 | | | 4.64E-06 | | | 1.06E-08 | | 33.96305 | | | 11.05512 |
| TRNP1 | | 2.246331 | | | 0.000112 | | | 1.56E-06 | | 15.87638 | | | 5.880276 |
| VMO1 | | 2.233347 | | | 2.22E-06 | | | 3.18E-09 | | 40.70431 | | | 12.1513 |
| HNMT | | 2.231704 | | | 8.93E-06 | | | 3.59E-08 | | 28.22255 | | | 9.862204 |
| GPC4 | | 2.230212 | | | 0.000297 | | | 6.10E-06 | | 12.85634 | | | 4.376703 |
| GLIS3 | | 2.225693 | | | 0.000238 | | | 4.52E-06 | | 13.46857 | | | 4.708337 |
| BBOX1 | | 2.224787 | | | 4.74E-06 | | | 1.11E-08 | | 33.71856 | | | 11.00981 |
| C1R | | 2.216215 | | | 7.81E-05 | | | 9.60E-07 | | 17.10549 | | | 6.409727 |
| ATP1A2 | | 2.21561 | | | 9.99E-06 | | | 4.30E-08 | | 27.45638 | | | 9.679807 |
| TUBB2B | | 2.20359 | | | 5.30E-06 | | | 1.36E-08 | | 32.69286 | | | 10.81487 |
| FAM134B | | 2.1919 | | | 2.71E-06 | | | 4.28E-09 | | 38.92698 | | | 11.88846 |
| GLDN | | 2.191461 | | | 1.29E-06 | | | 1.38E-09 | | 46.18237 | | | 12.86483 |
| SPON1 | | 2.188951 | | | 1.67E-05 | | | 9.76E-08 | | 24.24615 | | | 8.842382 |
| PDK4 | | 2.180187 | | | 5.57E-06 | | | 1.50E-08 | | 32.19766 | | | 10.71784 |
| KITLG | | 2.1609 | | | 0.001751 | | | 6.57E-05 | | 8.824014 | | | 1.721032 |
| CRYAB | | 2.159428 | | | 8.69E-07 | | | 8.36E-10 | | 49.79541 | | | 13.26772 |
| FOXO1 | | 2.151026 | | | 1.45E-05 | | | 7.97E-08 | | 25.00144 | | | 9.050866 |
| RGS2 | | 2.146059 | | | 0.000116 | | | 1.65E-06 | | 15.74656 | | | 5.821874 |
| PAPPA | | 2.130659 | | | 1.05E-05 | | | 4.57E-08 | | 27.20541 | | | 9.618697 |
| SORT1 | | 2.114467 | | | 8.67E-05 | | | 1.10E-06 | | 16.74148 | | | 6.257201 |
| CITED4 | | 2.09913 | | | 0.000165 | | | 2.70E-06 | | 14.59049 | | | 5.278819 |
| VCAN | | 2.094093 | | | 2.27E-05 | | | 1.55E-07 | | 22.6041 | | | 8.361719 |
| ACTA2 | | 2.084315 | | | 1.17E-05 | | | 5.80E-08 | | 26.23904 | | | 9.376805 |
| CNN1 | | 2.07215 | | | 1.09E-05 | | | 4.87E-08 | | 26.94064 | | | 9.553472 |
| FASN | | 2.070062 | | | 3.77E-05 | | | 3.23E-07 | | 20.21109 | | | 7.58477 |
| SH3PXD2A 2.064261 | | | | | 1.75E-05 | | | 1.05E-07 | | 23.96689 | | | 8.763358 |
| CYB5A | | 2.062799 | | | 1.17E-05 | | | 5.83E-08 | | 26.2186 | | | 9.371572 |
| TNFRSF21 | | 2.055996 | | | 9.98E-05 | | | 1.35E-06 | | 16.23665 | | | 6.039789 |
| SSBP2 | | 2.055634 | | | 3.84E-06 | | | 7.64E-09 | | 35.66362 | | | 11.35854 |
| SERPINF1 | | 2.047217 | | | 5.28E-05 | | | 5.16E-07 | | 18.81267 | | | 7.081765 |
| SERPING1 | | 2.027424 | | | 0.000291 | | | 5.92E-06 | | 12.9142 | | | 4.408703 |
| SEPT4 | | 2.026271 | | | 3.72E-06 | | | 6.94E-09 | | 36.18276 | | | 11.44726 |
| CD302 | | 2.020051 | | | 0.000113 | | | 1.58E-06 | | 15.84855 | | | 5.867799 |
| STAT5A | | 2.018268 | | | 0.00067 | | | 1.82E-05 | | 10.8298 | | | 3.157789 |
| WFDC1 | | 2.011713 | | | 4.34E-06 | | | 9.43E-09 | | 34.54755 | | | 11.16168 |
| RGCC | | 2.010449 | | | 6.75E-06 | | | 2.30E-08 | | 30.1777 | | | 10.30098 |
| FOXC1 | | 2.002106 | | | 1.24E-05 | | | 6.31E-08 | | 25.90529 | | | 9.290748 |
| INSIG1 | | 1.98832 | | | 0.000142 | | | 2.19E-06 | | 15.06755 | | | 5.508076 |
| AOX1 | | 1.984141 | | | 6.45E-05 | | | 7.14E-07 | | 17.9006 | | | 6.731262 |
| LBP | | 1.969582 | | | 7.88E-05 | | | 9.75E-07 | | 17.06532 | | | 6.393065 |
| SPTSSA | | 1.960582 | | | 4.34E-06 | | | 9.33E-09 | | 34.60183 | | | 11.17145 |
| HLA-DMA | | 1.959529 | | | 0.000513 | | | 1.28E-05 | | 11.44494 | | | 3.549362 |
| PECR | | 1.9539 | | | 5.84E-07 | | | 5.01E-10 | | 53.79355 | | | 13.66164 |
| PPM1L | | 1.949228 | | | 1.38E-06 | | | 1.59E-09 | | 45.21263 | | | 12.74815 |
| GCHFR | | 1.946389 | | | 2.50E-05 | | | 1.80E-07 | | 22.0832 | | | 8.200778 |
| ALDH1A3 | | 1.942623 | | | 5.20E-05 | | | 5.04E-07 | | 18.87748 | | | 7.10597 |
| SFRP1 | | 1.935782 | | | 7.05E-05 | | | 8.25E-07 | | 17.50859 | | | 6.574683 |
| PIK3R1 | | 1.933778 | | | 0.000192 | | | 3.30E-06 | | 14.14472 | | | 5.057612 |
| CSAD | | 1.929793 | | | 0.00037 | | | 8.37E-06 | | 12.23603 | | | 4.024488 |
| IDH1 | | 1.925448 | | | 3.11E-05 | | | 2.48E-07 | | 21.03639 | | | 7.863904 |
| TSKU | | 1.911662 | | | 0.000391 | | | 8.96E-06 | | 12.10715 | | | 3.949133 |
| NDNF | | 1.90921 | | | 0.000105 | | | 1.43E-06 | | 16.09513 | | | 5.977576 |
| EDNRB | | 1.906295 | | | 9.07E-06 | | | 3.70E-08 | | 28.09133 | | | 9.831403 |
| HLA-DRA | | 1.898487 | | | 2.73E-06 | | | 4.42E-09 | | 38.73592 | | | 11.85918 |
| CDKN2C 1.892306 | | | | | 7.23E-05 | | 8.64E-07 | | | 17.3835 | | | 6.523929 |
| KLF9 1.888247 | | | | | 1.98E-05 | | 1.27E-07 | | | 23.28229 | | | 8.564994 |
| EPAS1 1.880841 | | | | | 0.000105 | | 1.44E-06 | | | 16.08061 | | | 5.971158 |
| ITGA1 1.880601 | | | | | 8.42E-06 | | 3.26E-08 | | | 28.62994 | | | 9.956712 |
| NFKBIA 1.87606 | | | | | 2.95E-05 | | 2.25E-07 | | | 21.35177 | | | 7.967352 |
| DHCR24 1.870087 | | | | | 1.47E-05 | | 8.25E-08 | | | 24.87296 | | | 9.01593 |
| AMPH 1.866022 | | | | | 0.000214 | | 3.83E-06 | | | 13.81714 | | | 4.890534 |
| ECM2 1.849096 | | | | | 3.70E-05 | | 3.16E-07 | | | 20.27682 | | | 7.607465 |
| DCXR 1.84903 | | | | | 0.000408 | | 9.51E-06 | | | 11.99371 | | | 3.882163 |
| CDC42EP4 1.848732 | | | | | 0.000112 | | 1.57E-06 | | | 15.86573 | | | 5.875503 |
| COL8A2 1.846406 | | | | | 6.91E-07 | | 6.09E-10 | | | 52.23676 | | | 13.51421 |
| DDIT4 1.845605 | | | | | 0.00013 | | 1.93E-06 | | | 15.36454 | | | 5.647082 |
| RBP4 1.842311 | | | | | 1.63E-05 | | 9.32E-08 | | | 24.41702 | | | 8.890211 |
| RASL11B 1.842019 | | | | | 2.90E-06 | | 4.93E-09 | | | 38.10323 | | | 11.76073 |
| CFAP69 1.83118 | | | | | 8.63E-05 | | 1.09E-06 | | | 16.76809 | | | 6.268468 |
| REV3L 1.827147 | | | | | 2.32E-05 | | 1.61E-07 | | | 22.47866 | | | 8.323353 |
| RPS29 1.826379 | | | | | 6.44E-05 | | 7.10E-07 | | | 17.91474 | | | 6.736843 |
| GPD1 1.818416 | | | | | 1.47E-06 | | 1.80E-09 | | | 44.34996 | | | 12.64106 |
| IFNGR1 1.816991 | | | | | 6.56E-05 | | 7.33E-07 | | | 17.82629 | | | 6.701864 |
| PCDH18 1.809182 | | | | | 0.000833 | | 2.44E-05 | | | 10.3363 | | | 2.828302 |
| LEPROT 1.796077 | | | | | 4.81E-05 | | 4.57E-07 | | | 19.16616 | | | 7.21269 |
| ALDH2 1.786474 | | | | | 1.23E-05 | | 6.27E-08 | | | 25.93237 | | | 9.297782 |
| LGMN 1.783716 | | | | | 4.38E-05 | | 3.94E-07 | | | 19.60348 | | | 7.37106 |
| FBN2 1.755948 | | | | | 0.000132 | | 1.96E-06 | | | 15.32558 | | | 5.629005 |
| MARCKSL1 1.753482 | | | | | 4.90E-05 | | 4.66E-07 | | | 19.10722 | | | 7.191043 |
| WASF3 1.744709 | | | | | 6.57E-05 | | 7.38E-07 | | | 17.80889 | | | 6.694963 |
| IRS2 1.743567 | | | | | 0.00047 | | 1.15E-05 | | | 11.64658 | | | 3.673387 |
| CREG1 1.741028 | | | | | 5.48E-05 | | 5.61E-07 | | | 18.57178 | | | 6.991005 |
| DAAM2 1.738417 | | | | | 0.00047 | | 1.15E-05 | | | 11.64522 | | | 3.672558 |
| PTX3 1.734537 | | | | | 0.00025 | | 4.84E-06 | | | 13.32487 | | | 4.631858 |
| DCN 1.727483 | | | | | 0.000906 | | 2.74E-05 | | | 10.15097 | | | 2.7008 |
| AKR1C4 1.725469 | | | | | 5.07E-05 | | 4.89E-07 | | | 18.9669 | | | 7.139216 |
| HSPB6 1.72364 | | | | | 0.003018 | | 0.000139 | | | 7.807715 | | | 0.87945 |
| KIT 1.719282 | | | | | 5.94E-05 | | 6.32E-07 | | | 18.23887 | | | 6.863457 |
| ENPP2 1.714993 | | | | | 5.88E-06 | | 1.71E-08 | | | 31.56918 | | | 10.59184 |
| LIPE 1.71439 | | | | | 1.15E-05 | | 5.47E-08 | | | 26.47081 | | | 9.435792 |
| SCD5 1.711323 | | | | | 1.29E-06 | | 1.32E-09 | | | 46.46349 | | | 12.89795 |
| CNTNAP2 1.696878 | | | | | 9.43E-05 | | 1.25E-06 | | | 16.43072 | | | 6.124196 |
| SNAI2 1.692167 | | | | | 0.000122 | | 1.77E-06 | | | 15.56862 | | | 5.741009 |
| SELENBP1 1.691679 | | | | | 2.41E-05 | | 1.70E-07 | | | 22.28296 | | | 8.263005 |
| AQP11 1.686622 | | | | | 1.63E-05 | | 9.35E-08 | | | 24.40557 | | | 8.887019 |
| MAP1LC3A 1.683605 | | | | | 0.000731 | | 2.03E-05 | | | 10.64324 | | | 3.034908 |
| IDH2 1.680127 | | | | | 6.53E-05 | | 7.25E-07 | | | 17.85732 | | | 6.714158 |
| SPRY1 1.679977 | | | | | 0.000319 | | 6.74E-06 | | | 12.6582 | | | 4.266034 |
| ROR2 1.675454 | | | | | 2.48E-06 | | 3.65E-09 | | | 39.86606 | | | 12.02944 |
| ISM1 1.671278 | | | | | 0.000215 | | 3.88E-06 | | | 13.78995 | | | 4.876487 |
| SCARA5 1.665161 | | | | | 1.18E-05 | | 5.92E-08 | | | 26.15867 | | | 9.356202 |
| AKR1C3 1.659475 | | | | | 2.89E-06 | | 4.80E-09 | | | 38.25066 | | | 11.78388 |
| RARRES3 1.654439 | | | | | 1.15E-05 | | 5.48E-08 | | | 26.46681 | | | 9.43478 |
| GYG2 1.651302 | | | | | 0.000319 | | 6.74E-06 | | | 12.65565 | | | 4.2646 |
| C1orf198 1.649684 | | | | | 1.65E-05 | | 9.48E-08 | | | 24.35157 | | | 8.871939 |
| GFRA1 1.647306 | | | | | 3.53E-05 | | 2.99E-07 | | | 20.44279 | | | 7.664402 |
| MMP28 1.642758 | | | | | 1.09E-05 | | 4.94E-08 | | | 26.88636 | | | 9.540005 |
| PDE1A 1.640945 | | | | | 1.23E-05 | | 6.23E-08 | | | 25.95573 | | | 9.30384 |
| ALDOC 1.632938 | | | | | 9.53E-05 | | 1.27E-06 | | | 16.39052 | | | 6.106799 |
| ME1 1.625756 | | | | | 0.000436 | | 1.03E-05 | | | 11.84527 | | | 3.793607 |
| SORBS2 1.622697 | | | | | 0.00019 | | 3.25E-06 | | | 14.1786 | | | 5.074672 |
| FMO4 1.614591 | | | | | 2.95E-05 | | 2.26E-07 | | | 21.33771 | | | 7.962778 |
| ARHGAP28 1.614363 | | | | | 0.000292 | | 5.97E-06 | | | 12.89809 | | | 4.399806 |
| SMARCD3 | | 1.611758 | | | 0.000142 | | 2.16E-06 | | | 15.09606 | | | 5.521543 |
| AK4 | | 1.611408 | | | 1.29E-05 | | 6.68E-08 | | | 25.68246 | | | 9.232548 |
| FAM46B | | 1.598295 | | | 0.000797 | | 2.29E-05 | | | 10.44087 | | | 2.899322 |
| TGFBR3 | | 1.596454 | | | 0.002588 | | 0.000112 | | | 8.088651 | | | 1.120943 |
| LDLR | | 1.591314 | | | 0.00034 | | 7.43E-06 | | | 12.46541 | | | 4.156714 |
| PC | | 1.589317 | | | 0.001543 | | 5.59E-05 | | | 9.057333 | | | 1.902405 |
| GPM6B | | 1.582759 | | | 1.82E-06 | | 2.38E-09 | | | 42.51766 | | | 12.40274 |
| JAM2 | | 1.581914 | | | 0.000122 | | 1.77E-06 | | | 15.57255 | | | 5.742805 |
| KLF15 | | 1.580721 | | | 0.000154 | | 2.46E-06 | | | 14.79823 | | | 5.379578 |
| HADH | | 1.573858 | | | 5.87E-06 | | 1.70E-08 | | | 31.60951 | | | 10.60003 |
| STEAP1 | | 1.573149 | | | 0.00025 | | 4.83E-06 | | | 13.32953 | | | 4.634349 |
| NRP2 | | 1.573039 | | | 0.000121 | | 1.74E-06 | | | 15.60903 | | | 5.759456 |
| FRZB | | 1.570134 | | | 2.56E-06 | | 3.83E-09 | | | 39.58959 | | | 11.98844 |
| CLEC2D | | 1.569281 | | | 0.014406 | | 0.001064 | | | 5.513175 | | | -1.39993 |
| LPCAT3 | | 1.56903 | | | 3.06E-05 | | 2.41E-07 | | | 21.13667 | | | 7.896986 |
| ABCC3 | | 1.567836 | | | 0.000692 | | 1.89E-05 | | | 10.76372 | | | 3.114496 |
| HSPA2 | | 1.564099 | | | 7.49E-06 | | 2.76E-08 | | | 29.35999 | | | 10.12194 |
| UNG | | 1.563291 | | | 0.000426 | | 1.00E-05 | | | 11.89991 | | | 3.826327 |
| RXRA | | 1.56257 | | | 1.67E-05 | | 9.84E-08 | | | 24.21659 | | | 8.834067 |
| ELOVL5 | | 1.549539 | | | 0.000266 | | 5.33E-06 | | | 13.12707 | | | 4.525233 |
| ANGPT2 | | 1.539482 | | | 1.83E-05 | | 1.14E-07 | | | 23.66451 | | | 8.676566 |
| ALDH6A1 | | 1.534721 | | | 2.23E-05 | | 1.48E-07 | | | 22.74891 | | | 8.405708 |
| HIST1H2BD 1.531706 | | | | | 8.00E-05 | | 9.95E-07 | | | 17.01274 | | | 6.371192 |
| RGS18 1.53081 | | | | | 7.45E-05 | | 9.02E-07 | | | 17.26901 | | | 6.477134 |
| GABARAPL 1.529315 | | | | | 0.000142 | | 2.20E-06 | | | 15.05614 | | | 5.502679 |
| XYLT1 1.527132 | | | | | 7.14E-05 | | 8.46E-07 | | | 17.44038 | | | 6.547054 |
| PLXNB1 1.526742 | | | | | 0.000303 | | 6.28E-06 | | | 12.79606 | | | 4.343209 |
| GPBAR1 1.523356 | | | | | 1.10E-05 | | 5.05E-08 | | | 26.79864 | | | 9.51817 |
| THRSP 1.517872 | | | | | 2.32E-05 | | 1.59E-07 | | | 22.50142 | | | 8.330331 |
| GBE1 1.507466 | | | | | 4.94E-05 | | 4.73E-07 | | | 19.06359 | | | 7.174972 |
| SLC27A1 1.498856 | | | | | 7.52E-06 | | 2.81E-08 | | | 29.29206 | | | 10.10679 |
| NR1H3 1.498363 | | | | | 1.39E-05 | | 7.51E-08 | | | 25.22872 | | | 9.112147 |
| CEBPD 1.494786 | | | | | 0.000112 | | 1.55E-06 | | | 15.89119 | | | 5.886911 |
| CFLAR 1.494784 | | | | | 4.38E-05 | | 3.96E-07 | | | 19.5899 | | | 7.366202 |
| VIT 1.48415 | | | | | 5.56E-06 | | 1.47E-08 | | | 32.30658 | | | 10.73935 |
| HMGCS1 1.478317 | | | | | 5.87E-06 | | 1.69E-08 | | | 31.62944 | | | 10.60407 |
| ASS1 1.467685 | | | | | 8.09E-05 | | 1.01E-06 | | | 16.9754 | | | 6.355615 |
| ADIPOQ 1.465722 | | | | | 6.00E-06 | | 1.80E-08 | | | 31.31644 | | | 10.54025 |
| FAM213A 1.46525 | | | | | 0.000179 | | 3.03E-06 | | | 14.33354 | | | 5.152161 |
| CTSC 1.46227 | | | | | 9.31E-05 | | 1.22E-06 | | | 16.48666 | | | 6.14833 |
| LMO3 1.461125 | | | | | 0.000141 | | 2.15E-06 | | | 15.11434 | | | 5.530163 |
| MT1X 1.460564 | | | | | 0.000289 | | 5.87E-06 | | | 12.93408 | | | 4.419667 |
| SRPX2 1.460536 | | | | | 2.23E-05 | | 1.48E-07 | | | 22.75873 | | | 8.408677 |
| MYOM1 1.457544 | | | | | 3.17E-05 | | 2.57E-07 | | | 20.91993 | | | 7.825264 |
| LAMA4 1.457181 | | | | | 0.003116 | | 0.000145 | | | 7.752081 | | | 0.830773 |
| SEPP1 1.456392 | | | | | 0.000139 | | 2.10E-06 | | | 15.16731 | | | 5.555081 |
| PPARGC1A 1.453045 | | | | | 1.09E-05 | | 4.93E-08 | | | 26.89083 | | | 9.541116 |
| SAT1 1.446772 | | | | | 0.000363 | | 8.11E-06 | | | 12.29606 | | | 4.059326 |
| STIM1 1.446726 | | | | | 0.000204 | | 3.59E-06 | | | 13.95988 | | | 4.963822 |
| DLAT 1.444535 | | | | | 0.000186 | | 3.18E-06 | | | 14.22605 | | | 5.09849 |
| ACADS 1.433907 | | | | | 0.000155 | | 2.48E-06 | | | 14.78476 | | | 5.373084 |
| TMEM140 1.430528 | | | | | 1.31E-05 | | 6.80E-08 | | | 25.61286 | | | 9.214246 |
| TSHZ1 | | 1.428982 | | | 0.000101 | | 1.36E-06 | | | 16.21062 | | | 6.028392 |
| ACSL5 | | 1.425303 | | | 0.000111 | | 1.54E-06 | | | 15.91224 | | | 5.896321 |
| SOX4 | | 1.417965 | | | 0.000354 | | 7.78E-06 | | | 12.37583 | | | 4.105359 |
| CHCHD10 | | 1.417566 | | | 0.00197 | | 7.68E-05 | | | 8.603526 | | | 1.545754 |
| PIR | | 1.41428 | | | 2.02E-05 | | 1.32E-07 | | | 23.17081 | | | 8.532052 |
| ENTPD1 | | 1.411583 | | | 1.21E-05 | | 6.07E-08 | | | 26.05979 | | | 9.330749 |
| PTH1R | | 1.411368 | | | 6.83E-06 | | 2.35E-08 | | | 30.0839 | | | 10.28076 |
| HK2 | | 1.410935 | | | 0.00707 | | 0.000425 | | | 6.473258 | | | -0.37345 |
| CIDECP | | 1.410664 | | | 0.002055 | | 8.13E-05 | | | 8.523969 | | | 1.481556 |
| FBLN2 | | 1.403392 | | | 0.00074 | | 2.07E-05 | | | 10.6145 | | | 3.015795 |
| GGT5 | | 1.399112 | | | 0.000131 | | 1.94E-06 | | | 15.3516 | | | 5.641085 |
| WFS1 | | 1.397978 | | | 0.001076 | | 3.47E-05 | | | 9.774515 | | | 2.435115 |
| GALNT15 | | 1.396681 | | | 0.000222 | | 4.10E-06 | | | 13.67657 | | | 4.817613 |
| TNS1 | | 1.393101 | | | 0.004259 | | 0.000218 | | | 7.243829 | | | 0.37239 |
| DSTN | | 1.39165 | | | 0.003289 | | 0.000156 | | | 7.660569 | | | 0.750079 |
| GCLM | | 1.390509 | | | 0.000816 | | 2.37E-05 | | | 10.38465 | | | 2.861222 |
| FAM110B | | 1.390479 | | | 3.00E-05 | | 2.32E-07 | | | 21.24631 | | | 7.932953 |
| NFIL3 | | 1.386693 | | | 0.000121 | | 1.72E-06 | | | 15.63372 | | | 5.770706 |
| EFHD1 | | 1.378208 | | | 2.43E-05 | | 1.73E-07 | | | 22.22216 | | | 8.244131 |
| IFITM1 | | 1.37587 | | | 0.002766 | | 0.000123 | | | 7.963238 | | | 1.014014 |
| APOB | | 1.375645 | | | 0.000104 | | 1.42E-06 | | | 16.11567 | | | 5.986641 |
| RFTN2 | | 1.36975 | | | 6.98E-05 | | 8.11E-07 | | | 17.55402 | | | 6.593019 |
| IFITM3 | | 1.369746 | | | 0.000364 | | 8.18E-06 | | | 12.27996 | | | 4.049997 |
| NNMT | | 1.369524 | | | 0.000156 | | 2.51E-06 | | | 14.75775 | | | 5.360055 |
| ATP8B4 | | 1.367581 | | | 3.22E-05 | | 2.62E-07 | | | 20.85847 | | | 7.804773 |
| FSIP1 | | 1.367196 | | | 7.62E-05 | | 9.31E-07 | | | 17.18706 | | | 6.443437 |
| BOC | | 1.365765 | | | 7.82E-05 | | 9.65E-07 | | | 17.09134 | | | 6.403865 |
| DCLK1 | | 1.362932 | | | 0.000234 | | 4.41E-06 | | | 13.52085 | | | 4.735961 |
| MRGPRF | | 1.362558 | | | 0.000166 | | 2.73E-06 | | | 14.56697 | | | 5.267319 |
| PPP2R1B | | 1.357873 | | | 6.73E-05 | | 7.71E-07 | | | 17.68913 | | | 6.647255 |
| ZDHHC9 | | 1.346568 | | | 2.73E-05 | | 2.04E-07 | | | 21.67289 | | | 8.070929 |
| IFITM2 | | 1.346233 | | | 0.000913 | | 2.79E-05 | | | 10.12427 | | | 2.682252 |
| CXCL12 | | 1.339319 | | | 0.002091 | | 8.32E-05 | | | 8.49222 | | | 1.455792 |
| RAP1B | | 1.338851 | | | 0.001639 | | 6.02E-05 | | | 8.948145 | | | 1.818042 |
| FMO2 | | 1.33337 | | | 8.80E-06 | | 3.47E-08 | | | 28.36778 | | | 9.896089 |
| METTL7A | | 1.33156 | | | 0.000999 | | 3.12E-05 | | | 9.941015 | | | 2.553751 |
| FTH1 | | 1.328511 | | | 6.46E-05 | | 7.16E-07 | | | 17.89177 | | | 6.727779 |
| MYLIP | | 1.323882 | | | 5.91E-06 | | 1.74E-08 | | | 31.4867 | | | 10.57507 |
| CYP26B1 | | 1.321277 | | | 0.000651 | | 1.76E-05 | | | 10.89215 | | | 3.198424 |
| SSPN | | 1.314435 | | | 0.000382 | | 8.75E-06 | | | 12.15151 | | | 3.975158 |
| EBF1 | | 1.306241 | | | 0.000616 | | 1.64E-05 | | | 11.01519 | | | 3.277962 |
| PCP4 | | 1.302967 | | | 9.31E-06 | | 3.84E-08 | | | 27.93715 | | | 9.794985 |
| NFIB | | 1.298155 | | | 0.000254 | | 4.97E-06 | | | 13.2726 | | | 4.603831 |
| CD24 | | 1.297216 | | | 0.0004 | | 9.24E-06 | | | 12.04792 | | | 3.914242 |
| H19 | | 1.295209 | | | 2.37E-05 | | 1.67E-07 | | | 22.34578 | | | 8.28244 |
| BTG2 | | 1.290445 | | | 0.000137 | | 2.05E-06 | | | 15.21603 | | | 5.577921 |
| SNCAIP | | 1.281036 | | | 5.29E-05 | | 5.23E-07 | | | 18.77442 | | | 7.067439 |
| RAPGEF1 | | 1.280286 | | | 0.000265 | | 5.30E-06 | | | 13.13954 | | | 4.532005 |
| SERPINA3 | | 1.277167 | | | 0.000152 | | 2.40E-06 | | | 14.85194 | | | 5.405393 |
| MSMO1 | | 1.277085 | | | 0.000957 | | 2.97E-05 | | | 10.02313 | | | 2.611595 |
| PRRG1 | | 1.268725 | | | 8.78E-05 | | 1.13E-06 | | | 16.68544 | | | 6.233405 |
| FAM212B | | 1.267037 | | | 7.81E-05 | | 9.57E-07 | | | 17.11357 | | | 6.413075 |
| TXNRD1 | | 1.262516 | | | 0.000319 | | 6.74E-06 | | | 12.65777 | | | 4.265791 |
| ACAT2 | | 1.259659 | | | 0.001147 | | 3.79E-05 | | | 9.637773 | | | 2.336307 |
| THNSL2 | | 1.258176 | | | 0.000304 | | 6.31E-06 | | | 12.78901 | | | 4.339281 |
| ACSS2 | | 1.242407 | | | 7.46E-05 | | 9.05E-07 | | | 17.26169 | | | 6.47413 |
| HES6 | | 1.242347 | | | 5.40E-05 | | 5.39E-07 | | | 18.68911 | | | 7.03537 |
| RASL10B | | 1.24137 | | | 6.08E-06 | | 1.91E-08 | | | 31.05375 | | | 10.48606 |
| AXIN2 | | 1.240897 | | | 5.23E-05 | | 5.09E-07 | | | 18.85142 | | | 7.096247 |
| IRF1 | | 1.240776 | | | 0.000539 | | 1.37E-05 | | | 11.32791 | | | 3.476418 |
| PDE8B | | 1.23788 | | | 5.79E-05 | | 6.08E-07 | | | 18.34757 | | | 6.905379 |
| BTG1 | | 1.233754 | | | 0.001137 | | 3.74E-05 | | | 9.658816 | | | 2.351594 |
| PAPSS1 | | 1.233469 | | | 1.89E-05 | | 1.20E-07 | | | 23.50739 | | | 8.630957 |
| NABP1 | | 1.233177 | | | 0.001992 | | 7.80E-05 | | | 8.581086 | | | 1.527697 |
| GLIPR2 | | 1.227952 | | | 0.000947 | | 2.93E-05 | | | 10.04563 | | | 2.627374 |
| EYA1 | | 1.2256 | | | 6.98E-05 | | 8.12E-07 | | | 17.55187 | | | 6.592151 |
| COL3A1 | | 1.222774 | | | 0.010279 | | 0.000689 | | | 5.953539 | | | -0.91479 |
| ST3GAL1 | | 1.220669 | | | 0.002718 | | 0.00012 | | | 7.994037 | | | 1.040404 |
| BCL6 | | 1.220492 | | | 0.002653 | | 0.000116 | | | 8.045847 | | | 1.084604 |
| LPIN1 | | 1.216247 | | | 0.000325 | | 6.94E-06 | | | 12.59882 | | | 4.232535 |
| FADS1 | | 1.215384 | | | 0.000123 | | 1.78E-06 | | | 15.5538 | | | 5.734234 |
| CTSO | | 1.213521 | | | 0.001137 | | 3.75E-05 | | | 9.654719 | | | 2.34862 |
| TCEAL3 | | 1.210803 | | | 0.000124 | | 1.81E-06 | | | 15.51252 | | | 5.715316 |
| ECHDC3 | | 1.210273 | | | 6.64E-05 | | 7.52E-07 | | | 17.75992 | | | 6.675495 |
| ARRB1 | | 1.209037 | | | 0.000109 | | 1.50E-06 | | | 15.97853 | | | 5.925885 |
| RPS23 | | 1.205807 | | | 0.002085 | | 8.29E-05 | | | 8.49637 | | | 1.459164 |
| FAM13A | | 1.199404 | | | 5.27E-05 | | 5.14E-07 | | | 18.82485 | | | 7.086322 |
| ANGPTL4 | | 1.198012 | | | 0.004768 | | 0.000254 | | | 7.062745 | | | 0.202846 |
| FBLN1 | | 1.1957 | | | 0.002915 | | 0.000132 | | | 7.870107 | | | 0.933699 |
| STAT3 | | 1.193694 | | | 0.000173 | | 2.88E-06 | | | 14.43942 | | | 5.204629 |
| MYC | | 1.190671 | | | 0.001112 | | 3.64E-05 | | | 9.702119 | | | 2.382959 |
| TCEAL4 | | 1.190657 | | | 0.000689 | | 1.88E-05 | | | 10.77656 | | | 3.122923 |
| ABHD4 | | 1.188785 | | | 0.000195 | | 3.40E-06 | | | 14.07731 | | | 5.02355 |
| PRKAG2 | | 1.187987 | | | 0.001652 | | 6.09E-05 | | | 8.93289 | | | 1.806184 |
| NCAM2 | | 1.187522 | | | 6.75E-06 | | 2.26E-08 | | | 30.26921 | | | 10.32063 |
| MOCS1 | | 1.185542 | | | 8.70E-06 | | 3.40E-08 | | | 28.4456 | | | 9.914156 |
| SOCS2 | | 1.184984 | | | 0.000369 | | 8.31E-06 | | | 12.24884 | | | 4.031933 |
| PTGER4 | | 1.182745 | | | 0.000162 | | 2.63E-06 | | | 14.64448 | | | 5.305146 |
| CCDC85A | | 1.181878 | | | 0.000239 | | 4.54E-06 | | | 13.45939 | | | 4.703476 |
| GSTA4 1.171204 | | | | | 3.06E-05 | | 2.43E-07 | | | 21.09775 | | | 7.884166 |
| PDGFD 1.171155 | | | | | 1.45E-05 | | 8.00E-08 | | | 24.9879 | | | 9.047195 |
| DUSP5 1.169518 | | | | | 0.000751 | | 2.12E-05 | | | 10.57416 | | | 2.988894 |
| CYP39A1 1.165156 | | | | | 4.49E-05 | | 4.13E-07 | | | 19.46048 | | | 7.319704 |
| HACD2 1.165098 | | | | | 0.000731 | | 2.03E-05 | | | 10.64269 | | | 3.034541 |
| CXCL16 1.164112 | | | | | 0.000856 | | 2.53E-05 | | | 10.27729 | | | 2.787931 |
| HOXD12 1.16014 | | | | | 0.00014 | | 2.13E-06 | | | 15.12868 | | | 5.536918 |
| DGAT1 1.157511 | | | | | 0.001722 | | 6.44E-05 | | | 8.851427 | | | 1.742558 |
| TXNIP 1.154398 | | | | | 0.002706 | | 0.000119 | | | 8.005057 | | | 1.049826 |
| FMOD 1.149048 | | | | | 0.000124 | | 1.81E-06 | | | 15.51299 | | | 5.715532 |
| TP53I11 1.148146 | | | | | 0.008834 | | 0.000567 | | | 6.159492 | | | -0.69636 |
| EFNA1 1.147226 | | | | | 0.000141 | | 2.14E-06 | | | 15.11795 | | | 5.531862 |
| VKORC1L1 1.146639 | | | | | 4.38E-05 | | 3.96E-07 | | | 19.58771 | | | 7.365419 |
| TMEM135 1.146033 | | | | | 0.004041 | | 0.000205 | | | 7.323065 | | | 0.445522 |
| ABCA8 | | 1.14585 | | | 2.95E-05 | | 2.26E-07 | | | 21.33113 | | | 7.960636 |
| CYBRD1 | | 1.145515 | | | 0.003731 | | 0.000183 | | | 7.460679 | | | 0.571044 |
| SSH2 | | 1.143851 | | | 0.000162 | | 2.63E-06 | | | 14.64347 | | | 5.30465 |
| ST6GAL1 | | 1.14275 | | | 2.78E-05 | | 2.09E-07 | | | 21.59874 | | | 8.047167 |
| PLTP | | 1.142357 | | | 0.017582 | | 0.001392 | | | 5.25271 | | | -1.69899 |
| SH3D19 | | 1.139018 | | | 0.000362 | | 8.08E-06 | | | 12.30365 | | | 4.063713 |
| GSN | | 1.135101 | | | 0.000392 | | 8.99E-06 | | | 12.09985 | | | 3.944844 |
| ACOT2 | | 1.134289 | | | 0.01695 | | 0.001325 | | | 5.299753 | | | -1.64429 |
| ABCA1 | | 1.130665 | | | 0.00097 | | 3.02E-05 | | | 9.99722 | | | 2.593391 |
| GBP1 | | 1.126653 | | | 0.000127 | | 1.85E-06 | | | 15.46073 | | | 5.691515 |
| PTN | | 1.126384 | | | 0.003807 | | 0.000188 | | | 7.426333 | | | 0.539891 |
| IMPA2 | | 1.124402 | | | 0.000698 | | 1.92E-05 | | | 10.7414 | | | 3.099808 |
| G6PD | | 1.11929 | | | 0.000408 | | 9.53E-06 | | | 11.98942 | | | 3.879621 |
| IL18R1 | | 1.119079 | | | 7.23E-05 | | 8.63E-07 | | | 17.38775 | | | 6.52566 |
| ACER3 | | 1.116105 | | | 0.002343 | | 9.74E-05 | | | 8.277335 | | | 1.27922 |
| SMOC1 | | 1.115446 | | | 0.003705 | | 0.000181 | | | 7.472481 | | | 0.581722 |
| PYGL | | 1.108239 | | | 0.000325 | | 6.95E-06 | | | 12.59596 | | | 4.23092 |
| FLRT3 | | 1.108201 | | | 1.16E-05 | | 5.56E-08 | | | 26.40624 | | | 9.419421 |
| PPP1R1A | | 1.104685 | | | 5.48E-05 | | 5.61E-07 | | | 18.57368 | | | 6.991727 |
| GPAM | | 1.104461 | | | 0.008013 | | 0.000503 | | | 6.288689 | | | -0.56198 |
| AGPAT2 | | 1.102667 | | | 0.005489 | | 0.000303 | | | 6.857301 | | | 0.006325 |
| TBC1D16 | | 1.09937 | | | 0.002334 | | 9.66E-05 | | | 8.288084 | | | 1.288145 |
| DGAT2 | | 1.099358 | | | 1.03E-05 | | 4.46E-08 | | | 27.30253 | | | 9.642427 |
| CMTM8 | | 1.099249 | | | 0.000469 | | 1.14E-05 | | | 11.65234 | | | 3.676896 |
| SELM | | 1.098532 | | | 0.000664 | | 1.80E-05 | | | 10.85345 | | | 3.173228 |
| SMAD6 | | 1.095678 | | | 0.019015 | | 0.001546 | | | 5.153165 | | | -1.81574 |
| PHOSPHO2 1.095252 | | | | | 0.000172 | | 2.87E-06 | | | 14.44817 | | | 5.208946 |
| RHOBTB3 1.094783 | | | | | 0.043756 | | 0.00459 | | | 4.188544 | | | -3.02021 |
| MYL9 1.094533 | | | | | 0.001528 | | 5.51E-05 | | | 9.078219 | | | 1.91844 |
| SUCLG1 1.093449 | | | | | 0.000137 | | 2.07E-06 | | | 15.20182 | | | 5.571265 |
| PISD 1.091298 | | | | | 0.000929 | | 2.85E-05 | | | 10.0849 | | | 2.654824 |
| SETMAR 1.090205 | | | | | 4.18E-06 | | 8.82E-09 | | | 34.89415 | | | 11.22373 |
| TSPAN6 1.089842 | | | | | 0.00036 | | 7.96E-06 | | | 12.33205 | | | 4.080128 |
| TTC39C 1.089724 | | | | | 0.000964 | | 2.99E-05 | | | 10.00863 | | | 2.601417 |
| NEXN 1.085226 | | | | | 0.00075 | | 2.11E-05 | | | 10.57708 | | | 2.990844 |
| IL17RC 1.085214 | | | | | 5.96E-05 | | 6.36E-07 | | | 18.21926 | | | 6.855866 |
| KCNJ8 1.083994 | | | | | 3.81E-05 | | 3.28E-07 | | | 20.16011 | | | 7.567115 |
| ISG20 1.082598 | | | | | 8.78E-05 | | 1.13E-06 | | | 16.68316 | | | 6.232438 |
| MICAL1 1.082359 | | | | | 0.004663 | | 0.000247 | | | 7.098271 | | | 0.236376 |
| TMEM261 1.080309 | | | | | 0.001169 | | 3.89E-05 | | | 9.60081 | | | 2.309381 |
| LETMD1 1.078747 | | | | | 0.000445 | | 1.06E-05 | | | 11.78452 | | | 3.757057 |
| COL4A3BP 1.078304 | | | | | 0.000748 | | 2.10E-05 | | | 10.58832 | | | 2.998349 |
| PTPRM 1.07724 | | | | | 0.001707 | | 6.38E-05 | | | 8.866212 | | | 1.754144 |
| MESP1 1.076027 | | | | | 0.000217 | | 3.94E-06 | | | 13.75981 | | | 4.860882 |
| ZNF503 1.075619 | | | | | 0.026298 | | 0.002376 | | | 4.757693 | | | -2.29334 |
| SRXN1 1.074201 | | | | | 9.22E-06 | | 3.78E-08 | | | 27.99854 | | | 9.809514 |
| CYP27A1 1.073807 | | | | | 0.000157 | | 2.53E-06 | | | 14.73105 | | | 5.34715 |
| METRN 1.073523 | | | | | 0.002066 | | 8.19E-05 | | | 8.514061 | | | 1.473524 |
| ANXA6 1.071743 | | | | | 0.002247 | | 9.19E-05 | | | 8.355532 | | | 1.343922 |
| PRICKLE2 1.071116 | | | | | 1.94E-05 | | 1.24E-07 | | | 23.37443 | | | 8.592083 |
| WDR41 1.069642 | | | | | 0.000943 | | 2.90E-05 | | | 10.05815 | | | 2.636133 |
| RBMS1 1.068435 | | | | | 0.000776 | | 2.22E-05 | | | 10.49885 | | | 2.93841 |
| MYL5 1.068387 | | | | | 0.001295 | | 4.41E-05 | | | 9.40976 | | | 2.168709 |
| C6orf48 1.067753 | | | | | 0.001534 | | 5.55E-05 | | | 9.067432 | | | 1.910162 |
| CYB561A3 1.067458 | | | | | 0.00036 | | 7.99E-06 | | | 12.32473 | | | 4.0759 |
| SESN1 1.061604 | | | | | 0.001268 | | 4.29E-05 | | | 9.448837 | | | 2.197688 |
| TMEM175 1.057251 | | | | | 0.002586 | | 0.000112 | | | 8.090829 | | | 1.122788 |
| SLC6A10P | | 1.053763 | | | 0.041798 | | 0.004324 | | | 4.238372 | | | -2.9547 |
| TMED5 | | 1.051847 | | | 0.001479 | | 5.27E-05 | | | 9.142665 | | | 1.967714 |
| PTEN | | 1.050167 | | | 0.000246 | | 4.73E-06 | | | 13.37344 | | | 4.6578 |
| ECH1 | | 1.050029 | | | 0.003995 | | 0.000201 | | | 7.345957 | | | 0.466533 |
| TAGLN | | 1.048962 | | | 0.000758 | | 2.14E-05 | | | 10.55571 | | | 2.976557 |
| OSBPL1A | | 1.048063 | | | 0.000346 | | 7.60E-06 | | | 12.42115 | | | 4.131385 |
| CMBL | | 1.047661 | | | 0.001295 | | 4.40E-05 | | | 9.410694 | | | 2.169402 |
| TLN2 | | 1.047213 | | | 0.00074 | | 2.07E-05 | | | 10.61134 | | | 3.013694 |
| CCDC126 | | 1.046856 | | | 0.001079 | | 3.49E-05 | | | 9.766376 | | | 2.429269 |
| PDLIM7 | | 1.045036 | | | 0.000414 | | 9.70E-06 | | | 11.95631 | | | 3.85995 |
| C5 | | 1.044384 | | | 0.000426 | | 1.00E-05 | | | 11.89738 | | | 3.824818 |
| VCL | | 1.04159 | | | 0.001698 | | 6.33E-05 | | | 8.876965 | | | 1.762559 |
| TPM2 | | 1.03974 | | | 0.008163 | | 0.000514 | | | 6.264064 | | | -0.58744 |
| GMDS | | 1.038485 | | | 0.002463 | | 0.000105 | | | 8.18187 | | | 1.199524 |
| ENDOD1 | | 1.037231 | | | 5.83E-05 | | 6.16E-07 | | | 18.3108 | | | 6.891229 |
| PDE4D | | 1.035535 | | | 0.000208 | | 3.70E-06 | | | 13.89477 | | | 4.930484 |
| CUL4B | | 1.034468 | | | 0.001393 | | 4.86E-05 | | | 9.263896 | | | 2.059581 |
| RSPO1 | | 1.034363 | | | 0.000113 | | 1.59E-06 | | | 15.83352 | | | 5.86105 |
| ALDH9A1 | | 1.034175 | | | 0.000215 | | 3.87E-06 | | | 13.79958 | | | 4.881467 |
| GATSL3 | | 1.033278 | | | 0.001268 | | 4.30E-05 | | | 9.447308 | | | 2.196556 |
| ALDH1B1 | | 1.03279 | | | 0.016889 | | 0.001319 | | | 5.303976 | | | -1.6394 |
| RASL11A | | 1.030642 | | | 0.00123 | | 4.12E-05 | | | 9.510015 | | | 2.242843 |
| DPYSL4 | | 1.026593 | | | 4.15E-05 | | 3.70E-07 | | | 19.79061 | | | 7.437649 |
| ZNRF3 | | 1.026375 | | | 7.29E-05 | | 8.79E-07 | | | 17.33992 | | | 6.506157 |
| ID4 | | 1.02559 | | | 0.006051 | | 0.000342 | | | 6.715691 | | | -0.13179 |
| GSR | | 1.024958 | | | 0.000319 | | 6.74E-06 | | | 12.658 | | | 4.265919 |
| OPLAH | | 1.018045 | | | 0.000115 | | 1.62E-06 | | | 15.78298 | | | 5.838307 |
| TLE1 | | 1.016693 | | | 0.000283 | | 5.72E-06 | | | 12.98587 | | | 4.448145 |
| CACHD1 | | 1.016024 | | | 2.27E-05 | | 1.55E-07 | | | 22.59654 | | | 8.359414 |
| MYLK | | 1.013605 | | | 0.001516 | | 5.44E-05 | | | 9.096132 | | | 1.932167 |
| BNIP3L | | 1.012497 | | | 0.00104 | | 3.30E-05 | | | 9.853785 | | | 2.491823 |
| GCAT | | 1.012363 | | | 8.68E-05 | | 1.11E-06 | | | 16.73395 | | | 6.254005 |
| TMEM59L | | 1.011117 | | | 0.000278 | | 5.61E-06 | | | 13.02263 | | | 4.468294 |
| PRUNE2 | | 1.01081 | | | 0.001684 | | 6.27E-05 | | | 8.891376 | | | 1.773823 |
| ITPR1 | | 1.010113 | | | 0.001328 | | 4.53E-05 | | | 9.367268 | | | 2.137075 |
| NSMF | | 1.008113 | | | 0.002959 | | 0.000135 | | | 7.844317 | | | 0.911317 |
| HES4 | | 1.007311 | | | 0.000614 | | 1.63E-05 | | | 11.0231 | | | 3.283045 |
| CHSY3 | | 1.000637 | | | 0.000217 | | 3.92E-06 | | | 13.76923 | | | 4.865766 |
| PRDM8 | | -1.00025 | | | 0.002052 | | 8.11E-05 | | | -8.52722 | | | 1.484186 |
| PPP1R18 | | -1.00114 | | | 0.002658 | | 0.000116 | | | -8.04227 | | | 1.081557 |
| CALB2 | | -1.00146 | | | 2.01E-05 | | 1.30E-07 | | | -23.2011 | | | 8.541036 |
| DDX10 | | -1.00293 | | | 0.001602 | | 5.85E-05 | | | -8.99136 | | | 1.851539 |
| SH3GLB2 | | -1.00347 | | | 0.001511 | | 5.42E-05 | | | -9.10281 | | | 1.937279 |
| YWHAH | | -1.01293 | | | 0.017387 | | 0.001372 | | | -5.26651 | | | -1.68291 |
| DBNDD1 | | -1.01325 | | | 0.000204 | | 3.60E-06 | | | -13.9554 | | | 4.961549 |
| CYR61 | | -1.01516 | | | 0.021951 | | 0.001871 | | | -4.975 | | | -2.02816 |
| TEAD4 | | -1.01675 | | | 0.002313 | | 9.53E-05 | | | -8.30634 | | | 1.303278 |
| DEPDC1 | | -1.0188 | | | 0.016289 | | 0.001261 | | | -5.34775 | | | -1.5888 |
| PRKCDBP | | -1.01888 | | | 4.92E-05 | | 4.70E-07 | | | -19.0837 | | | 7.18238 |
| WDR54 | | -1.01952 | | | 0.001451 | | 5.13E-05 | | | -9.18407 | | | 1.999213 |
| CCNF | | -1.02357 | | | 0.000261 | | 5.19E-06 | | | -13.1814 | | | 4.554704 |
| LRR1 | | -1.02507 | | | 0.002107 | | 8.44E-05 | | | -8.47273 | | | 1.439937 |
| DLX1 | | -1.02568 | | | 3.34E-05 | | 2.77E-07 | | | -20.6841 | | | 7.74626 |
| ABHD17C | | -1.02647 | | | 0.00073 | | 2.03E-05 | | | -10.646 | | | 3.036758 |
| LOX | | -1.02846 | | | 0.009122 | | 0.000591 | | | -6.1152 | | | -0.7429 |
| MATN2 | | -1.0302 | | | 0.000893 | | 2.67E-05 | | | -10.1902 | | | 2.727953 |
| NMB | | -1.03086 | | | 0.002065 | | 8.17E-05 | | | -8.51643 | | | 1.475442 |
| MYO19 | | -1.03132 | | | 0.00021 | | 3.75E-06 | | | -13.8664 | | | 4.915896 |
| CALHM2 | | -1.03172 | | | 0.00054 | | 1.38E-05 | | | -11.3165 | | | 3.469281 |
| CBX5 | | -1.03177 | | | 0.000192 | | 3.29E-06 | | | -14.151 | | | 5.060765 |
| CA12 | | -1.03288 | | | 0.001969 | | 7.67E-05 | | | -8.60489 | | | 1.546846 |
| FOXC2 | | -1.03434 | | | 0.000252 | | 4.89E-06 | | | -13.3071 | | | 4.622338 |
| TFPI | | -1.03458 | | | 6.79E-05 | | 7.81E-07 | | | -17.6561 | | | 6.634023 |
| RAB34 | | -1.03513 | | | 0.000159 | | 2.58E-06 | | | -14.6895 | | | 5.327009 |
| TMEM205 | | -1.03537 | | | 0.003126 | | 0.000146 | | | -7.74641 | | | 0.825794 |
| PKMYT1 | | -1.0364 | | | 3.31E-05 | | 2.72E-07 | | | -20.7404 | | | 7.76523 |
| H1F0 | | -1.03687 | | | 0.000109 | | 1.50E-06 | | | -15.9747 | | | 5.924178 |
| COL5A1 | | -1.03887 | | | 0.000364 | | 8.18E-06 | | | -12.2793 | | | 4.049589 |
| PTPRF | | -1.03984 | | | 0.007712 | | 0.000479 | | | -6.34181 | | | -0.50731 |
| HBEGF | | -1.04153 | | | 0.005699 | | 0.000318 | | | -6.80199 | | | -0.04736 |
| HPCAL1 | | -1.0424 | | | 0.000362 | | 8.09E-06 | | | -12.3025 | | | 4.063055 |
| FANCI | | -1.0481 | | | 0.000366 | | 8.26E-06 | | | -12.2621 | | | 4.039648 |
| MCM3 | | -1.04869 | | | 0.001445 | | 5.10E-05 | | | -9.1925 | | | 2.005605 |
| RRBP1 | | -1.05031 | | | 0.00692 | | 0.000412 | | | -6.50694 | | | -0.33948 |
| MAPK13 | | -1.05115 | | | 2.41E-05 | | 1.71E-07 | | | -22.267 | | | 8.258062 |
| TMEM184B | | -1.0514 | | | 0.003058 | | 0.000141 | | | -7.78629 | | | 0.860738 |
| GDF15 | | -1.05346 | | | 0.00057 | | 1.48E-05 | | | -11.1895 | | | 3.389238 |
| CHIC2 | | -1.05638 | | | 0.000936 | | 2.88E-05 | | | -10.0719 | | | 2.645718 |
| TMEM171 | | -1.05675 | | | 0.000217 | | 3.93E-06 | | | -13.7658 | | | 4.863993 |
| POLE2 | | | -1.06162 | | 3.05E-05 | | 2.38E-07 | | | | -21.1769 | | 7.910202 |
| LRRC32 | | | -1.06294 | | 0.00037 | | 8.37E-06 | | | | -12.2358 | | 4.024333 |
| CLCC1 | | | -1.06332 | | 0.000163 | | 2.66E-06 | | | | -14.6246 | | 5.295475 |
| DRAM1 | | | -1.06548 | | 0.000533 | | 1.35E-05 | | | | -11.3565 | | 3.494336 |
| MAP4K2 | | | -1.0656 | | 0.000557 | | 1.43E-05 | | | | -11.2501 | | 3.427559 |
| HMGN2 | | | -1.0667 | | 0.015223 | | 0.001152 | | | | -5.4356 | | -1.48804 |
| CBR3 | | | -1.06723 | | 1.74E-05 | | 1.04E-07 | | | | -24.0163 | | 8.777414 |
| DNMT1 | | | -1.06951 | | 0.00177 | | 6.68E-05 | | | | -8.80085 | | 1.702794 |
| IDS | | | -1.06982 | | 3.86E-05 | | 3.34E-07 | | | | -20.1048 | | 7.547901 |
| ADAMTS6 | | | -1.07249 | | 0.000374 | | 8.49E-06 | | | | -12.2081 | | 4.008238 |
| JUN | | | -1.07322 | | 0.001069 | | 3.44E-05 | | | | -9.78755 | | 2.444466 |
| KIF14 | | | -1.07564 | | 0.000311 | | 6.50E-06 | | | | -12.7275 | | 4.304929 |
| SCHIP1 | | | -1.07579 | | 0.000208 | | 3.70E-06 | | | | -13.8936 | | 4.929895 |
| ALDH3A1 | | | -1.07778 | | 0.001265 | | 4.28E-05 | | | | -9.45432 | | 2.201749 |
| TSPO | | | -1.07789 | | 0.000647 | | 1.74E-05 | | | | -10.9084 | | 3.209003 |
| DOCK2 | | | -1.08043 | | 2.83E-05 | | 2.14E-07 | | | | -21.5211 | | 8.02218 |
| DRAP1 | | | -1.08374 | | 0.022747 | | 0.001961 | | | | -4.93194 | | -2.08017 |
| DEK | | | -1.0847 | | 5.24E-06 | | 1.33E-08 | | | | -32.8134 | | 10.8382 |
| PYCARD | | | -1.08675 | | 0.00091 | | 2.76E-05 | | | | -10.1398 | | 2.693033 |
| TGFBR2 | | | -1.08894 | | 0.001067 | | 3.42E-05 | | | | -9.79694 | | 2.451198 |
| PROCR | | | -1.08923 | | 0.000562 | | 1.45E-05 | | | | -11.2264 | | 3.412571 |
| SLFN11 | | | -1.09191 | | 0.00116 | | 3.85E-05 | | | | -9.61428 | | 2.319202 |
| CYGB | | | -1.0934 | | 4.54E-05 | | 4.22E-07 | | | | -19.3994 | | 7.297629 |
| P4HA2 | | | -1.09416 | | 0.001706 | | 6.36E-05 | | | | -8.86887 | | 1.756227 |
| E2F2 | | | -1.09443 | | 0.000402 | | 9.31E-06 | | | | -12.0346 | | 3.90636 |
| SMAD3 | | | -1.09784 | | 0.001373 | | 4.77E-05 | | | | -9.29103 | | 2.079994 |
| FIP1L1 | | | -1.09855 | | 0.001858 | | 7.10E-05 | | | | -8.71349 | | 1.633655 |
| COMMD7 | | | -1.09905 | | 0.001149 | | 3.80E-05 | | | | -9.63498 | | 2.334272 |
| PEAR1 | | | -1.1024 | | 0.000204 | | 3.60E-06 | | | | -13.9506 | | 4.959065 |
| DONSON | | | -1.10308 | | 6.24E-05 | | 6.80E-07 | | | | -18.033 | | 6.783324 |
| BRI3 | | | -1.10372 | | 0.000939 | | 2.89E-05 | | | | -10.0661 | | 2.64171 |
| ARHGDIB | | | -1.10547 | | 0.000142 | | 2.20E-06 | | | | -15.0614 | | 5.50519 |
| MFAP2 | | | -1.10888 | | 0.000692 | | 1.89E-05 | | | | -10.7636 | | 3.114398 |
| CKS1B | | | -1.10978 | | 0.000394 | | 9.06E-06 | | | | -12.0862 | | 3.936833 |
| ITGB2 | | | -1.11003 | | 0.002252 | | 9.23E-05 | | | | -8.34951 | | 1.338956 |
| BGN | | | -1.11423 | | 0.000186 | | 3.18E-06 | | | | -14.2203 | | 5.095628 |
| MND1 | | | -1.11572 | | 5.40E-05 | | 5.39E-07 | | | | -18.6854 | | 7.03397 |
| C1GALT1 | | | -1.11855 | | 0.00617 | | 0.000351 | | | | -6.68861 | | -0.15846 |
| LOXL3 | | | -1.11862 | | 5.67E-05 | | 5.89E-07 | | | | -18.4356 | | 6.939122 |
| PRMT6 | | | -1.11888 | | 6.72E-05 | | 7.66E-07 | | | | -17.7072 | | 6.654456 |
| MLKL | | | -1.12339 | | 0.002494 | | 0.000106 | | | | -8.16065 | | 1.181699 |
| CAPRIN2 | | | -1.12441 | | 0.008144 | | 0.000513 | | | | -6.26699 | | -0.58441 |
| FARP1 | | | -1.12669 | | 0.000254 | | 4.95E-06 | | | | -13.2783 | | 4.606886 |
| EPHA2 | | | -1.12684 | | 1.32E-05 | | 6.92E-08 | | | | -25.5476 | | 9.197039 |
| MLLT11 | | | -1.12769 | | 0.000173 | | 2.88E-06 | | | | -14.445 | | 5.207375 |
| HIST1H4C | | | -1.12869 | | 0.000194 | | 3.35E-06 | | | | -14.1084 | | 5.039303 |
| MCM2 | | | -1.13336 | | 0.000116 | | 1.64E-06 | | | | -15.7531 | | 5.824826 |
| MSN | | | -1.13517 | | 0.001328 | | 4.53E-05 | | | | -9.36637 | | 2.136408 |
| SMC4 | | | -1.13786 | | 0.001952 | | 7.57E-05 | | | | -8.62271 | | 1.561158 |
| PRSS3 | | | -1.13849 | | 3.06E-05 | | 2.44E-07 | | | | -21.0954 | | 7.88338 |
| NDC80 | | | -1.1391 | | 0.000862 | | 2.55E-05 | | | | -10.2645 | | 2.779133 |
| HMGB2 | | | -1.14254 | | 0.009671 | | 0.000637 | | | | -6.0356 | | -0.82713 |
| ASF1B | | | -1.14333 | | 0.001106 | | 3.60E-05 | | | | -9.71727 | | 2.393903 |
| ARPC1B | | | -1.14452 | | 0.002958 | | 0.000135 | | | | -7.84597 | | 0.912754 |
| FRMD6 | | | -1.14623 | | 0.030669 | | 0.002889 | | | | -4.58447 | | -2.5096 |
| PHF19 | | | -1.14722 | | 0.000893 | | 2.67E-05 | | | | -10.1906 | | 2.72823 |
| PTGER1 | | | -1.15025 | | 0.002177 | | 8.78E-05 | | | | -8.41776 | | 1.395045 |
| EXO1 | | | -1.1519 | | 0.000463 | | 1.13E-05 | | | | -11.6809 | | 3.694279 |
| SUGCT | | | -1.15232 | | 0.000192 | | 3.31E-06 | | | | -14.1363 | | 5.053391 |
| PRR11 | | | -1.15233 | | 0.00054 | | 1.37E-05 | | | | -11.3211 | | 3.472182 |
| GTSE1 | | | -1.15655 | | 9.42E-05 | | 1.24E-06 | | | | -16.4375 | | 6.12713 |
| SHROOM3 | | | -1.15723 | | 0.000993 | | 3.10E-05 | | | | -9.95266 | | 2.561983 |
| FN1 | | | -1.15877 | | 0.013234 | | 0.000954 | | | | -5.6214 | | -1.27836 |
| CTHRC1 | | | -1.1606 | | 6.86E-05 | | 7.92E-07 | | | | -17.6165 | | 6.618169 |
| DDX39A | | | -1.16546 | | 2.50E-05 | | 1.81E-07 | | | | -22.0703 | | 8.196735 |
| STIL | | | -1.16693 | | 0.000249 | | 4.78E-06 | | | | -13.3515 | | 4.646098 |
| IRX3 | | | -1.16811 | | 5.83E-05 | | 6.14E-07 | | | | -18.3185 | | 6.894201 |
| POLQ | | | -1.1695 | | 5.67E-05 | | 5.93E-07 | | | | -18.4171 | | 6.932045 |
| GSTO1 | | | -1.16993 | | 0.000186 | | 3.17E-06 | | | | -14.2337 | | 5.102325 |
| INO80C | | | -1.17487 | | 0.000477 | | 1.17E-05 | | | | -11.6151 | | 3.654188 |
| STEAP3 | | | -1.17776 | | 0.00032 | | 6.77E-06 | | | | -12.6479 | | 4.260211 |
| SPHK1 | | | -1.17803 | | 0.000154 | | 2.44E-06 | | | | -14.8168 | | 5.388529 |
| CERCAM | | | -1.17865 | | 0.000536 | | 1.36E-05 | | | | -11.343 | | 3.48584 |
| SHB | | | -1.18219 | | 0.000108 | | 1.49E-06 | | | | -15.996 | | 5.933675 |
| INPP4B | | | -1.18382 | | 5.24E-06 | | 1.33E-08 | | | | -32.7871 | | 10.83311 |
| DCK | | | -1.18542 | | 0.000142 | | 2.18E-06 | | | | -15.0753 | | 5.511732 |
| MCM10 | | | -1.18592 | | 1.82E-05 | | 1.13E-07 | | | | -23.7142 | | 8.690911 |
| SDC4 | | | -1.1869 | | 3.80E-05 | | 3.26E-07 | | | | -20.1797 | | 7.573901 |
| LXN | | | -1.18939 | | 7.60E-05 | | 9.26E-07 | | | | -17.1993 | | 6.448482 |
| MTHFD1L | | | -1.19035 | | 0.000341 | | 7.46E-06 | | | | -12.4578 | | 4.152366 |
| TNS3 | | | -1.19155 | | 0.000124 | | 1.82E-06 | | | | -15.5096 | | 5.713957 |
| S100A3 | | | -1.19278 | | 0.002041 | | 8.04E-05 | | | | -8.5388 | | 1.493564 |
| SIRPA | | | -1.19309 | | 0.001129 | | 3.71E-05 | | | | -9.67321 | | 2.362037 |
| TMSB15A | | | -1.19438 | | 0.000727 | | 2.02E-05 | | | | -10.6566 | | 3.043762 |
| ADCY7 | | | -1.19526 | | 6.08E-05 | | 6.55E-07 | | | | -18.1363 | | 6.823672 |
| COL1A1 | | | -1.19899 | | 0.016809 | | 0.001311 | | | | -5.3103 | | -1.63206 |
| PLOD2 | | | -1.20086 | | 0.000902 | | 2.72E-05 | | | | -10.1649 | | 2.710433 |
| AES | | | -1.21137 | | 0.002902 | | 0.000132 | | | | -7.87826 | | 0.940765 |
| HERC4 | | | -1.21323 | | 0.000999 | | 3.12E-05 | | | | -9.941 | | 2.553741 |
| KIF2C | | | -1.21367 | | 0.000185 | | 3.15E-06 | | | | -14.2453 | | 5.10815 |
| SLIT2 | | | -1.2147 | | 0.000451 | | 1.08E-05 | | | | -11.7509 | | 3.736738 |
| RANGAP1 | | | -1.21648 | | 0.001038 | | 3.29E-05 | | | | -9.85789 | | 2.494746 |
| ANXA2 | | | -1.2214 | | 0.012597 | | 0.000898 | | | | -5.6821 | | -1.21085 |
| CDK2 | | | -1.22272 | | 6.45E-05 | | 7.14E-07 | | | | -17.9003 | | 6.731146 |
| TSPAN4 | | | -1.22343 | | 0.001137 | | 3.75E-05 | | | | -9.65545 | | 2.349151 |
| FTSJ1 | | | -1.22392 | | 3.11E-05 | | 2.48E-07 | | | | -21.0429 | | 7.866064 |
| PDGFC | | | -1.22401 | | 0.009583 | | 0.000628 | | | | -6.05071 | | -0.81108 |
| DPYSL3 | | | -1.22631 | | 0.00061 | | 1.62E-05 | | | | -11.0356 | | 3.29109 |
| MCM5 | | | -1.22716 | | 7.18E-05 | | 8.52E-07 | | | | -17.4217 | | 6.53948 |
| RHBDF2 | | | -1.22745 | | 0.001012 | | 3.19E-05 | | | | -9.91065 | | 2.53225 |
| E2F7 | | | -1.2311 | | 0.000268 | | 5.37E-06 | | | | -13.1137 | | 4.517978 |
| GREM2 | | | -1.23283 | | 6.91E-06 | | 2.41E-08 | | | | -29.9722 | | 10.25657 |
| HEG1 | | | -1.23896 | | 0.002187 | | 8.86E-05 | | | | -8.40626 | | 1.385623 |
| MMP2 | | | -1.24279 | | 0.000561 | | 1.44E-05 | | | | -11.2326 | | 3.416504 |
| PGBD3 | | | -1.24381 | | 3.31E-05 | | 2.74E-07 | | | | -20.7248 | | 7.759984 |
| TIMP2 | | | -1.25398 | | 0.000328 | | 7.04E-06 | | | | -12.5715 | | 4.217086 |
| HSPB11 | | | -1.25553 | | 0.001063 | | 3.41E-05 | | | | -9.80531 | | 2.457197 |
| SLC7A1 | | | -1.25927 | | 1.50E-05 | | 8.42E-08 | | | | -24.7945 | | 8.994493 |
| UGDH | | | -1.26392 | | 0.001352 | | 4.67E-05 | | | | -9.32342 | | 2.104299 |
| NOV | | | -1.26475 | | 8.66E-05 | | 1.10E-06 | | | | -16.751 | | 6.261226 |
| UBE2E3 | | | -1.26662 | | 0.000109 | | 1.51E-06 | | | | -15.9552 | | 5.915492 |
| TRMT5 | | | -1.26669 | | 0.000923 | | 2.82E-05 | | | | -10.1022 | | 2.666916 |
| DIAPH3 | | | -1.26936 | | 5.82E-05 | | 6.12E-07 | | | | -18.3281 | | 6.89788 |
| TMPO | | | -1.27141 | | 0.001436 | | 5.05E-05 | | | | -9.2055 | | 2.015465 |
| LACTB | | | -1.27283 | | 4.37E-05 | | 3.92E-07 | | | | -19.6223 | | 7.377776 |
| CDH2 | | | -1.27843 | | 0.000164 | | 2.69E-06 | | | | -14.5972 | | 5.282096 |
| SPDL1 | | | -1.28379 | | 2.32E-05 | | 1.60E-07 | | | | -22.4871 | | 8.32594 |
| GALNT5 | | | -1.28735 | | 0.000194 | | 3.38E-06 | | | | -14.088 | | 5.028948 |
| FJX1 | | | -1.28786 | | 6.07E-05 | | 6.53E-07 | | | | -18.1445 | | 6.826859 |
| AHNAK2 | | | -1.28929 | | 0.001937 | | 7.48E-05 | | | | -8.64027 | | 1.575229 |
| KIF4A | | | -1.29237 | | 1.47E-05 | | 8.24E-08 | | | | -24.8785 | | 9.017448 |
| H2AFZ | | | -1.29494 | | 0.000971 | | 3.02E-05 | | | | -9.99361 | | 2.590853 |
| CSRNP1 | | | -1.29816 | | 0.000948 | | 2.94E-05 | | | | -10.0401 | | 2.623474 |
| APOBEC3B | | | -1.30135 | | 0.000165 | | 2.70E-06 | | | | -14.5846 | | 5.275962 |
| VAMP8 | | | -1.30144 | | 7.05E-05 | | 8.31E-07 | | | | -17.4877 | | 6.566239 |
| LMNA | | | -1.3015 | | 8.45E-05 | | 1.06E-06 | | | | -16.8389 | | 6.298372 |
| WISP1 | | | -1.30435 | | 0.000192 | | 3.29E-06 | | | | -14.1479 | | 5.059229 |
| CKAP2 | | | -1.30505 | | 0.001903 | | 7.32E-05 | | | | -8.67114 | | 1.599911 |
| TUBA1A | | | -1.3094 | | 0.002859 | | 0.000129 | | | | -7.90404 | | 0.963053 |
| CRIP2 | | | -1.3099 | | 8.80E-06 | | 3.50E-08 | | | | -28.3289 | | 9.887032 |
| PPP1R14B | | | -1.31049 | | 0.00054 | | 1.37E-05 | | | | -11.323 | | 3.473359 |
| TMEM158 | | | -1.31094 | | 0.00101 | | 3.17E-05 | | | | -9.91876 | | 2.537998 |
| SPC24 | | | -1.32344 | | 0.002415 | | 0.000102 | | | | -8.218 | | 1.229777 |
| ISLR | | | -1.32424 | | 0.000137 | | 2.07E-06 | | | | -15.1959 | | 5.568496 |
| GLT8D2 | | | -1.33314 | | 0.000121 | | 1.73E-06 | | | | -15.6189 | | 5.763958 |
| CCL2 | | | -1.33591 | | 0.000111 | | 1.54E-06 | | | | -15.9029 | | 5.892138 |
| S100A16 | | | -1.33594 | | 7.27E-05 | | 8.73E-07 | | | | -17.358 | | 6.513539 |
| CENPN | | | -1.3396 | | 2.37E-05 | | 1.67E-07 | | | | -22.3483 | | 8.283218 |
| FSCN1 | | | -1.34003 | | 5.28E-05 | | 5.17E-07 | | | | -18.8067 | | 7.079528 |
| NT5DC2 | | | -1.34088 | | 3.19E-06 | | 5.62E-09 | | | | -37.3522 | | 11.64083 |
| CLDN11 | | | -1.34612 | | 9.07E-06 | | 3.70E-08 | | | | -28.0941 | | 9.832047 |
| DACT1 | | | -1.34646 | | 3.17E-05 | | 2.58E-07 | | | | -20.917 | | 7.824288 |
| CENPK | | | -1.34795 | | 0.000518 | | 1.30E-05 | | | | -11.4167 | | 3.531841 |
| SH2D4A | | | -1.34907 | | 1.39E-05 | | 7.55E-08 | | | | -25.2118 | | 9.107611 |
| EXT1 | | | -1.35024 | | 0.002249 | | 9.21E-05 | | | | -8.35303 | | 1.341864 |
| UBE2T | | | -1.35938 | | 6.26E-05 | | 6.85E-07 | | | | -18.0122 | | 6.775179 |
| FAM111A | | | -1.36177 | | 0.00032 | | 6.78E-06 | | | | -12.6453 | | 4.258794 |
| STX1A | | | -1.36921 | | 2.22E-05 | | 1.47E-07 | | | | -22.778 | | 8.414495 |
| MYO1B | | | -1.36972 | | 0.000285 | | 5.78E-06 | | | | -12.9637 | | 4.435993 |
| SLC16A3 | | | -1.37452 | | 0.00038 | | 8.67E-06 | | | | -12.1685 | | 3.985108 |
| GPSM2 | | | -1.38252 | | 5.42E-05 | | 5.43E-07 | | | | -18.6654 | | 7.026425 |
| SOCS5 | | | -1.38532 | | 0.002371 | | 9.87E-05 | | | | -8.25841 | | 1.26348 |
| PLP2 | | | -1.38734 | | 0.002814 | | 0.000126 | | | | -7.93354 | | 0.98849 |
| IGFBP6 | | | -1.38862 | | 0.000197 | | 3.45E-06 | | | | -14.0436 | | 5.006475 |
| LRRC17 | | | -1.38934 | | 3.90E-05 | | 3.38E-07 | | | | -20.0666 | | 7.534594 |
| S100A13 | | | -1.39005 | | 0.000806 | | 2.33E-05 | | | | -10.4137 | | 2.880955 |
| FGF5 | | | -1.39164 | | 2.23E-05 | | 1.50E-07 | | | | -22.7219 | | 8.397524 |
| CSTF3 | | | -1.3938 | | 8.10E-05 | | 1.01E-06 | | | | -16.9657 | | 6.351555 |
| CDK1 | | | -1.39655 | | 8.61E-05 | | 1.09E-06 | | | | -16.7787 | | 6.272942 |
| NCAPD2 | | | -1.39698 | | 2.23E-05 | | 1.49E-07 | | | | -22.7264 | | 8.398895 |
| AK5 | | | -1.40468 | | 1.42E-05 | | 7.71E-08 | | | | -25.1275 | | 9.084943 |
| CCL26 | | | -1.40475 | | 0.000168 | | 2.78E-06 | | | | -14.5197 | | 5.244157 |
| FEN1 | | | -1.41214 | | 1.07E-05 | | 4.74E-08 | | | | -27.0514 | | 9.580848 |
| FBXO5 | | | -1.4135 | | 0.00013 | | 1.92E-06 | | | | -15.37 | | 5.6496 |
| ECT2 | | | -1.41491 | | 0.000339 | | 7.40E-06 | | | | -12.4731 | | 4.1611 |
| PPFIBP1 | | | -1.41541 | | 0.000363 | | 8.11E-06 | | | | -12.2961 | | 4.059318 |
| ZWINT | | | -1.41904 | | 6.10E-05 | | 6.60E-07 | | | | -18.1179 | | 6.816507 |
| RAPH1 | | | -1.42416 | | 0.000756 | | 2.13E-05 | | | | -10.5616 | | 2.980465 |
| TACC3 | | | -1.43329 | | 1.17E-05 | | 5.81E-08 | | | | -26.2286 | | 9.374142 |
| RAB27B | | | -1.43504 | | 0.000395 | | 9.09E-06 | | | | -12.0789 | | 3.932489 |
| RAD51AP1 | | | -1.43693 | | 0.000157 | | 2.53E-06 | | | | -14.7322 | | 5.347692 |
| LMNB2 | | | -1.43774 | | 0.000256 | | 5.02E-06 | | | | -13.2494 | | 4.591383 |
| ITGA2 | | | -1.4386 | | 5.68E-06 | | 1.61E-08 | | | | -31.8714 | | 10.65284 |
| LARP6 | | | -1.44585 | | 3.79E-06 | | 7.37E-09 | | | | -35.8529 | | 11.39109 |
| KIF20B | | | -1.44952 | | 0.000559 | | 1.44E-05 | | | | -11.2437 | | 3.423495 |
| TRIOBP | | | -1.45001 | | 0.00012 | | 1.70E-06 | | | | -15.6603 | | 5.782804 |
| RASA1 | | | -1.45006 | | 0.000734 | | 2.05E-05 | | | | -10.6319 | | 3.027403 |
| RAB3B | | | -1.45193 | | 4.92E-05 | | 4.69E-07 | | | | -19.0856 | | 7.183073 |
| KIF11 | | | -1.45197 | | 0.001257 | | 4.23E-05 | | | | -9.47118 | | 2.21421 |
| BDNF | | | -1.47041 | | 0.000219 | | 4.02E-06 | | | | -13.7153 | | 4.837803 |
| TTK | | | -1.4741 | | 2.23E-05 | | 1.51E-07 | | | | -22.6811 | | 8.385156 |
| POC1A | | | -1.47575 | | 0.000429 | | 1.01E-05 | | | | -11.8832 | | 3.816317 |
| FOXQ1 | | | -1.4809 | | 0.000231 | | 4.35E-06 | | | | -13.551 | | 4.751854 |
| ITGA3 | | | -1.48766 | | 0.000444 | | 1.06E-05 | | | | -11.7907 | | 3.760774 |
| TGM2 | | | -1.49248 | | 6.54E-05 | | 7.27E-07 | | | | -17.8487 | | 6.710754 |
| CAP2 | | | -1.49453 | | 4.70E-05 | | 4.41E-07 | | | | -19.2659 | | 7.249139 |
| DNAJC9 | | | -1.49773 | | 8.87E-06 | | 3.54E-08 | | | | -28.2741 | | 9.874265 |
| IL7R | | | -1.49866 | | 6.21E-05 | | 6.76E-07 | | | | -18.0506 | | 6.790227 |
| TAGLN2 | | | -1.49922 | | 0.003715 | | 0.000182 | | | | -7.46759 | | 0.577296 |
| PSG3 | | | -1.5001 | | 4.49E-05 | | 4.13E-07 | | | | -19.4664 | | 7.321843 |
| MSANTD3 | | | -1.50015 | | 0.001595 | | 5.81E-05 | | | | -9.00053 | | 1.858626 |
| LINC01119 | | | -1.50123 | | 1.10E-05 | | 5.06E-08 | | | | -26.7881 | | 9.515533 |
| FOXD1 | | | -1.50455 | | 0.000109 | | 1.49E-06 | | | | -15.9861 | | 5.929265 |
| GNG11 | | | -1.50876 | | 0.00188 | | 7.21E-05 | | | | -8.6924 | | 1.616871 |
| STAMBPL1 | | | -1.50941 | | 0.000457 | | 1.10E-05 | | | | -11.7163 | | 3.715791 |
| GPER1 | | | -1.51384 | | 9.03E-05 | | 1.17E-06 | | | | -16.5906 | | 6.192967 |
| MAPKAPK3 -1.51614 | | | | | 0.002285 | | 9.39E-05 | | | | -8.32676 | | 1.320174 |
| CDC25B -1.51808 | | | | | 0.003576 | | 0.000173 | | | | -7.52749 | | 0.631313 |
| PKM -1.5296 | | | | | 0.001689 | | 6.29E-05 | | | | -8.88657 | | 1.770065 |
| NEK2 -1.52966 | | | | | 0.0045 | | 0.000235 | | | | -7.15413 | | 0.288833 |
| NUDT1 | | | -1.5322 | | 5.02E-05 | | | 4.81E-07 | | | -19.0135 | | 7.156456 |
| PBK | | | -1.53674 | | 5.33E-05 | | | 5.27E-07 | | | -18.7506 | | 7.058495 |
| SNAR-A1 | | | -1.53699 | | 0.001063 | | | 3.40E-05 | | | -9.80601 | | 2.457695 |
| SH3RF1 | | | -1.54013 | | 0.000155 | | | 2.47E-06 | | | -14.7855 | | 5.373445 |
| MGLL | | | -1.5407 | | 0.000434 | | | 1.02E-05 | | | -11.8566 | | 3.800381 |
| DIAPH1 | | | -1.54474 | | 0.000246 | | | 4.73E-06 | | | -13.3735 | | 4.657843 |
| HAPLN1 | | | -1.54911 | | 0.000218 | | | 3.99E-06 | | | -13.7314 | | 4.846128 |
| GDF5 | | | -1.54946 | | 6.75E-06 | | | 2.28E-08 | | | -30.2346 | | 10.31321 |
| RRM2 | | | -1.54951 | | 6.00E-05 | | | 6.42E-07 | | | -18.1928 | | 6.845622 |
| ASAP2 | | | -1.5555 | | 7.16E-06 | | | 2.56E-08 | | | -29.7068 | | 10.19864 |
| PLK4 | | | -1.55607 | | 2.87E-05 | | | 2.17E-07 | | | -21.4762 | | 8.007684 |
| BCAT1 | | | -1.55791 | | 0.000103 | | | 1.40E-06 | | | -16.1373 | | 5.996162 |
| PNP | | | -1.56145 | | 2.43E-05 | | | 1.74E-07 | | | -22.2015 | | 8.237711 |
| RACGAP1 | | | -1.56965 | | 7.63E-05 | | | 9.33E-07 | | | -17.1812 | | 6.441006 |
| RGMB | | | -1.56992 | | 0.000225 | | | 4.18E-06 | | | -13.632 | | 4.794343 |
| DAB2 | | | -1.57066 | | 4.43E-05 | | | 4.04E-07 | | | -19.5293 | | 7.344461 |
| CLIC1 | | | -1.57595 | | 6.75E-06 | | | 2.27E-08 | | | -30.2499 | | 10.31649 |
| FAM83D | | | -1.58571 | | 0.000135 | | | 2.02E-06 | | | -15.2523 | | 5.594879 |
| RRM1 | | | -1.58668 | | 9.44E-05 | | | 1.25E-06 | | | -16.4179 | | 6.118655 |
| MT1E | | | -1.59061 | | 0.001522 | | | 5.48E-05 | | | -9.08646 | | 1.924756 |
| KIFC1 | | | -1.59074 | | 7.09E-05 | | | 8.38E-07 | | | -17.4649 | | 6.557015 |
| FXYD5 | | | -1.59342 | | 3.15E-05 | | | 2.55E-07 | | | -20.9544 | | 7.836723 |
| ADGRG1 | | | -1.59632 | | 5.67E-05 | | | 5.92E-07 | | | -18.4204 | | 6.933329 |
| KCNK2 | | | -1.59896 | | 0.000168 | | | 2.78E-06 | | | -14.526 | | 5.247246 |
| TUBA1C | | | -1.60627 | | 0.000406 | | | 9.46E-06 | | | -12.0045 | | 3.888534 |
| NEK7 | | | -1.60896 | | 0.001019 | | | 3.22E-05 | | | -9.89396 | | 2.520405 |
| MTMR11 | | | -1.61135 | | 5.48E-05 | | | 5.58E-07 | | | -18.5896 | | 6.997744 |
| CENPE | | | -1.61532 | | 0.000262 | | | 5.23E-06 | | | -13.1659 | | 4.546302 |
| CDCA8 | | | -1.61668 | | 4.13E-05 | | | 3.67E-07 | | | -19.8137 | | 7.445824 |
| PSG5 | | | -1.61868 | | 1.43E-05 | | | 7.82E-08 | | | -25.0749 | | 9.070742 |
| SMAGP | | | -1.6203 | | 2.23E-05 | | | 1.49E-07 | | | -22.741 | | 8.403302 |
| IGFBP3 | | | -1.62175 | | 5.50E-05 | | | 5.66E-07 | | | -18.5495 | | 6.982538 |
| AURKB | | | -1.62178 | | 5.28E-05 | | | 5.18E-07 | | | -18.8016 | | 7.077614 |
| ESM1 | | | -1.63829 | | 3.06E-05 | | | 2.40E-07 | | | -21.1373 | | 7.897202 |
| DBNDD2 | | | -1.64132 | | 7.96E-06 | | | 3.00E-08 | | | -28.9941 | | 10.03979 |
| MAD2L1 | | | -1.64668 | | 0.001302 | | | 4.44E-05 | | | -9.3995 | | 2.16108 |
| PAMR1 | | | -1.64915 | | 9.31E-05 | | | 1.23E-06 | | | -16.4767 | | 6.144026 |
| FOSL1 | | | -1.65201 | | 0.000159 | | | 2.57E-06 | | | -14.7013 | | 5.33276 |
| FHL2 | | | -1.65227 | | 5.57E-06 | | | 1.52E-08 | | | -32.1393 | | 10.70627 |
| SGK1 | | | -1.65348 | | 6.75E-06 | | | 2.28E-08 | | | -30.2282 | | 10.31184 |
| CDC45 | | | -1.66085 | | 2.27E-05 | | | 1.55E-07 | | | -22.5941 | | 8.358659 |
| NREP | | | -1.66209 | | 0.000833 | | | 2.44E-05 | | | -10.3372 | | 2.828929 |
| EFHD2 | | | -1.66521 | | 0.000321 | | | 6.83E-06 | | | -12.6315 | | 4.250986 |
| SH3BGRL3 | | | -1.66524 | | 2.34E-05 | | | 1.63E-07 | | | -22.4179 | | 8.304677 |
| TNFRSF11B -1.66533 | | | | | 0.000279 | | | 5.64E-06 | | | -13.0141 | | 4.463618 |
| SERTAD4 -1.66557 | | | | | 0.00028 | | | 5.67E-06 | | | -13.004 | | 4.458093 |
| FMN2 -1.6702 | | | | | 5.68E-06 | | | 1.61E-08 | | | -31.8736 | | 10.65328 |
| SERPINE2 -1.67277 | | | | | 1.98E-05 | | | 1.28E-07 | | | -23.2764 | | 8.563266 |
| DLGAP5 -1.67299 | | | | | 8.80E-05 | | | 1.14E-06 | | | -16.6678 | | 6.225915 |
| POSTN -1.67345 | | | | | 0.000787 | | | 2.26E-05 | | | -10.4688 | | 2.918165 |
| CCDC34 | | | -1.67822 | | 4.49E-05 | | | 4.15E-07 | | | -19.4475 | | 7.315006 |
| GPR1 | | | -1.68495 | | 5.48E-05 | | | 5.56E-07 | | | -18.5994 | | 7.001476 |
| SLC7A5 | | | -1.69067 | | 0.000218 | | | 3.97E-06 | | | -13.7411 | | 4.851179 |
| DSP | | | -1.69434 | | 4.08E-06 | | | 8.43E-09 | | | -35.1351 | | 11.26638 |
| KDELR3 | | | -1.69557 | | 0.000164 | | | 2.67E-06 | | | -14.6128 | | 5.289691 |
| MCM7 | | | -1.6957 | | 9.60E-05 | | | 1.28E-06 | | | -16.3578 | | 6.092602 |
| MEG3 | | | -1.70039 | | 0.000298 | | | 6.16E-06 | | | -12.836 | | 4.36541 |
| PFKP | | | -1.70423 | | 0.000337 | | | 7.35E-06 | | | -12.4864 | | 4.168692 |
| OIP5 | | | -1.7185 | | 0.000215 | | | 3.87E-06 | | | -13.7996 | | 4.881465 |
| TRPV2 | | | -1.71931 | | 3.75E-06 | | | 7.23E-09 | | | -35.9589 | | 11.40922 |
| GABBR2 | | | -1.71961 | | 0.000171 | | | 2.83E-06 | | | -14.479 | | 5.224123 |
| CKAP2L | | | -1.72137 | | 1.60E-05 | | | 9.09E-08 | | | -24.5072 | | 8.915284 |
| CARD10 | | | -1.72868 | | 2.03E-06 | | | 2.74E-09 | | | -41.6373 | | 12.28268 |
| CENPM | | | -1.73008 | | 5.84E-05 | | | 6.19E-07 | | | -18.2947 | | 6.88504 |
| TMEM173 | | | -1.73095 | | 3.58E-05 | | | 3.05E-07 | | | -20.3835 | | 7.644122 |
| KIF23 | | | -1.73109 | | 8.69E-06 | | | 3.38E-08 | | | -28.4741 | | 9.920753 |
| ARHGEF3 | | | -1.73595 | | 3.06E-05 | | | 2.40E-07 | | | -21.1478 | | 7.900631 |
| CENPA | | | -1.74251 | | 1.08E-06 | | | 1.09E-09 | | | -47.8703 | | 13.05906 |
| ETV5 | | | -1.74402 | | 2.08E-05 | | | 1.36E-07 | | | -23.0503 | | 8.496243 |
| CD59 | | | -1.74839 | | 5.65E-05 | | | 5.85E-07 | | | -18.4562 | | 6.947011 |
| CTSK | | | -1.75582 | | 0.000118 | | | 1.68E-06 | | | -15.6955 | | 5.798745 |
| ELN | | | -1.75818 | | 0.002187 | | | 8.85E-05 | | | -8.40763 | | 1.386743 |
| PRR16 | | | -1.7789 | | 7.05E-05 | | | 8.29E-07 | | | -17.4943 | | 6.568898 |
| SHISA2 | | | -1.78425 | | 2.83E-06 | | | 4.63E-09 | | | -38.4605 | | 11.81661 |
| NCAPG | | | -1.78539 | | 0.000366 | | | 8.25E-06 | | | -12.2639 | | 4.040704 |
| PAFAH1B3 | | | -1.81087 | | 0.000225 | | | 4.18E-06 | | | -13.6321 | | 4.794394 |
| BUB1 | | | -1.81544 | | 8.74E-05 | | | 1.12E-06 | | | -16.7038 | | 6.241211 |
| HJURP | | | -1.81948 | | 4.43E-06 | | | 9.81E-09 | | | -34.3399 | | 11.1241 |
| SYNJ2 | | | -1.82683 | | 5.67E-05 | | | 5.92E-07 | | | -18.4199 | | 6.933108 |
| PORCN | | | -1.83205 | | 3.95E-05 | | | 3.43E-07 | | | -20.0217 | | 7.518925 |
| CRABP2 | | | -1.83638 | | 1.89E-05 | | | 1.20E-07 | | | -23.4971 | | 8.627962 |
| SLC9A7 | | | -1.83714 | | 7.02E-06 | | | 2.47E-08 | | | -29.8557 | | 10.23122 |
| CDCA3 | | | -1.8384 | | 4.65E-06 | | | 1.07E-08 | | | -33.8885 | | 11.04135 |
| TPX2 | | | -1.84359 | | 3.06E-05 | | | 2.41E-07 | | | -21.1299 | | 7.894742 |
| STMN1 | | | -1.84489 | | 5.16E-06 | | | 1.28E-08 | | | -32.9892 | | 10.87201 |
| MOK | | | -1.85024 | | 5.70E-05 | | | 5.97E-07 | | | -18.3966 | | 6.924182 |
| ENG | | | -1.85943 | | 2.41E-05 | | | 1.70E-07 | | | -22.2749 | | 8.260502 |
| UBASH3B | | | -1.86271 | | 1.34E-05 | | | 7.05E-08 | | | -25.4731 | | 9.177314 |
| TRIP13 | | | -1.86503 | | 6.08E-06 | | | 1.90E-08 | | | -31.0826 | | 10.49204 |
| BDKRB1 | | | -1.86852 | | 1.39E-05 | | | 7.52E-08 | | | -25.2247 | | 9.111076 |
| DCBLD2 | | | -1.87076 | | 0.001497 | | | 5.35E-05 | | | -9.12105 | | 1.951225 |
| KRT7 | | | -1.87342 | | 1.42E-06 | | | 1.66E-09 | | | -44.8964 | | 12.70927 |
| CDCA5 | | | -1.88026 | | 0.000261 | | | 5.20E-06 | | | -13.1768 | | 4.552215 |
| LYPD6B | | | -1.88067 | | 4.37E-05 | | | 3.90E-07 | | | -19.6313 | | 7.381018 |
| EZR | | | -1.88101 | | 1.14E-05 | | | 5.28E-08 | | | -26.618 | | 9.472939 |
| CENPW | | | -1.8826 | | 1.39E-05 | | | 7.53E-08 | | | -25.2173 | | 9.109097 |
| FOXM1 | | | -1.89267 | | 9.63E-06 | | | 4.12E-08 | | | -27.6317 | | 9.722095 |
| VEGFC | | | -1.90218 | | 0.000357 | | | 7.86E-06 | | | -12.3561 | | 4.093973 |
| SERINC2 | | | -1.91643 | | 0.000573 | | | 1.49E-05 | | | -11.1741 | | 3.379457 |
| GLIPR1 | | | -1.91713 | | 3.45E-06 | | | 6.32E-09 | | | -36.7028 | | 11.5344 |
| SMS | | | -1.9266 | | 4.45E-05 | | | 4.08E-07 | | | -19.4993 | | 7.333703 |
| GINS2 | | | -1.93151 | | 6.60E-06 | | | 2.12E-08 | | | -30.5566 | | 10.38186 |
| TEK | | | -1.93913 | | 6.46E-06 | | | 2.06E-08 | | | -30.7008 | | 10.41231 |
| KCNMA1 | | | -1.94677 | | 0.00044 | | | 1.04E-05 | | | -11.82 | | 3.778426 |
| CCNA2 | | | -1.94718 | | 4.82E-06 | | | 1.13E-08 | | | -33.5925 | | 10.98628 |
| TBC1D2 | | | -1.95102 | | 9.42E-06 | | | 3.94E-08 | | | -27.8277 | | 9.76899 |
| DKK3 | | | -1.95939 | | 1.65E-05 | | | 9.56E-08 | | | -24.3226 | | 8.86382 |
| KRT81 | | | -1.96089 | | 0.000133 | | | 1.98E-06 | | | -15.3045 | | 5.619197 |
| ASPM | | | -1.96105 | | 0.000441 | | | 1.05E-05 | | | -11.8109 | | 3.772928 |
| KRTAP1-3 | | | -1.96714 | | 6.98E-05 | | | 8.12E-07 | | | -17.5489 | | 6.590959 |
| HMMR | | | -1.97051 | | 2.34E-05 | | | 1.64E-07 | | | -22.416 | | 8.304108 |
| ANLN | | | -1.98296 | | 0.000333 | | | 7.20E-06 | | | -12.5267 | | 4.191644 |
| LYPD1 | | | -1.99078 | | 0.00048 | | | 1.18E-05 | | | -11.5902 | | 3.638888 |
| MELK | | | -1.99947 | | 0.000533 | | | 1.35E-05 | | | -11.3563 | | 3.494205 |
| CRIP1 | | | -2.00206 | | 7.16E-06 | | | 2.59E-08 | | | -29.6448 | | 10.185 |
| DOCK10 | | | -2.00612 | | 0.00032 | | | 6.79E-06 | | | -12.6429 | | 4.257445 |
| CENPF | | | -2.01131 | | 3.51E-05 | | | 2.97E-07 | | | -20.4728 | | 7.674629 |
| MCUB | | | -2.01587 | | 4.54E-05 | | | 4.22E-07 | | | -19.3989 | | 7.297448 |
| TMEM106C -2.02287 | | | | | 1.08E-05 | | | 4.84E-08 | | | -26.9654 | | 9.559611 |
| PRRX2 -2.03138 | | | | | 0.002159 | | | 8.69E-05 | | | -8.43188 | | 1.406599 |
| FAM64A -2.03308 | | | | | 1.83E-05 | | | 1.15E-07 | | | -23.6636 | | 8.676304 |
| AEBP1 -2.0426 | | | | | 0.000332 | | | 7.17E-06 | | | -12.5362 | | 4.197073 |
| CEP55 -2.06237 | | | | | 5.70E-06 | | | 1.62E-08 | | | -31.8229 | | 10.6431 |
| S100A4 -2.06442 | | | | | 6.18E-05 | | | 6.71E-07 | | | -18.0706 | | 6.798032 |
| SH2B3 -2.06464 | | | | | 0.000246 | | | 4.69E-06 | | | -13.3917 | | 4.667534 |
| FLG -2.07434 | | | | | 2.34E-05 | | | 1.63E-07 | | | -22.4304 | | 8.308514 |
| PTGES -2.09037 | | | | | 0.000142 | | | 2.17E-06 | | | -15.0836 | | 5.515648 |
| TYMS -2.09471 | | | | | 0.000329 | | | 7.06E-06 | | | -12.5645 | | 4.213121 |
| NCEH1 -2.09934 | | | | | 1.67E-05 | | | 9.73E-08 | | | -24.2571 | | 8.845465 |
| FGF2 -2.10984 | | | | | 7.16E-06 | | | 2.62E-08 | | | -29.6051 | | 10.17627 |
| EGR1 -2.11107 | | | | | 0.000142 | | | 2.19E-06 | | | -15.0644 | | 5.506592 |
| MEDAG -2.11763 | | | | | 2.90E-06 | | | 4.93E-09 | | | -38.1061 | | 11.76118 |
| EMP3 -2.12389 | | | | | 0.000406 | | | 9.45E-06 | | | -12.0051 | | 3.888913 |
| APCDD1L -2.13035 | | | | | 7.05E-05 | | | 8.32E-07 | | | -17.4855 | | 6.565326 |
| GPNMB -2.16585 | | | | | 6.71E-05 | | | 7.63E-07 | | | -17.7181 | | 6.658823 |
| TNFRSF12A -2.20408 | | | | | 0.000112 | | | 1.56E-06 | | | -15.874 | | 5.879191 |
| AURKA -2.22394 | | | | | 1.24E-05 | | | 6.37E-08 | | | -25.8707 | | 9.281753 |
| CPA4 -2.22676 | | | | | 5.57E-06 | | | 1.51E-08 | | | -32.1739 | | 10.71314 |
| ITPR3 -2.22769 | | | | | 0.000145 | | | 2.25E-06 | | | -15.0021 | | 5.477054 |
| MLPH -2.24597 | | | | | 5.36E-06 | | | 1.38E-08 | | | -32.5966 | | 10.79615 |
| CDKN3 -2.25544 | | | | | 6.23E-06 | | | 1.97E-08 | | | -30.9113 | | 10.45643 |
| GPR68 -2.26224 | | | | | 5.84E-07 | | | 4.96E-10 | | | -53.882 | | 13.6698 |
| OCIAD2 -2.26728 | | | | | 3.75E-06 | | | 7.12E-09 | | | -36.0476 | | 11.42434 |
| PHLDA1 -2.26792 | | | | | 5.47E-05 | | | 5.53E-07 | | | -18.6154 | | 7.007518 |
| CDC20 -2.27392 | | | | | 0.000111 | | | 1.54E-06 | | | -15.913 | | 5.896657 |
| LPXN -2.27794 | | | | | 5.91E-06 | | | 1.74E-08 | | | -31.4784 | | 10.57338 |
| NUSAP1 -2.29044 | | | | | 8.48E-05 | | | 1.07E-06 | | | -16.8253 | | 6.292637 |
| MALL -2.30914 | | | | | 9.38E-06 | | | 3.90E-08 | | | -27.8636 | | 9.777518 |
| PTTG3P -2.31722 | | | | | 7.81E-05 | | | 9.60E-07 | | | -17.1049 | | 6.409469 |
| CCND1 -2.33961 | | | | | 7.16E-06 | | | 2.60E-08 | | | -29.6379 | | 10.1835 |
| GREM1 -2.34603 | | | | | 2.41E-05 | | | 1.71E-07 | | | -22.2567 | | 8.254866 |
| CCNB2 -2.34635 | | | | | 4.80E-07 | | | 3.34E-10 | | | -57.1755 | | 13.95822 |
| PLAUR -2.36687 | | | | | 4.72E-05 | | | 4.44E-07 | | | -19.2487 | | 7.242885 |
| ANKRD1 -2.39509 | | | | | 6.14E-05 | | | 6.66E-07 | | | -18.0914 | | 6.806146 |
| TOP2A -2.40104 | | | | | 4.42E-05 | | | 4.00E-07 | | | -19.558 | | 7.354772 |
| CD44 -2.40745 | | | | | 2.63E-05 | | | 1.93E-07 | | | -21.8531 | | 8.1283 |
| FAP -2.42823 | | | | | 3.06E-05 | | | 2.43E-07 | | | -21.1014 | | 7.885375 |
| RAC2 -2.43329 | | | | | 2.63E-05 | | | 1.93E-07 | | | -21.861 | | 8.130796 |
| TMEM200A -2.44953 | | | | | 1.67E-05 | | | 9.81E-08 | | | -24.2272 | | 8.837063 |
| MARCH4 -2.45329 | | | | | 7.08E-07 | | | 6.39E-10 | | | -51.8645 | | 13.47786 |
| BIRC5 -2.47003 | | | | | 7.52E-06 | | | 2.79E-08 | | | -29.3158 | | 10.1121 |
| NTN4 -2.47118 | | | | | 0.000152 | | | 2.41E-06 | | | -14.8418 | | 5.400502 |
| SLC20A1 -2.48421 | | | | | 1.17E-05 | | | 5.72E-08 | | | -26.2934 | | 9.390696 |
| PODXL -2.49154 | | | | | 1.72E-05 | | | 1.02E-07 | | | -24.0818 | | 8.79601 |
| EXOG -2.49189 | | | | | 7.23E-05 | | | 8.65E-07 | | | -17.3814 | | 6.523053 |
| FAM20C -2.50882 | | | | | 6.75E-06 | | | 2.31E-08 | | | -30.1675 | | 10.29877 |
| SCARA3 -2.54001 | | | | | 1.22E-05 | | | 6.16E-08 | | | -26.0001 | | 9.31534 |
| UBE2C -2.54585 | | | | | 5.66E-06 | | | 1.58E-08 | | | -31.962 | | 10.67097 |
| ARHGAP22 -2.57973 | | | | | 8.42E-06 | | | 3.26E-08 | | | -28.6309 | | 9.956942 |
| KLF2 | | | -2.59779 | | 1.17E-05 | | | 5.79E-08 | | | -26.2451 | | 9.378351 |
| DKK1 | | | -2.60623 | | 2.51E-05 | | | 1.82E-07 | | | -22.0554 | | 8.192058 |
| TK1 | | | -2.61759 | | 1.53E-06 | | | 1.98E-09 | | | -43.7407 | | 12.56349 |
| MT1A | | | -2.6266 | | 5.57E-06 | | | 1.53E-08 | | | -32.1102 | | 10.7005 |
| BHLHE40 | | | -2.63639 | | 5.84E-07 | | | 5.02E-10 | | | -53.7742 | | 13.65985 |
| KIF20A | | | -2.64458 | | 1.87E-06 | | | 2.49E-09 | | | -42.2311 | | 12.36407 |
| IER3 | | | -2.73645 | | 1.15E-05 | | | 5.40E-08 | | | -26.5233 | | 9.449055 |
| C15orf52 | | | -2.7398 | | 1.38E-06 | | | 1.58E-09 | | | -45.2416 | | 12.75169 |
| PRC1 | | | -2.74872 | | 1.74E-05 | | | 1.05E-07 | | | -23.9866 | | 8.768969 |
| C11orf87 | | | -2.77124 | | 1.47E-06 | | | 1.87E-09 | | | -44.1079 | | 12.61044 |
| PTTG1 | | | -2.80139 | | 1.08E-05 | | | 4.82E-08 | | | -26.987 | | 9.564942 |
| ABI3BP | | | -2.82884 | | 3.48E-05 | | | 2.92E-07 | | | -20.5208 | | 7.690969 |
| NGF | | | -2.8351 | | 8.24E-06 | | | 3.14E-08 | | | -28.7961 | | 9.994775 |
| NRG1 | | | -2.85287 | | 7.16E-06 | | | 2.57E-08 | | | -29.6768 | | 10.19206 |
| UHRF1 | | | -2.87281 | | 1.47E-06 | | | 1.87E-09 | | | -44.1121 | | 12.61097 |
| AXL | | | -2.89414 | | 4.49E-05 | | | 4.14E-07 | | | -19.4579 | | 7.318782 |
| TFPI2 | | | -3.02095 | | 5.04E-06 | | | 1.24E-08 | | | -33.1635 | | 10.9053 |
| SULF1 | | | -3.0306 | | 8.80E-06 | | | 3.49E-08 | | | -28.3407 | | 9.88978 |
| KIAA0101 | | | -3.10793 | | 1.15E-05 | | | 5.45E-08 | | | -26.486 | | 9.439625 |
| RGS4 | | | -3.11531 | | 1.82E-05 | | | 1.12E-07 | | | -23.7348 | | 8.696861 |
| MT2A | | | -3.16407 | | 2.98E-06 | | | 5.18E-09 | | | -37.815 | | 11.71511 |
| KRT34 | | | -3.24471 | | 1.36E-07 | | | 4.97E-11 | | | -76.193 | | 15.17206 |
| HMGA1 | | | -3.29229 | | 1.45E-05 | | | 7.96E-08 | | | -25.0082 | | 9.052709 |
| KRTAP1-5 | | | -3.38502 | | 9.44E-08 | | | 2.90E-11 | | | -82.6206 | | 15.45815 |
| NT5E | | | -3.42162 | | 5.37E-07 | | | 4.18E-10 | | | -55.2758 | | 13.79547 |
| IL6 | | | -3.53444 | | 2.72E-07 | | | 1.44E-10 | | | -64.8998 | | 14.53189 |
| NTM | | | -3.80405 | | 8.51E-08 | | | 1.41E-11 | | | -92.0987 | | 15.8032 |
| LOXL4 | | | -3.85519 | | 4.47E-07 | | | 2.95E-10 | | | -58.2515 | | 14.04629 |
| EVA1A | | | -3.9569 | | 2.69E-06 | | | 4.19E-09 | | | -39.0446 | | 11.90639 |
| SLC14A1 | | | -4.05613 | | 1.05E-07 | | | 3.43E-11 | | | -80.5819 | | 15.37254 |
| KRT19 | | | -4.07472 | | 8.51E-08 | | | 1.94E-11 | | | -87.8074 | | 15.65697 |
| PLAT | | | -4.10928 | | 8.51E-08 | | | 2.11E-11 | | | -86.7102 | | 15.61704 |
| SERPINE1 | | | -4.52771 | | 2.22E-05 | | | 1.47E-07 | | | -22.7889 | | 8.417803 |
| CEMIP | | | -4.65351 | | 8.51E-08 | | | 1.87E-11 | | | -88.2431 | | 15.67253 |

|>=1).
